# Supplementary material for: Catalytic undirected methylation of unactivated C(sp3)−H bonds suitable for complex molecules
Source: Nat Commun. 2024 Sep 27;15:8307. doi: 10.1038/s41467-024-52245-1 (PMC11437150; doi:10.1038/s41467-024-52245-1)
Supplement: Supplementary file 1 — Supplementary Information [file 41467_2024_52245_MOESM1_ESM.pdf]

Supplementary Information

Catalytic Undirected Methylation of Unactivated C(sp^3)−H Bonds Suitable for Complex Molecules

Jin-Fay Tan, Yi Cheng Kang and John F. Hartwig*

*Corresponding author. Email: jhartwig@berkeley.edu

Table of Contents

Supplementary Methods
1. General Information......3
1.1 Reagents and Solvents......3
1.2 Equipment and Instrumentation......3
1.3 Naming of Compounds......3
1.4 Reaction Set-up for Photochemistry......4

2. Preparation/Purchasing of Reagents and Substrates......5
2.1 Ligands......5
2.2 Photosensitisers, Photocatalysts......5
2.3 Bidentate Ligands......6
2.4 Peroxides, Peracetates, Perbenzoates, Peracids......6
2.5 Substrates......8

3. Reaction Development of C(sp^3)−H Methylation......25
3.1 Evaluation of Ligands and Nickel Metal Catalysts......25
3.2 Evaluation of Metal Catalysts......26
3.3 Evaluation of Photosensitisers and Wavelengths of Visible Light......27
3.4 Evaluation of Solvents and Peroxides......28
3.5 Evaluation of Oxidants......29
3.6 Evaluation of Concentrations and Temperatures......30
3.7 Evaluation of the Effects of Various Acidic Additives......31
3.8 Evaluation of Deuteration of Tertiary C−H bond in MeCN-d_3......32
3.9 Evaluation of the Effects of Various Bidentate Additives......33
3.10 Variations of the Stoichiometries of 2,4-Pentanedione......34
3.11 Evaluation of Peroxides......35
3.12 Evaluation of Protecting Groups on Secondary Amine......36

4. General Procedures for the Methylation of Unactivated C(sp^3)−H Bonds......37
5. Substrate Scope of the Methylation of Unactivated C(sp^3)−H Bonds......38
6. Mechanistic Investigations......52
6.1 Studies of Potential Inhibition of the Reaction by the Products......52
6.2 The Effect of the Stoichiometries of [Ir] and [Ni] on the Conversion of Dicumyl Peroxide...53
6.3 Investigations of the Post-reaction Fate of Ir-F......54
6.4 Time-dependent Quantitative Changes of Dicumyl Peroxide, **1a** and **2a**......57
6.5 The Formation of 3-Methyl Acetylacetone under the Standard Condition......60
6.6 Investigation of Sequential Additions of $Ni(acac)_2$......65
6.7 Evaluation of the Conditions in Reference 10......66
6.8 Evaluation of the selectivity of the C–H methylation......68
6.9 Evaluation of the Rate of Consumption of Dicumyl Peroxide......69
7. NMR Spectra......74
Supplementary References......147

Supplementary Methods

1. General Information

1.1 Reagents and Solvents

All reagents purchased from commercial sources were used as received. Pentane, Et_2O, THF, CH_2Cl_2, toluene, and hexane were collected from a solvent purification system containing a 0.33 m column of activated alumina under nitrogen (solvent purification system purchased from Innovative Technologies, Newburyport, MA). Acetonitrile, 2,2,2-trifluoroethanol, ethyl acetate, and DMSO were degassed by three freeze-pump-thaw cycles prior to use. Deuterated solvents were purchased from Cambridge Isotope Laboratories and used as received.

1.2 Equipment and Instrumentation

All manipulations were performed under an inert atmosphere with a Schlenk manifold or a nitrogen glovebox unless otherwise noted. All glassware was dried at 150 °C for at least 12 hours before use. Flash column chromatography was conducted on an automated Teledyne ISCO CombiFlash® Rf or Rf+ system, with prepacked RediSep Gold® Silica Gel Disposable Flash Columns (20–40 microns). Reverse-phase chromatography was performed using C18 RediSep cartridges (43 g and 26 g). Thin-layer chromatography (TLC) was performed using Merck Kieselgel 60 F254 fluorescent-treated silica. Visualization of the developed chromatogram was performed under UV light or by staining with a solution of $KMnO_4$ or ceric ammonium molybdate, followed by heating. Organic solvents were concentrated under reduced pressure on a Büchi rotary evaporator using a water bath.

The ^{1}H, ^{13}C, and ^{19}F NMR spectra were recorded on a Bruker 400, 500, or 600 MHz spectrometer. The chemical shifts are given in parts per million (ppm). The ^{1}H chemical shifts are reported relative to the residual protiated solvent ($CHCl_3$ in $CDCl_3$: δ 7.260 ppm). The ^{13}C chemical shifts are reported in ppm relative to the deuterated solvent as a reference. Coupling constants (J) are given in Hertz (Hz), rounded to the nearest 0.1 Hz. The ^{1}H NMR spectra are reported as follows: ppm (multiplicity, coupling constants, number of protons). Abbreviations are as follows: s (singlet), d (doublet), t (triplet), q (quartet), m (multiplet), brs (broad singlet). High-resolution mass spectra were obtained on a high-resolution mass spectrometer at the QB3/Chemistry Mass Spectrometry Facility at UC Berkeley and on the Perkin Elmer AxION2 TOF MS operated by the LBNL Catalysis Facility. In general procedure for photochemical reactions, a Kessil H150W 450 nm blue LED lamp (DiCon Fiberoptics Inc.) or a Kessil PR160L 370 nm Gen 2 Violet LED lamp was used with light intensity set to 100%, except where noted otherwise. Reaction vessels were placed at approximately 2 cm from the light source. A cooling fan was used to maintain the temperature at 25 °C. LC-MS data were collected using a Waters ACQUITY UPLC I-Class PLUS equipped with an ACQUITY PDA detector and QDa Detector.

1.3 Naming of Compounds

Compound names were generated by ChemDraw 20.0 software (PerkinElmer), following the IUPAC nomenclature.

1.4. Reaction Set-up for Photochemistry

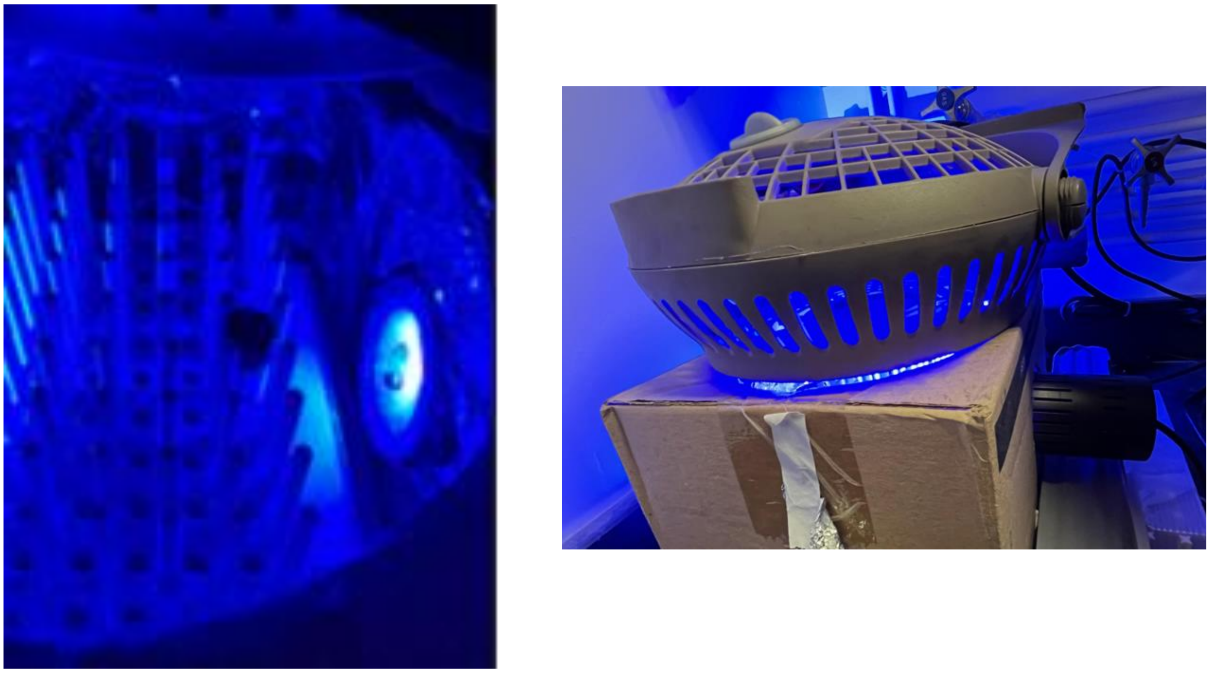

Supplementary Figure 1. General reaction set-up for Sections 3, 4, 5, 6

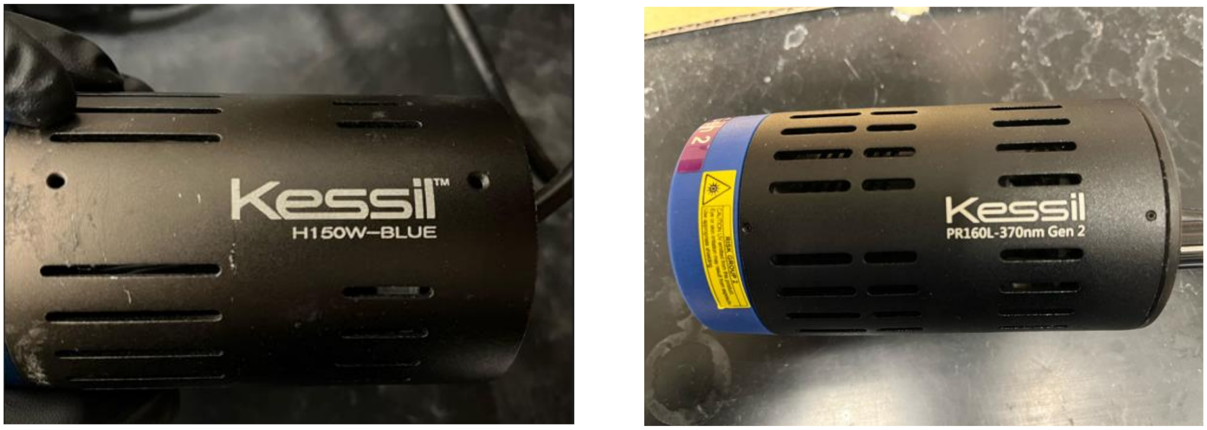

Supplementary Figure 2. Kessil LED lamps used for general procedures in Sections 3, 4, 5, 6

2. Preparation/Purchasing of Reagents and Substrates

2.1 Ligands

All ligands below were purchased from various commercial vendors.

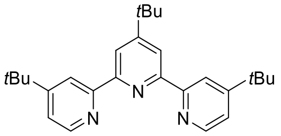

L1
Ambeed
CAS: 115091-29-7

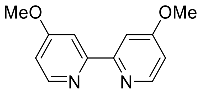

L2
Sigma-Aldrich
CAS: 17217-57-1

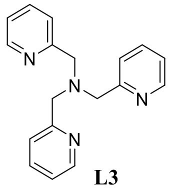

L3
TCI Chemicals
CAS: 16858-01-8

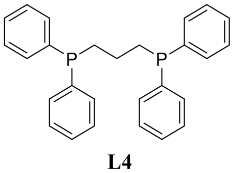

L4
Sigma-Aldrich
CAS: 1663-45-2

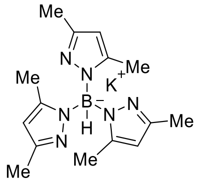

L5
Sigma-Aldrich
CAS: 17567-17-8

Supplementary Figure 3. Ligands used in the development of methylation of tertiary C(sp^3)–H bonds.

2.2 Photosensitisers, Photocatalysts

All photosensitisers and photocatalysts below were purchased from various commercial vendors.

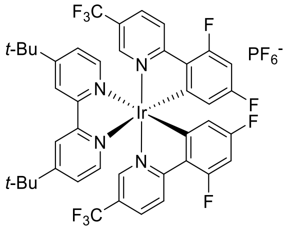

(Ir[dF(CF_3)ppy]$_2$(dtbpy))PF_6 (Ir-F)
Sigma-Aldrich
CAS: 1335047-34-1

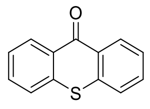

Thioxanthone
Sigma-Aldrich
CAS: 492-22-8

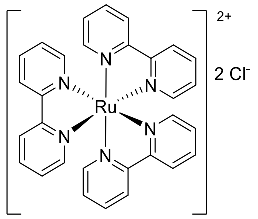

[Ru(bpy)$_3$]Cl_2
Sigma-Aldrich
CAS: 14323-06-9

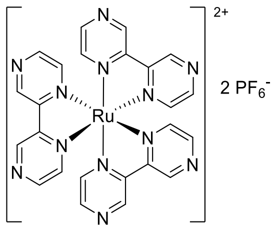

[Ru(bpz)$_3$](PF_6)$_2$
Sigma-Aldrich
CAS: 80907-56-8

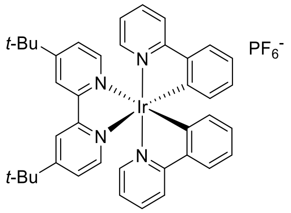

[Ir(dtbbpy)(ppy)$_2$]PF_6
Sigma-Aldrich
CAS: 676525-77-2

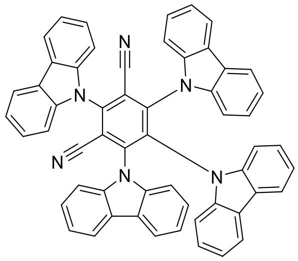

4CzIPn
Sigma-Aldrich
CAS: 1416881-52-1

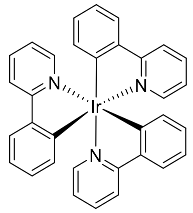

Ir(ppy)$_3$
Sigma-Aldrich
CAS: 94928-86-6

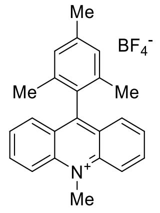

9-Mesityl-10-methylacridinium tetrafluoroborate
Sigma-Aldrich
CAS: 1442433-71-7

Supplementary Figure 4. Photosensitisers used in the development of methylation of tertiary C(sp^3)–H bonds.

2.3 Bidentate Ligands

All bidentate ligands below were purchased from various commercial vendors.

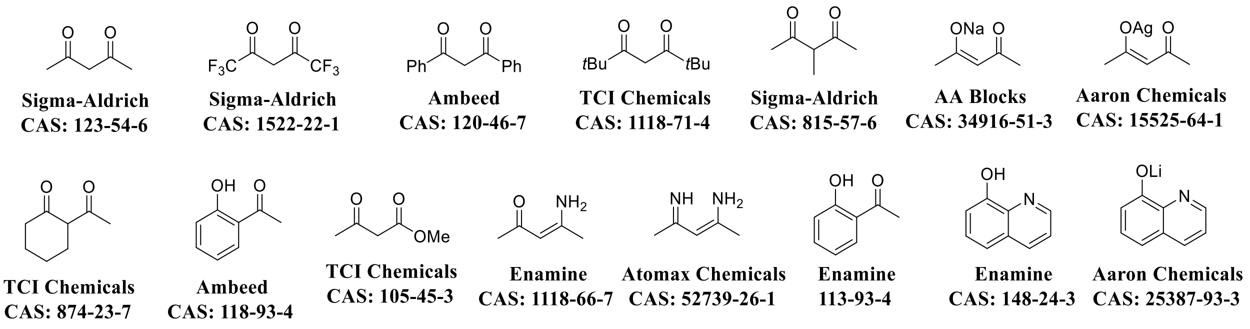

Supplementary Figure 5. Bidendate derivatives used in the development of methylation of tertiary C(sp^3)–H bonds.

2.4 Peroxides, Peracetates, Perbenzoates, Peracids

Peroxides, hydroperoxides, peracetates, perbenzoates, and peracids below were purchased from various commercial vendors or prepared according to literature procedures.[1]

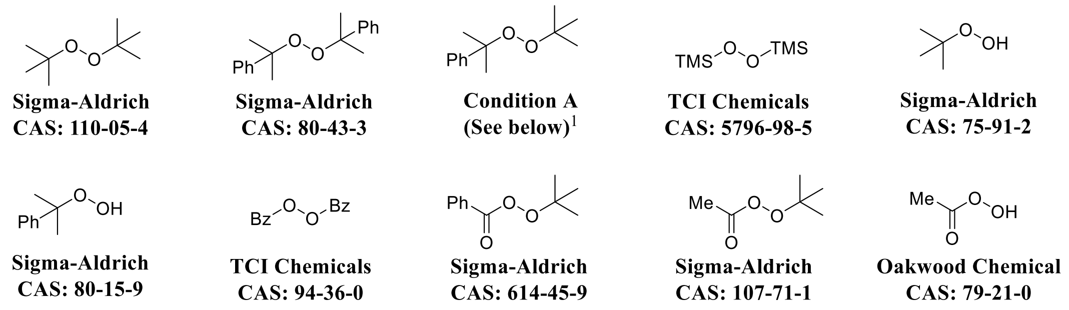

Supplementary Figure 6. Peroxides, hydroperoxides, peracetates, perbenzoates, and peracids used in the development of methylation of tertiary C(sp^3)–H bonds.

Safety handling:

Peroxides, peracetates, perbenzoates, and peracids are known to pose explosion risks. Therefore, appropriate care should be taken when handling these reagents. For example, do not store these chemicals in open, partially empty, or transparent containers. Do not allow open flames, other sources of heat or sparks, friction, grinding, or forms of impact near these reagents.

For more comprehensive safety guidelines, please see:

https://ehs.berkeley.edu/sites/default/files/pecguidelines.pdf

General Procedure A: Trichloroacetimidate (2.28 g, 22.5 mmol) and a solution of cumene hydroperoxide (2.28 g, 4 mL 15.0 mmol) in CH_2Cl_2 (4 mL) were added to a flask charged with a stirring bar, and the flask was cooled to −20 °C (NaCl + ice water) with stirring. Then, $BF_3.Et_2O$ diluted with 6 mL CH_2Cl_2 (74 µL, 0.600 mmol) was added dropwise to the reaction mixture. The reaction mixture was allowed to stir and reach room temperature, then approximately 20 mg $NaHCO_3$ was added to quench the reaction. The solid was removed by filtration. After evaporation of volatile materials and purification with flash column chromatography (SiO_2) eluting with hexane, (2-(tert-butylperoxy)propan-2-yl)benzene (1.12 g, 36%) was obtained as a colorless oil.[1]

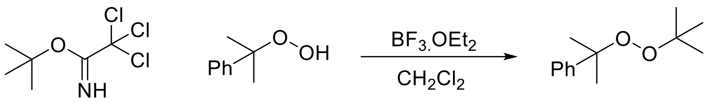

(2-(tert-butylperoxy)propan-2-yl)benzene:

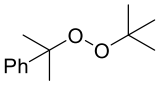

The titled compound was synthesized according to **General Procedure A**.

^{1}H NMR (500 MHz, $CDCl_3$) δ 7.50–7.46 (m, 2H), 7.36–7.30 (m, 2H), 7.26–7.21 (m, 1H), 1.58 (s, 6H), 1.24 (s, 9H).

^{13}C NMR (126 MHz, $CDCl_3$) δ 146.4, 127.99, 126.8, 125.7, 81.4, 78.9, 27.1, 26.8.

The spectra matched those reported in the literature.[1]

2.5 Substrates

Substrates in **Supplementary Table 1** were purchased from commercial vendors (**1a**), prepared according to literature procedures (**1b**–**1s**, **1u**–**4a**, **7a**, **9a**, **10a**),[2–9] or synthesized independently (**1t**, **4b**, **4c**, **8a**).

Number	Structure	Commercial vendors or reported literature
**1a**	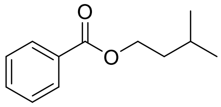	Sigma-Aldrich<br>CAS: 94-46-2
**1b**[2]	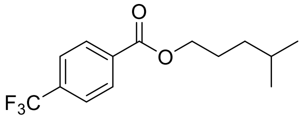	*Angew. Chem. Int. Ed.* **2021**, 60, 8276−8283.
**1c**[2]	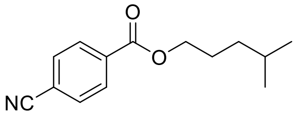	*Angew. Chem. Int. Ed.* **2021**, 60, 8276−8283.
**1d**[2]	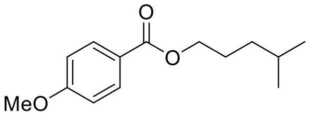	*J. Am. Chem. Soc.* **2018**, 140, 16026−16031.
**1e**[2]	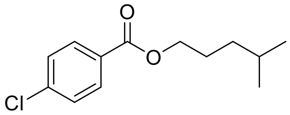	*Angew. Chem. Int. Ed.* **2021**, 60, 8276−8283.
**1f**[2]	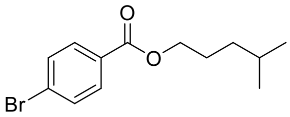	*Angew. Chem. Int. Ed.* **2021**, 60, 8276−8283.
**1g**[2]	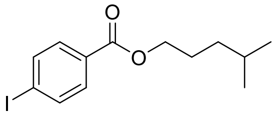	*Angew. Chem. Int. Ed.* **2021**, 60, 8276−8283.
**1h**[2]	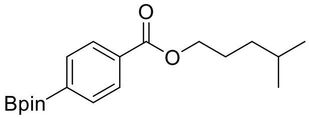	*Angew. Chem. Int. Ed.* **2021**, 60, 8276−8283.

**1i**[2]	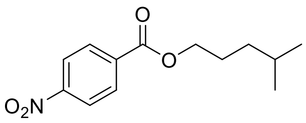 **(Unsuccessful for methylation)**	*Angew. Chem. Int. Ed.* **2021**, 60, 8276−8283.
**1j**[2]	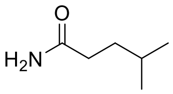 **(Unsuccessful for methylation)**	*Angew. Chem. Int. Ed.* **2021**, 60, 8276−8283.
**1k**[2]	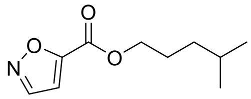	*Angew. Chem. Int. Ed.* **2021**, 60, 8276−8283.
**1l**[2]	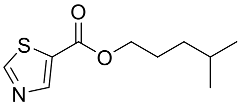	*Angew. Chem. Int. Ed.* **2021**, 60, 8276−8283.
**1m**[2]	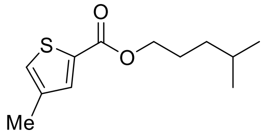	*Angew. Chem. Int. Ed.* **2021**, 60, 8276−8283.
**1n**[2]	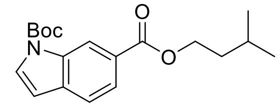	*Angew. Chem. Int. Ed.* **2021**, 60, 8276−8283.
**1o**[2]	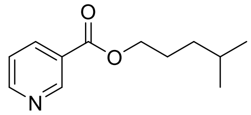	*Angew. Chem. Int. Ed.* **2021**, 60, 8276−8283.
**1p**[2]	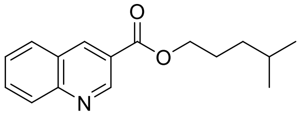	*Angew. Chem. Int. Ed.* **2021**, 60, 8276−8283.
**1q**[2]	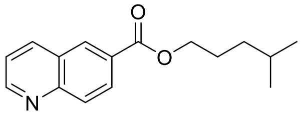	*Angew. Chem. Int. Ed.* **2021**, 60, 8276−8283.

**1r**[2]	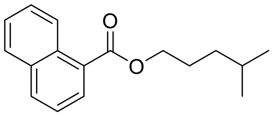	*Angew. Chem. Int. Ed.* **2021**, 60, 8276−8283.
**1s**[3]	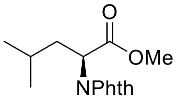	*J. Am. Chem. Soc.* **2016**, 138, 16200−16203.
**1t**[4]	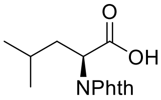	*Org. Lett.* **2018**, 20, 7100−7103.
**1u**	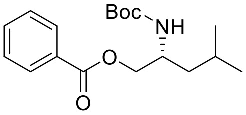	See below
**1v**[5]	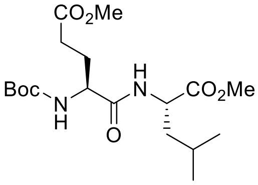	*Chem. Sci.* **2016**, 7, 2679−2683.
**1w**[2]	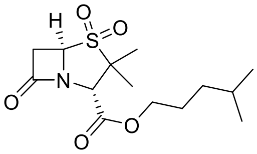	*Angew. Chem. Int. Ed.* **2021**, 60, 8276−8283.
**1x**[2]	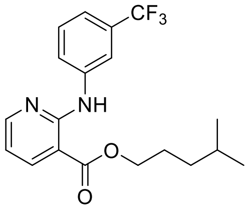	*Angew. Chem. Int. Ed.* **2021**, 60, 8276−8283.
**1y**[2]	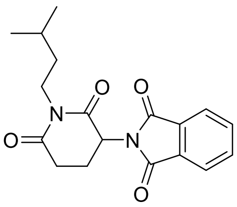	*Angew. Chem. Int. Ed.* **2021**, 60, 8276−8283.
**1z**[2]	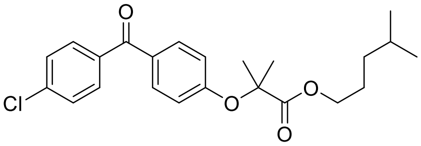	*Angew. Chem. Int. Ed.* **2021**, 60, 8276−8283.

**1aa**[6]	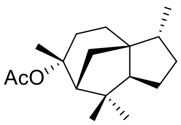	*ACS Catal.* **2020**, 10, 5454−5461.
**1ab**[7]	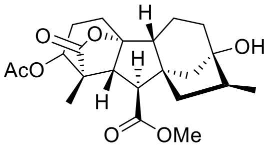	*J. Am. Chem. Soc.* **1960**, 3040−3045.
**4a**[8]	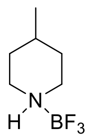	*J. Am. Chem. Soc.* **2015**, 137, 14590–14593.
**4b**	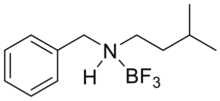	See below
**4c**	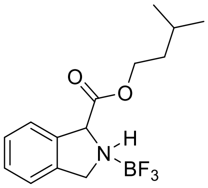	See below
**7a**[9]	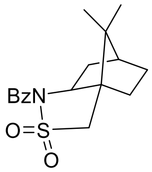 **(Unsuccessful for methylation)**	*Org. Process Res. Dev.* **2009**, 13, 255–262.
**8a**	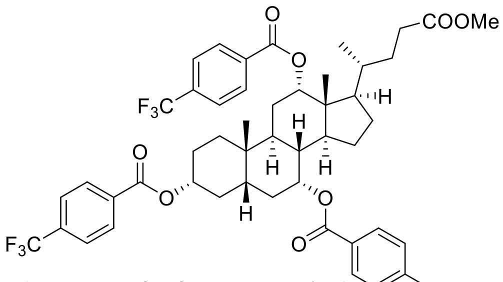 **(Unsuccessful for methylation)**	See below

Supplementary Table 1. Substrates for the methylation of tertiary C(sp^3)–H bonds.

1b (4-Methylpentyl 4-(trifluoromethyl)benzoate):

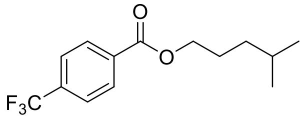

^{1}H NMR (500 MHz, $CDCl_3$) δ 8.15 (d, *J* = 7.8 Hz, 2H), 7.70 (d, *J* = 8.2 Hz, 2H), 4.34 (t, *J* = 6.8 Hz, 2H), 1.84–1.73 (m, 2H), 1.62 (dq, *J* = 13.3, 6.6 Hz, 1H), 1.35–1.29 (m, 2H), 0.92 (d, *J* = 6.6 Hz, 6H).

^{13}C NMR (126 MHz, $CDCl_3$) δ 165.6, δ 134.5 (q, *J* = 32.7 Hz), 133.9, 130.1, 125.5 (q, *J* = 3.8 Hz), 123.8 (q, *J* = 272.6 Hz), 66.1, 35.2, 27.9, 26.7, 22.6.

^{19}F NMR (470 MHz, $CDCl_3$) δ -63.11.

The spectra matched those reported in the literature.[2]

1c (4-Methylpentyl 4-cyanobenzoate):

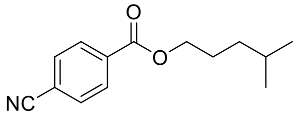

^{1}H NMR (500 MHz, $CDCl_3$) δ 8.13 (d, *J* = 8.2 Hz, 2H), 7.74 (d, *J* = 8.1 Hz, 2H), 4.33 (t, *J* = 6.8 Hz, 2H), 1.87–1.68 (m, 2H), 1.61 (qd, *J* = 11.9, 5.3 Hz, 1H), 1.37–1.25 (m, 2H), 0.91 (d, *J* = 6.6 Hz, 6H).

^{13}C NMR (126 MHz, $CDCl_3$) δ 165.1, 134.4, 132.3, 130.2, 118.1, 116.4, 66.3, 35.1, 27.9, 26.6, 22.6.

The spectra matched those reported in the literature.[2]

1d (4-Methylpentyl 4-methoxybenzoate):

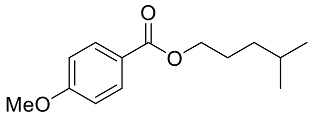

^{1}H NMR (500 MHz, $CDCl_3$) δ 8.11–7.90 (m, 2H), 6.96–6.85 (m, 2H), 4.27 (t, *J* = 6.7 Hz, 2H), 3.85 (d, *J* = 1.3 Hz, 3H), 1.81–1.70 (m, 2H), 1.60 (dp, *J* = 13.4, 6.7 Hz, 1H), 1.36–1.26 (m, 2H), 0.91 (d, *J* = 6.7 Hz, 6H).

^{13}C NMR (126 MHz, $CDCl_3$) δ 166.6, 163.4, 131.6, 123.1, 113.7, 65.2, 55.5, 35.3, 27.9, 26.8, 22.6.

The spectra matched those reported in the literature.[2]

1e (4-Methylpentyl 4-chlorobenzoate):

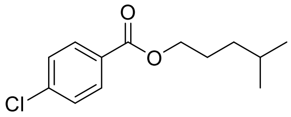

^{1}H NMR (500 MHz, $CDCl_3$) δ 8.01–7.91 (m, 2H), 7.39 (dd, *J* = 8.5, 1.8 Hz, 2H), 4.29 (t, *J* = 6.8 Hz, 2H), 1.75 (dq, *J* = 11.2, 6.9 Hz, 2H), 1.60 (dp, *J* = 13.3, 6.7 Hz, 1H), 1.35–1.24 (m, 2H), 0.95 – 0.86 (m, 6H).

^{13}C NMR (126 MHz, $CDCl_3$) δ 165.9, 165.8, 139.3, 131.0, 129.1, 128.8, 65.7, 35.2, 27.9, 26.7, 22.6.

The spectra matched those reported in the literature.[2]

1f (4-Methylpentyl 4-bromobenzoate):

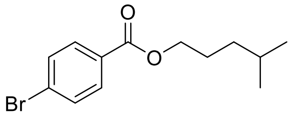

^{1}H NMR (500 MHz, $CDCl_3$) δ 7.97 – 7.83 (m, 1H), 7.66 – 7.52 (m, 1H), 4.29 (t, *J* = 6.8 Hz, 2H), 1.84 – 1.69 (m, 2H), 1.66 – 1.55 (m, 1H), 1.35 – 1.24 (m, 2H), 0.91 (d, *J* = 6.6 Hz, 4H).

^{13}C NMR (126 MHz, $CDCl_3$) δ 166.1, 131.8, 131.2, 129.6, 128.0, 65.8, 35.2, 27.9, 26.7, 22.7.

The spectra matched those reported in the literature.[2]

1g (4-Methylpentyl 4-iodobenzoate):

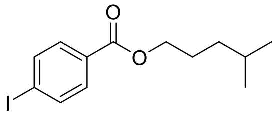

^{1}H NMR (500 MHz, $CDCl_3$) δ 7.78 (dd, *J* = 8.5, 1.4 Hz, 2H), 7.73 (d, *J* = 8.6 Hz, 2H), 4.28 (t, *J* = 6.8 Hz, 2H), 1.80–1.70 (m, 2H), 1.59 (dp, *J* = 13.3, 6.7 Hz, 1H), 1.33–1.25 (m, 2H), 0.90 (d, *J* = 6.7 Hz, 6H).

^{13}C NMR (126 MHz, $CDCl_3$) δ 166.2, 137.7, 131.1, 130.1, 100.6, 65.7, 35.2, 27.8, 26.7, 22.6.

The spectra matched those reported in the literature.[2]

1h (4-methylpentyl 4-(4,4,5,5-tetramethyl-1,3,2-dioxaborolan-2-yl)benzoate):

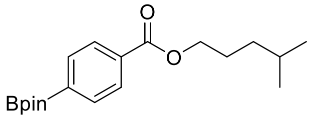

^{1}H NMR (500 MHz, $CDCl_3$) δ 8.02 (d, *J* = 8.3 Hz, 2H), 7.87 (d, *J* = 8.3 Hz, 2H), 4.30 (t, *J* = 6.8 Hz, 2H), 1.82–1.71 (m, 2H), 1.61 (dp, *J* = 13.3, 6.7 Hz, 1H), 1.36 (s, 12H), 1.34–1.28 (m, 2H), 0.92 (d, *J* = 6.6 Hz, 6H).

^{13}C NMR (126 MHz, $CDCl_3$) δ 166.9, 134.8, 132.9, 128.7, 84.3, 65.6, 35.2, 27.9, 26.8, 25.0, 22.7.

The spectra matched those reported in the literature.[2]

1i (4-Methylpentyl 4-nitrobenzoate):

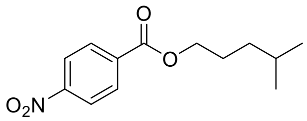

^{1}H NMR (500 MHz, $CDCl_3$) δ 8.33–8.25 (m, 2H), 8.23–8.15 (m, 2H), 4.35 (t, *J* = 6.8 Hz, 2H), 1.84–1.71 (m, 2H), 1.61 (dq, *J* = 13.3, 6.7 Hz, 1H), 1.37–1.25 (m, 2H), 0.92 (d, *J* = 6.7 Hz, 6H).

^{13}C NMR (126 MHz, $CDCl_3$) δ 164.9, 150.6, 136.0, 130.8, 123.6, 66.5, 35.2, 27.9, 26.6, 22.6.

The spectra matched those reported in the literature.[2]

1j (4-methylpentanamide):

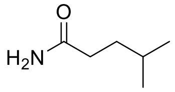

^{1}H NMR (500 MHz, $CDCl_3$) δ 5.73 (brs, 1H), 5.49 (brs, 1H), 2.20 (t, *J* = 7.7 Hz, 2H), 1.63 (tt, *J* = 11.0, 6.5 Hz, 2H), 1.54 (dq, *J* = 13.3, 6.7 Hz, 1H), 1.25–1.17 (m, 2H), 0.88 (d, *J* = 6.7 Hz, 6H).

^{13}C NMR (126 MHz, $CDCl_3$) δ 176.0, 38.6, 36.3, 27.9, 23.5, 22.6.

The spectra matched those reported in the literature.[2]

1k (4-methylpentyl isoxazole-5-carboxylate):

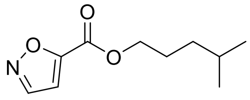

^{1}H NMR (500 MHz, $CDCl_3$) δ 8.36 (d, *J* = 1.8 Hz, 1H), 6.94 (d, *J* = 1.8 Hz, 1H), 4.35 (t, *J* = 6.8 Hz, 2H), 1.87–1.71 (m, 2H), 1.59 (dp, *J* = 13.3, 6.7 Hz, 1H), 1.36–1.23 (m, 2H), 0.90 (d, *J* = 6.7 Hz, 6H).

^{13}C NMR (126 MHz, $CDCl_3$) δ 160.2, 156.9, 150.8, 108.8, 66.8, 34.9, 27.8, 26.5, 22.6.

The spectra matched those reported in the literature.[2]

1l (4-methylpentyl thiazole-5-carboxylate):

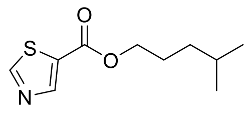

^{1}H NMR (500 MHz, $CDCl_3$) δ 8.93 (s, 1H), 8.49 (s, 1H), 4.30 (t, *J* = 6.8 Hz, 2H), 1.87–1.66 (m, 2H), 1.59 (dp, *J* = 13.3, 6.7 Hz, 1H), 1.33–1.20 (m, 2H), 0.90 (d, *J* = 6.7 Hz, 6H).

^{13}C NMR (126 MHz, $CDCl_3$) δ 161.3, 157.9, 148.8, 130.0, 66.2, 35.0, 27.7, 26.5, 22.5.

The spectra matched those reported in the literature.[2]

1m (4-methylpentyl 4-methylthiophene-2-carboxylate):

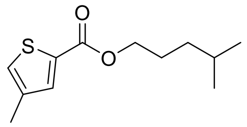

^{1}H NMR (500 MHz, $CDCl_3$) δ 7.59 (d, *J* = 1.5 Hz, 1H), 7.12 (s, 1H), 4.25 (t, *J* = 6.8 Hz, 2H), 2.28 (s, 2H), 1.79–1.69 (m, 2H), 1.60 (dp, *J* = 13.4, 6.6 Hz, 1H), 1.35–1.24 (m, 2H), 0.91 (d, *J* = 6.6 Hz, 6H).

^{13}C NMR (126 MHz, $CDCl_3$) δ 162.6, 138.5, 135.2, 133.8, 128.0, 65.6, 35.2, 27.9, 26.7, 22.6, 15.7.

The spectra matched those reported in the literature.[2]

1n (1-(tert-butyl) 6-(4-methylpentyl) 1H-indole-1,6-dicarboxylate):

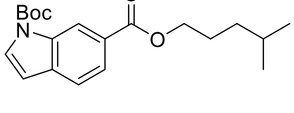

^{1}H NMR (500 MHz, $CDCl_3$) δ 8.84 (s, 1H), 7.98–7.87 (m, 1H), 7.76 (t, J = 3.2 Hz, 1H), 7.59 (dt, J = 8.3, 1.6 Hz, 1H), 6.62 (dd, J = 3.7, 0.8 Hz, 1H), 4.33 (t, J = 6.8 Hz, 2H), 1.87–1.75 (m, 2H), 1.71 (s, 9H), 1.67–1.55 (m, 2H), 1.39 – 1.29 (m, 2H), 0.92 (d, J = 6.6 Hz, 6H).

^{13}C NMR (126 MHz, $CDCl_3$) δ 167.4, 149.6, 134.6, 134.4, 129.0, 126.4, 124.0, 120.7, 117.2, 107.3, 84.5, 65.5, 35.3, 28.3, 27.9, 26.9, 22.7.

The spectra matched those reported in the literature.[2]

1o (4-methylpentyl nicotinate):

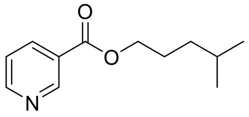

^{1}H NMR (500 MHz, $CDCl_3$) δ 9.22 (s, 1H), 8.76 (dt, J = 4.7, 1.8 Hz, 1H), 8.28 (dq, J = 8.0, 1.8 Hz, 1H), 7.42–7.32 (m, 1H), 4.33 (t, J = 6.8 Hz, 2H), 1.83–1.73 (m, 2H), 1.60 (dtd, J = 13.3, 6.7, 1.4 Hz, 1H), 1.37–1.26 (m, 2H), 0.91 (d, J = 6.6 Hz, 6H).

^{13}C NMR (126 MHz, $CDCl_3$) δ 165.5, 153.4, 151.0, 137.2, 126.5, 123.4, 66.0, 35.2, 27.9, 26.7, 22.6.

The spectra matched those reported in the literature.[2]

1p (4-methylpentyl quinoline-3-carboxylate):

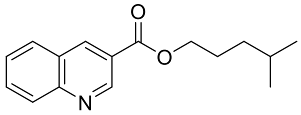

^{1}H NMR (500 MHz, $CDCl_3$) δ 9.43 (d, J = 2.1 Hz, 1H), 8.80 (dd, J = 2.2, 0.9 Hz, 1H), 8.13 (dq, J = 8.4, 0.9 Hz, 1H), 7.91 (dd, J = 8.1, 1.4 Hz, 1H), 7.80 (ddd, J = 8.5, 6.9, 1.5 Hz, 1H), 7.59 (ddd, J = 8.2, 6.9, 1.2 Hz, 1H), 4.38 (t, J = 6.8 Hz, 2H), 1.85–1.77 (m, 2H), 1.61 (dt, J = 13.4, 6.7 Hz, 1H), 1.40–1.29 (m, 2H), 0.91 (d, J = 6.6 Hz, 6H).

^{13}C NMR (126 MHz, $CDCl_3$) δ 165.5, 150.2, 149.9, 138.7, 131.8, 129.6, 129.2, 127.5, 126.9, 123.4, 66.0, 35.2, 27.9, 26.7, 22.6.

The spectra matched those reported in the literature.[2]

1q (4-methylpentyl quinoline-6-carboxylate):

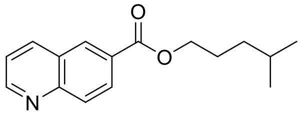

^{1}H NMR (500 MHz, $CDCl_3$) δ 8.98 (dd, *J* = 4.2, 1.8 Hz, 1H), 8.56 (d, *J* = 1.9 Hz, 1H), 8.29 (dd, *J* = 8.8, 1.9 Hz, 1H), 8.25 (ddd, *J* = 8.4, 1.7, 0.8 Hz, 1H), 8.13 (dd, *J* = 8.9, 0.8 Hz, 1H), 7.44 (dd, *J* = 8.3, 4.2 Hz, 1H), 4.36 (t, *J* = 6.8 Hz, 2H), 1.86–1.73 (m, 2H), 1.62 (dp, *J* = 13.3, 6.7 Hz, 1H), 1.42–1.27 (m, 2H), 0.92 (d, *J* = 6.7 Hz, 6H).

^{13}C NMR (126 MHz, $CDCl_3$) δ 166.3, 152.5, 150.2, 137.4, 130.97, 129.9, 129.1, 128.6, 127.5, 121.9, 65.9, 35.2, 27.9, 26.8, 22.6.

The spectra matched those reported in the literature.[2]

1r (4-methylpentyl 1-naphthoate):

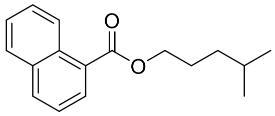

^{1}H NMR (500 MHz, $CDCl_3$) δ 8.97–8.88 (m, 1H), 8.27–8.13 (m, 1H), 8.02 (d, *J* = 8.3 Hz, 1H), 7.89 (dt, *J* = 8.3, 0.9 Hz, 1H), 7.68–7.60 (m, 1H), 7.59–7.46 (m, 2H), 4.42 (t, *J* = 6.8 Hz, 2H), 1.92–1.78 (m, 2H), 1.66 (dp, *J* = 13.4, 6.7 Hz, 1H), 1.44–1.33 (m, 2H), 0.95 (d, *J* = 7.8 Hz, 6H).

^{13}C NMR (126 MHz, $CDCl_3$) δ 167.8, 134.0, 133.3, 131.5, 130.2, 128.6, 127.8, 127.7, 126.3, 126.0, 124.6, 65.6, 35.3, 27.9, 26.8, 22.7.

The spectra matched those reported in the literature.[2]

1s (methyl (S)-2-(1,3-dioxoisoindolin-2-yl)-4-methylpentanoate):

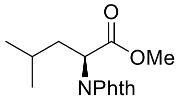

^{1}H NMR (500 MHz, $CDCl_3$) δ 7.86 (dd, *J* = 5.4, 3.0 Hz, 2H), 7.74 (dd, *J* = 5.5, 3.0 Hz, 2H), 4.95 (ddd, *J* = 11.7, 4.6, 2.4 Hz, 1H), 3.72 (s, 3H), 2.33 (ddd, *J* = 14.2, 11.5, 4.0 Hz, 1H), 1.96 (ddd, *J* = 14.5, 10.8, 4.4 Hz, 1H), 1.54–1.42 (m, 1H), 0.93 (dd, *J* = 13.5, 6.6 Hz, 6H).

^{13}C NMR (126 MHz, $CDCl_3$) δ 170.4, 167.9, 134.3, 131.98, 123.7, 52.9, 50.7, 37.4, 25.2, 23.3, 21.2.

The spectra matched those reported in the literature.[3]

1t ((S)-2-(1,3-dioxoisoindolin-2-yl)-4-methylpentanoic acid):

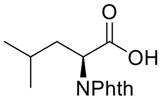

^{1}H NMR (500 MHz, $CDCl_3$) δ 7.86 (dd, J = 5.4, 3.0 Hz, 2H), 7.73 (dd, J = 5.5, 3.0 Hz, 2H), 5.00 (dd, J = 11.5, 4.4 Hz, 1H), 2.37 (ddd, J = 14.2, 11.5, 4.2 Hz, 1H), 1.95 (ddd, J = 14.4, 10.2, 4.4 Hz, 1H), 1.50 (dqd, J = 17.1, 6.5, 3.3 Hz, 1H), 0.94 (dd, J = 10.7, 6.6 Hz, 6H).

^{13}C NMR (126 MHz, $CDCl_3$) δ 176.1, 167.8, 134.4, 131.95, 123.7, 49.5, 41.3, 30.5, 29.3.

The spectra matched those reported in the literature.[4]

1u (2,2,2-trifluoro-1-(4-methylpiperidin-1-yl)ethan-1-one):

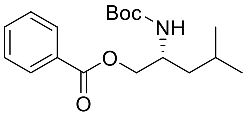

Benzoic acid (214 mg, 1.05 equiv, 1.75 mmol) and 4-dimethylamino pyridine (20.4 mg, 0.10 equiv, 0.167 mmol) were added to a flask. CH_2Cl_2 (0.10 M) and D-Leucinol (362 mg, 1.0 equiv, 1.67 mmol) were then added, followed by N,N'-diisopropylcarbodiimide (DIC) (274 μL, 1.05 equiv, 1.75 mmol). The reaction mixture was allowed to stir at room temperature for 24 hours, before concentration under reduced pressure. The crude residue was directly purified by flash-column chromatography (SiO_2) eluting with hexane and EtOAc (1:4) to yield **1u** as a white solid (518 mg, 97% yield).

^{1}H NMR (500 MHz, $CDCl_3$) δ 8.03 (dd, J = 8.4, 1.4 Hz, 2H), 7.58–7.51 (m, 1H), 7.42 (t, J = 7.8 Hz, 2H), 4.56 (d, J = 9.3 Hz, 1H), 4.27 (qd, J = 11.2, 5.1 Hz, 2H), 4.09 (dt, J = 10.0, 5.2 Hz, 1H), 1.73 (p, J = 6.6 Hz, 1H), 1.40 (s, 9H), 1.37 (m, 1H, overlapped), 0.95 (d, J = 6.7 Hz, 6H)

^{13}C NMR (126 MHz, $CDCl_3$) δ 166.65, 155.56, 133.19, 130.17, 129.83, 128.52, 79.55, 67.48, 48.08, 41.20, 28.48, 24.94, 23.15, 22.37.

HRMS (m/z): (ESI+) calc'd for $C_{18}H_{28}NO_4$ $[M+H]^+$: 322.2018, found: 322.2020

1v (methyl (S)-4-((tert-butoxycarbonyl)amino)-5-(((S)-1-methoxy-4-methyl-1-oxopentan-2-yl)amino)-5-oxopentanoate):

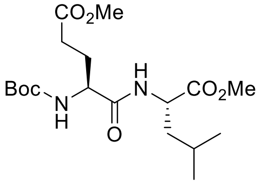

^{1}H NMR (500 MHz, $CDCl_3$) δ 6.63 (s, 1H), 5.23 (s, 1H), 4.59 (td, *J* = 8.9, 4.6 Hz, 1H), 4.24–4.14 (m, 1H), 3.72 (s, 3H), 3.70 (s, 3H), 2.60–2.40 (m, 2H), 2.12 (dtd, *J* = 14.2, 7.1, 5.9 Hz, 1H), 1.93 (dq, *J* = 14.5, 7.2 Hz, 1H), 1.70–1.61 (m, 2H), 1.60–1.51 (m, 1H), 1.43 (s, 9H), 0.97 – 0.89 (m, 6H).

^{13}C NMR (126 MHz, $CDCl_3$) δ 174.1, 173.2, 171.5, 155.8, 80.2, 53.6, 52.4, 52.0, 50.9, 41.4, 30.3, 28.4, 28.1, 24.9, 22.96, 21.9.

The spectra matched those reported in the literature.[5]

1w (4-methylpentyl (2S,5R)-3,3-dimethyl-7-oxo-4-thia-1-azabicyclo[3.2.0]heptane-2-carboxylate 4,4-dioxide):

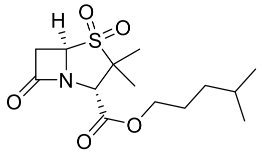

^{1}H NMR (500 MHz, $CDCl_3$) δ 4.61 (dd, *J* = 4.2, 2.1 Hz, 1H), 4.38 (s, 1H), 4.19 (t, *J* = 6.8 Hz, 2H), 3.54–3.39 (m, 2H), 1.73–1.64 (m, 2H), 1.62 (s, 3H), 1.57 (p, *J* = 6.7 Hz, 1H), 1.42 (s, 3H), 1.28–1.20 (m, 2H), 0.90 (d, *J* = 6.6 Hz, 6H).

^{13}C NMR (126 MHz, $CDCl_3$) δ 170.9, 167.2, 67.0, 63.4, 62.8, 61.2, 38.5, 35.0, 27.8, 26.5, 22.6, 22.6, 20.5, 18.8.

The spectra matched those reported in the literature.[2]

1x (4-methylpentyl 1-naphthoate):

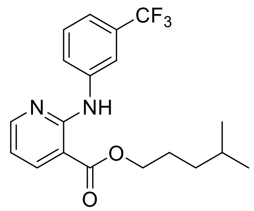

^{1}H NMR (500 MHz, $CDCl_3$) δ 10.41 (s, 1H), 8.41 (dd, *J* = 4.7, 2.0 Hz, 1H), 8.27 (dd, *J* = 7.8, 2.0 Hz, 1H), 8.10 (d, *J* = 2.0 Hz, 1H), 7.89 (dd, *J* = 8.2, 2.2 Hz, 1H), 7.43 (t, *J* = 8.0 Hz, 1H), 7.31–7.27 (m, 1H), 6.80 (dd, *J* = 7.8, 4.7 Hz, 1H), 4.33 (t, *J* = 6.7 Hz, 2H), 1.84–1.75 (m, 2H), 1.63 (dp, *J* = 13.4, 6.7 Hz, 1H), 1.37–1.26 (m, 2H), 0.94 (d, *J* = 6.6 Hz, 6H).

^{13}C NMR (126 MHz, $CDCl_3$) δ 167.7, 155.9, 153.1, 140.5, 140.3, 131.3 (q, *J* = 32.2 Hz), 129.3, 124.3 (q, *J* = 272.5 Hz), 123.6, 119.1 (q, *J* = 3.8 Hz), 117.2 (q, *J* = 4.1 Hz), 114.2, 107.9, 66.0, 35.2, 27.9, 26.7, 22.7.

^{19}F NMR (470 MHz, $CDCl_3$) δ -62.60.

The spectra matched those reported in the literature.[2]

1y (4-methylpentyl 2-((3-(trifluoromethyl)phenyl)amino)nicotinate):

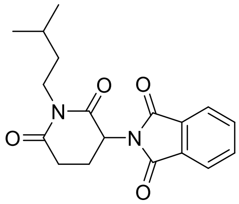

^{1}H NMR (500 MHz, $CDCl_3$) δ 7.89–7.86 (m, 2H), 7.77–7.74 (m, 2H), 5.03–4.93 (m, 1H), 3.90–3.71 (m, 2H), 3.02–2.92 (m, 1H), 2.87–2.69 (m, 2H), 2.18–2.04 (m, 1H), 1.65–1.54 (m, 1H), 1.49–1.35 (m, 2H), 0.92 (d, *J* = 6.7 Hz, 6H).

^{13}C NMR (126 MHz, $CDCl_3$) δ 170.9, 168.5, 167.6, 134.5, 131.9, 123.9, 50.3, 39.5, 36.6, 32.2, 26.4, 22.6, 22.5, 22.2.

The spectra matched those reported in the literature.[2]

1z (4-methylpentyl 2-(4-(4-chlorobenzoyl)phenoxy)-2-methylpropanoate):

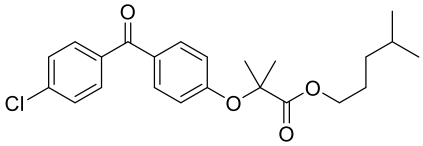

^{1}H NMR (500 MHz, $CDCl_3$) δ 7.71 (dd, *J* = 17.6, 8.7 Hz, 4H), 7.44 (d, *J* = 8.5 Hz, 2H), 6.86 (d, *J* = 8.8 Hz, 2H), 4.14 (t, *J* = 6.7 Hz, 2H), 1.67 (s, 6H), 1.62–1.55 (m, 2H), 1.47 (dp, *J* = 13.3, 6.7 Hz, 1H), 1.13–1.07 (m, 2H), 0.82 (d, *J* = 6.6 Hz, 6H).

^{13}C NMR (126 MHz, $CDCl_3$) δ 194.4, 173.9, 159.9, 138.5, 136.5, 132.2, 131.3, 130.4, 128.7, 117.3, 79.6, 66.3, 34.95, 27.7, 26.4, 25.6, 22.6.

The spectra matched those reported in the literature.[2]

1aa ((3R,3aS,6R,7R,8aS)-3,6,8,8-tetramethyloctahydro-1H-3a,7-methanoazulen-6-yl acetate):

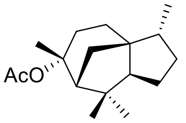

^{1}H NMR (500 MHz, $CDCl_3$) δ 2.39 (dd, *J* = 5.1, 1.5 Hz, 1H), 2.07–0.75 (m, 27H).

^{13}C NMR (126 MHz, $CDCl_3$) δ 170.5, 86.4, 57.0, 56.8, 54.1, 43.5, 41.5, 41.1, 37.1, 33.3, 31.4, 28.6, 27.1, 26.0, 25.4, 22.9, 15.7.

The spectra matched those reported in the literature.[6]

1ab (methyl (1S,4aR,4bR,7S,9aS,10S,10aR)-2-acetoxy-7-hydroxy-1,8-dimethyl-13-oxododecahydro-4a,1-(epoxymethano)-7,9a-methanobenzo[a]azulene-10-carboxylate):

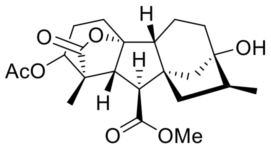

1ab was synthesized based on a reported route.[7]

^{1}H NMR (500 MHz, $CDCl_3$) δ 4.96 (brs, 1H), 3.74 (s, 3H), 3.13 (d, *J* = 10.2 Hz, 1H), 2.62 (d, *J* = 10.3 Hz, 1H), 2.12 (s, 3H), 2.08–1.60 (m, 14H), 1.29 (d, *J* = 35.1 Hz, 1H), 1.04 (s, 3H), 0.99 (d, *J* = 7.1 Hz, 3H).

^{13}C NMR (126 MHz, $CDCl_3$) δ 177.0, 173.3, 170.4, 93.3, 78.7, 71.6, 55.6, 53.4, 52.50, 52.49, 52.2, 51.2, 46.6, 43.8, 41.9, 30.0, 27.6, 25.6, 21.3, 16.9, 15.2, 14.6.

HRMS (ESI) m/z for $C_{22}H_{34}NO_7$ $[M+NH_4]^+$ calcd.: 424.2335, found: 424.2332.

4a ((Trifluoro(4-methylpiperidine-1-ium-yl)borate):

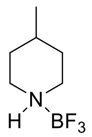

^{1}H NMR (500 MHz, $CDCl_3$) δ 3.67 (s, 1H), 3.45–3.33 (m, 2H), 2.77–2.64 (m, 2H), 1.87 (d, *J* = 14.4 Hz, 2H), 1.61 (ddtdd, *J* = 11.8, 6.5, 5.0, 3.8, 2.0 Hz, 1H), 1.32–1.19 (m, 2H), 0.98 (dt, *J* = 6.6, 1.8 Hz, 3H).

^{13}C NMR (126 MHz, $CDCl_3$) δ 45.96, 32.9, 29.7, 21.7.

^{19}F NMR (470 MHz, $CDCl_3$) δ -157.5 (dd, *J* = 32.9, 16.5 Hz).

The spectra matched those reported in the literature.[8]

General Procedure B was adapted from White and co-workers.[8]

General Procedure B: To a flame-dried round bottom flask equipped with a magnetic stir bar was added secondary amine (1.0 equiv) and CH_2Cl_2 (0.10 M). The solution was cooled to 0 ºC, and BF_3•OEt_2 (0.90 equiv) was added. The solution was stirred at 0 ºC for 30 mins, followed by 1 hour at room temperature. The solvent was removed via evaporation. The crude material was purified by flash column chromatography (SiO_2) eluting with EtOAc and hexane (1:19) to afford the BF_3 complexed amine.

4b (Trifluoro(N-benzyl-N-isopentylamino)borate):

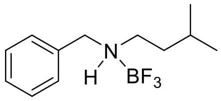

The titled compound was synthesized according to **General procedure B** and afforded as a colorless oil.

^{1}H NMR (500 MHz, $CDCl_3$) δ 7.46 (dd, *J* = 7.9, 1.7 Hz, 2H), 7.41–7.34 (m, 3), 6.34 (s, 1H), 4.17 (s, 2H), 2.92–2.80 (m, 2H), 1.59 (ddd, *J* = 8.4, 5.0, 2.6 Hz, 3H), 0.84 (d, *J* = 6.2 Hz, 6H).

^{13}C NMR (126 MHz, $CDCl_3$) δ 131.13, 130.02, 129.56, 129.39, 51.46, 45.21, 34.64, 25.98, 22.26.

^{19}F NMR (470 MHz, $CDCl_3$) δ -153.64 (dd, *J* = 32.8, 16.0 Hz).

HRMS (m/z): (ESI+) calc'd for $[C_{12}H_{20}N]^+$: 178.1590, found: 178.1590 (the BF_3 adduct is unstable and spontaneously decomplexes on HRMS)

4c (Isopentyl 2-(trifluoro-l4-boraneyl)isoindoline-1-carboxylate):

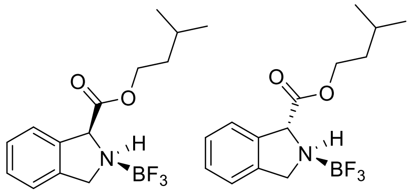

The titled compound was synthesized according to **General procedure B**.

Isolated as a mixture of diastereomers in the form of a colorless oil.

Major diasteromer: ^{1}H NMR (500 MHz, $CDCl_3$) δ 7.53–7.28 (m, 4H, overlapped), 6.03 (s, 1H), 5.37 (d, *J* = 5.9 Hz, 1H), 4.65 (d, *J* = 7.3 Hz, 2H), 4.42–4.33 (m, 2H), 1.64 (m, 2H), 0.96 (t, *J* = 6.8 Hz, 6H).

Minor diasteromer: ^{1}H NMR (500 MHz, $CDCl_3$) δ 7.53–7.29 (m, 4H, overlapped), 5.74 (s, 1H), 4.65 (d, *J* = 7.3 Hz, 2H), 4.30 (td, *J* = 6.9, 2.9 Hz, 2H), 1.77–1.69 (m, 1H, overlapped), 1.62–1.56 (m, 2H), 0.91 (dd, *J* = 9.3, 6.5 Hz, 6H).

^{13}C NMR (126 MHz, $CDCl_3$) δ 170.90, 170.53, 154.32, 153.90, 137.96, 137.43, 135.65, 135.39, 128.80, 128.72, 127.75, 127.73, 123.18, 123.13, 122.94, 122.92, 80.59, 80.47, 65.61, 65.27, 64.20, 64.19, 52.40, 52.14, 37.45, 37.41, 28.58, 28.47, 25.14, 25.11, 22.52, 22.50.

^{19}F NMR (470 MHz, $CDCl_3$) δ -157.60 (dd, *J* = 32.6, 16.0 Hz).

HRMS (m/z): (ESI+) calc'd for $[C_{14}H_{20}NO_2]^+$: 234.1489, found: 234.1490 (the BF_3 adduct is unstable and spontaneously decomplexes on HRMS)

7a (((3aS,6S)-8,8-dimethyl-2,2-dioxidotetrahydro-3H-3a,6-methanobenzo[c]isothiazol-1(4H)-yl)(phenyl)methanone):

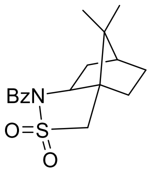

^{1}H NMR (500 MHz, $CDCl_3$) δ 7.79–7.74 (m, 2H), 7.58–7.53 (m, 1H), 7.47–7.41 (m, 2H), 4.19 (dd, *J* = 7.6, 4.9 Hz, 1H), 3.53 (d, *J* = 13.7 Hz, 1H), 3.42 (d, *J* = 13.7 Hz, 1H), 2.17–2.04 (m, 2H), 2.03–1.87 (m, 3H), 1.48 (dd, *J* = 12.1, 9.9 Hz, 1H), 1.44–1.37 (m, 1H), 1.35 (s, 3H), 1.03 (s, 3H).

^{13}C NMR (126 MHz, $CDCl_3$) δ 170.2, 133.9, 132.8, 129.6, 128.2, 66.2, 53.71, 48.2, 48.0, 45.3, 38.5, 33.3, 26.7, 21.4, 20.1.

The spectra matched those reported in the literature.[9]

8a ((3R,5S,7R,8R,9S,10S,12S,13R,14S,17R)-17-((R)-5-methoxy-5-oxopentan-2-yl)-10,13-dimethylhexadecahydro-1H-cyclopenta[a]phenanthrene-3,7,12-triyl tris(4-(trifluoromethyl)benzoate)):

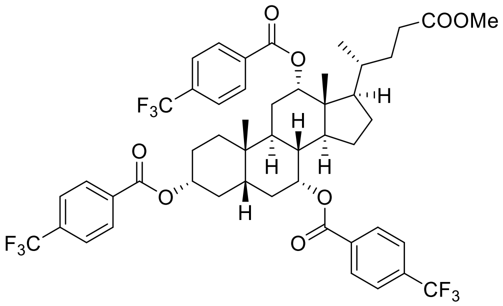

The titled compound was synthesized according to **General Procedure C** adapted from literature.[2]

General Procedure C: 4-Trifluoromethyl benzoic acid (4 equiv) and 4-dimethylamino pyridine (0.30 equiv) were added to a flask. CH_2Cl_2 (0.10 M) and methyl cholate (1.0 equiv) were then added, followed by N,N'-diisopropylcarbodiimide (DIC) (4 equiv). The reaction mixture was allowed to stir at room temperature for 24 hours, before concentration under reduced pressure. The crude residue was directly purified by flash-column chromatography (SiO_2) eluting with EtOAc and hexane (1:9) to yield benzoylated methyl cholate **8a** as a white solid.

^{1}H NMR (500 MHz, $CDCl_3$) δ 8.02 (d, *J* = 8.5 Hz, 2H), 7.96 (d, *J* = 8.6 Hz, 2H), 7.68 (d, *J* = 8.5 Hz, 2H), 7.44 (d, *J* = 8.5 Hz, 2H), 7.39 (d, *J* = 8.5 Hz, 2H), 7.32 (d, *J* = 8.5 Hz, 2H), 5.43–5.38 (m, 1H), 5.28 (d, *J* = 3.1 Hz, 1H), 4.74 (ddt, *J* = 15.8, 10.6, 4.5 Hz, 1H), 3.60 (s, 3H), 2.38 (td, *J* = 12.4, 4.4 Hz, 1H), 2.28–2.06 (m, 5H), 2.00 (dt, *J* = 14.9, 3.9 Hz, 1H), 1.87 (tq, *J* = 12.1, 3.5 Hz, 3H), 1.80–1.58 (m, 8H), 1.45–1.30 (m, 2H), 1.28–1.10 (m, 4H), 1.03 (s, 3H), 0.85 (s, 3H), 0.83 (d, *J* = 6.6 Hz, 3H).

^{13}C NMR (126 MHz, $CDCl_3$) δ 174.5, 165.0, 164.8, 164.5, 139.8, 139.7, 139.5, 131.0, 130.9, 130.8, 129.4, 129.4, 129.2, 129.0, 128.8, 76.5, 74.3, 72.1, 51.6, 48.1, 45.7, 43.9, 40.6, 38.5, 35.1, 34.8, 34.6, 34.5, 31.5, 31.0, 30.8, 29.1, 27.3, 26.7, 25.5, 23.2, 22.6, 17.7, 12.4.

^{19}F NMR (470 MHz, $CDCl_3$) δ -63.42, -63.46, -63.57.

HRMS (m/z): (ESI+) calc'd for $[C_{49}H_{52}F_9O_8]^+$: 939.3519, found: 939.3519

9a (4-methylpentyl pyrazine-2-carboxylate):

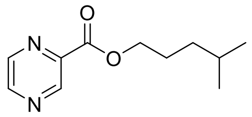

^1H NMR (500 MHz, $CDCl_3$) δ 9.30 (s, 1H), 8.74 (d, J = 11.4 Hz, 2H), 4.44–4.41 (m, 2H), 1.87–1.77 (m, 2H), 1.60 (dp, J = 14.0, 6.9 Hz, 1H), 1.31 (q, J = 7.2 Hz, 2H), 0.90 (d, J = 6.7 Hz, 6H).

^{13}C NMR (126 MHz, $CDCl_3$) δ 164.1, 147.7, 146.4, 144.6, 143.8, 66.9, 35.0, 27.9, 26.7, 22.6.

The spectra matched those reported in the literature.[2]

2,2,2-trifluoro-1-(4-methylpiperidin-1-yl)ethan-1-one:

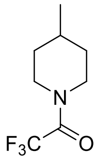

^1H NMR (500 MHz, $CDCl_3$) δ 4.51 (ddt, J = 13.4, 4.7, 2.2 Hz, 1H), 4.06–3.94 (m, 1H), 3.22–3.05 (m, 1H), 2.83–2.66 (m, 1H), 1.83–1.74 (m, 2H), 1.70 (dtd, J = 11.0, 6.6, 3.6 Hz, 1H), 1.38–1.13 (m, 2H), 0.99 (dd, J = 6.6, 1.4 Hz, 3H).

^{13}C NMR (126 MHz, $CDCl_3$) δ 155.5 (q, J = 35.4 Hz), 116.8 (q, J = 288.0 Hz), 46.2 (q, J = 3.5 Hz), 44.1, 34.6, 33.6, 30.98, 21.6.

^{19}F NMR (470 MHz, $CDCl_3$) δ -68.90.

The spectra matched those reported in the literature.[15]

3. Reaction Development of C(sp^3)−H Methylation

3.1 Evaluation of Ligands and Nickel Metal Catalysts

General Procedure D: Ir-F (1.2 mg, 1 mol%, 1.0 μmol), nickel catalyst (5 mol% based on [M], 5.0 μmol), ligand (5 mol%, 5.0 μmol), and dicumyl peroxide (0.600 mmol, 6 equiv, 162 mg) were weighed into a vial charged with a magnetic stirrer. 0.3 mL degassed MeCN was added, followed by isoamyl benzoate substrate **1a** (0.100 mmol, 20 μL). The vial was sealed, and the headspace of the vial was flushed with a stream of N_2 for 1 min. The mixture was then stirred and irradiated with a blue LED for 16 hours with cooling fans maintaining the temperature at 25 °C. The reaction mixture was then concentrated *in vacuo* and filtered through a short silica plug, followed by rinsing the plug with CH_2Cl_2. Volatile materials were evaporated under reduced pressure, and 1,3,5-trimethoxybenzene was added as an internal standard. Crude ^{1}H NMR spectroscopy was performed in $CDCl_3$.

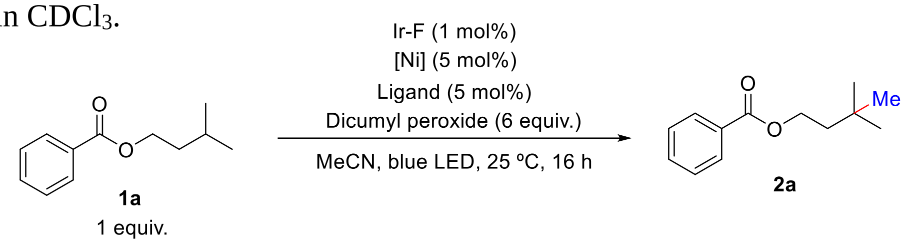

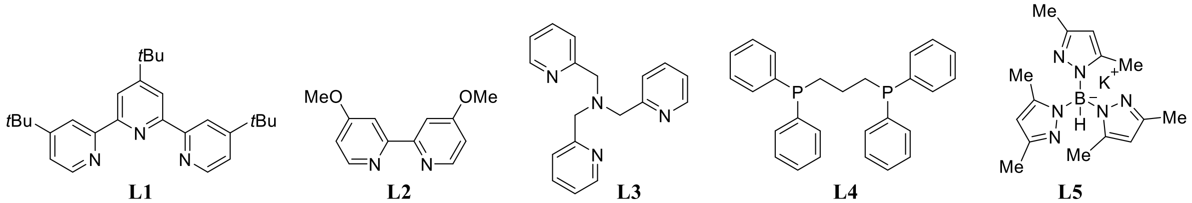

Entry	[Ni]	Ligand	Yield (%)
**1**	$NiCl_2$.diglyme	**L1**	<1
**2**	$NiCl_2$.diglyme	**L2**	<1
**3**	$NiCl_2$.diglyme	**L3**	<1
**4**	$NiCl_2$.diglyme	**L4**	<1
**5**	$NiCl_2$.diglyme	**L5**	<1
**6**	$NiBr_2$	**L1**	<1
**7**	$Ni(acac)_2$	**L1**	18
**8**	$Ni(acac)_2$	**L2**	17
**9**	$Ni(acac)_2$	**L3**	10
**10**	$Ni(acac)_2$	**L4**	13
**11**	$Ni(acac)_2$	**L5**	9
**12**	**$Ni(acac)_2$**	**-**	**20**
**13**	$Ni(dpm)_2$	-	19
**14**	$Ni(hfac)_2$	-	4

Supplementary Table 2. General Procedure D: 0.1 mmol **1a**, 1.0 μmol Ir-F, 5.0 μmol catalyst, 5.0 μmol ligand, 0.6 mmol dicumyl peroxide, in MeCN, irradiation under a blue LED Kessil lamp for at 25 °C for 16 hours. Yields were determined by ^{1}H NMR.

3.2 Evaluation of Metal Catalysts

General Procedure E: Ir-F (1.2 mg, 1 mol %, 1.0 µmol), metal catalyst (5 mol% based on [M], 5.0 µmol), and dicumyl peroxide (0.600 mmol, 6 equiv, 162 mg) were weighed into a vial charged with a magnetic stirrer. 0.3 mL of degassed MeCN was added, followed by isoamyl benzoate **1a** (0.100 mmol, 20 µL). The vial was sealed, and the headspace of the vial was flushed with a stream of N_2 for 1 min. The mixture was then stirred and irradiated with a blue LED for 16 hours with cooling fans maintaining the temperature at 25 °C. The reaction mixture was then concentrated *in vacuo* and filtered through a short silica plug, rinsing with CH_2Cl_2. Volatile materials were evaporated under reduced pressure, and 1,3,5-trimethoxybenzene was added as an internal standard. Crude ^1H NMR spectroscopy was performed in $CDCl_3$.

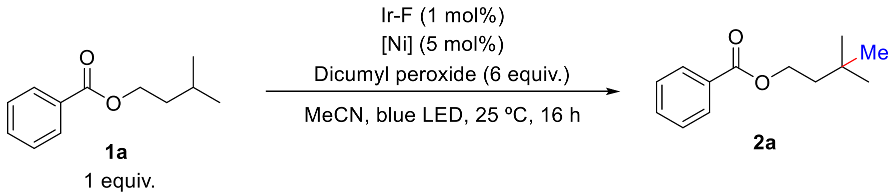

Entry	[M]	Yield (%)
**1**	**$Ni(acac)_2$**	**20**
**2**	$Cu(acac)_2$	0
**3**	$Co(acac)_2$	0
**4**	$Fe(acac)_2$	0
**5**	$Fe(acac)_3$	0
**6**	$Mn(acac)_2$	0
**7**	Ni(TPP)	0
**8**	Ni(OEP)	0
**9**	Fe(TPP)Cl	0
**10**	Fe(OEP)Cl	0
**11**	Fe(II) phthalocyanine	0
**12**	Cu(TPP)	0
**13**	Cu(II) phthalocyanine	0
**14**	Mn(TPP)Cl	0
**15**	Mn(III) phthalocyanine chloride	0
**16**	Mn(II) phthalocyanine	0
**17**	Co(TPP)	0
**18**	Co(II) phthalocyanine	0

Supplementary Table 3. General Procedure E: 0.1 mmol **1a**, 1.0 µmol Ir-F, 5.0 µmol catalyst, 5.0 µmol ligand, 0.6 mmol dicumyl peroxide, in MeCN**,** irradiation under a blue LED Kessil lamp at 25 °C for 16 hours. Yields were

determined by ^{1}H NMR with an internal standard. TPP: 5,10,15,20-Tetraphenyl-21*H*,23*H*-porphine. OEP: 2,3,7,8,12,13,17,18-Octaethyl-21*H*,23*H*-porphine.

3.3 Evaluation of Photosensitisers and Wavelengths of Visible Light

General Procedure F: Photosensitiser (1 mol%, 1.0 µmol), $Ni(acac)_2$ (1.3 mg, 5 mol%, 5.0 µmol), and dicumyl peroxide (0.600 mmol, 6 equiv, 162 mg) were weighed into a vial charged with a magnetic stirrer. 0.3 mL degassed MeCN was added, followed by isoamyl benzoate substrate **1a** (0.100 mmol, 20 µL). The vial was sealed, and the headspace of the vial was flushed with a stream of N_2 for 1 min. The mixture was then stirred and irradiated with a blue LED for 16 hours with cooling fans maintaining the temperature at 25 °C. The reaction mixture was then concentrated *in vacuo* and filtered through a short silica plug, followed by rinsing the plug with CH_2Cl_2. Volatile materials were evaporated under reduced pressure, and 1,3,5-trimethoxybenzene was added as an internal standard. Crude ^{1}H NMR spectroscopy was performed in $CDCl_3$.

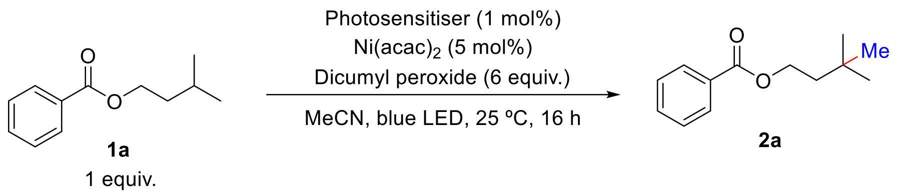

Entry	Photosensitiser	Yield (%)
**1**	**Ir-F**	**20**
**2**	4CzIPn	0
**3**	$Ru(bpy)_3Cl_2$	0
**4**	$Ru(bpz)_3(PF_6)_2$	0
**5**	$[Ir(dtbbpy)(ppy)_2]PF_6$	0
**6**	9-Mesityl-10-methylacridinium tetrafluoroborate	0
**7**	$Ir(ppy)_3$	0
**8**	Thioxanthone (10 mol%)	15
**9**[a]	Thioxanthone (10 mol%)	17
**10**[a]	Ir-F	13

Supplementary Table 4. General Procedure F: 0.1 mmol **1a**, 1.0 µmol photosensitiser, 5.0 µmol catalyst, 5.0 µmol ligand, 0.6 mmol dicumyl peroxide, in MeCN, irradiation under a blue LED Kessil lamp at 25 °C for 16 hours. Yields were determined by ^{1}H NMR with an internal standard. **a.** Irradiation with 370nm purple Kessil lamp.

3.4 Evaluation of Solvents and Peroxides

General Procedure G: Ir-F (1.2 mg, 1 mol%, 1.0 µmol), Ni(acac)$_2$ (1.3 mg, 5 mol%, 5.0 µmol), and oxidant (0.600 mmol, 6 equiv) were weighed into a vial charged with a magnetic stirrer. 0.3 mL degassed solvent was added, followed by isoamyl benzoate substrate **1a** (0.100 mmol, 20 µL). The vial was sealed, and the headspace of the vial was flushed with a stream of N_2 for 1 min. The mixture was then stirred and irradiated with a blue LED for 16 hours with cooling fans maintaining the temperature at 25 °C. The reaction mixture was then concentrated *in vacuo* and filtered through a short silica plug, followed by rinsing the plug with CH_2Cl_2. Volatile materials were evaporated under reduced pressure, and 1,3,5-trimethoxybenzene was added as an internal standard. Crude ^{1}H NMR spectroscopy was performed in $CDCl_3$.

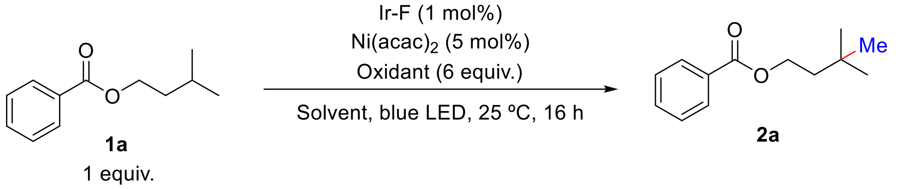

Entry	Oxidant	Solvent	Yield (%)
**1**	**Dicumyl peroxide**	**MeCN**	**20**
**2**	Dicumyl peroxide	Acetone	19
**3**	Dicumyl peroxide	TFE	8
**4**	Dicumyl peroxide	HFIP	<5
**5**	Dicumyl peroxide	DMSO	5
**6**	Dicumyl peroxide	DCE	7
**7**	**Dicumyl peroxide**	**EtOAc**	**20**
**8**	Dicumyl peroxide	MeCN/H_2O (1:1)	<1
**9**	*t*BuOO*t*Bu	MeCN	10
**10**	*t*BuOO*t*Bu	Acetone	9
**11**	*t*BuOO*t*Bu	TFE	19
**12**	*t*BuOO*t*Bu	HFIP	<5
**13**	*t*BuOO*t*Bu	EtOAc	7
**14**	*t*BuOO*t*Bu	DCE	<5
**15**	$K_2S_2O_8$	MeCN	0
**16**	$K_2S_2O_8$	DMSO	0
**17**	$K_2S_2O_8$	MeCN/H_2O (1:1)	0

**18**	$(NH_4)_2S_2O_8$	MeCN	0

Supplementary Table 5. General Procedure G: 0.1 mmol **1a**, 1.0 µmol Ir-F, 5.0 µmol $Ni(acac)_2$, 0.6 mmol oxidant, in stated solvents, irradiation under a blue LED Kessil lamp at 25 °C for 16 hours. Yields were determined by ^1H NMR with an internal standard.

3.5 Evaluation of Oxidants

General Procedure H: Ir-F (1.2 mg, 1 mol%, 1.0 µmol), $Ni(acac)_2$ (1.3 mg, 5 mol%, 5.0 µmol), and oxidant (0.600 mmol, 6 equiv) were weighed into a vial charged with a magnetic stirrer. 0.3 mL of degassed MeCN was added, followed by isoamyl benzoate **1a** (0.100 mmol, 20 µL). The vial was sealed, and the headspace of the vial was flushed with a stream of N_2 for 1 min. The mixture was then stirred and irradiated with a blue LED for 16 hours with cooling fans maintaining the temperature at 25 °C. The reaction mixture was then concentrated *in vacuo* and filtered through a short silica plug, followed by rinsing the plug with CH_2Cl_2. Volatile materials were evaporated under reduced pressure, and 1,3,5-trimethoxybenzene was added as an internal standard. Crude ^1H NMR spectroscopy was performed in $CDCl_3$.

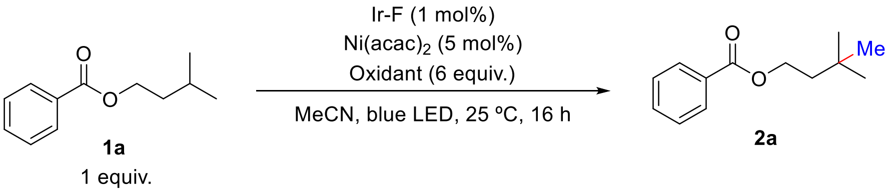

Entry	Oxidant	Yield (%)
**1**	**Dicumyl peroxide**	**20**
**2**	$PhCO_2OtBu$	<5
**3**[a]	$PhCO_2OtBu$	0
**4**	$MeCO_2OtBu$	<5
**5**[a]	$MeCO_2OtBu$	<5
**6**	6 eq. TMS_2O_2, 6 eq. dicumyl peroxide	9
**7**[a]	6 eq. TMS_2O_2, 6 eq. dicumyl peroxide	0
**8**	6 eq. $(BzO)_2$, 6 eq. dicumyl peroxide	<5
**9**	6 eq. $(BzO)_2$, 6 eq. dicumyl peroxide	0
**10**	$MeCO_3H$	0
**11**	*t*BuOOH	0
**12**	Cumene hydroperoxide	0

Supplementary Table 6. General Procedure H: 0.1 mmol **1a**, 1.0 µmol Ir-F, 5.0 µmol $Ni(acac)_2$, 0.6 mmol oxidant, in MeCN, irradiation under a blue LED Kessil lamp at 25 °C for 16 hours. Yields were determined by ^1H NMR with an internal standard. **a.** TFE was used as solvent.

3.6 Evaluation of Concentrations and Temperatures

Ir-F (1.2 mg, 1.0 mol%, 1.0 µmol), $Ni(acac)_2$ (1.3 mg, 5 mol%, 5.0 µmol), and dicumyl peroxide (0.60 mmol, 6 equiv, 162 mg) were weighed into a vial charged with a magnetic stirrer. MeCN was added, followed by isoamyl benzoate substrate **1a** (0.100 mmol, 20 µL). The vial was sealed, and the headspace of the vial was flushed with a stream of N_2 for 1 min. The mixture was then stirred and irradiated with a blue LED for 16 hours with cooling fans and a heating block maintaining the temperature at the stated temperatures. The reaction mixture was then concentrated *in vacuo* and filtered through a short silica plug, followed by rinsing the plug with CH_2Cl_2. Volatile materials were evaporated under reduced pressure, and 1,3,5-trimethoxybenzene was added as an internal standard. Crude ^1H NMR spectroscopy was performed in $CDCl_3$.

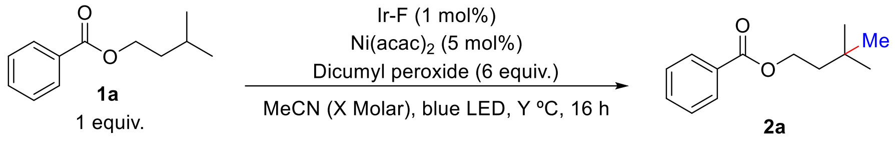

Entry	X	Y	Yield (%)
**1**	0.33	25	17
**2**	0.67	25	19
**3**	1.0	25	20
**4**	2.0	25	20
**5**	1.0	40	24
**6**	1.0	60	12
**7**	1.0	80	7

Supplementary Table 7. Procedure: 0.1 mmol **1a**, 1.0 µmol Ir-F, 5.0 µmol $Ni(acac)_2$, 0.6 mmol dicumyl peroxide, in MeCN, irradiation under a blue LED Kessil lamp for 16 hours. NMR yields.

3.7 Evaluation of the Effects of Various Acidic Additives

General Procedure I: Ir-F (1.2 mg, 0.5 mol%, 1.0 μmol), $Ni(acac)_2$ (2.6 mg, 5 mol%, 10.0 μmol), and dicumyl peroxide (1.20 mmol, 6 equiv, 324 mg) were weighed into a vial charged with a magnetic stirrer. If the additive is a solid, it was also weighed into the vial at this step (1 equiv). 0.2 mL degassed MeCN was then added, followed by isoamyl benzoate substrate **1a** (0.200 mmol, 39.0 μL). If the additive is a liquid, it was added to the mixture via a micropipette at this step. The vial was sealed, and the headspace of the vial was flushed with a stream of N_2 for 1 min. The mixture was then stirred and irradiated with a blue LED for 16 hours with cooling fans maintaining the temperature at 25 °C. The reaction mixture was then concentrated *in vacuo* and filtered through a short silica plug,, followed by rinsing the plug with CH_2Cl_2. Volatile materials were evaporated under reduced pressure, and 1,3,5-trimethoxybenzene was added as an internal standard. Crude ^1H NMR spectroscopy was performed in $CDCl_3$.

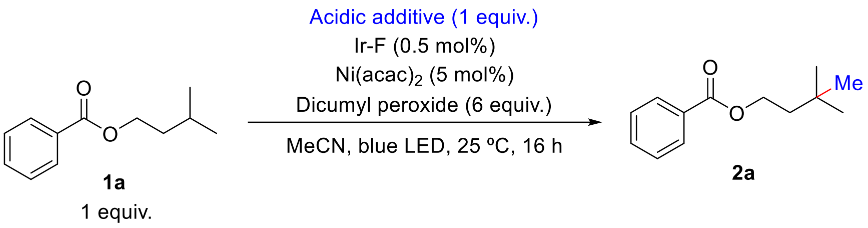

Entry	Acidic additive	Yield (%)
**1**	**None**	**20**
**2**	TFA	4
**3**	$MeB(OH)_2$	20
**4**	$B(OH)_3$	6
**5**	$ZnBr_2$	<5

Supplementary Table 8. General Procedure I: 0.2 mmol **1a**, 1.0 μmol Ir-F, 10.0 μmol $Ni(acac)_2$, 1.2 mmol dicumyl peroxide, 0.2 mmol acidic additive, in MeCN, irradiation under a blue LED Kessil lamp at 25 °C for 16 hours. Yields were determined by ^1H NMR with an internal standard.

Discussion

The acidic additives in reported conditions[10] were found to be either detrimental or have no positive effect on the yield of the reaction.

3.8 Evaluation of Deuteration of the Tertiary C−H bond in MeCN-d_3

Ir-F (1.2 mg, 0.5 mol%, 1.0 µmol), Ni(acac)$_2$ (2.6 mg, 5 mol%, 10.0 µmol), and dicumyl peroxide (1.20 mmol, 6 equiv, 324 mg) were weighed into a vial charged with a magnetic stirrer. 0.2 mL degassed MeCN-d_3 was then added, followed by isoamyl benzoate substrate **1a** (0.200 mmol, 39.0 µL). The vial was sealed, and the headspace of the vial was flushed with a stream of N_2 for 1 min. The mixture was then stirred and irradiated with a blue LED for 16 hours with cooling fans maintaining the temperature at 25 °C. The reaction mixture was then concentrated *in vacuo* and filtered through a short silica plug, followed by rinsing the plug with CH_2Cl_2. Volatile materials were evaporated under reduced pressure, and a known amount of 1,3,5-trimethoxybenzene was added as an internal standard. Crude ^{1}H NMR spectroscopy was performed in $CDCl_3$.

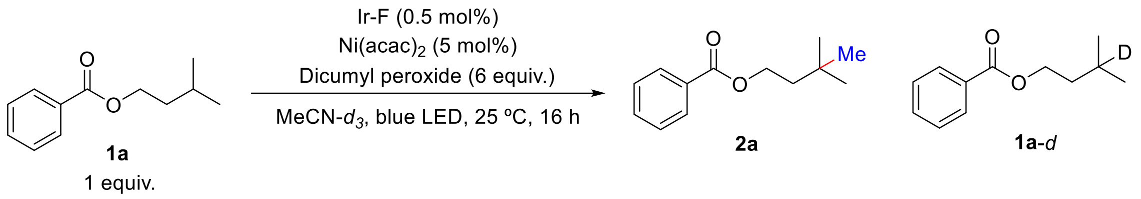

Entry	Solvent	Conversion of 1a	Yield of 2a (%)	1a-*d*
**1**	MeCN	37	20	-
**2**	MeCN-d_3	36	20	0%

Supplementary Table 9. Evaluation of deuteration of the tertiary C−H bond in MeCN-d_3.

Discussion

No deuteration of the tertiary C−H bond in **1a** was observed. This observation implies that the tertiary radical generated from HAT of **1a** is likely to undergo unspecific degradations if formation of the C(sp^3)−methyl bond is not fast enough.

3.9 Investigations of the Effects of Various Bidentate Additives

General Procedure J: Ir-F (1.2 mg, 0.5 mol%, 1.0 µmol), $Ni(acac)_2$ (2.6 mg, 5 mol%, 10.0 µmol), and dicumyl peroxide (1.20 mmol, 6 equiv, 324 mg) were weighed into a vial charged with a magnetic stirrer. If the additive is a solid, it was also weighed into the vial at this step (1 equiv). 0.2 mL degassed MeCN was then added, followed by isoamyl benzoate substrate **1a** (0.200 mmol, 39.0 µL). If the additive is a liquid, it was added to the mixture via a micropipette. The vial was sealed, and the headspace of the vial was flushed with a stream of N_2 for 1 min. The mixture was then stirred and irradiated with a blue LED for 16 hours with cooling fans maintaining the temperature at 25 °C. The reaction mixture was then concentrated *in vacuo* and filtered through a short silica plug, followed by rinsing the plug with CH_2Cl_2. Volatile materials were evaporated, and a known amount of 1,3,5-trimethoxybenzene was added as an internal standard. Crude ^1H NMR spectroscopy was performed in $CDCl_3$.

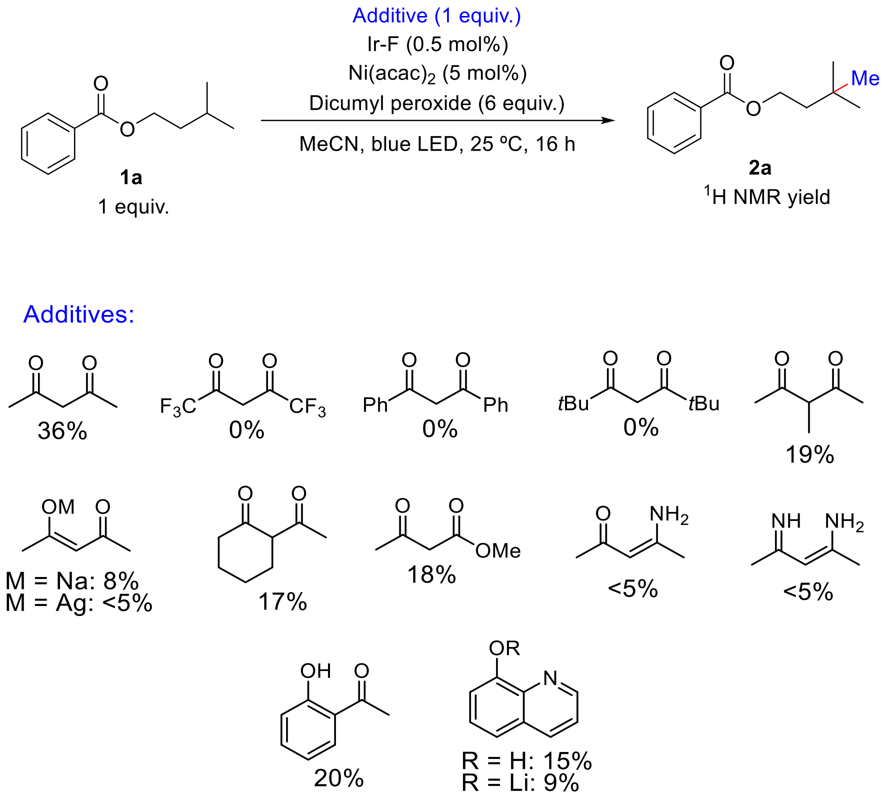

Supplementary Figure 7. Investigation of the effects of various bidentate additives.

3.10 Variations of the Stoichiometries of 2,4-Pentanedione

General Procedure K: Ir-F (1.2 mg, 0.5 mol%, 1.0 µmol), $Ni(acac)_2$ (2.6 mg, 5 mol%, 10.0 µmol), and dicumyl peroxide (1.20 mmol, 6 equiv, 324 mg) were weighed into a vial charged with a magnetic stirrer. 0.2 mL degassed MeCN was then added, followed by isoamyl benzoate substrate **1a** (0.200 mmol, 39.0 µL) and 2,4-pentanedione. The vial was sealed, and the headspace of the vial was flushed with a stream of N_2 for 1 min. The mixture was then stirred and irradiated with a blue LED for 16 hours with cooling fans maintaining the temperature at 25 °C.

After concentrating *in vacuo*, the crude residue was directly purified by flash column chromatography (SiO_2) eluting with EtOAc and hexane (1:9). In cases where the methylated product **2a** and the isoamyl benzoate substrate **1a** were inseparable, the mixed fractions were combined, concentrated, and subjected to reverse-phase C18 column chromatography eluting with a gradient of 10%→90% MeCN in H_2O (0.1% formic acid v/v) to give the desired methylated product after evaporations of volatile materials. (Except for entry **1**, all other entries are isolated yields)

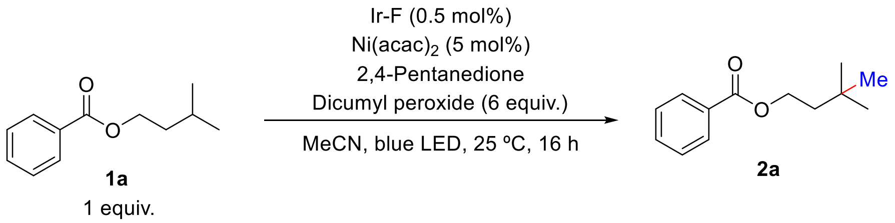

Entry	2,4-pentanedione equiv	Yield (%)
**1**	1	36
**2**	2	32
**3**	0.75	35
**4**	**0.5**	**36**
**5**	0.3	31
**6**	0.2	32

Supplementary Table 10. General Procedure K: 0.2 mmol **1a**, 1.0 µmol Ir-F, 10.0 µmol $Ni(acac)_2$, 1.2 mmol dicumyl peroxide, 2,4-pentanedione, in MeCN, irradiation under a blue LED Kessil lamp at 25 °C for 16 hours. Isolated yields (entries **2**−**6**).

Discussions

The amount of 2,4-pentanedione could be reduced to 0.5 equiv without compromising the yield of the reaction.

3.11 Evaluation of Peroxides

General Procedure L: Ir-F (1.2 mg, 0.5 mol%, 1.0 µmol), $Ni(acac)_2$ (2.6 mg, 5 mol%, 10.0 µmol), and dicumyl peroxide (1.20 mmol, 6 equiv, 324 mg) were weighed into a vial charged with a magnetic stirrer. The degassed solvent (0.2 mL) was added, followed by isoamyl benzoate substrate **1a** (0.200 mmol, 39.0 µL) and 2,4-pentanedione (10.0 µmol, 0.5 equiv, 10 µL). The vial was sealed, and the headspace of the vial was flushed with a stream of N_2 for 1 min. The mixture was then stirred and irradiated with a blue LED for 16 hours with cooling fans maintaining the temperature at 25 °C. After concentrating *in vacuo*, the crude residue was directly purified by flash column chromatography (SiO_2) eluting with EtOAc and hexane (1:9). In cases where the methylated product **2a** and the isoamyl benzoate substrate **1a** were inseparable, the mixed fractions were combined, concentrated, and subjected to reverse-phase C18 column chromatography with a gradient of 10%→90% MeCN in H_2O (0.1% formic acid v/v) to give the desired methylated product. (Except for entry **1**, all other entries are isolated yields)

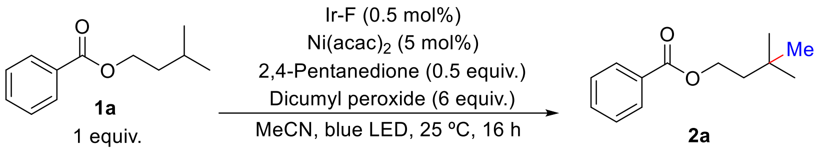

Entry	Deviations from standard condition	Yield (%)
**1**	-	36
**2**	6 equiv *t*BuOO*t*Bu in TFE	30
**3**	8 equiv cumyl tert-butyl peroxide in MeCN	28
**4**	8 equiv cumyl tert-butyl peroxide in MeCN and TFE (1:1)	48
**5**	6 equiv cumyl tert-butyl peroxide in MeCN and TFE (1:1)	34
**6**	**4 equiv dicumyl peroxide and 4 equiv *t*BuOO*t*Bu, in MeCN and TFE (1:1)**	**50**
**7**	3 equiv dicumyl peroxide and 3 equiv *t*BuOO*t*Bu, in MeCN and TFE (1:1)	41
**8**	5 equiv dicumyl peroxide and 5 equiv *t*BuOO*t*Bu, in MeCN and TFE (1:1)	50
**9**	4 equiv dicumyl peroxide and 4 equiv *t*BuOO*t*Bu, in MeCN	35
**10**	4 equiv dicumyl peroxide and 4 equiv *t*BuOO*t*Bu, in TFE	29
**11**[a]	4 equiv dicumyl peroxide and 4 equiv *t*BuOO*t*Bu, in MeCN and TFE (1:1)	15
**12**[b]	4 equiv dicumyl peroxide and 4 equiv *t*BuOO*t*Bu, in MeCN and TFE (1:1)	25
**13**	8 equiv 1,1-diphenyl ethyl tert-butyl peroxide, in MeCN and TFE (1:1)	28
**14**	4 equiv dicumyl peroxide and 8 equiv cumyl tert-butyl peroxide in MeCN	46
**15**[c]	4 equiv dicumyl peroxide and 4 equiv *t*BuOO*t*Bu, in MeCN and TFE (1:1)	27
**16**[d]	4 equiv dicumyl peroxide and 4 equiv *t*BuOO*t*Bu, in MeCN and TFE (1:1)	50

Supplementary Table 11. General Procedure L: 0.2 mmol **1a**, 1.0 µmol Ir-F, 10.0 µmol $Ni(acac)_2$, 0.8 mmol dicumyl peroxide, in stated solvents, irradiation under a blue LED Kessil lamp at 25 °C for 16 hours. Isolated yields. **a.** With 5 mol% $NiCl_2$.diglyme and 5 mol% 4,4',4"-^{t}Bu-tpy, 0.5 equiv $B(OH)_3$, no 2,4-pentanedione. **b**. With 5 mol%

NiCl$_2$.diglyme and 5 mol% 4,4',4''-^{t}Bu-tpy, no 2,4-pentanedione. **c**. No 2,4-pentanedione. **d**. Ni(dpm)$_2$ was used instead of Ni(acac)$_2$.

3.12 Evaluation of Protecting Groups on the Secondary Amine

Ir-F (1.2 mg, 0.5 mol%, 0.5 μmol), Ni(dpm)$_2$ (4.3 mg, 5 mol%, 10.0 μmol), and dicumyl peroxide (0.800 mmol, 4 equiv, 216 mg) were weighed into a vial charged with a magnetic stirrer. A pre-formed 1:1 mixture of degassed MeCN and TFE (0.2 mL) were added, followed by protected amine substrate (0.200 mmol, 1 equiv), di-tert-butyl peroxide (0.800 mmol, 4 equiv, 146 μL), and 2,4-pentanedione (10.0 μmol, 0.5 equiv, 10 μL). The vial was sealed, and the headspace of the vial was flushed with a stream of N_2 for 1 min. The mixture was then stirred and irradiated under a blue LED Kessil lamp for 16 hours with cooling fans maintaining the temperature at 25 °C. For entries **1**, **2**, **3**, **4** and **6**, the reaction mixture was concentrated *in vacuo* and filtered through a short silica plug, followed by rinsing the plug with CH_2Cl_2. Volatile materials were evaporated, and a known amount of 1,3,5-trimethoxybenzene was added as an internal standard. Crude ^{1}H NMR spectroscopy was performed in $CDCl_3$. For entry **5**, the reaction mixture was concentrated under reduced pressure, and the crude mixture was directly purified by flash column chromatography (SiO_2 deactivated with 5% Et_3N) eluting with hexane.

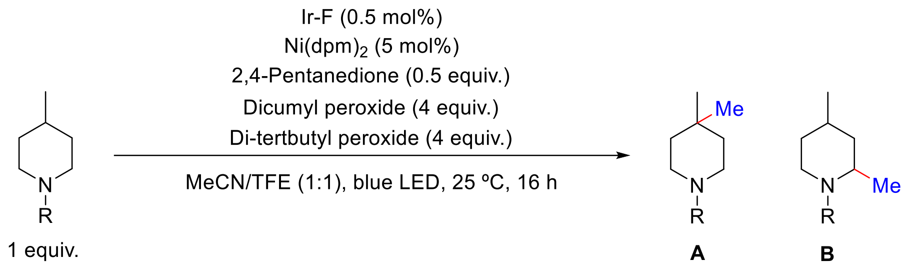

Entry	R	Observation
**1**	Bz	Complex mixture
**2**	Boc	Complex mixture
**3**	Trifluoroacetyl	Complex mixture
**4**	HCl salt	<5% conversion
**5**	BF_3 complex	41% **A** (isolated)
**6**	BF_3 complex (with Ni(acac)$_2$ instead of Ni(dpm)$_2$ as catalyst)	35% **A**

Supplementary Table 12. Evaluation of protecting groups on secondary amines.

4. General Procedures for the Methylation of Unactivated C(sp^3)−H Bonds

General Procedure M:

Ir-F (1.2 mg, 0.5 mol%, 1.0 μmol), Ni(acac)$_2$ (2.6 mg, 5 mol%, 10.0 μmol), and dicumyl peroxide (0.800 mmol, 4 equiv, 216 mg) were weighed into a vial charged with a magnetic stirrer. A pre-formed 1:1 mixture of degassed MeCN and TFE (0.2 mL) were added, followed by the substrate (0.200 mmol, 1 equiv), di-tert-butyl peroxide (0.800 mmol, 4 equiv, 146 μL), and 2,4-pentanedione (10.0 μmol, 0.5 equiv, 10 μL). The vial was sealed, and the headspace of the vial was flushed with a stream of N_2 for 1 min. The mixture was then stirred and irradiated under a blue LED Kessil lamp for 16 hours with cooling fans maintaining the temperature at 25 °C.

After concentrating *in vacuo*, the crude residue was directly purified by flash column chromatography (SiO_2) eluting with EtOAc and hexane. In cases where the methylated product and the substrate were inseparable, the mixed fractions were combined, concentrated, and subjected to reverse-phase C18 column chromatography with a gradient of 10%→90% MeCN in H_2O (0.1% formic acid v/v, unless otherwise stated) to give the desired methylated product after evaporations of volatile materials.

General Procedure N:

Ir-F (1.2 mg, 0.5 mol%, 0.5 μmol), Ni(dpm)$_2$ (4.3 mg, 5 mol%, 10.0 μmol), and dicumyl peroxide (0.800 mmol, 4 equiv, 216 mg) were weighed into a vial charged with a magnetic stirrer. A pre-formed 1:1 mixture of degassed MeCN and TFE (0.2 mL) were added, followed by the substrate (0.200 mmol, 1 equiv), di-tert-butyl peroxide (0.800 mmol, 4 equiv, 146 μL), and 2,4-pentanedione (10.0 μmol, 0.5 equiv, 10 μL). The vial was sealed, and the headspace of the vial was flushed with a stream of N_2 for 1 min. The mixture was then stirred and irradiated under a blue LED Kessil lamp for 16 hours with cooling fans maintaining the temperature at 25 °C.

After concentrating *in vacuo*, the crude residue was directly purified by flash column chromatography (SiO_2) eluting with EtOAc and hexane. In cases where the methylated product and the substrate were inseparable, the mixed fractions were combined, concentrated, and subjected to reverse-phase C18 column chromatography with a gradient of 10%→90% MeCN in H_2O (0.1% formic acid v/v, unless otherwise stated) to give the desired methylated product after evaporations of volatile materials.

5. Substrate Scope of the Methylation of Unactivated C(sp^3)–H Bonds

2a (3,3-dimethylbutyl benzoate):

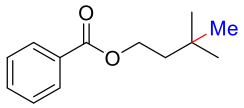

The titled compound was synthesized according to **General Procedure M**.

1a (38.5 mg, 0.20 mmol, 1.0 equiv) was allowed to react, and **2a** was isolated as a colorless oil (20.7 mg, 50% yield).

^{1}H NMR (500 MHz, $CDCl_3$) δ 8.07–8.01 (m, 2H), 7.59–7.52 (m, 1H), 7.48–7.39 (m, 2H), 4.39 (t, *J* = 7.2 Hz, 2H), 1.71 (t, *J* = 7.3 Hz, 2H), 1.00 (s, 9H).

^{13}C NMR (126 MHz, $CDCl_3$) δ 166.9, 132.9, 130.7, 129.7, 128.5, 62.9, 42.1, 29.8.

The spectra matched those reported in the literature.[11]

2b (4,4-dimethylpentyl 4-(trifluoromethyl)benzoate):

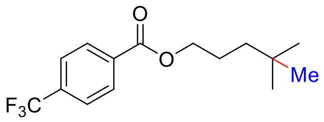

The titled compound was synthesized according to **General Procedure M**.

1b (54.9 mg, 0.20 mmol, 1.0 equiv) was allowed to react, and **2b** was isolated as a colorless oil (33.9 mg, 59% yield).

^{1}H NMR (500 MHz, $CDCl_3$) δ 8.16 (ddd, *J* = 7.9, 1.6, 0.9 Hz, 2H), 7.71 (dt, *J* = 8.1, 0.7 Hz, 2H), 4.33 (t, *J* = 6.8 Hz, 2H), 1.81–1.71 (m, 2H), 1.36–1.27 (m, 2H), 0.92 (s, 9H).

^{13}C NMR (126 MHz, $CDCl_3$) δ 134.49 (d, *J* = 32.6 Hz), 125.54 (q, *J* = 3.7 Hz), 123.81 (d, *J* = 272.5 Hz).

^{13}C NMR (126 MHz, $CDCl_3$) δ 165.6, 134.49 (q, *J* = 32.6 Hz), 133.9, 130.1, 125.54 (q, *J* = 3.7 Hz), 123.81 (q, *J* = 272.5 Hz), 66.6, 40.2, 30.3, 29.4, 24.3.

^{19}F NMR (470 MHz, $CDCl_3$) δ -63.09.

HRMS (m/z): (ESI+) calc'd for $C_{15}H_{20}F_3O_2$ $[M+H]^+$: 289.1415, found: 289.1416

2c (4,4-dimethylpentyl 4-cyanobenzoate):

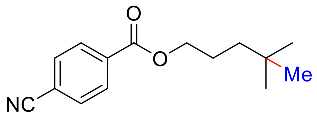

The titled compound was synthesized according to **General Procedure M**.

1c (46.3 mg, 0.20 mmol, 1.0 equiv) was allowed to react, and **2c** was isolated as a colorless oil (24.9 mg, 51% yield).

^{1}H NMR (500 MHz, $CDCl_3$) δ 8.16–8.12 (m, 2H), 7.77–7.72 (m, 2H), 4.33 (t, *J* = 6.8 Hz, 2H), 1.81–1.68 (m, 2H), 1.35–1.26 (m, 2H), 0.92 (s, 9H).

^{13}C NMR (126 MHz, $CDCl_3$) δ 165.1, 134.5, 132.4, 130.2, 118.2, 116.4, 66.8, 40.2, 30.3, 29.4, 24.2.

HRMS (m/z): (ESI+) calc'd for $C_{15}H_{20}NO_2$ $[M+H]^+$: 246.1494, found: 246.1498

2d (4,4-dimethylpentyl 4-methoxybenzoate):

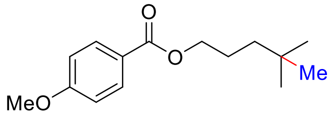

The titled compound was synthesized according to **General Procedure M**.

1d (47.3 mg, 0.20 mmol, 1.0 equiv) was allowed to react, and **2d** was isolated as a colorless oil (26.0 mg, 52% yield).

^{1}H NMR (500 MHz, $CDCl_3$) δ 8.00 (d, *J* = 8.9 Hz, 2H), 6.92 (d, *J* = 8.9 Hz, 2H), 4.26 (t, *J* = 6.8 Hz, 2H), 3.86 (s, 3H), 1.77–1.67 (m, 2H), 1.34–1.27 (m, 2H), 0.91 (s, 9H).

^{13}C NMR (126 MHz, $CDCl_3$) δ 166.6, 163.4, 131.7, 123.1, 113.7, 65.7, 55.6, 40.3, 30.3, 29.5, 24.4.

HRMS (m/z): (ESI+) calc'd for $C_{15}H_{23}O_2$ $[M+H]^+$: 251.1647, found: 251.1643

2e (4,4-dimethylpentyl 4-chlorobenzoate):

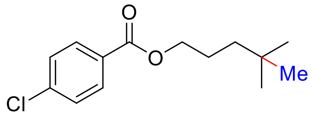

The titled compound was synthesized according to **General Procedure M**.

1e (48.1 mg, 0.20 mmol, 1.0 equiv) was allowed to react, and **2e** was isolated as a colorless oil (27.6 mg, 54% yield).

^{1}H NMR (500 MHz, $CDCl_3$) δ 7.83–7.78 (m, 2H), 7.76–7.72 (m, 2H), 4.28 (t, J = 6.8 Hz, 2H), 1.78–1.68 (m, 2H), 1.34–1.25 (m, 2H), 0.91 (s, 9H).

^{13}C NMR (126 MHz, $CDCl_3$) δ 165.97, 139.4, 131.1, 129.1, 128.8, 66.3, 40.2, 30.3, 29.5, 24.3.

HRMS (m/z): (ESI+) calc'd for $C_{14}H_{20}ClO_2$ $[M+H]^+$: 255.1152, found: 255.1153

2f (4,4-dimethylpentyl 4-bromobenzoate):

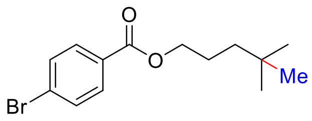

The titled compound was synthesized according to **General Procedure M**.

1f (57.0 mg, 0.20 mmol, 1.0 equiv) was allowed to react, and **2f** was isolated as a colorless oil (30.5 mg, 51% yield).

^{1}H NMR (500 MHz, $CDCl_3$) δ 7.90 (d, J = 8.5 Hz, 2H), 7.58 (d, J = 8.5 Hz, 2H), 4.29 (t, J = 6.8 Hz, 2H), 1.79–1.67 (m, 2H), 1.34–1.27 (m, 2H), 0.92 (s, 9H).

^{13}C NMR (126 MHz, $CDCl_3$) δ 166.1, 131.8, 131.2, 129.6, 128.0, 66.3, 40.2, 30.3, 29.4, 24.3.

HRMS (m/z): (ESI+) calc'd for $C_{14}H_{20}BrO_2$ $[M+H]^+$: 299.0641, found: 299.0635

2g (4,4-dimethylpentyl 4-iodobenzoate):

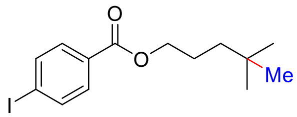

The titled compound was synthesized according to **General Procedure M**.

1g (66.4 mg, 0.20 mmol, 1.0 equiv) was allowed to react, and **2g** was isolated as a colorless oil (25.4 mg, 37% yield).

^{1}H NMR (500 MHz, $CDCl_3$) δ 7.80 (d, J = 8.5 Hz, 2H), 7.74 (d, J = 8.6 Hz, 2H), 4.28 (t, J = 6.8 Hz, 2H), 1.78–1.66 (m, 2H), 1.35–1.25 (m, 2H), 0.91 (s, 9H).

^{13}C NMR (126 MHz, $CDCl_3$) δ 166.3, 137.8, 131.2, 130.2, 100.7, 66.3, 40.2, 30.3, 29.4, 24.3.

HRMS (m/z): (ESI+) calc'd for $C_{14}H_{20}IO_2$ $[M+H]^+$: 347.0508, found: 347.0501

2h (4,4-dimethylpentyl 4-(4,4,5,5-tetramethyl-1,3,2-dioxaborolan-2-yl)benzoate):

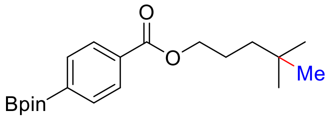

The titled compound was synthesized according to **General Procedure M**.

1h (66.5 mg, 0.20 mmol, 1.0 equiv) was allowed to react, and **2h** was isolated as a colorless oil (38.0 mg, 55% yield).

^{1}H NMR (500 MHz, $CDCl_3$) δ 8.02 (d, *J* = 8.2 Hz, 2H), 7.87 (d, *J* = 8.3 Hz, 2H), 4.30 (t, *J* = 6.9 Hz, 2H), 1.80–1.69 (m, 2H), 1.36 (s, 12H), 1.33–1.27 (m, 2H), 0.91 (s, 9H).

^{13}C NMR (126 MHz, $CDCl_3$) δ 166.9, 134.8, 132.9, 128.7, 84.3, 66.1, 40.2, 30.3, 29.5, 25.0, 24.3.

HRMS (m/z): (ESI+) calc'd for $C_{20}H_{32}BO_4$ $[M+H]^+$: 347.2388, found: 347.2392

2k (4,4-dimethylpentyl isoxazole-5-carboxylate):

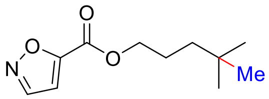

The titled compound was synthesized according to **General Procedure M**.

1k (39.4 mg, 0.20 mmol, 1.0 equiv) was allowed to react, and **2k** was isolated as a colorless oil (21.9 mg, 52% yield).

^{1}H NMR (500 MHz, $CDCl_3$) δ 8.36 (d, *J* z= 1.8 Hz, 1H), 6.95 (d, *J* = 1.8 Hz, 1H), 4.35 (t, *J* = 6.9 Hz, 2H), 1.80–1.70 (m, 2H), 1.33–1.25 (m, 2H), 0.91 (s, 9H).

^{13}C NMR (126 MHz, $CDCl_3$) δ 160.3, 156.9, 150.8, 108.8, 67.3, 39.97, 30.3, 29.4, 24.1.

HRMS (m/z): (ESI+) calc'd for $C_{11}H_{18}NO_3$ $[M+H]^+$: 212.1287, found: 212.1288

2l (4,4-dimethylpentyl thiazole-5-carboxylate):

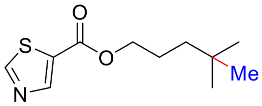

The titled compound was synthesized according to **General Procedure M**.

1l (42.7 mg, 0.20 mmol, 1.0 equiv) was allowed to react, and **2l** was isolated as a colorless oil (20.6 mg, 45% yield).

^{1}H NMR (500 MHz, $CDCl_3$) δ 8.93 (d, *J* = 0.7 Hz, 1H), 8.50 (d, *J* = 0.7 Hz, 1H), 4.30 (t, *J* = 6.8 Hz, 2H), 1.77–1.68 (m, 2H), 1.32–1.24 (m, 2H), 0.91 (s, 9H).

^{13}C NMR (126 MHz, $CDCl_3$) δ 161.4, 158.1, 148.9, 130.1, 66.8, 40.1, 30.3, 29.4, 24.2.

HRMS (m/z): (ESI+) calc'd for $C_{11}H_{18}NO_2S$ $[M+H]^+$: 228.1058, found: 228.1061

2m (4,4-dimethylpentyl 4-methylthiophene-2-carboxylate):

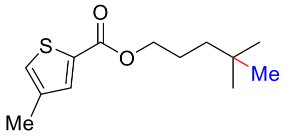

The titled compound was synthesized according to **General Procedure M**.

1m (45.3 mg, 0.20 mmol, 1.0 equiv) was allowed to react, and **2m** was isolated as a colorless oil (23.3 mg, 49% yield).

^{1}H NMR (500 MHz, $CDCl_3$) δ 7.59 (d, *J* = 1.5 Hz, 1H), 7.13 (dd, *J* = 1.7, 1.1 Hz, 1H), 4.24 (t, *J* = 6.8 Hz, 2H), 1.76–1.64 (m, 2H), 1.31–1.25 (m, 2H), 0.91 (s, 9H).

^{13}C NMR (126 MHz, $CDCl_3$) δ 162.6, 138.6, 135.2, 133.8, 128.0, 66.1, 40.1, 30.3, 29.4, 24.3, 15.7.

HRMS (m/z): (ESI+) calc'd for $C_{13}H_{21}O_2S$ $[M+H]^+$: 241.1262, found: 241.1260

2n (1-(tert-butyl) 6-(4,4-dimethylpentyl) 1H-indole-1,6-dicarboxylate):

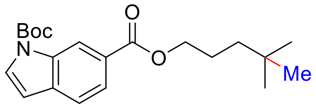

The titled compound was synthesized according to **General Procedure M**.

1n (69.1 mg, 0.20 mmol, 1.0 equiv) was allowed to react, and **2n** was isolated as a colorless oil (29.5 mg, 41% yield).

^{1}H NMR (500 MHz, $CDCl_3$) δ 8.85 (s, 1H), 7.94 (dd, *J* = 8.2, 1.5 Hz, 1H), 7.76 (d, *J* = 3.7 Hz, 1H), 7.59 (dd, *J* = 8.2, 0.7 Hz, 1H), 6.62 (dd, *J* = 3.7, 0.8 Hz, 1H), 4.32 (t, *J* = 6.9 Hz, 2H), 1.82–1.74 (m, 2H), 1.71 (s, 9H), 1.37–1.30 (m, 2H), 0.92 (s, 9H).

^{13}C NMR (126 MHz, $CDCl_3$) δ 167.4, 149.6, 134.7, 134.4, 128.96, 126.5, 123.97, 120.7, 117.3, 107.3, 84.5, 65.98, 40.3, 30.3, 29.5, 28.3, 24.4.

HRMS (m/z): (ESI+) calc'd for $C_{21}H_{30}NO_4$ $[M+H]^+$: 360.2175, found: 360.2172

2o (4,4-dimethylpentyl nicotinate):

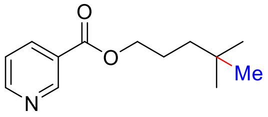

The titled compound was synthesized according to **General Procedure M**.

1o (41.5 mg, 0.20 mmol, 1.0 equiv) was allowed to react, and **2o** was isolated as a colorless oil (17.0 mg, 38% yield).

^{1}H NMR (500 MHz, $CDCl_3$) δ 9.23 (dd, *J* = 2.2, 0.9 Hz, 1H), 8.78 (dd, *J* = 4.8, 1.8 Hz, 1H), 8.30 (dt, *J* = 7.9, 2.0 Hz, 1H), 7.39 (ddd, *J* = 7.9, 4.8, 0.9 Hz, 1H), 4.33 (t, *J* = 6.8 Hz, 2H), 1.82–1.69 (m, 2H), 1.37–1.27 (m, 2H), 0.92 (s, 9H).

^{13}C NMR (126 MHz, $CDCl_3$) δ 165.5, 153.5, 151.1, 137.2, 126.5, 123.4, 66.5, 40.2, 30.3, 29.4, 24.2.

HRMS (m/z): (ESI+) calc'd for $C_{13}H_{20}NO_2$ $[M+H]^+$: 222.1494, found: 222.1490

2p (4,4-dimethylpentyl quinoline-3-carboxylate):

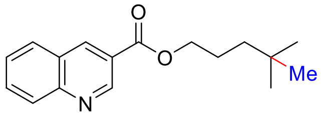

The titled compound was synthesized according to **General Procedure M**.

1p (51.5 mg, 0.20 mmol, 1.0 equiv) was allowed to react, and **2p** was isolated as a colorless oil (13.7 mg, 25% yield).

^{1}H NMR (500 MHz, $CDCl_3$) δ 9.46 (d, *J* = 2.1 Hz, 1H), 8.84 (dd, *J* = 2.1, 0.9 Hz, 1H), 8.17 (dq, *J* = 8.5, 0.9 Hz, 1H), 7.95 (dd, *J* = 8.2, 1.4 Hz, 1H), 7.84 (ddd, *J* = 8.4, 6.9, 1.4 Hz, 1H), 7.63 (ddd, *J* = 8.1, 6.9, 1.2 Hz, 1H), 4.40 (t, *J* = 6.8 Hz, 2H), 1.87–1.75 (m, 2H), 1.38–1.31 (m, 2H), 0.94 (s, 9H).

^{13}C NMR (126 MHz, $CDCl_3$) δ 165.6, 150.3, 149.98, 138.8, 131.9, 129.7, 129.3, 127.6, 127.0, 123.5, 66.5, 40.2, 30.3, 29.5, 24.3.

HRMS (m/z): (ESI+) calc'd for $C_{17}H_{22}NO_2$ $[M+H]^+$: 272.1651, found: 272.1655

2q (4,4-dimethylpentyl quinoline-6-carboxylate):

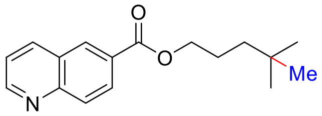

The titled compound was synthesized according to **General Procedure M**.

1q (51.5 mg, 0.20 mmol, 1.0 equiv) was allowed to react, and **2q** was isolated as a colorless oil (12.4 mg, 23% yield).

^{1}H NMR (500 MHz, $CDCl_3$) δ 9.01 (dd, *J* = 4.2, 1.8 Hz, 1H), 8.60 (d, *J* = 1.9 Hz, 1H), 8.35–8.26 (m, 2H), 8.16 (d, *J* = 8.8 Hz, 1H), 7.48 (dd, *J* = 8.3, 4.2 Hz, 1H), 4.38 (t, *J* = 6.8 Hz, 2H), 1.84–1.75 (m, 2H), 1.40–1.30 (m, 2H), 0.94 (s, 9H).

^{13}C NMR (126 MHz, $CDCl_3$) δ 166.3, 152.6, 150.2, 137.5, 131.0, 129.9, 129.1, 128.7, 127.6, 121.96, 66.5, 40.3, 30.3, 29.5, 24.4.

HRMS (m/z): (ESI+) calc'd for $C_{17}H_{22}NO_2$ $[M+H]^+$: 272.1651, found: 272.1656

2r (4,4-dimethylpentyl quinoline-6-carboxylate):

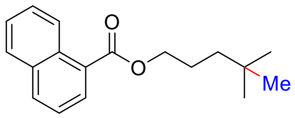

The titled compound was synthesized according to **General Procedure M**.

1r (51.3 mg, 0.20 mmol, 1.0 equiv) was allowed to react, and **2r** was isolated as a white solid (32.3 mg, 60% yield).

^{1}H NMR (500 MHz, $CDCl_3$) δ 8.91 (dq, *J* = 8.7, 0.9 Hz, 1H), 8.18 (dd, *J* = 7.3, 1.3 Hz, 1H), 8.02 (dt, *J* = 8.3, 1.1 Hz, 1H), 7.93–7.86 (m, 1H), 7.61 (ddd, *J* = 8.5, 6.8, 1.4 Hz, 1H), 7.57–7.48 (m, 2H), 4.40 (t, *J* = 6.8 Hz, 2H), 1.85–1.75 (m, 2H), 1.40–1.32 (m, 2H), 0.94 (s, 9H).

^{13}C NMR (126 MHz, $CDCl_3$) δ 167.9, 133.99, 133.3, 131.5, 130.2, 128.7, 127.8, 127.7, 126.3, 126.0, 124.7, 66.2, 40.4, 30.3, 29.5, 24.4.

HRMS (m/z): (ESI+) calc'd for $C_{18}H_{23}O_2$ $[M+H]^+$: 271.1698, found: 271.1695

2s (methyl (S)-2-(1,3-dioxoisoindolin-2-yl)-4,4-dimethylpentanoate):

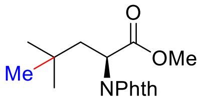

The titled compound was synthesized according to **General Procedure M**.

1s (55.1 mg, 0.20 mmol, 1.0 equiv) was allowed to react, and **2s** was isolated as a white solid (35.8 mg, 62% yield).

^{1}H NMR (500 MHz, $CDCl_3$) δ 7.87 (dd, *J* = 5.5, 3.1 Hz, 2H), 7.74 (dd, *J* = 5.5, 3.0 Hz, 2H), 4.96 (dd, *J* = 9.6, 3.2 Hz, 1H), 3.71 (s, 3H), 2.33 – 2.17 (m, 2H), 0.92 (s, 9H).

^{13}C NMR (126 MHz, $CDCl_3$) δ 170.7, 167.9, 134.3, 132.1, 123.7, 53.0, 49.7, 41.4, 30.5, 29.3.

HRMS (m/z): (ESI+) calc'd for $C_{16}H_{20}NO_4$ $[M+H]^+$: 290.1392, found: 290.1394

Reaction to form 2s on 5 mmol scale:

Ir-F (28 mg, 0.5 mol%, 25.0 μmol), $Ni(acac)_2$ (24.7 mg, 5 mol%, 250.0 μmol), and dicumyl peroxide (5.41 g, 4 equiv, 20.0 mmol) were weighed into a vial charged with a magnetic stirrer. A pre-formed 1:1 mixture of degassed MeCN and TFE (2 mL) were added, followed by substrate **1s** (5.0 mmol, 1 equiv, 1.38 g), di-tert-butyl peroxide (3.66 mL, 4 equiv, 20.0 mmol), and 2,4-pentanedione (2.5 mmol, 0.5 equiv, 255 μL). The vial was sealed, the mixture was then stirred and irradiated under two blue LED Kessil lamps for 16 hours with cooling fans maintaining the temperature at 25 °C. After concentrating *in vacuo*, the crude residue was directly purified by flash column chromatography (SiO_2) eluting with EtOAc and hexane. The mixed fractions containing the methylated product and the substrate were combined, concentrated, and subjected to reverse-phase column chromatography (with a 43 g C18 cartridge) with a gradient of 10%→90% MeCN in H_2O (0.1% formic acid v/v) to give **2s** (735.6 mg, 51% yield) after evaporation of volatile materials.

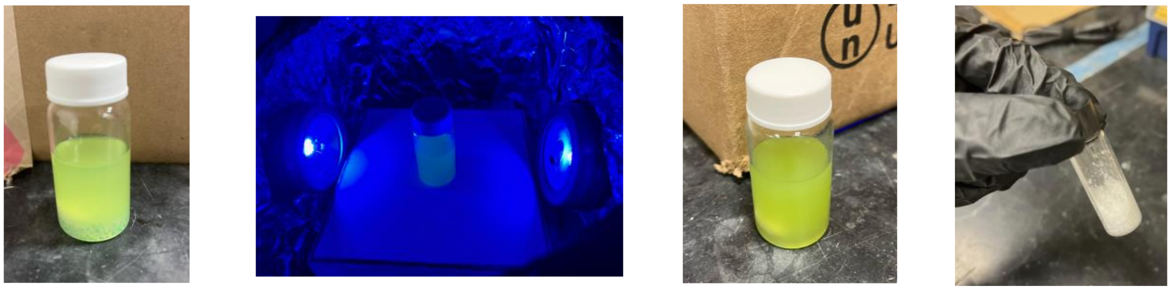

Before reaction | During reaction | After reaction | Product

Supplementary Figure 8. Photographs of the reaction to form **2s** on a 5 mmol scale.

2t ((S)-2-(1,3-dioxoisoindolin-2-yl)-4,4-dimethylpentanoic acid):

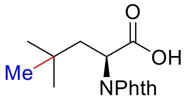

The titled compound was synthesized according to **General Procedure M**.

1t (52.3 mg, 0.20 mmol, 1.0 equiv) was allowed to react, and **2t** was isolated as a white solid (16.7 mg, 30% yield).

^1H NMR (500 MHz, $CDCl_3$) δ 7.85 (dd, *J* = 5.5, 3.1 Hz, 2H), 7.72 (dd, *J* = 5.5, 3.1 Hz, 2H), 4.99 (dd, *J* = 9.7, 2.8 Hz, 1H), 2.30 (dd, *J* = 15.0, 9.7 Hz, 1H), 2.20 (dd, *J* = 15.0, 2.8 Hz, 1H), 0.91 (s, 9H).

^{13}C NMR (126 MHz, $CDCl_3$) δ 175.2, 168.0, 134.3, 132.0, 123.7, 41.4, 30.5, 29.7, 29.3.

HRMS (m/z): (ESI+) calc'd for $C_{15}H_{18}NO_4$ $[M+H]^+$: 276.1236, found: 276.1237

2u ((R)-2-((tert-butoxycarbonyl)amino)-4,4-dimethylpentyl benzoate):

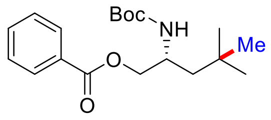

The titled compound was synthesized according to **General Procedure M**.

1u (64.3 mg, 0.20 mmol, 1.0 equiv) was allowed to react, and **2u** was isolated as a colorless solid (26.8 mg, 40% yield).

^1H NMR (500 MHz, $CDCl_3$) δ 8.04 (dd, J = 8.3, 1.4 Hz, 2H), 7.58–7.52 (m, 1H), 7.43 (t, J = 7.8 Hz, 2H), 4.47 (d, J = 9.3 Hz, 1H), 4.21 (d, J = 5.7 Hz, 2H), 4.17–4.09 (m, 1H), 1.53–1.44 (m, 1H), 1.39 (s, 9H), 1.34 (dd, J = 14.5, 8.9 Hz, 1H), 0.98 (s, 9H).

^{13}C NMR (126 MHz, $CDCl_3$) δ 166.6, 155.2, 133.1, 130.2, 129.8, 128.5, 79.5, 68.5, 47.1, 45.8, 30.6, 29.9, 28.5.

The spectra matched those reported in the literature.[10]

2v (methyl (S)-4-((tert-butoxycarbonyl)amino)-5-(((S)-1-methoxy-4,4-dimethyl-1-oxopentan-2-yl)amino)-5-oxopentanoate):

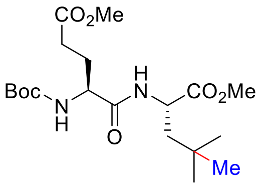

The titled compound was synthesized according to **General Procedure M**.

1v (83.7 mg, 0.20 mmol, 1.0 equiv) was allowed to react, and **2v** was isolated as a white solid (35.2 mg, 41% yield).

^1H NMR (500 MHz, $CDCl_3$) δ 6.68 (d, J = 8.5 Hz, 1H), 5.24 (d, J = 8.2 Hz, 1H), 4.58 (td, J = 8.6, 3.5 Hz, 1H), 4.24–4.10 (m, 1H), 3.70 (s, 3H), 3.68 (s, 3H), 2.57–2.37 (m, 2H), 2.18–2.07 (m, 1H), 1.91 (dq, J = 14.5, 7.3 Hz, 1H), 1.78 (dd, J = 14.4, 3.5 Hz, 1H), 1.49 (dd, J = 14.5, 8.9 Hz, 1H), 1.42 (s, 9H), 0.94 (s, 9H).

^{13}C NMR (126 MHz, $CDCl_3$) δ 174.1, 173.6, 171.2, 155.7, 80.2, 53.5, 52.5, 51.99, 50.1, 46.0, 30.8, 30.3, 29.6, 28.4, 28.1.

HRMS (m/z): (ESI+) calc'd for $C_{19}H_{35}N_2O_7$ $[M+H]^+$: 403.2444, found: 403.2449

2w (4,4-dimethylpentyl (2S,5R)-3,3-dimethyl-7-oxo-4-thia-1-azabicyclo[3.2.0]heptane-2-carboxylate 4,4-dioxide):

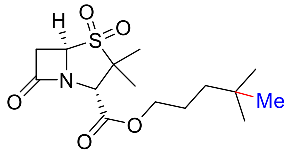

The titled compound was synthesized according to **General Procedure M**.

1w (63.5 mg, 0.20 mmol, 1.0 equiv) was allowed to react, and **2w** was isolated as a white solid (39.7 mg, 60% yield).

^{1}H NMR (500 MHz, $CDCl_3$) δ 4.61 (dd, *J* = 4.2, 2.2 Hz, 1H), 4.38 (s, 1H), 4.18 (td, *J* = 6.9, 0.9 Hz, 2H), 3.54–3.41 (m, 2H), 1.70–1.63 (m, 2H), 1.62 (s, 3H), 1.42 (s, 3H), 1.27–1.18 (m, 2H), 0.90 (s, 9H).

^{13}C NMR (126 MHz, $CDCl_3$) δ 170.9, 167.1, 67.5, 63.4, 62.8, 61.3, 40.1, 38.5, 30.2, 29.4, 24.1, 20.5, 18.8.

HRMS (m/z): (ESI+) calc'd for $C_{15}H_{26}NO_5S$ $[M+H]^+$: 332.1532, found: 332.1533

2x (4,4-dimethylpentyl 2-((3-(trifluoromethyl)phenyl)amino)nicotinate):

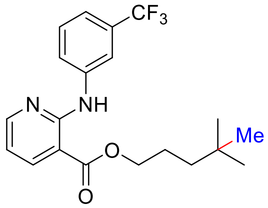

The titled compound was synthesized according to **General Procedure M**.

1x (73.3 mg, 0.20 mmol, 1.0 equiv) was allowed to react, and **2x** was isolated as a colorless oil (45.1 mg, 59% yield).

^{1}H NMR (500 MHz, $CDCl_3$) δ 10.43 (s, 1H), 8.42 (dd, *J* = 4.9, 2.0 Hz, 1H), 8.29 (dd, *J* = 7.8, 2.0 Hz, 1H), 8.08 (s, 1H), 7.96–7.83 (m, 1H), 7.44 (t, *J* = 8.0 Hz, 1H), 7.29 (d, *J* = 7.8 Hz, 1H), 6.81 (dd, *J* = 7.8, 4.8 Hz, 1H), 4.32 (t, *J* = 6.8 Hz, 2H), 1.85–1.71 (m, 2H), 1.36–1.27 (m, 2H), 0.94 (s, 9H).

^{13}C NMR (126 MHz, $CDCl_3$) δ 167.67, 155.92, 153.08, 140.53, 140.30, 131.27 (q, *J* = 32.1 Hz), 129.32, 124.34 (q, *J* = 272.4 Hz), 123.57, 119.08 (q, *J* = 3.9 Hz), 117.21 (q, *J* = 4.0 Hz), 114.20, 107.89, 66.49, 40.26, 30.31, 29.44, 24.22.

^{19}F NMR (470 MHz, $CDCl_3$) δ -62.62.

HRMS (m/z): (ESI+) calc'd for $C_{20}H_{24}F_3N_2O_2$ $[M+H]^+$: 381.1790, found: 381.1789

2y (42-(1-(3,3-dimethylbutyl)-2,6-dioxopiperidin-3-yl)isoindoline-1,3-dione):

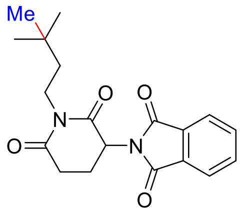

The titled compound was synthesized according to **General Procedure M**.

1y (65.7 mg, 0.20 mmol, 1.0 equiv) was allowed to react, and **2y** was isolated as a white solid (37.2 mg, 54% yield).

^{1}H NMR (500 MHz, $CDCl_3$) δ 7.81 (dd, *J* = 5.4, 3.0 Hz, 2H), 7.69 (dd, *J* = 5.4, 3.0 Hz, 2H), 4.94–4.86 (m, 1H), 3.80–3.71 (m, 2H), 2.96–2.86 (m, 1H), 2.79–2.63 (m, 2H), 2.08–2.00 (m, 1H), 1.41–1.29 (m, 2H), 0.87 (s, 9H).

^{13}C NMR (126 MHz, $CDCl_3$) δ 170.8, 168.5, 167.6, 134.5, 131.9, 123.9, 50.4, 41.0, 39.5, 37.8, 36.6, 32.2, 30.1, 29.3, 26.4, 22.6, 22.1.

HRMS (m/z): (ESI+) calc'd for $C_{19}H_{23}N_2O_4$ $[M+H]^+$: 343.1658, found: 343.1659

2z (4,4-dimethylpentyl 2-(4-(4-chlorobenzoyl)phenoxy)-2-methylpropanoate):

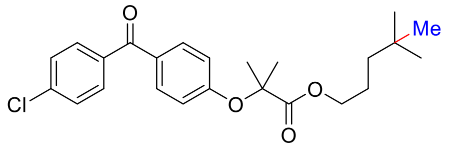

The titled compound was synthesized according to **General Procedure M**.

1z (83.4 mg, 0.20 mmol, 1.0 equiv) was allowed to react, and **2z** was isolated as a colorless oil (44.0 mg, 51% yield).

^{1}H NMR (500 MHz, $CDCl_3$) δ 7.72 (d, *J* = 8.9 Hz, 2H), 7.68 (d, *J* = 8.5 Hz, 2H), 7.43 (d, *J* = 8.5 Hz, 2H), 6.85 (d, *J* = 8.8 Hz, 2H), 4.13 (t, *J* = 6.7 Hz, 2H), 1.67 (s, 6H), 1.59–1.50 (m, 2H), 1.12–1.02 (m, 2H), 0.80 (s, 9H).

^{13}C NMR (126 MHz, $CDCl_3$) δ 194.2, 173.8, 159.8, 138.4, 136.5, 132.1, 131.2, 130.4, 128.6, 117.3, 79.5, 66.7, 40.0, 30.1, 29.3, 25.6, 24.0, 22.5.

HRMS (m/z): (ESI+) calc'd for $C_{24}H_{30}ClO_4$ $[M+H]^+$: 417.1830, found: 417.1839

2aa ((3aR,6R,7R,8aS)-3,3,6,8,8-pentamethyloctahydro-1H-3a,7-methanoazulen-6-yl acetate):

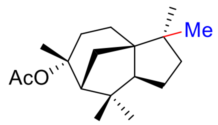

The titled compound was synthesized according to **General Procedure M**, but at the scale of 0.150 mmol.

1aa (39.7 mg, 0.150 mmol, 1.0 equiv) was allowed to react, and **2aa** was isolated as a colorless oil (13.8 mg, 33% yield).

^{1}H NMR (500 MHz, $CDCl_3$) δ 2.46–2.35 (m, 1H), 2.07–1.99 (m, 1H), 1.99–0.78 (m, 28H).

^{13}C NMR (126 MHz, $CDCl_3$) δ 170.53, 86.42, 56.98, 56.85, 54.12, 43.49, 41.47, 41.11, 37.10, 33.34, 31.36, 28.62, 27.09, 27.06, 26.00, 25.43, 22.94, 15.65.

HRMS (m/z): (ESI+) calc'd for $C_{18}H_{30}O_2Na$ $[M+Na]^+$: 301.2143, found: 301.2145

2ab (methyl (1S,4aR,4bR,7S,9aS,10S,10aR)-2-acetoxy-7-hydroxy-1,8,8-trimethyl-13-oxododecahydro-4a,1-(epoxymethano)-7,9a-methanobenzo[a]azulene-10-carboxylate):

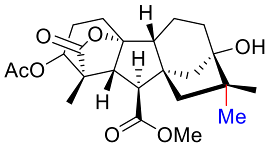

The titled compound was synthesized according to **General Procedure M**.

1ab (81.3 mg, 0.20 mmol, 1.0 equiv) was allowed to react, and **2ab** was isolated as a colorless gum (41.0 mg, 49% yield).

^{1}H NMR (500 MHz, $CDCl_3$) δ 4.95 (brs, 1H), 3.72 (s, 3H), 3.10 (d, J = 10.1 Hz, 1H), 2.61 (d, J = 10.1 Hz, 1H), 2.12 (s, 3H), 2.05–1.24 (m, 14H), 1.04–1.00 (m, 9H).

^{13}C NMR (126 MHz, $CDCl_3$) δ 177.0, 173.3, 170.4, 93.3, 78.7, 71.6, 55.6, 53.4, 52.5, 52.2, 51.2, 46.6, 43.8, 41.9, 30.0, 27.6, 27.1, 25.6, 21.3, 16.9, 15.3, 14.6.

HRMS (m/z): (ESI+) calc'd for $C_{23}H_{33}O_7$ $[M+H]^+$: 421.2226, found: 421.2228

5a ((Trifluoro(4,4-dimethylpiperidine-1-ium-yl)borate):

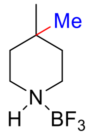

The titled compound was synthesized according to **General Procedure N**.

4a (33.4 mg, 0.20 mmol, 1.0 equiv) was allowed to react, and **5a** was isolated as a white solid (15.0 mg, 41% yield).

^{1}H NMR (500 MHz, $CDCl_3$) δ 3.52 (s, 1H), 3.23 (dq, *J* = 13.7, 2.4 Hz, 2H), 2.88 (tdd, *J* = 13.7, 11.6, 3.1 Hz, 2H), 1.57 (dd, *J* = 14.7, 2.6 Hz, 2H), 1.49 (td, *J* = 14.0, 4.4 Hz, 2H), 1.02 (s, 6H).

^{13}C NMR (126 MHz, $CDCl_3$) δ 42.2, 37.3, 32.1, 28.3, 23.4.

^{19}F NMR (470 MHz, $CDCl_3$) δ -157.6 (dd, *J* = 32.6, 16.0 Hz).

HRMS (m/z): (ESI+) calc'd for $[C_7H_{16}N]^+$: 114.1277, found: 114.1278 (the BF_3 adduct is unstable and spontaneously decomplexes on HRMS)

5b (Trifluoro(N-benzyl-N-(3,3-dimethylbutyl)amino)borate):

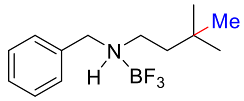

The titled compound was synthesized according to **General Procedure N**.

4b (49.0 mg, 0.20 mmol, 1.0 equiv) was allowed to react, and **5b** was afforded as a colorless oil (19.5 mg, 38% yield).

^{1}H NMR (500 MHz, $CDCl_3$) δ 7.47–7.32 (m, 5H), 4.13 (s, 2H), 2.93–2.79 (m, 2H), 1.67–1.49 (m, 2H), 0.86 (s, 9H).

^{13}C NMR (126 MHz, $CDCl_3$) δ 132.0, 129.9, 129.3, 129.3, 51.6, 43.6, 39.6, 29.9, 29.3.

^{19}F NMR (470 MHz, $CDCl_3$) δ -153.64 (dd, *J* = 32.8, 16.0 Hz).

HRMS (m/z): (ESI+) calc'd for $[C_{13}H_{22}N]^+$: 192.1747, found: 192.1747 (the BF_3 adduct is unstable and spontaneously decomplexes on HRMS)

5c (3,3-dimethylbutyl 2-(trifluoro-l4-boraneyl)isoindoline-1-carboxylate):

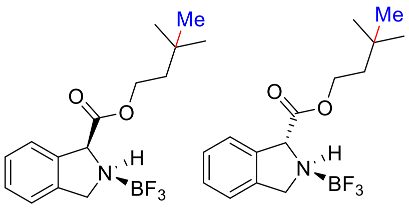

The titled compound was synthesized according to **General Procedure N**.

4c (60.2 mg, 0.20 mmol, 1.0 equiv) was allowed to react, and **5c** was afforded (21.9 mg, 35% yield) as a mixture of diastereomers (1.7:1) in the form of a colorless oil.

Major diastereomer: ^{1}H NMR (500 MHz, $CDCl_3$) δ 7.51–7.28 (m, 4H, overlapped), 6.65 (brs, 1H), 5.35 (s, 1H), 4.64 (s, 2H), 4.45–4.31 (m, 2H), 1.74–1.64 (m, 2H), 0.97 (s, 9H).

Minor diasteromer: ^{1}H NMR (500 MHz, $CDCl_3$) δ 7.51–7.28 (m, 4H, overlapped), 6.11 (brs, 1H), 5.63 (s, 1H), 4.88–4.76 (m, 2H), 4.26 (t, *J* = 7.6 Hz, 2H), 1.64–1.53 (m, 2H), 0.91 (s, 9H).

^{13}C NMR (126 MHz, $CDCl_3$) δ 169.2, 168.5, 135.5, 135.4, 133.0, 132.8, 130.1, 129.9, 129.0, 128.8, 123.6, 123.2, 123.0, 66.1, 65.8, 65.3, 65.3, 52.7, 52.1, 41.7, 41.5, 29.9, 29.8, 29.6, 29.6.

^{19}F NMR (470 MHz, $CDCl_3$) δ -156.51 (dd, *J* = 32.6, 16.0 Hz).

HRMS (m/z): (ESI+) calc'd for $[C_{15}H_{22}NO_2]^+$: 248.1651, found: 248.1645 (the BF_3 adduct is unstable and spontaneously decomplexes on HRMS)

6 (3,3-dimethylbutyl isoindoline-1-carboxylate):

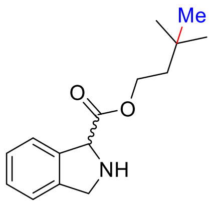

The title compound was synthesized according to the deprotection procedure adapted from Dieter and Watson.[12] To a 2 dram vial equipped with a magnetic stir bar was added **5c** (79 mg, 0.250 mmol, 1.0 equiv), cesium fluoride (190 mg, 1.25 mmol, 5.0 equiv) and MeCN (1.5 mL). The vial was sealed and heated to 85 ºC. The reaction was monitored by TLC. The reaction was basified with 1 M NaOH (20 mL) and extracted with CH_2Cl_2 (3 x 10 mL). The combined organic layer was dried over anhydrous Na_2SO_4, filtered, and concentrated to afford **6** as a pale-yellow oil (49.3 mg, 80% yield). No further purification was required.

^{1}H NMR (500 MHz, $CDCl_3$) δ 7.48–7.44 (m, 1H), 7.30–7.26 (m, 1H), 7.25–7.22 (m, 2H), 4.99 (s, 1H), 4.49 (dd, *J* = 14.0, 2.4 Hz, 1H), 4.31–4.16 (m, 3H), 2.15 (brs, 1H), 1.71–1.53 (m, 2H), 0.95 (s, 9H).

^{13}C NMR (126 MHz, $CDCl_3$) δ 173.2, 141.4, 138.3, 128.3, 127.1, 123.5, 122.8, 66.1, 63.5, 52.6, 41.9, 29.9, 29.7.

HRMS (m/z): (ESI+) calc'd for $C_{15}H_{22}NO_2$ $[M+H]^+$: 248.1651, found: 248.1646

6. Mechanistic Investigations

6.1 Studies of Potential Inhibition of the Reaction by the Products

Ir-F (1.2 mg, 1 mol%, 1.0 µmol), $Ni(acac)_2$ (1.3 mg, 5 mol%, 5.0 µmol), and dicumyl peroxide (0.600 mmol, 6 equiv, 162 mg) were weighed into a vial charged with a magnetic stirrer. 0.3 mL degassed MeCN was added, followed by isoamyl benzoate substrate **1a** (0.100 mmol, 1 equiv, 20 µL) and additive (3 equiv). The vial was sealed, and the headspace of the vial was flushed with a stream of N_2 for 1 min. The mixture was then stirred and irradiated with a blue LED for 16 hours with cooling fans maintaining the temperature at 25 °C. The reaction mixture was concentrated *in vacuo* and filtered through a short silica plug, followed by rinsing the plug with CH_2Cl_2. 1,3,5-trimethoxybenzene was added as an internal standard. Crude ^1H NMR spectroscopy was performed in $CDCl_3$.

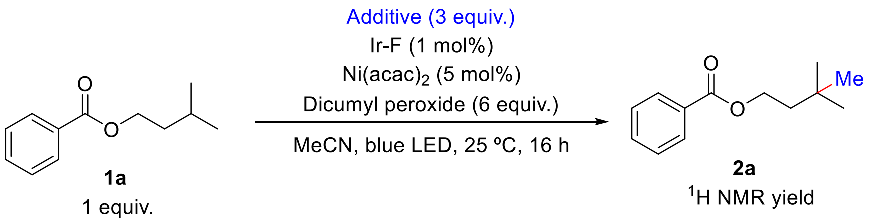

Entry	Additive	Yield 2a (%)
**1**	None	20
**2**	**2a**	20
**3**	α-Cumyl alcohol	21
**4**	Acetophenone	18

Supplementary Table 13. Condition: 0.1 mmol **1a**, 1.0 µmol Ir-F, 5.0 µmol $Ni(acac)_2$, 0.6 mmol dicumyl peroxide, 3 equiv additive, in MeCN, irradiation under a blue LED Kessil lamp at 25 °C for 16 hours. Yields were determined by ^1H NMR with an internal standard.

Discussion

The three defined, isolable products from the standard reaction are methylated product **2a**, α-cumyl alcohol, and acetophenone. The presence of these products at the beginning of the reaction was found to have no significant effect on the outcome of the reaction, therefore excluding the possibility of inhibition of the reaction by these compounds.

6.2 The Effect of the Stoichiometries of [Ir] and [Ni] on the Conversion of Dicumyl Peroxide

Ir-F (x mol%), Ni(acac)$_2$ (y mol%), and dicumyl peroxide (0.600 mmol, 6 equiv, 162 mg) were weighed into a vial charged with a magnetic stirrer. 0.3 mL degassed MeCN was added, followed by isoamyl benzoate substrate **1a** (0.100 mmol, 1 equiv, 20 μL). The vial was sealed, and the headspace of the vial was flushed with a stream of N_2 for 1 min. The mixture was then stirred and irradiated with a blue LED for 16 hours with cooling fans maintaining the temperature at 25 °C. The reaction mixture was concentrated *in vacuo* and filtered through a short silica plug, followed by rinsing the plug with CH_2Cl_2. 1,3,5-trimethoxybenzene was added as an internal standard. Crude ^{1}H NMR spectroscopy was performed in $CDCl_3$.

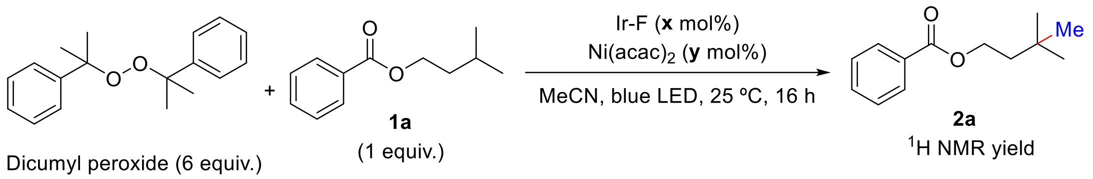

Entry	x	y	Conversion of dicumyl peroxide (%)	Yield 2a (%)
**1**	1	5	69	20
**2**	0.5	5	71	20
**3**	2	5	35	12
**4**	2	10	66	20
**5**	1	0	14	0

Supplementary Table 14. 0.1 mmol **1a**, 1.0 μmol Ir-F, 5.0 μmol Ni(acac)$_2$, 0.6 mmol dicumyl peroxide, in MeCN, irradiation under a blue LED Kessil lamp at 25 °C for 16 hours. Conversions of dicumyl peroxide and yields of **2a** were determined by ^{1}H NMR with an internal standard.

Discussion

The amount of Ir-F could be lowered to 0.5 mol% without impacting the conversion of peroxide (**Entry 2**). However, increasing the loading of Ir-F to 2 mol% led to a significant drop in both the conversion of dicumyl peroxide and the yield of **2a** (**Entry 3**). This suggests that a higher loading of Ir-F potentially catalyses the inhibition of the ability of Ir-F to activate the peroxide. The proportional increase of the loading of Ni(acac)$_2$ restored the capability of Ir-F to activate the peroxide (**Entry 4**), while the absence of Ni(acac)$_2$ greatly diminished the activity of Ir-F (**Entry 5**). The presence of the nickel catalyst appears to have a beneficial effect in prolonging the catalytic turnover of Ir-F in the photolysis of dicumyl peroxide.

6.3 Investigations of the Post-reaction Fate of Ir-F

4 hours, with Ni(acac)$_2$:

Ir-F (1.2 mg, 1 mol%, 1.0 µmol), Ni(acac)$_2$ (1.3 mg, 5 mol%, 5.0 µmol), and dicumyl peroxide (0.600 mmol, 6 equiv, 162 mg) were weighed into a vial charged with a magnetic stirrer. 0.3 mL degassed MeCN was added, followed by isoamyl benzoate substrate **1a** (0.100 mmol, 1 equiv, 20 µL). The vial was sealed, and the headspace of the vial was flushed with a stream of N_2 for 1 min. The mixture was then stirred and irradiated with a blue LED for 4 hours with cooling fans maintaining the temperature at 25 °C. The reaction mixture was concentrated *in vacuo*, and filtered through a short silica plug with rinsing using EtOAc. Volatile materials were evaporated under reduced pressure, and an aliquot was removed for the preparation of LC-MS sample by dilution with MeCN. The results are shown in **Supplementary Figure 9**.

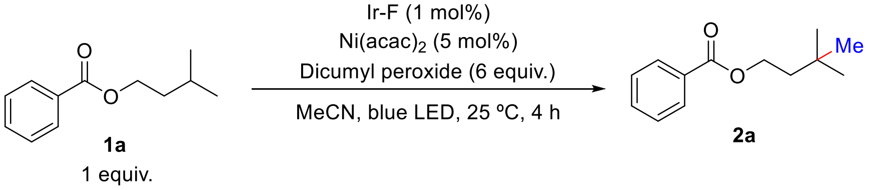

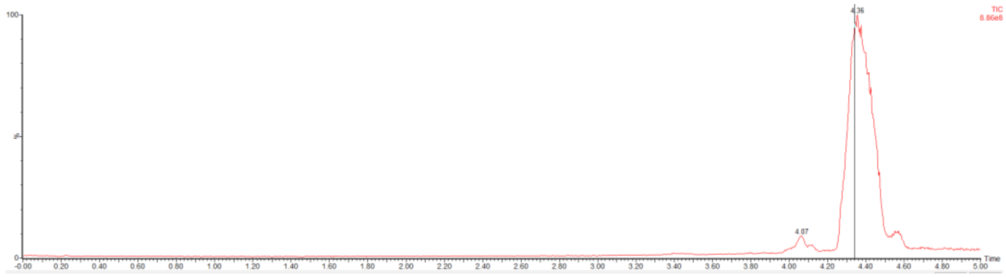

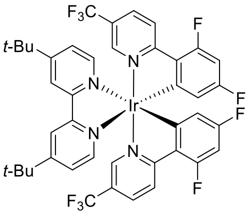

Chemical Formula: $C_{42}H_{34}F_{10}IrN_4$
Exact Mass: 977.2253
Molecular Weight: 976.9630
m/z: 977.2253 (100.0%), 975.2230 (59.5%), 978.2287 (45.4%), 976.2263 (27.0%), 979.2320 (10.1%), 977.2297 (6.0%), 978.2223 (1.5%), 980.2354 (1.5%)

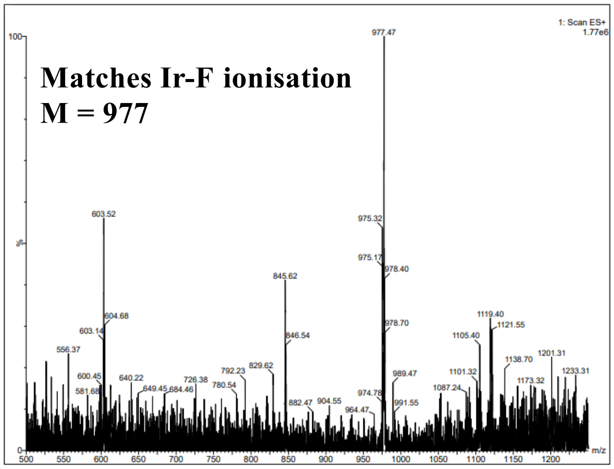

Supplementary Figure 9. Mass spectrum from the major UV-Vis peak containing Ir-F from LC-MS chromatogram of the methylation reaction after 4 hours.

16 hours, with Ni(acac)$_2$:

Ir-F (1.2 mg, 1 mol%, 1.0 µmol), Ni(acac)$_2$ (1.3 mg, 5 mol%, 5.0 µmol), and dicumyl peroxide (0.600 mmol, 6 equiv, 162 mg) were weighed into a vial charged with a magnetic stirrer. 0.3 mL MeCN was added, followed by isoamyl benzoate substrate **1a** (0.100 mmol, 1 equiv, 20 µL). The vial was sealed, and the headspace of the vial was flushed with a stream of N_2 for 1 min. The mixture was then stirred and irradiated with a blue LED for 16 hours with cooling fans maintaining the temperature at 25 °C. The reaction mixture was concentrated *in vacuo*, and filtered through a short silica plug with rinsing using EtOAc. Volatile materials were evaporated under reduced pressure, and an aliquot was removed for the preparation of LC-MS sample by dilution with MeCN. The results are shown in **Supplementary Figure 10**.

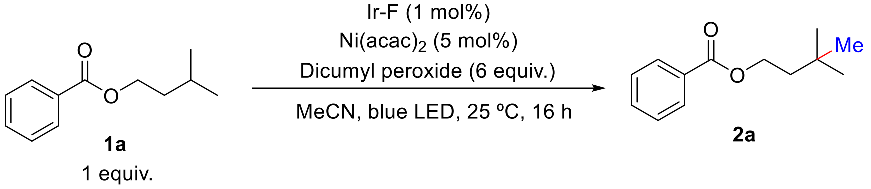

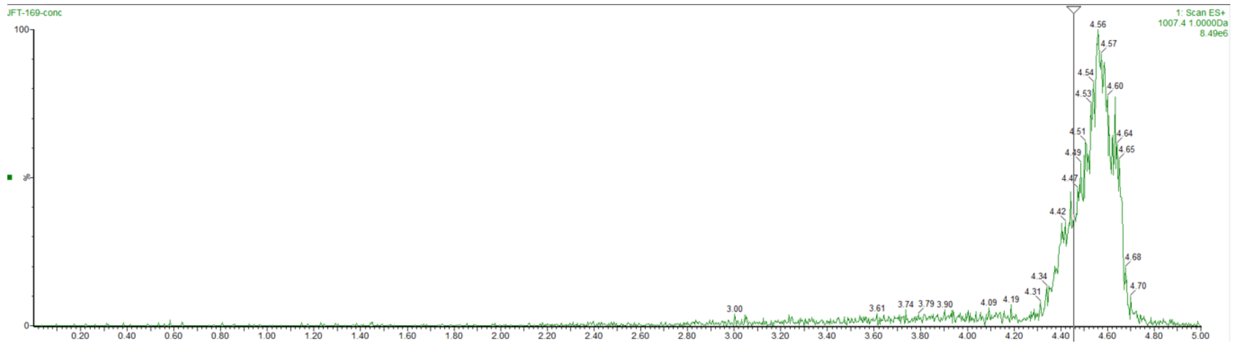

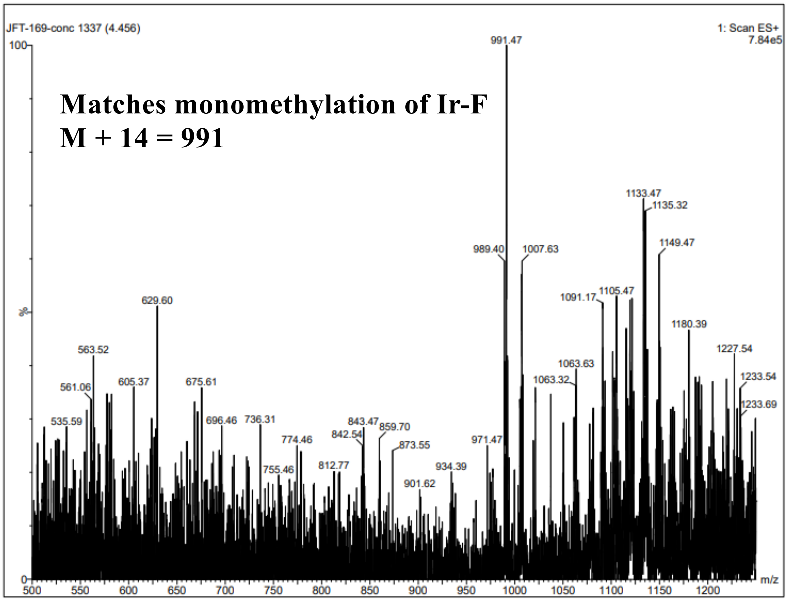

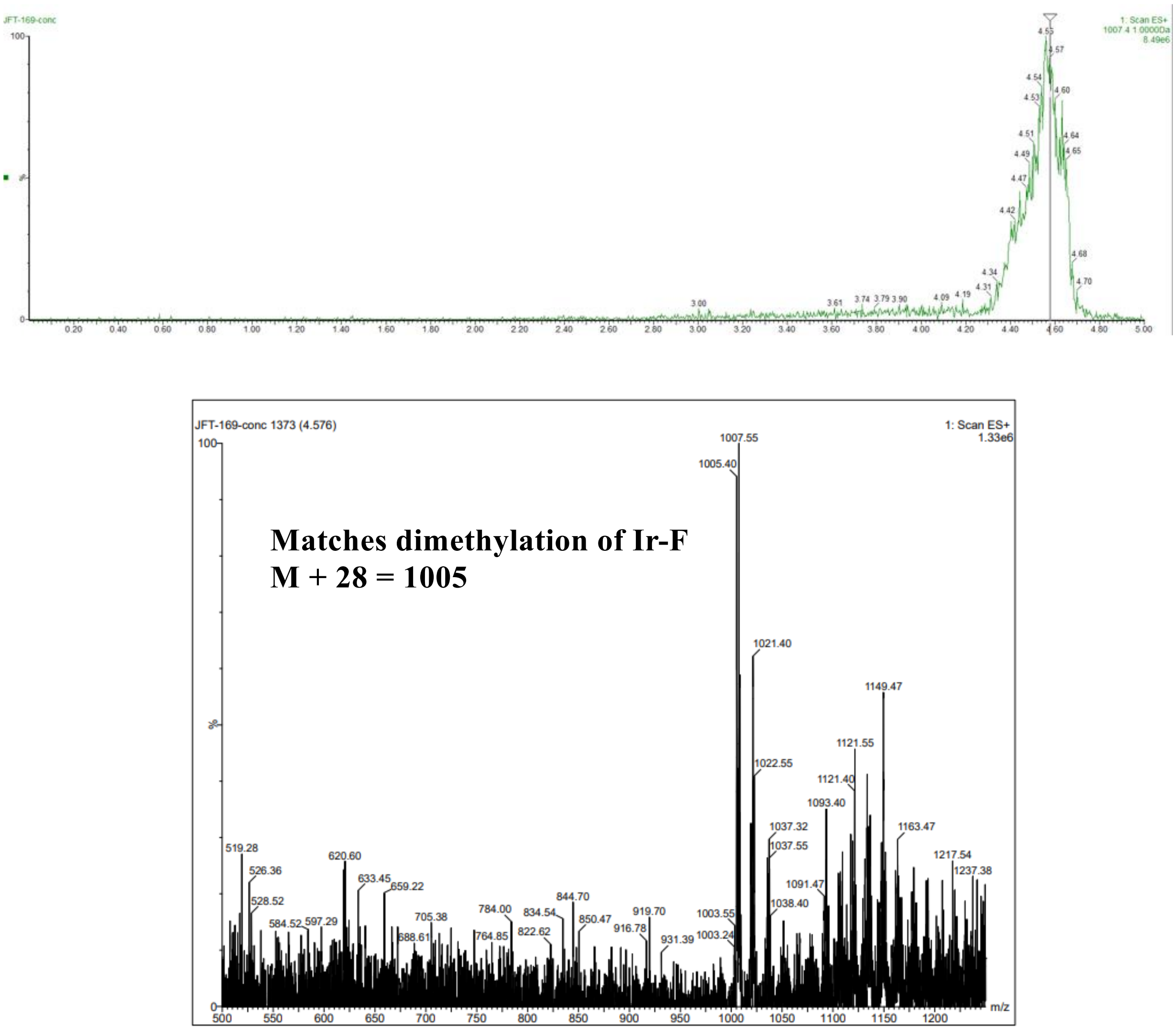

Supplementary Figure 10. Mass spectra from the major UV-Vis peak containing the derivatives of Ir-F from LC-MS chromatogram of the methylation reaction after 16 hours.

Discussion

In the presence of $Ni(acac)_2$, the parent m/z of Ir-F ([M] = 977) was detected after 4 hours of reaction time. After 16 hours of reaction time, Ir-F was found to undergo changes in its m/z, where m/z = [M+14] and [M+28] were detected as the major post-reaction species. These m/z correspond to the mono-methylated and di-methylated derivatives of Ir-F, possibly formed via the addition of methyl radicals to the aromatic ligands of Ir-F. The same set of m/z was observed when the reaction was run for 4 hours without $Ni(acac)_2$. Based on the drastic deactivation of Ir-F in the absence of $Ni(acac)_2$ (Section 6.2, entry 5), it is reasonable to postulate that this methylating process contributes to the deactivation of Ir-F.

6.4 Time-dependent Quantitative Changes of Peroxide, 1a, and 2a

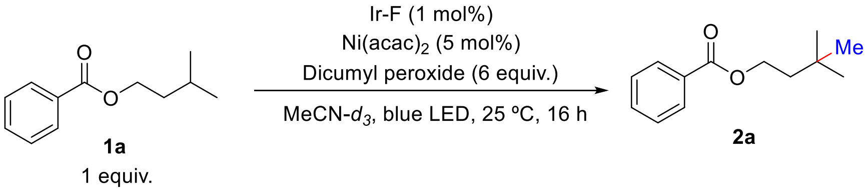

Ir-F (1.2 mg, 1 mol%, 1.0 µmol), Ni(acac)$_2$ (1.3 mg, 5 mol%, 5.0 µmol), and dicumyl peroxide (0.600 mmol, 6 equiv, 162 mg) were weighed into a vial charged with a magnetic stirrer. 0.3 mL degassed MeCN-*d*$_3$ was added, followed by isoamyl benzoate substrate **1a** (0.100 mmol, 1 equiv, 20 µL). The vial was sealed, and the headspace of the vial was flushed with a stream of N_2 for 1 min. The mixture was then stirred and irradiated with a blue LED with cooling fans maintaining the temperature at 25 °C. After 2 hours, and reaction mixture was concentrated *in vacuo* and filtered through a short silica plug, followed by rinsing the plug with CH_2Cl_2. A known amount of 1,3,5-trimethoxybenzene was added as an internal standard. ^{1}H NMR was performed in $CDCl_3$. The same procedure was repeated in duplicates of seven, and the reactions were stopped at designated time points (4 hours, 6 hours, 8 hours, 10 hours, 12 hours, 14 hours, and 16 hours). The absolute quantities of dicumyl peroxide, **1a**, and **2a** were obtained based on ^{1}H NMR spectroscopy in all duplicates. The results are shown in **Supplementary Figures 11** and **12**.

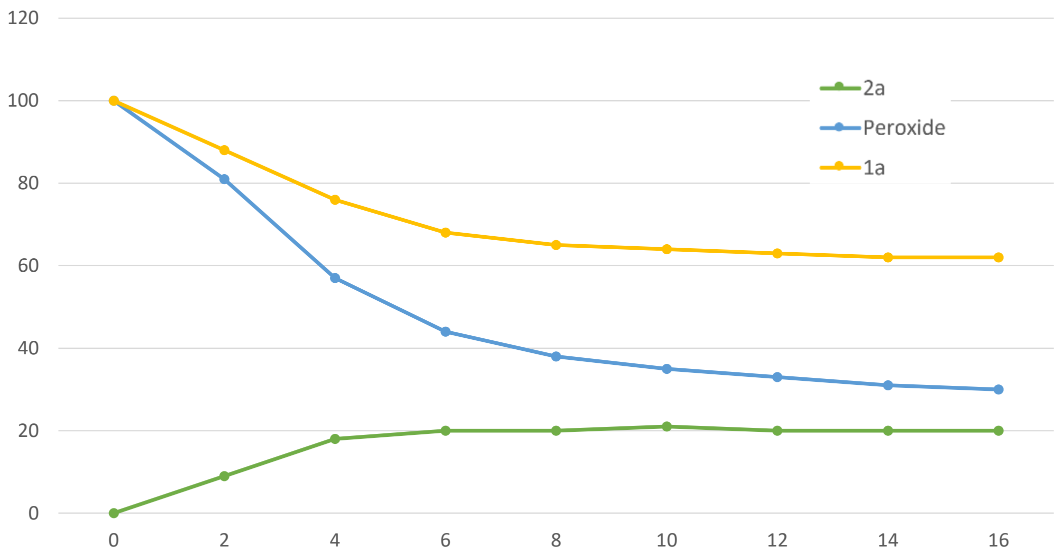

Supplementary Figure 11. Changes in peroxide, **1a**, and **2a** as a function of time.

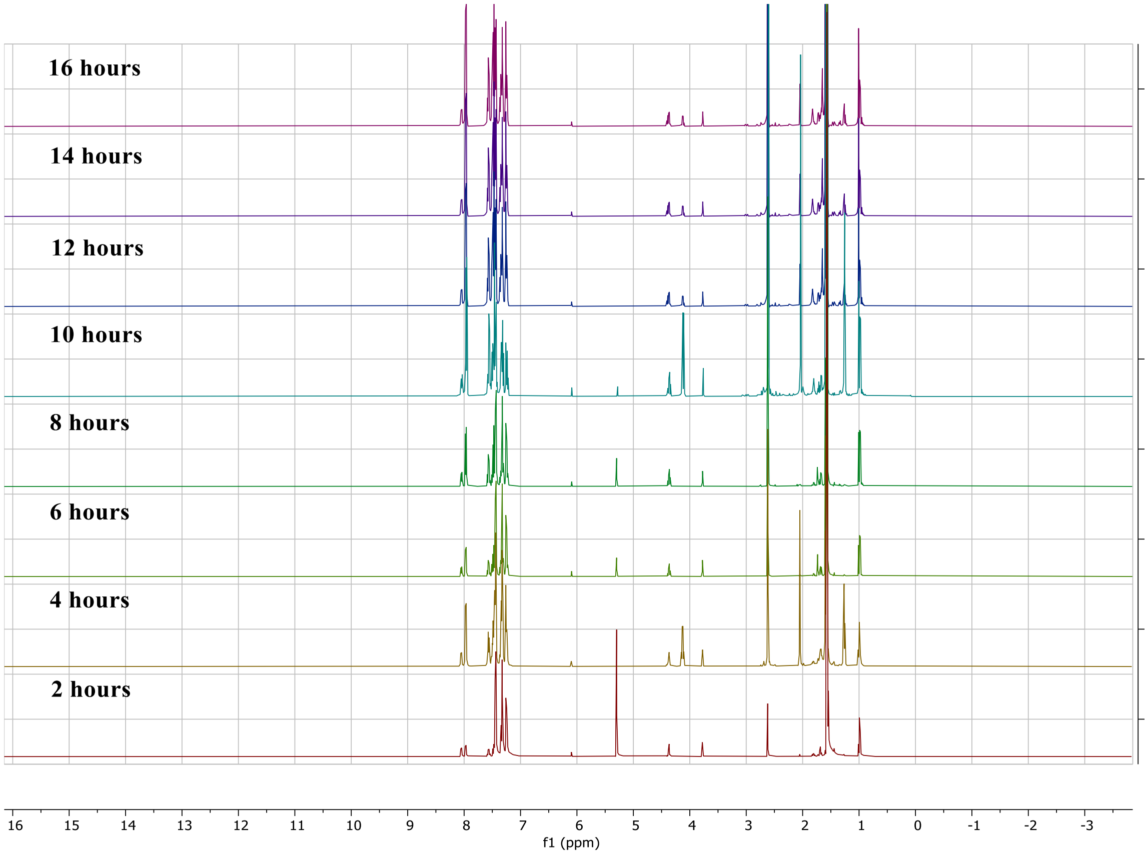
16 hours
14 hours
12 hours
10 hours
8 hours
6 hours
4 hours
2 hours
f1 (ppm)

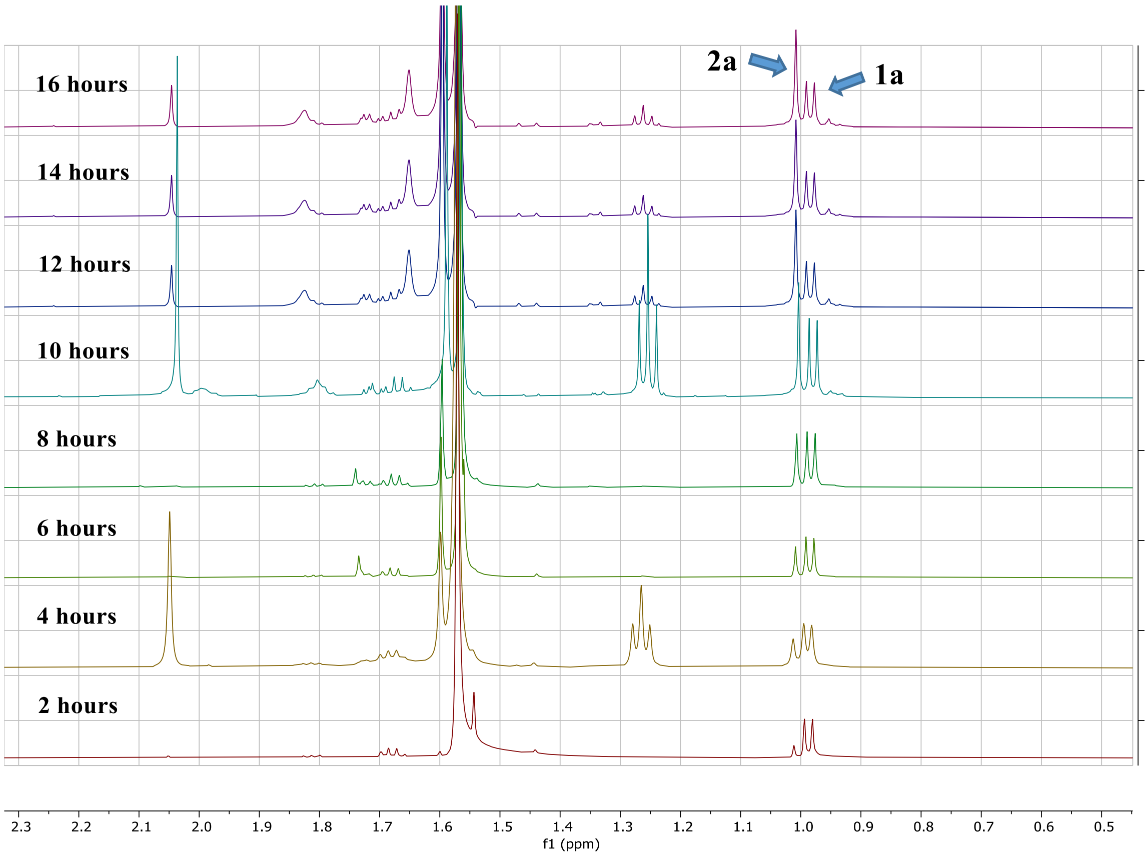
2a
1a
16 hours
14 hours
12 hours
10 hours
8 hours
6 hours
4 hours
2 hours
f1 (ppm)

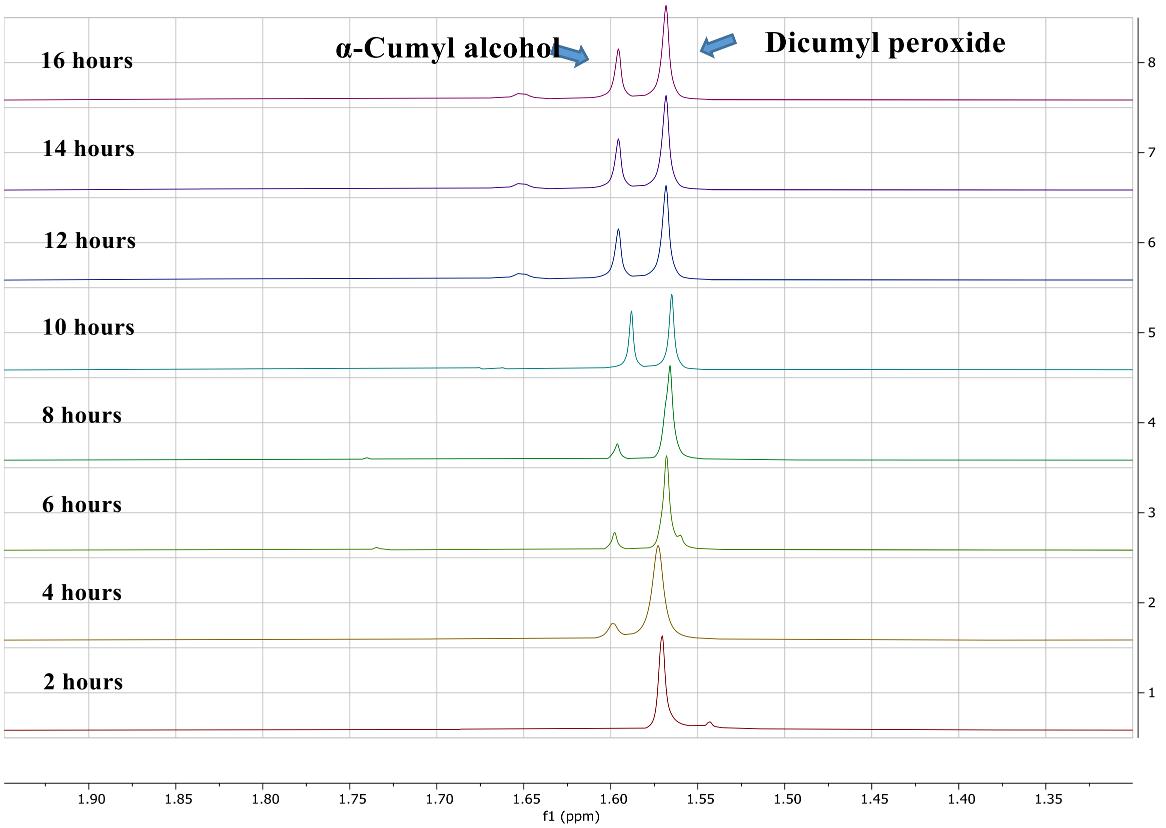

Supplementary Figure 12. Crude ^{1}H NMR spectra at different time points.

Discussion

The formation of **2a** reached a plateau shortly after 4 hours, while the consumption of **1a** and dicumyl peroxide continued after 4 hours. During this period, the amounts of acetophenone and cumyl alcohol also continued to increase, indicating the production of methyl radicals and HAT to alkoxy radicals. The termination of the C(sp^3)−Me coupling and a non-productive activation of peroxide during the 4−16 hour window suggest that Ni(acac)$_2$ becomes deactivated first, followed by more rapid deactivation of Ir-F when Ni(acac)$_2$ is no longer present to trap the methyl radicals.

6.5 The Formation of 3-Methyl Acetylacetonate under the Standard Condition

6.5.1 Reaction conducted in the presence of 2,4-pentanedione and $Ni(acac)_2$:

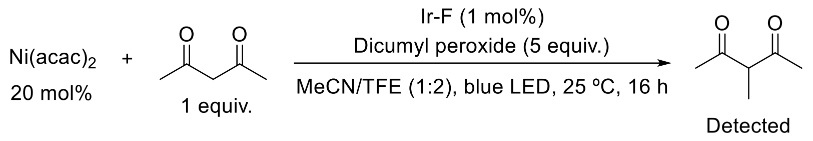

Ir-F (2.4 mg, 1 mol%, 2.0 μmol), $Ni(acac)_2$ (10.4 mg, 20 mol%, 40.0 μmol), and dicumyl peroxide (1.0 mmol, 5 equiv, 270 mg) were weighed into a vial charged with a magnetic stirrer. A pre-formed 1:2 mixture of degassed MeCN and TFE (0.3 mL) were added, followed by 2,4-pentanedione (0.200 mmol, 1 equiv, 20 μL). The vial was sealed, and the headspace of the vial was flushed with a stream of N_2 for 1 min. The mixture was then stirred and irradiated with a blue LED for 16 hours with cooling fans maintaining the temperature at 25 °C. The reaction mixture was concentrated *in vacuo* and filtered through a short silica plug, followed by rinsing the plug with CH_2Cl_2. After evaporation of the volatile materials, the sample was diluted in MeCN and analyzed by LC-MS, and ^1H NMR spectroscopy was performed in $CDCl_3$. The results are shown in **Supplementary Figures 13** and **14**. The analytical samples were recovered, combined, and concentrated. The crude residue was purified by preparative thin-layer chromatography with EtOAc and hexane (2:3), to afford 3-methyl-2,4-pentanedione as a colourless oil (3.4 mg, 15% yield).

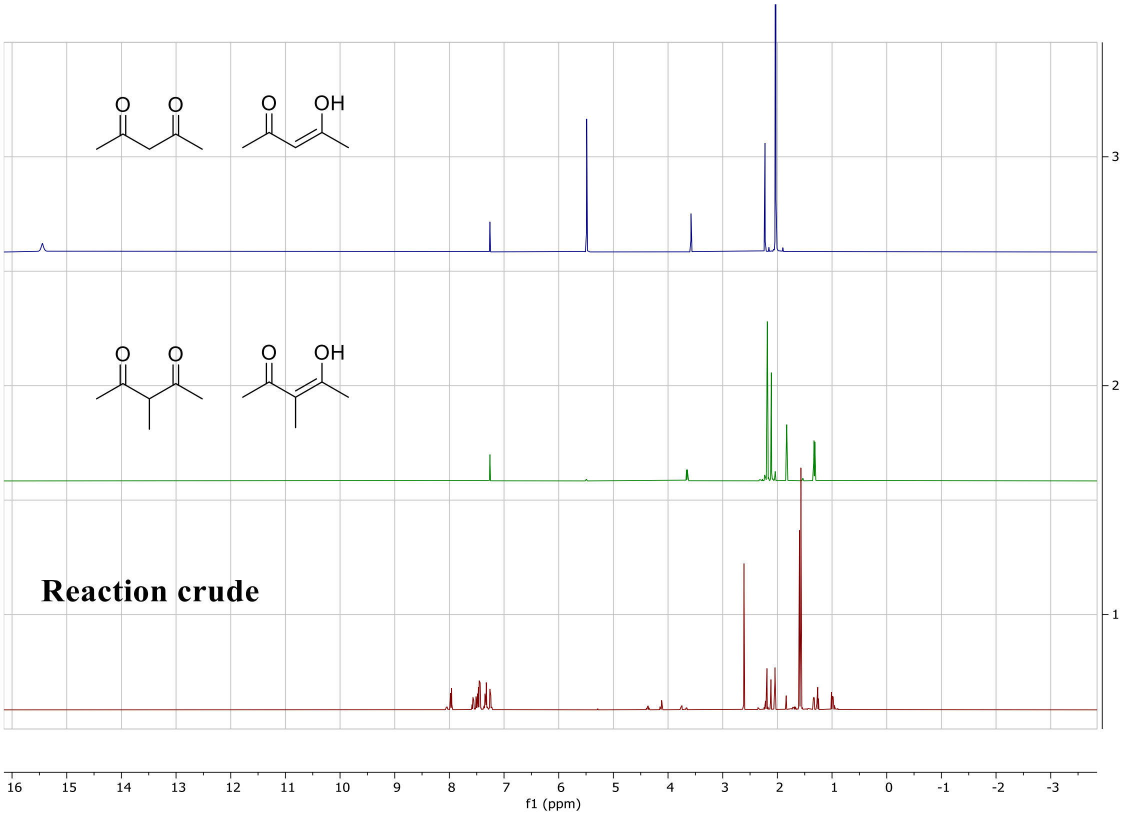

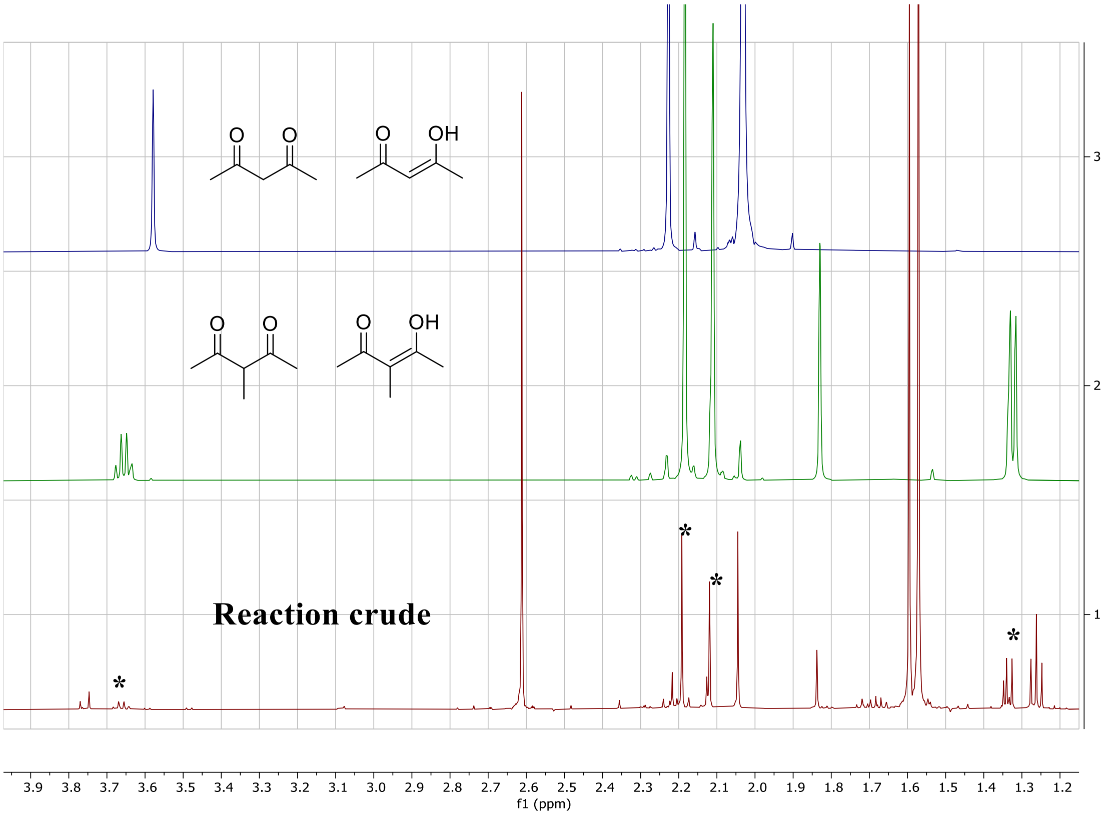

Supplementary Figure 13. Crude ^{1}H NMR spectrum of the reaction carried out at Section 6.5.1.

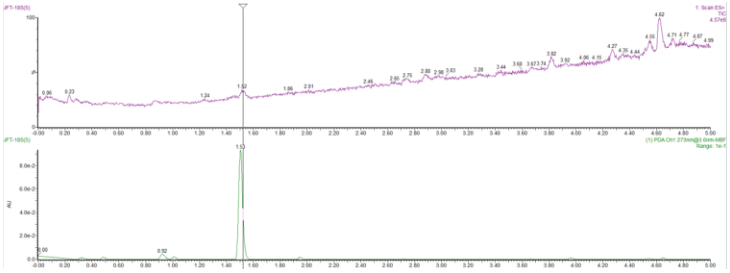

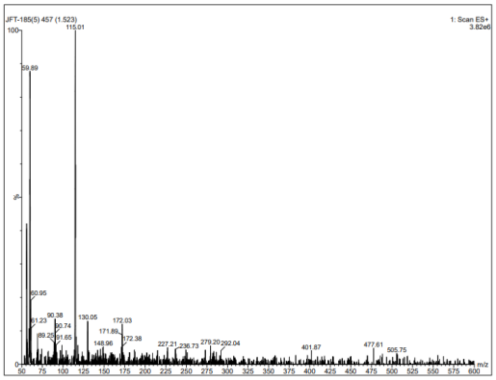

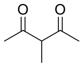

Chemical Formula: $C_6H_{10}O_2$
Exact Mass: 114.0681
Molecular Weight: 114.1440
m/z: 114.0681 (100.0%), 115.0714 (6.5%)

Supplementary Figure 14. LC-MS chromatogram and mass spectrum of the reaction carried out in Section 6.5.1.

6.5.2 Reaction conducted in the presence of 2,4-pentanedione only:

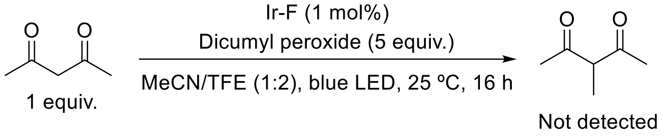

Ir-F (2.4 mg, 1 mol%, 2.0 μmol) and dicumyl peroxide (1.0 mmol, 5 equiv, 270 mg) were weighed into a vial charged with a magnetic stirrer. A pre-formed 1:2 mixture of degassed MeCN and TFE (0.3 mL) were added, followed by 2,4-pentanedione (0.200 mmol, 1 equiv, 20 μL). The vial was sealed, and the headspace of the vial was flushed with a stream of N_2 for 1 min. The mixture was then stirred and irradiated with a blue LED for 16 hours with cooling fans maintaining the temperature at 25 °C. The reaction mixture was concentrated *in vacuo* and filtered through a short silica plug, followed by rinsing the plug with CH_2Cl_2. After evaporation of the volatile materials, the sample was diluted in MeCN and analyzed by LC-MS, and ^{1}H NMR spectroscopy was performed in $CDCl_3$.

Observation

In the absence of Ni(acac)$_2$, 3-methyl-2,4-pentanedione was not detected.

6.5.3 Reaction conducted in the presence of Ni(acac)$_2$ only:

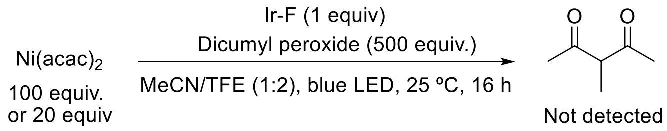

Ir-F (2.4 mg, 1 equiv, 2.0 μmol), Ni(acac)$_2$ (52.0 mg, 100 equiv, 0.200 mol), and dicumyl peroxide (1.0 mmol, 500 equiv, 270 mg) were weighed into a vial charged with a magnetic stirrer. A pre-formed 1:2 mixture of degassed MeCN and TFE (0.3 mL) were added. The vial was sealed, and the headspace of the vial was flushed with a stream of N_2 for 1 min. The mixture was then stirred and irradiated with a blue LED for 16 hours with cooling fans maintaining the temperature at 25 °C. The reaction mixture was concentrated *in vacuo* and filtered through a short silica plug, followed by rinsing the plug with CH_2Cl_2. After evaporation of the volatile materials, the sample was diluted in MeCN and analyzed by LC-MS, and ^{1}H NMR spectroscopy was performed in $CDCl_3$.

The same procedure was repeated with 20 equiv Ni(acac)$_2$ (10.4 mg, 40.0 μmol).

Observation

In the absence of 2,4-pentanedione, 3-methyl-2,4-pentanedione was not detected.

Discussion

The detection of 3-methyl-2,4-pentanedione in the combined presence of Ni(acac)$_2$ and 2,4-pentanedione (Section 6.5.1) indicates a reaction between methyl radicals and the ligands of Ni(acac)$_2$, which would form 3-methyl-2,4-pentanedione. This methylation of the ligand is analogous to the mechanism reported in the Cu-catalyzed methylation of 1,3-dicarbonyl derivatives.[13,14] The presence of 2,4-pentanedione was required for detectable quantities of 3-methyl-2,4-pentanedione. The reason for the lack of detection of 3-methyl-2,4-pentanedione in the sole presence of Ni(acac)$_2$ without added 2,4-pentanedione in Section 6.5.3 is unclear.

6.6 Investigation of Sequential Additions of $Ni(acac)_2$

Ir-F (1.2 mg, 1 mol%, 1.0 µmol), $Ni(acac)_2$ (1.3 mg, 5 mol%, 5.0 µmol), and dicumyl peroxide (0.600 mmol, 6 equiv, 162 mg) were weighed into a vial charged with a magnetic stirrer. 0.3 mL degassed MeCN was added, followed by isoamyl benzoate substrate **1a** (0.100 mmol, 1 equiv, 20 µL). The vial was sealed, and the headspace of the vial was flushed with a stream of N_2 for 1 min. The mixture was then stirred and irradiated with a blue LED with cooling fans maintaining the temperature at 25 °C. After 4 hours, an additional batch of $Ni(acac)_2$ (2.6 mg, 10 mol%, 10.0 µmol) was added to the mixture, followed by stirring and irradiation with a blue LED for another 16 hours. The reaction mixture was concentrated *in vacuo* and filtered through a short silica plug, followed by rinsing the plug with CH_2Cl_2. Volatile materials were evaporated under reduced pressure. 1,3,5-trimethoxybenzene was added as an internal standard. Crude ^1H NMR spectroscopy was performed in $CDCl_3$.

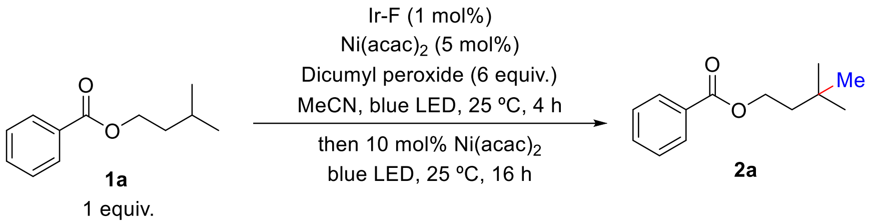

Entry	Deviation	Yield 2a (%)
**1**	-	25
**2**	No additional $Ni(acac)_2$ after 4 hours (total irradiation time 20 hours)	20

Supplementary Table 15. Investigation of sequential addition of $Ni(acac)_2$.

6.7 Evaluation of the Conditions in Reference 10

Condition 1: Ir-F (2.4 mg, 1.0 mol%, 2.0 µmol), $NiCl_2$•diglyme (1.7 mg, 4 mol%, 8.0 µmol), 4,4',4''-^{t}Bu-tpy (3.2 mg, 4 mol%, 8.0 µmol), $B(OH)_3$ (6.2 mg, 0.5 equiv, 0.100 mmol), and dicumyl peroxide (1.200 mmol, 6 equiv, 324 mg) were weighed into a vial charged with a magnetic stirrer. Degassed MeCN (0.2 mL) was added, followed by substrate **1a**, **1s**, **1v** or **1ab** (0.200 mmol, 1 equiv). The vial was sealed, and the headspace of the vial was flushed with a stream of N_2 for 1 min. The mixture was then stirred and irradiated under a blue LED Kessil lamp for 16 hours with cooling fans maintaining the temperature at 25 °C. The reaction mixture was then concentrated *in vacuo*, and filtered through a short silica plug, followed by rinsing the plug with CH_2Cl_2. Volatile materials were evaporated under reduced pressure, and a known amount of 1,3,5-trimethoxybenzene was added as an internal standard. Crude ^{1}H NMR spectroscopy was performed in $CDCl_3$.

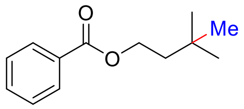

2a
1%

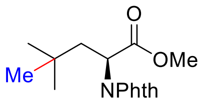

2s
2%

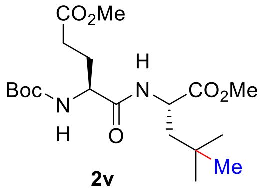

2v
2%

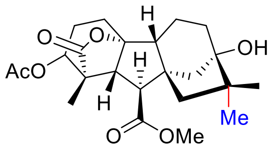

2ab
0%

Condition 2: Ir-F (2.4 mg, 1.0 mol%, 2.0 µmol), $NiCl_2$•diglyme (1.7 mg, 4 mol%, 8.0 µmol), 4,4',4''-^{t}Bu-tpy (3.2 mg, 4 mol%, 8.0 µmol) were weighed into a vial charged with a magnetic stirrer. Degassed TFE (0.2 mL) was added, followed by substrate **1a**, **1s**, **1u**, **1v** or **1ab** (0.200 mmol, 1 equiv).and di-tert-butyl peroxide (1.200 mmol, 6 equiv, 219 µL). The vial was sealed, and the headspace of the vial was flushed with a stream of N_2 for 1 min. The mixture was then stirred and irradiated under a blue LED Kessil lamp for 16 hours with cooling fans maintaining the temperature at 25 °C. The reaction mixture was then concentrated *in vacuo* and filtered through a short silica plug, followed by rinsing the plug with CH_2Cl_2. Volatile materials were evaporated under reduced pressure, and a known amount of 1,3,5-trimethoxybenzene was added as an internal standard. Crude ^{1}H NMR spectroscopy was performed in $CDCl_3$.

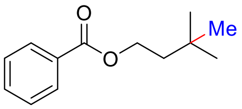

2a
8%

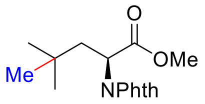

2s
9%

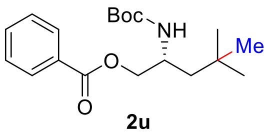

2u
18%
0.3 mmol scale: 22%

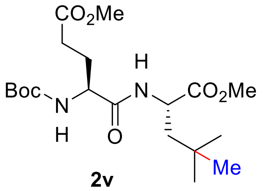

2v
4%

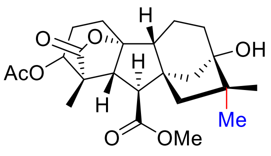

2ab
0%

Condition 3: Ir-F (2.4 mg, 1.0 mol%, 2.0 μmol), $NiCl_2$•diglyme (1.7 mg, 4 mol%, 8.0 μmol), tris(2-pyridylmethyl)amine (2.3 mg, 4 mol%, 8.0 μmol), $MeB(OH)_2$ (6.0 mg, 0.5 equiv, 0.100 mmol), and dicumyl peroxide (1.200 mmol, 6 equiv, 324 mg) were weighed into a vial charged with a magnetic stirrer. Degassed MeCN (0.2 mL) was added, followed by **1a**, **1s**, **1v** or **1ab** (0.200 mmol, 1 equiv). The vial was sealed, and the headspace of the vial was flushed with a stream of N_2 for 1 min. The mixture was then stirred and irradiated under a blue LED Kessil lamp for 16 hours with cooling fans maintaining the temperature at 25 °C. The reaction mixture was then concentrated *in vacuo*, and filtered through a short silica plug, followed by rinsing the plug with CH_2Cl_2. Volatile materials were evaporated under reduced pressure, and a known amount of 1,3,5-trimethoxybenzene was added as an internal standard. Crude ^{1}H NMR spectroscopy was performed in $CDCl_3$.

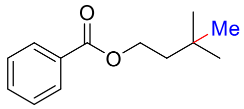

2a
4%

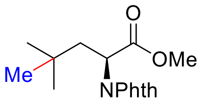

2s
5%

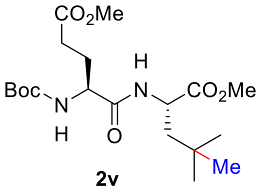

2v
3%

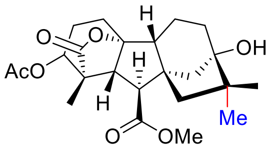

2ab
0%

6.8 Evaluation of the selectivity of the C–H methylation

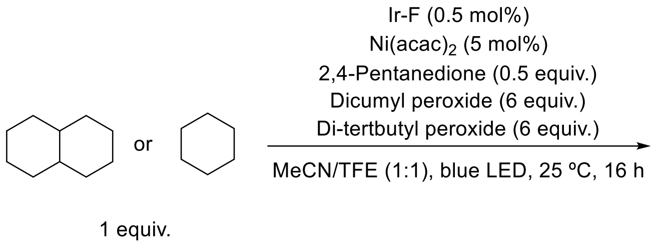

Conditions:

Ir-F (1.2 mg, 0.5 mol%, 1.0 µmol), Ni(acac)$_2$ (2.6 mg, 5 mol%, 10.0 µmol), and dicumyl peroxide (0.800 mmol, 4 equiv, 216 mg) were weighed into a vial charged with a magnetic stirrer. A pre-formed 1:1 mixture of MeCN and TFE (0.2 mL) were added, followed by the substrate (*cis*-decahydronaphthalene or hexane, 0.200 mmol, 1 equiv), di-tert-butyl peroxide (0.800 mmol, 4 equiv, 146 µL), and 2,4-pentanedione (10.0 µmol, 0.5 equiv, 10 µL). The vial was sealed, and the headspace of the vial was flushed with a stream of N_2 for 1 min. The mixture was then stirred and irradiated under a blue LED Kessil lamp for 16 hours with cooling fans maintaining the temperature at 25 °C. The reaction mixture was then concentrated *in vacuo*, filtered through a short silica plug, and flushed with diethyl ether (~8 mL).

In the case of the reaction with *cis*- decahydronaphthalene, volatile materials were evaporated under reduced pressure, and 1,3,5-trimethoxybenzene was added as an internal standard. The crude product was analyzed by quantitative ^{1}H NMR spectroscopy in $CDCl_3$.

In the case of the reaction with cyclohexane, the solution obtained after filtration through the silica plug was made up to 10.00 mL in a volumetric flask. Subsequently, a 62.5 µL aliquot was taken and diluted to 250.0 µL with diethyl ether. The resultant solution (5.00 mM) was subjected to analysis by GC-FID. The yield of methylcyclohexane was quantified by reference to an authentic standard.

The yield of methylcyclohexane was 0.8%.

The yield of *cis*-9-methyldecalin (lit. methyl peak at 0.96 ppm in $CDCl_3$) was <5%, while the yield of *trans*-9-methyldecalin (lit. methyl peak at 0.82 ppm in $CDCl_3$) was 27%.[16] The yields of 1-methylated or 2-methylated decalins were <5%.

Discussion:

These results demonstrate that the site selectivity of hydrogen atom abstraction is not solely governed by the substrate's intrinsic electronic properties. The system of Li and coworkers is more reactive toward secondary C–H bonds.[17] They reported a 37% yield for the methylation of cyclohexane, while we observed only trace yield with cyclohexane.

In addition, they reported that decalin underwent methylation in 12% yield at the secondary C–H bonds (3:2 ratio of 1-methyl to 2-methyl-*trans*-decalin, no tertiary C–H methylation observed). In contrast, our protocol gave a 27% yield of *trans*-9-methyldecalin and <5% yield of 1-methyl and 2-methyl-*trans*-decalin. Thus, the methylation of decalin catalyzed by GaN is selective for secondary C–H bonds while our reaction is highly selective for tertiary C–H bonds. While no

selectivity can be observed in the methylation of cyclohexane, the low reactivity of that substrate under our reaction conditions also is consistent with a higher rate of functionalization of tertiary C–H bonds than secondary C–H bonds.

The formation of *trans*-9-methyldecalin starting from *cis*-decalin suggests that hydrogen atom abstraction of the tertiary C(sp^3)–H bonds in *cis*-decalin occurs more rapidly than methylation of the tertiary radical by the nickel catalyst. We hypothesize that epimerization occurs by the tertiary radical abstracting a hydrogen atom from solvent.

6.9 Evaluation of the rate of consumption of dicumyl peroxide

Condition 1 (iridium photocatalyst only):

Dicumyl peroxide (0.600 mmol, 1 equiv, 162 mg) was weighed into an oven-dried vial equipped with a magnetic stir bar. A stock solution of Ir-F in acetonitrile (0.300 mL of a 3.33 mM solution, 0.167 mol%, 1.0 μmol) was added. The vial was sealed, and the headspace of the vial was flushed with a stream of N_2 for 1 min. The mixture was then stirred and irradiated with 400 nm LEDs with cooling fans maintaining the temperature at 25 °C. The experiments were performed using a photoreactor (Penn PhD Photoreactor M2) for reproducibility After the given reaction time, the reaction mixture was diluted with dichloromethane and filtered through a short silica plug (~2 mL of silica), rinsing with dichloromethane. A known amount of 1,3,5-trimethoxybenzene was added as an internal standard. ^{1}H NMR was performed in $CDCl_3$. The absolute quantities of dicumyl peroxide and acetophenone were determined by quantitative ^{1}H NMR spectroscopy.

Condition 2 (iridium photocatalyst and nickel catalyst):

$Ni(acac)_2$ (1.3 mg, 0.833 mol%, 5.0 μmol) and dicumyl peroxide (0.600 mmol, 1 equiv, 162 mg) were weighed into an oven-dried vial equipped with a magnetic stir bar. A stock solution of Ir-F in acetonitrile (0.300 mL of a 3.33 mM solution, 0.167 mol%, 1.0 μmol) was added. The vial was sealed, and the headspace of the vial was flushed with a stream of N_2 for 1 min. The mixture was then stirred and irradiated with 400 nm LEDs with cooling fans maintaining the temperature at 25 °C. The experiments were performed using a photoreactor (Penn PhD Photoreactor M2) for reproducibility. After the given reaction time, the reaction mixture was diluted with dichloromethane and filtered through a short silica plug (~2 mL of silica), rinsing with dichloromethane. A known amount of 1,3,5-trimethoxybenzene was added as an internal standard. ^{1}H NMR was performed in $CDCl_3$. The absolute quantities of dicumyl peroxide and acetophenone were determined by quantitative ^{1}H NMR spectroscopy.

Condition 3 (nickel catalyst only):

$Ni(acac)_2$ (1.3 mg, 0.833 mol%, 5.0 μmol) and dicumyl peroxide (0.600 mmol, 1 equiv, 162 mg) were weighed into an oven-dried vial equipped with a magnetic stir bar. Acetonitrile (0.300 mL) was added. The vial was sealed, and the headspace of the vial was flushed with a stream of N_2 for 1 min. The mixture was then stirred and irradiated with 400 nm LEDs with cooling fans maintaining the temperature at 25 °C. The experiments were performed using a photoreactor (Penn PhD Photoreactor M2) for reproducibility. After the given reaction time, the reaction mixture was diluted with dichloromethane and filtered through a short silica plug (~2 mL of silica), rinsing with dichloromethane. A known amount of 1,3,5-trimethoxybenzene was added as an internal standard. ^{1}H NMR was performed in $CDCl_3$. The absolute quantities of dicumyl peroxide and acetophenone were determined by quantitative ^{1}H NMR spectroscopy.

Condition 4 (control, no iridium or nickel catalysts):

Dicumyl peroxide (0.600 mmol, 1 equiv, 162 mg) was weighed into an oven-dried vial equipped with a magnetic stir bar. Acetonitrile (0.300 mL) was added. The vial was sealed, and the headspace of the vial was flushed with a stream of N_2 for 1 min. The mixture was then stirred and

irradiated with 400 nm LEDs with cooling fans maintaining the temperature at 25 °C. The experiments were performed using a photoreactor (Penn PhD Photoreactor M2) for reproducibility. After the given reaction time, the reaction mixture was diluted with dichloromethane and filtered through a short silica plug (~2 mL of silica), rinsing with dichloromethane. A known amount of 1,3,5-trimethoxybenzene was added as an internal standard. ^{1}H NMR was performed in $CDCl_3$. The absolute quantities of dicumyl peroxide and acetophenone were determined by quantitative ^{1}H NMR spectroscopy.

The plots below show the rate of conversion of dicumyl peroxide and the rate of formation of acetophenone in the presence of both catalysts (Condition 2) and in the presence of the iridium photocatalyst alone (Condition 1).

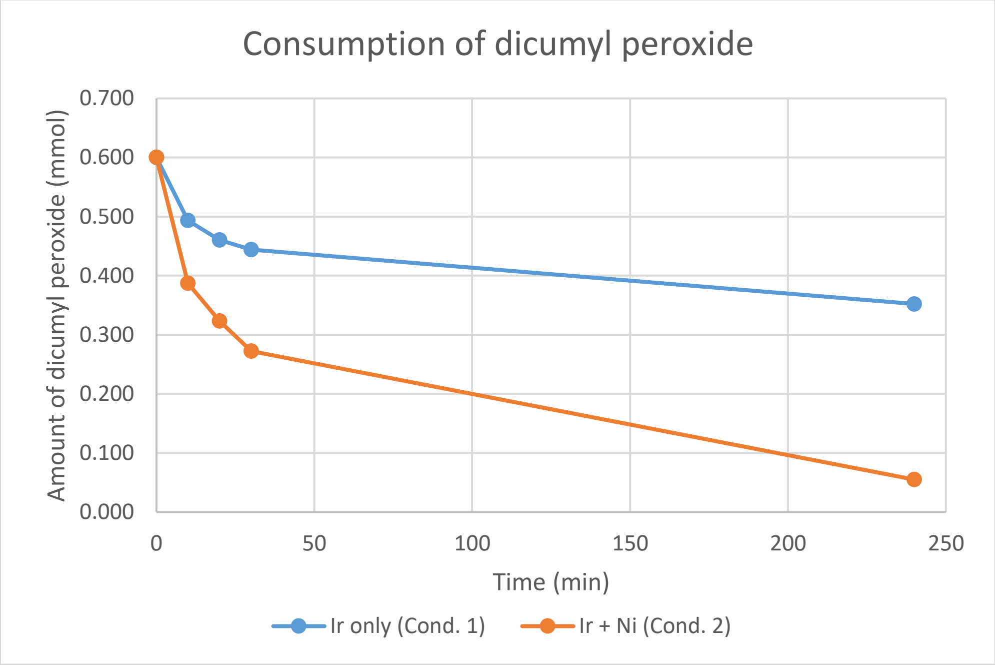

Supplementary Figure 15. Consumption of dicumyl peroxide with time under Conditions 1 and 2.

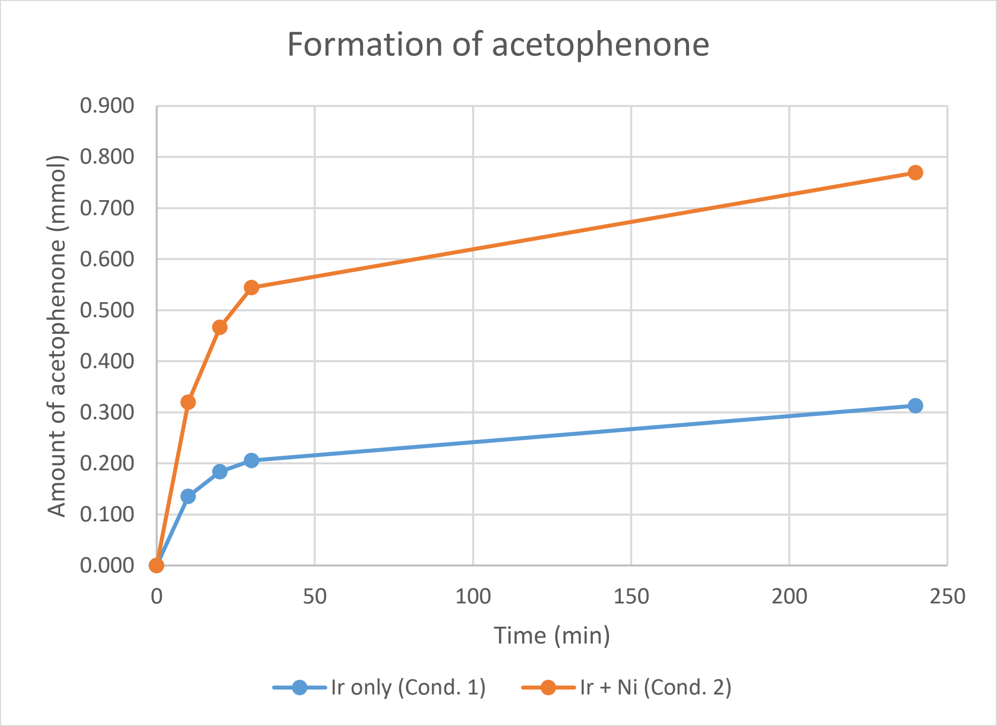

Supplementary Figure 16. Formation of acetophenone with time under Conditions 1 and 2.

Condition	Catalysts	DCP remaining (mmol)	Acetophenone (mmol)
Initial	-	0.600	0.000
1	Ir	0.352	0.313
2	Ir and Ni	0.055	0.769
3	Ni	0.332	0.420
4	None	0.349	0.384

Supplementary Table 16. Conversion of dicumyl peroxide (DCP) and the formation of acetophenone after 4 hours under Conditions 1-4.

Discussion:

The plots show that the rate of consumption of dicumyl peroxide and the rate of formation of acetophenone are significantly higher in the presence of both catalysts (Condition 2) than in the presence of the iridium photocatalyst alone (Condition 1). This is consistent with our hypothesis that $Ni(acac)_2$ plays an important role in prolonging the lifetime of the iridium photocatalyst.

Based on the results in the table, the background homolysis of dicumyl peroxide under irradiation by 400 nm LEDs is significant (Conditions 4), as 42% of the peroxide is consumed and a 32% yield of acetophenone is observed (via β-scission). We suspect that the LEDs used in the Penn PhD Photoreactor have a significant amount of emission in the ultraviolet wavelengths.

The conversion of dicumyl peroxide and formation of acetophenone in the presence of the iridium photocatalyst alone (Condition 1) or the nickel catalyst alone (Condition 3) are comparable to those

of the background reaction (Condition 4). In contrast, when both catalysts are present (Condition 2), the consumption of dicumyl peroxide and formation of acetophenone are both greatly increased.

These results are consistent with our mechanistic investigations and support catalyst cooperativity. Neither catalyst alone is competent at promoting the homolysis of dicumyl peroxide, but the combination of the two catalysts leads to increased consumption of the peroxide and formation of acetophenone above the background reaction. The iridium photocatalyst is capable of promoting homolysis of dicumyl peroxide by energy transfer, but it is rapidly deactivated by the methyl radicals generated by β-scission in the absence of the nickel catalyst. The nickel catalyst prolongs the lifetime of the iridium catalyst by capturing methyl radicals.

7. NMR Spectra

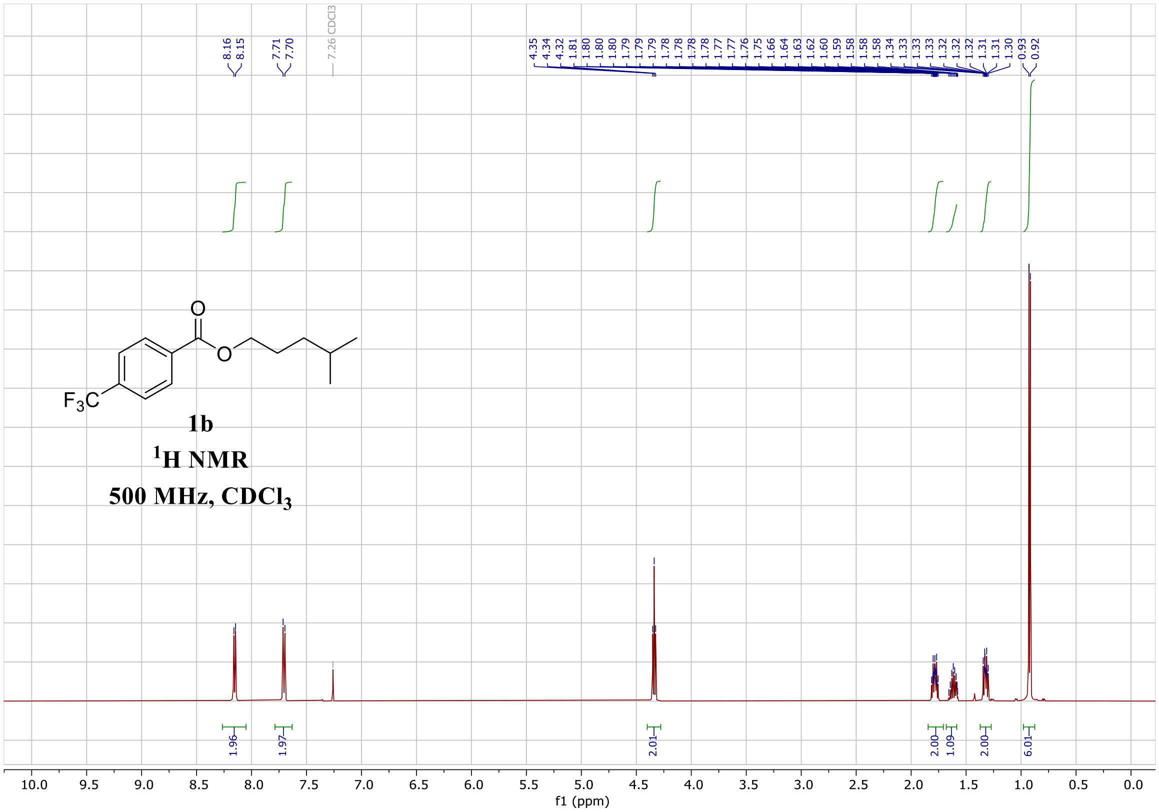

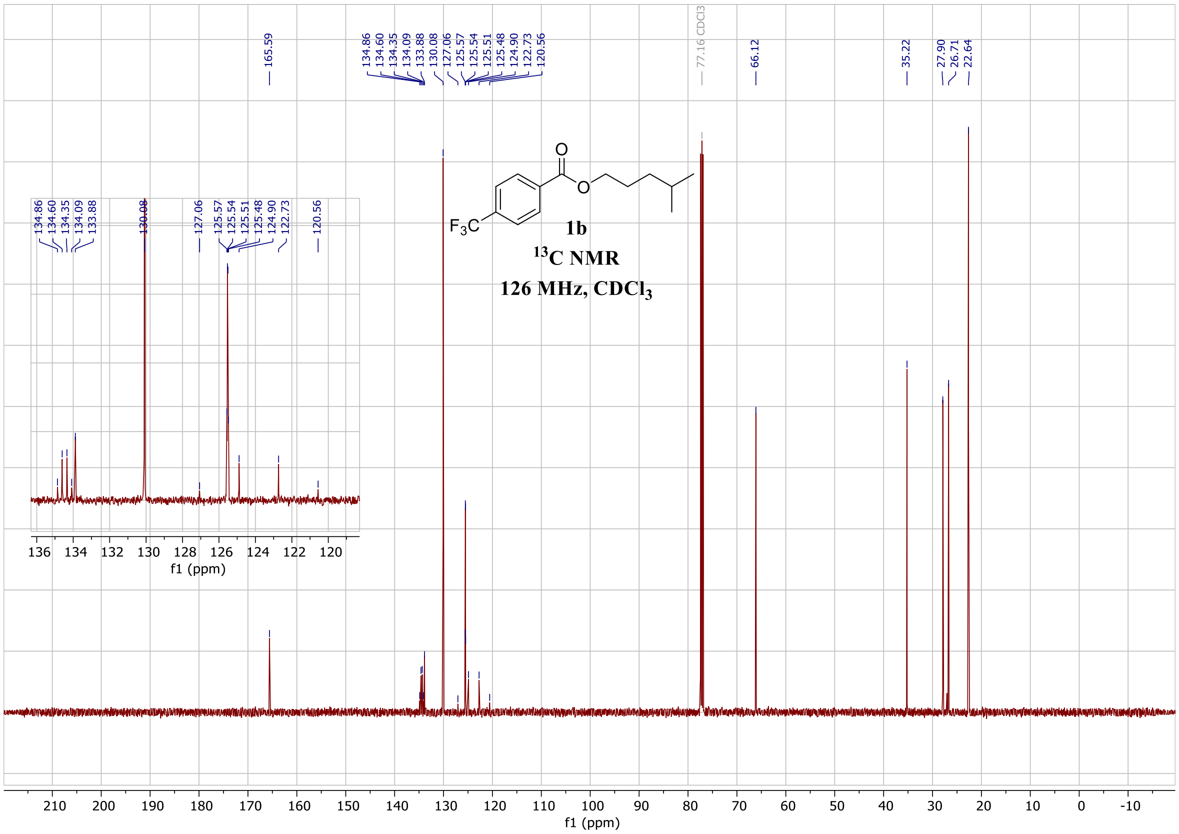

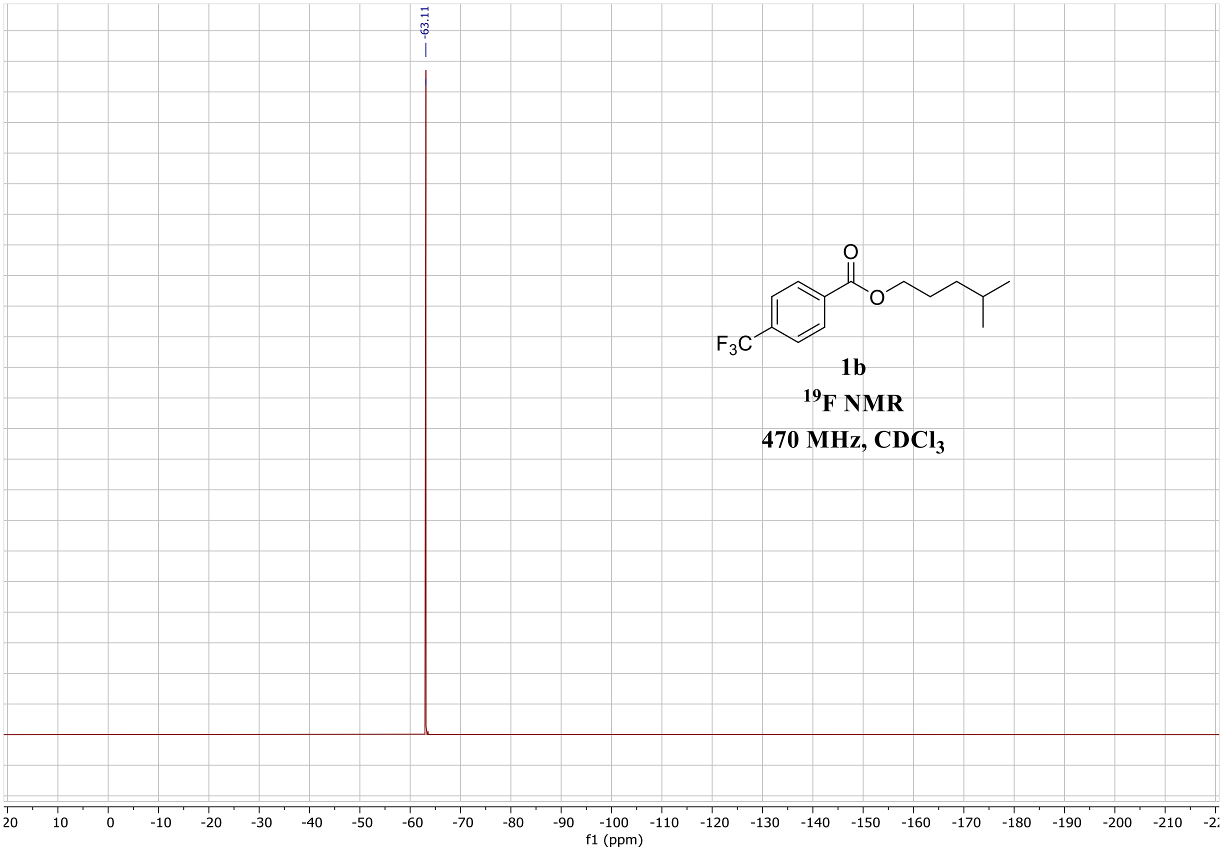

-63.11
F3C
O
O
1b
19F NMR
470 MHz, CDCl3
20 10 0 -10 -20 -30 -40 -50 -60 -70 -80 -90 -100 -110 -120 -130 -140 -150 -160 -170 -180 -190 -200 -210
f1 (ppm)

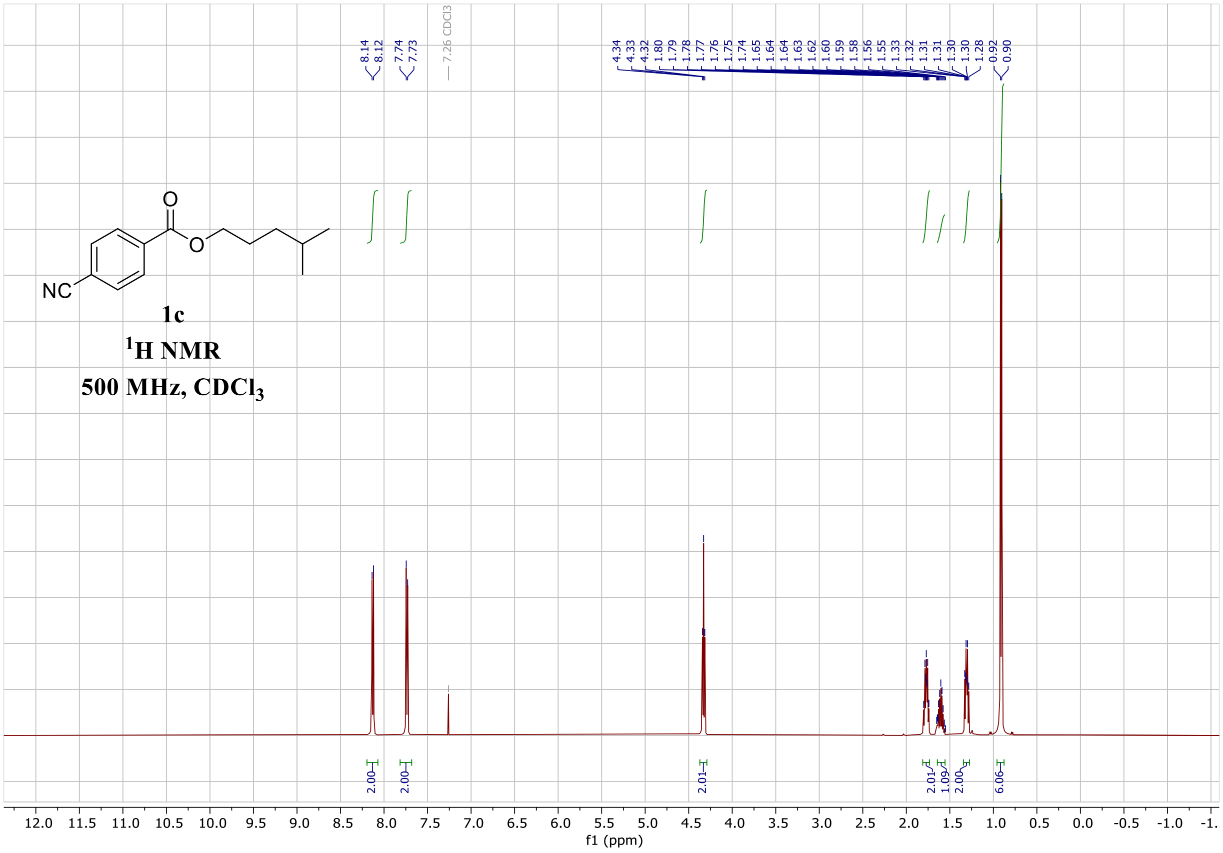

8.14
8.12
7.74
7.73
7.26 CDCl3
4.34
4.33
4.32
1.80
1.79
1.78
1.77
1.76
1.75
1.74
1.65
1.64
1.63
1.62
1.60
1.59
1.58
1.56
1.55
1.33
1.32
1.31
1.31
1.30
1.28
0.92
0.90
NC
O
O
1c
1H NMR
500 MHz, CDCl3
2.00
2.00
2.01
2.01
1.09
2.00
6.06
12.0 11.5 11.0 10.5 10.0 9.5 9.0 8.5 8.0 7.5 7.0 6.5 6.0 5.5 5.0 4.5 4.0 3.5 3.0 2.5 2.0 1.5 1.0 0.5 0.0 -0.5 -1.0
f1 (ppm)

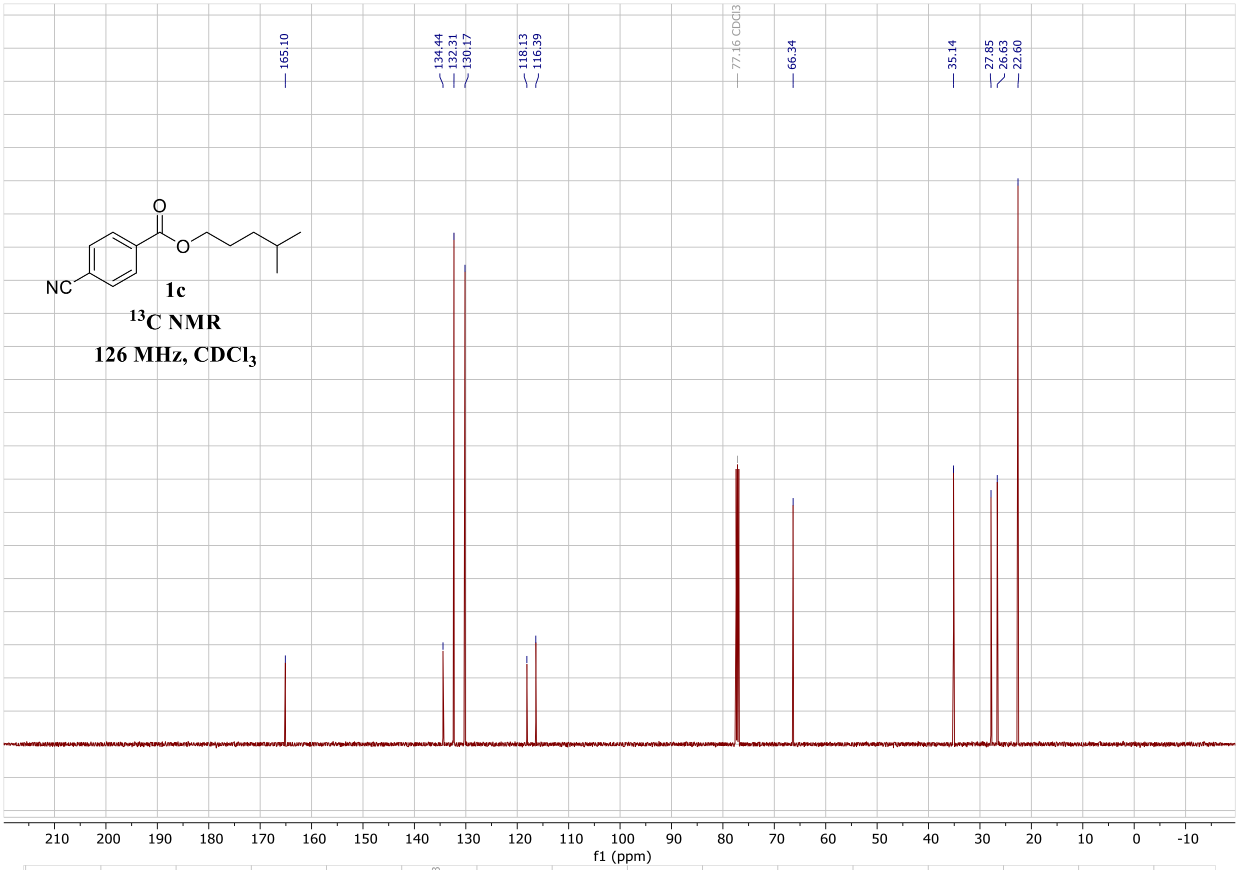
NC
O
O
1c
^{13}C NMR
126 MHz, $CDCl_3$
165.10
134.44
132.31
130.17
118.13
116.39
77.16 CDCl3
66.34
35.14
27.85
26.63
22.60
f1 (ppm)

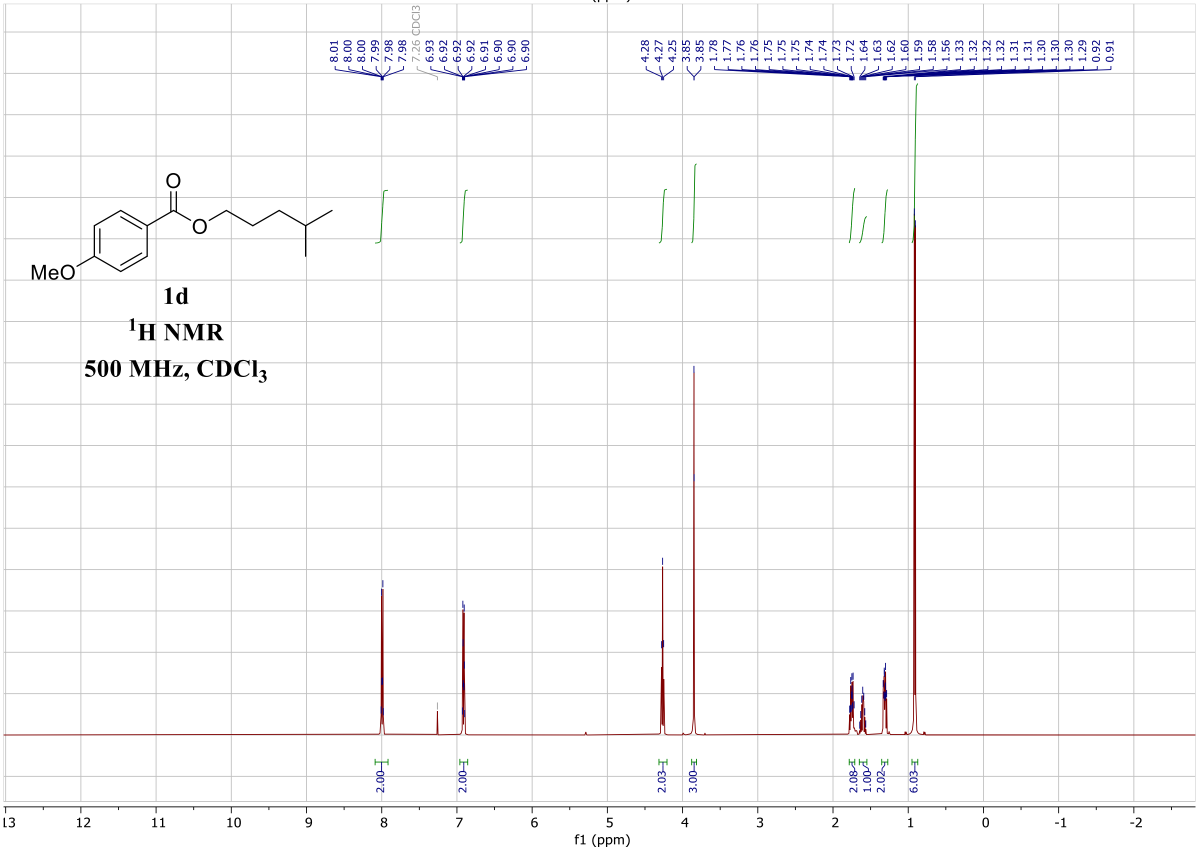
MeO
O
O
1d
^{1}H NMR
500 MHz, $CDCl_3$
7.26 CDCl3
2.00
2.00
2.03
3.00
2.08
1.00
2.02
6.03
f1 (ppm)

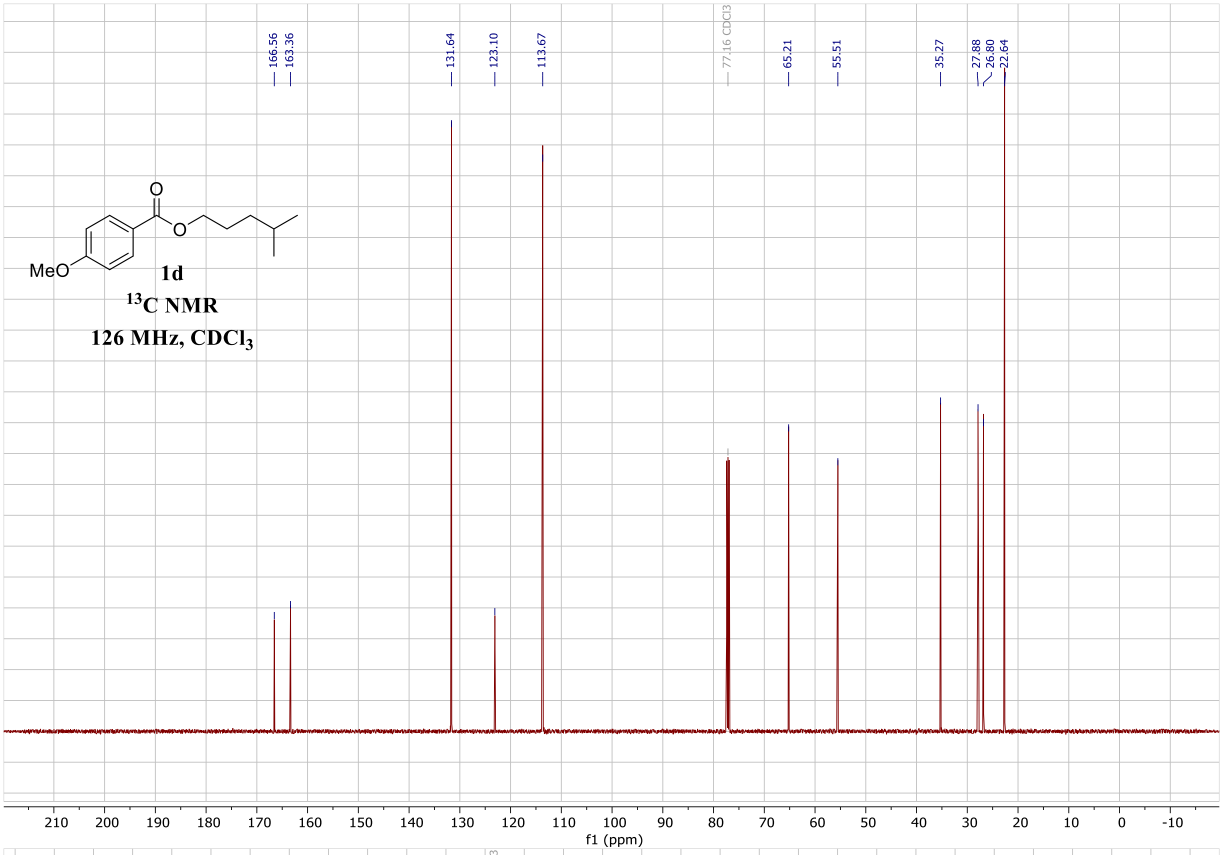
166.56
163.36
131.64
123.10
113.67
77.16 CDCl3
65.21
55.51
35.27
27.88
26.80
22.64
MeO
O
1d
13C NMR
126 MHz, CDCl3
f1 (ppm)

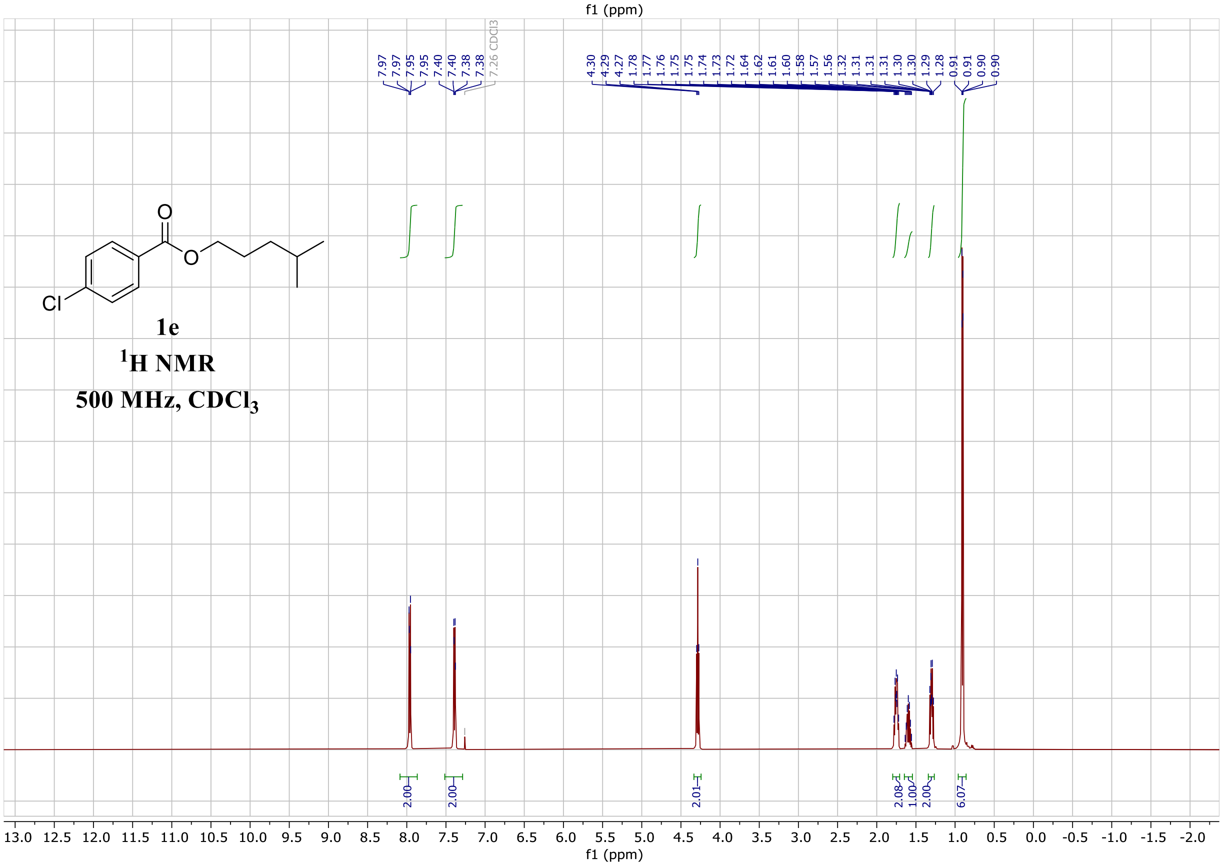
7.97
7.97
7.95
7.95
7.40
7.40
7.38
7.38
7.26 CDCl3
4.30
4.29
4.27
1.78
1.77
1.76
1.75
1.75
1.74
1.73
1.72
1.64
1.62
1.61
1.60
1.58
1.57
1.56
1.32
1.31
1.31
1.31
1.30
1.30
1.29
1.28
0.91
0.91
0.90
0.90
Cl
O
1e
1H NMR
500 MHz, CDCl3
2.00
2.00
2.01
2.08
1.00
2.00
6.07
f1 (ppm)

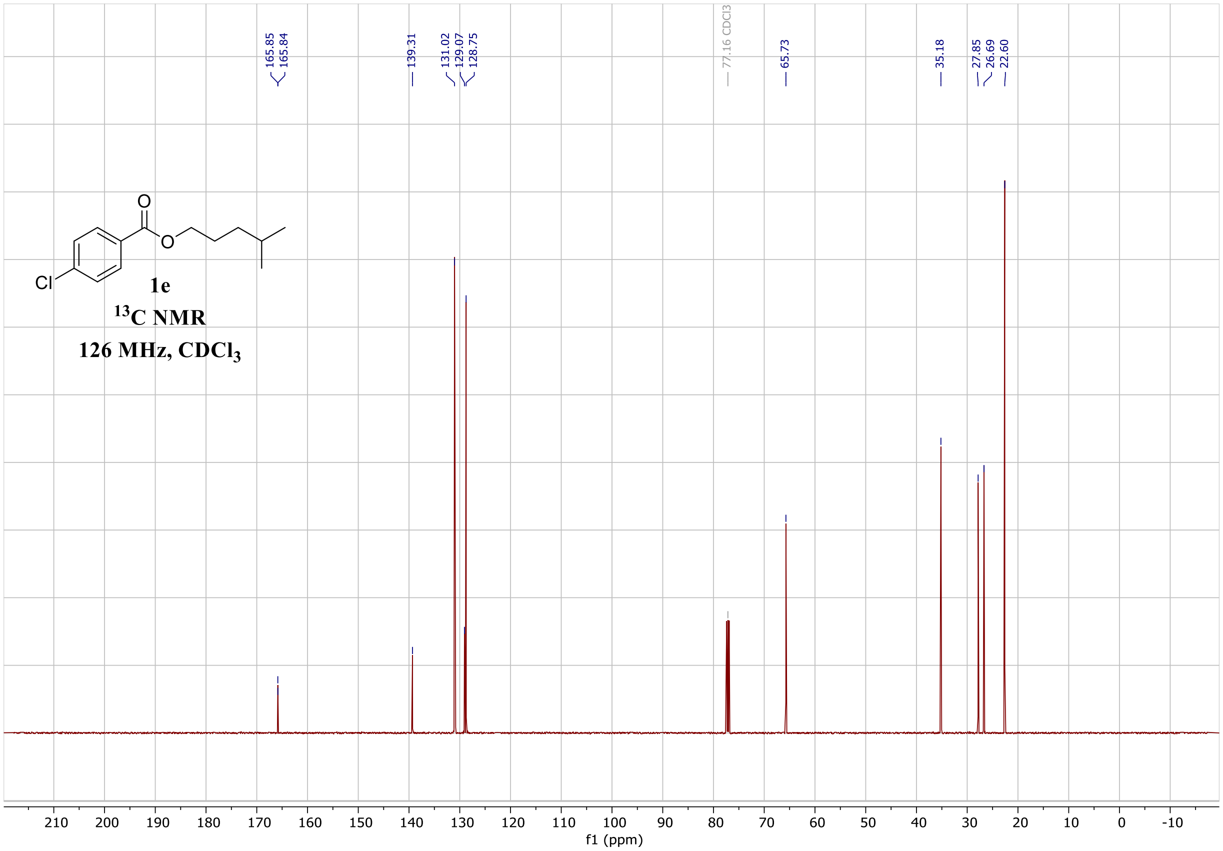
165.85
165.84
139.31
131.02
129.07
128.75
77.16 CDCl3
65.73
35.18
27.85
26.69
22.60
O
O
Cl
1e
13C NMR
126 MHz, CDCl3
f1 (ppm)

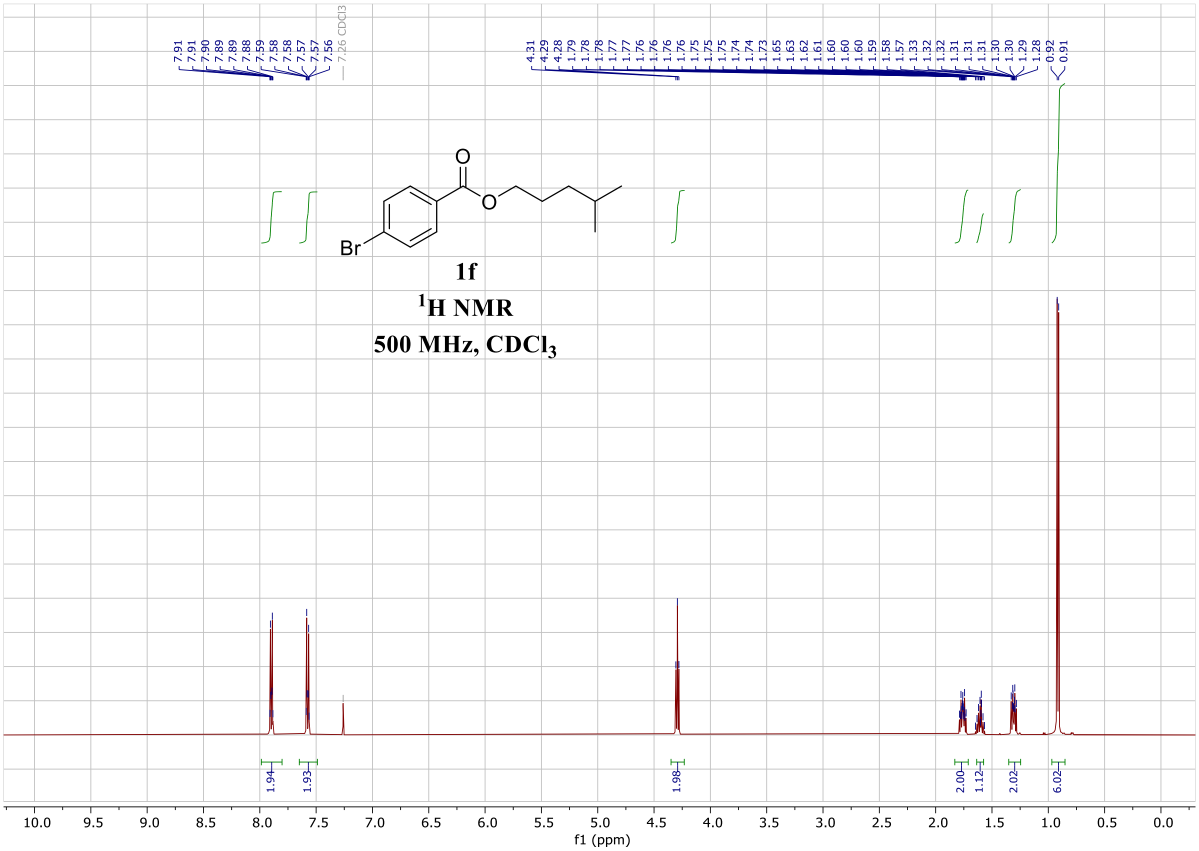
7.26 CDCl3
O
O
Br
1f
1H NMR
500 MHz, CDCl3
1.94
1.93
1.98
2.00
1.12
2.02
6.02
f1 (ppm)

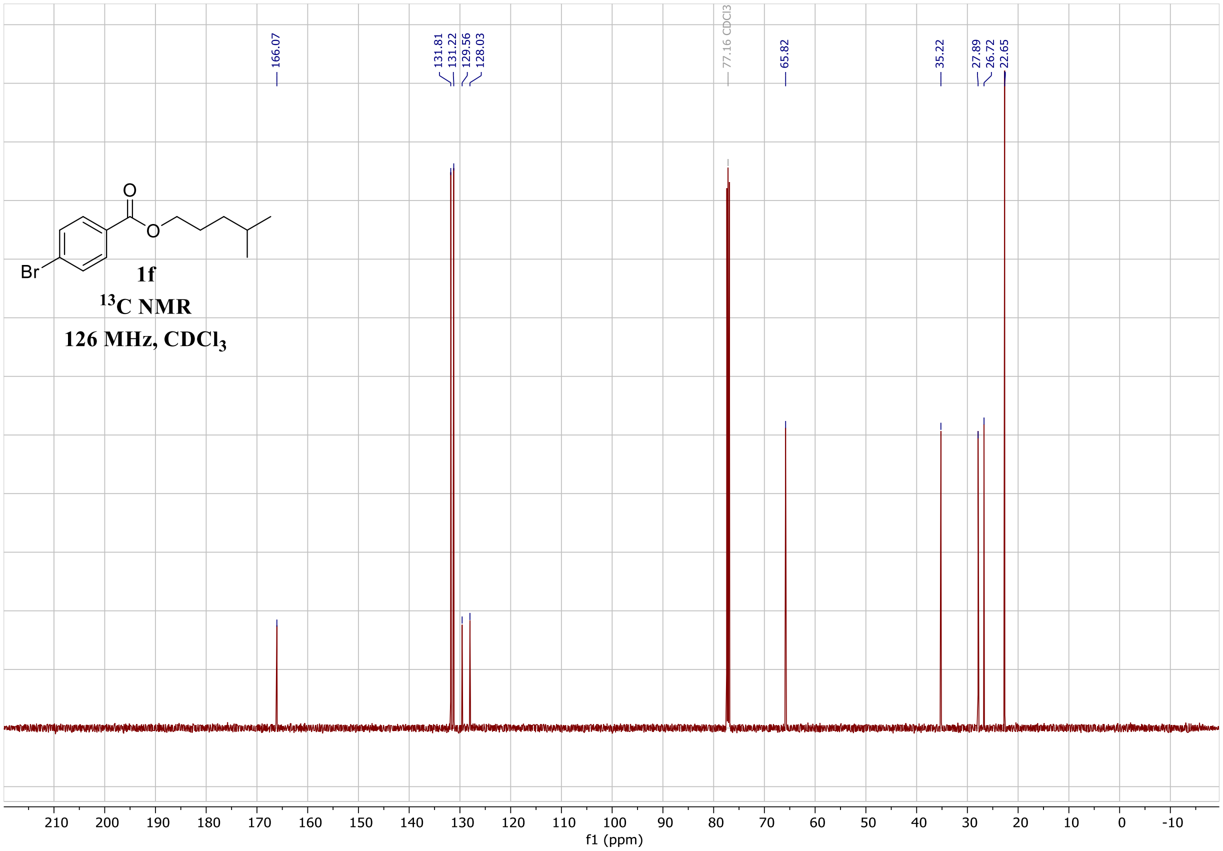
1f
13C NMR
126 MHz, CDCl3
166.07
131.81
131.22
129.56
128.03
77.16 CDCl3
65.82
35.22
27.89
26.72
22.65
f1 (ppm)

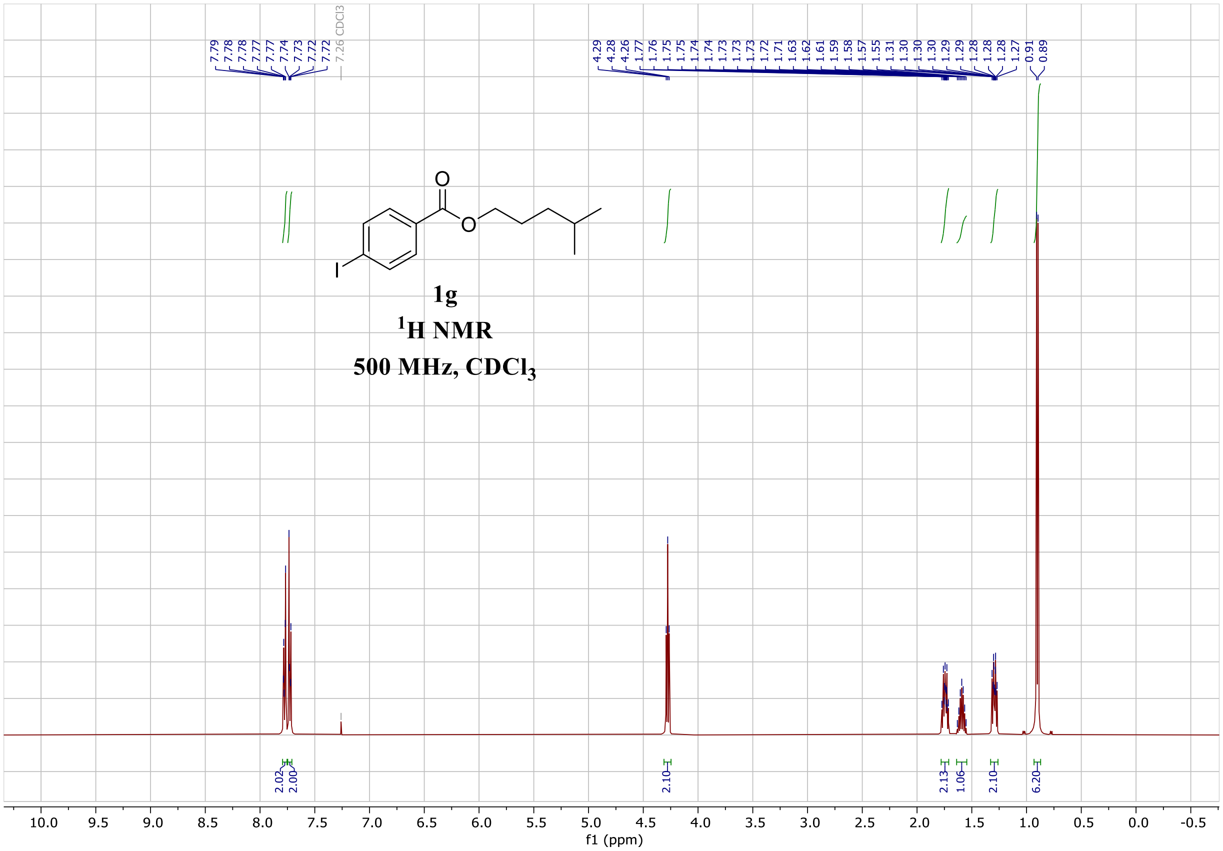
1g
1H NMR
500 MHz, CDCl3
7.79
7.78
7.78
7.77
7.77
7.74
7.73
7.72
7.72
7.26 CDCl3
4.29
4.28
4.26
1.77
1.76
1.75
1.75
1.74
1.74
1.73
1.73
1.73
1.72
1.71
1.63
1.62
1.61
1.59
1.58
1.57
1.55
1.31
1.30
1.30
1.29
1.29
1.28
1.28
1.27
0.91
0.89
2.02
2.00
2.10
2.13
1.06
2.10
6.20
f1 (ppm)

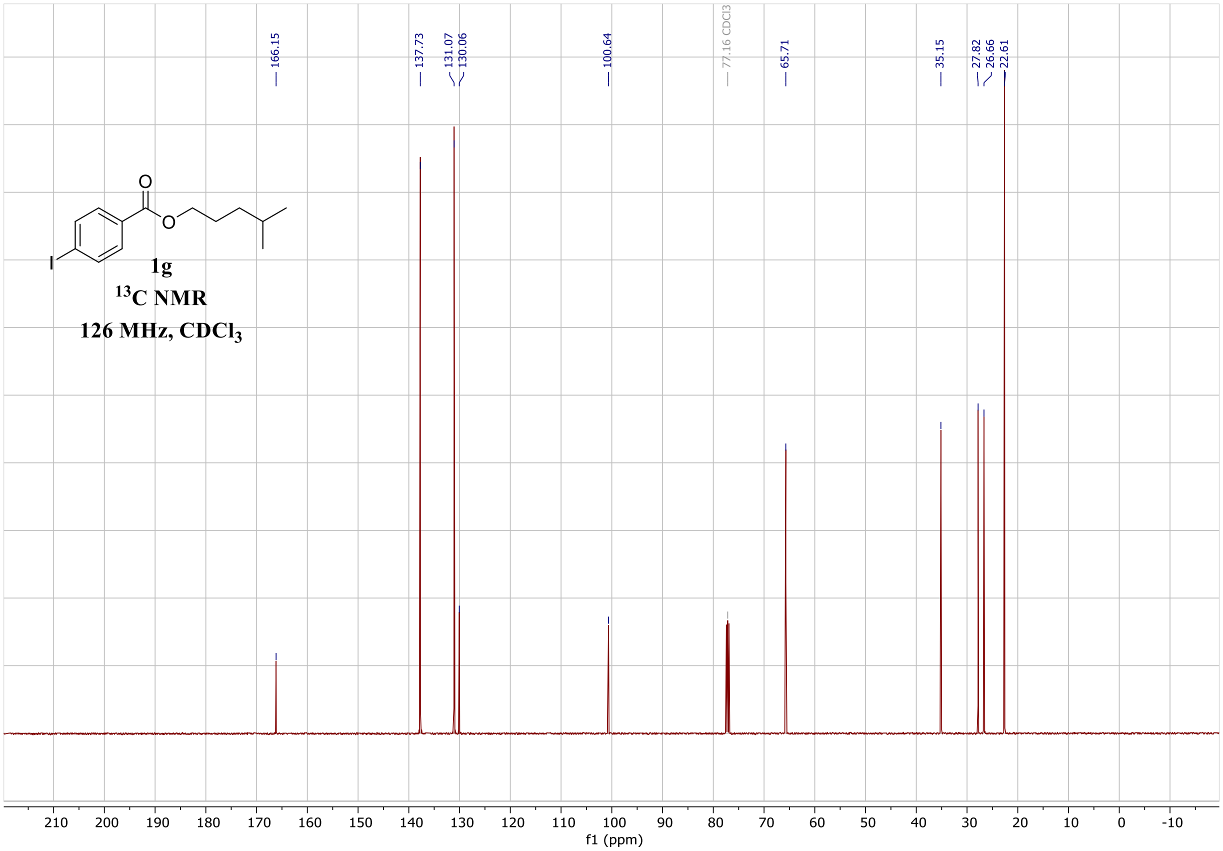
166.15
137.73
131.07
130.06
100.64
77.16 CDCl3
65.71
35.15
27.82
26.66
22.61
O
O
I
1g
^{13}C NMR
126 MHz, CDCl$_3$
f1 (ppm)

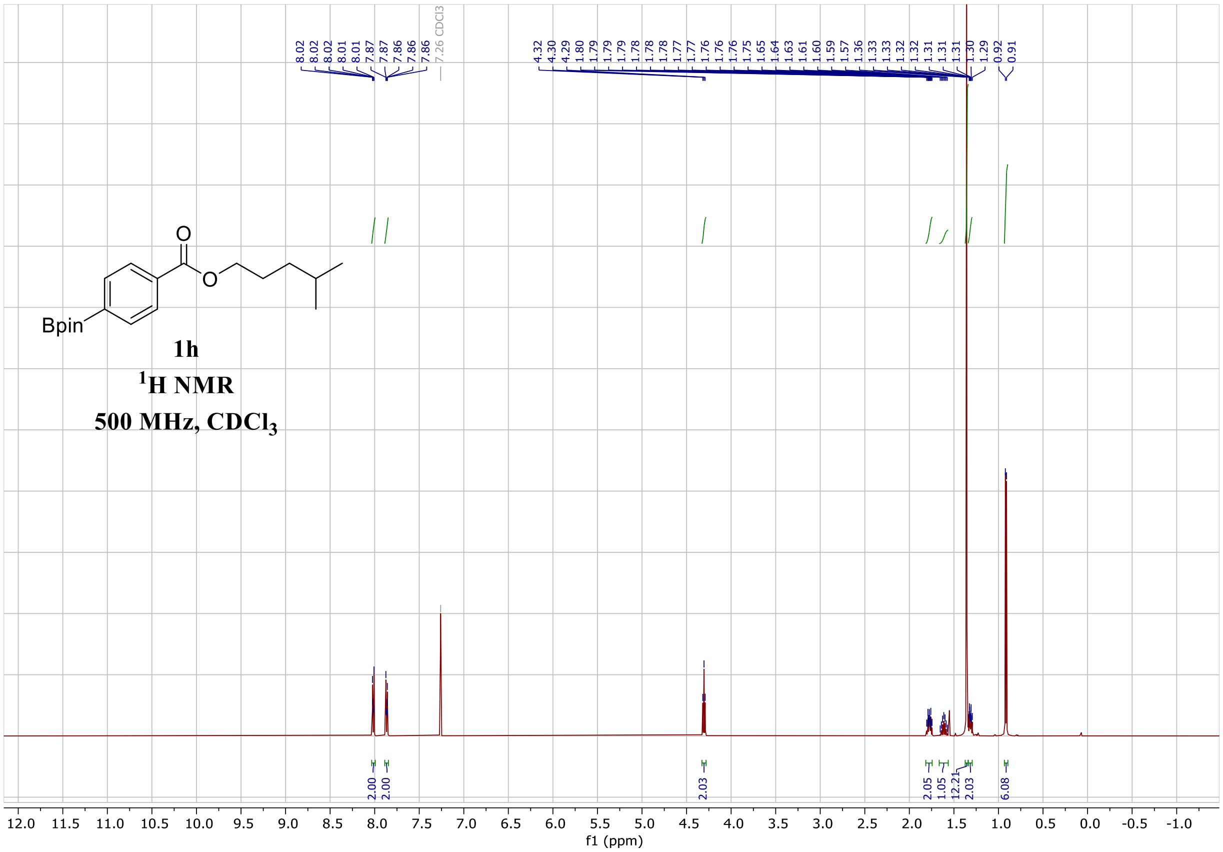
8.02
8.02
8.01
8.01
7.87
7.87
7.86
7.86
7.26 CDCl3
4.32
4.30
4.29
1.80
1.79
1.79
1.79
1.78
1.78
1.78
1.77
1.77
1.76
1.76
1.76
1.75
1.65
1.64
1.63
1.61
1.60
1.59
1.57
1.36
1.33
1.33
1.32
1.32
1.31
1.31
1.31
1.30
1.29
0.92
0.91
O
O
Bpin
1h
^{1}H NMR
500 MHz, CDCl$_3$
2.00
2.00
2.03
2.05
1.05
12.21
2.03
6.08
f1 (ppm)

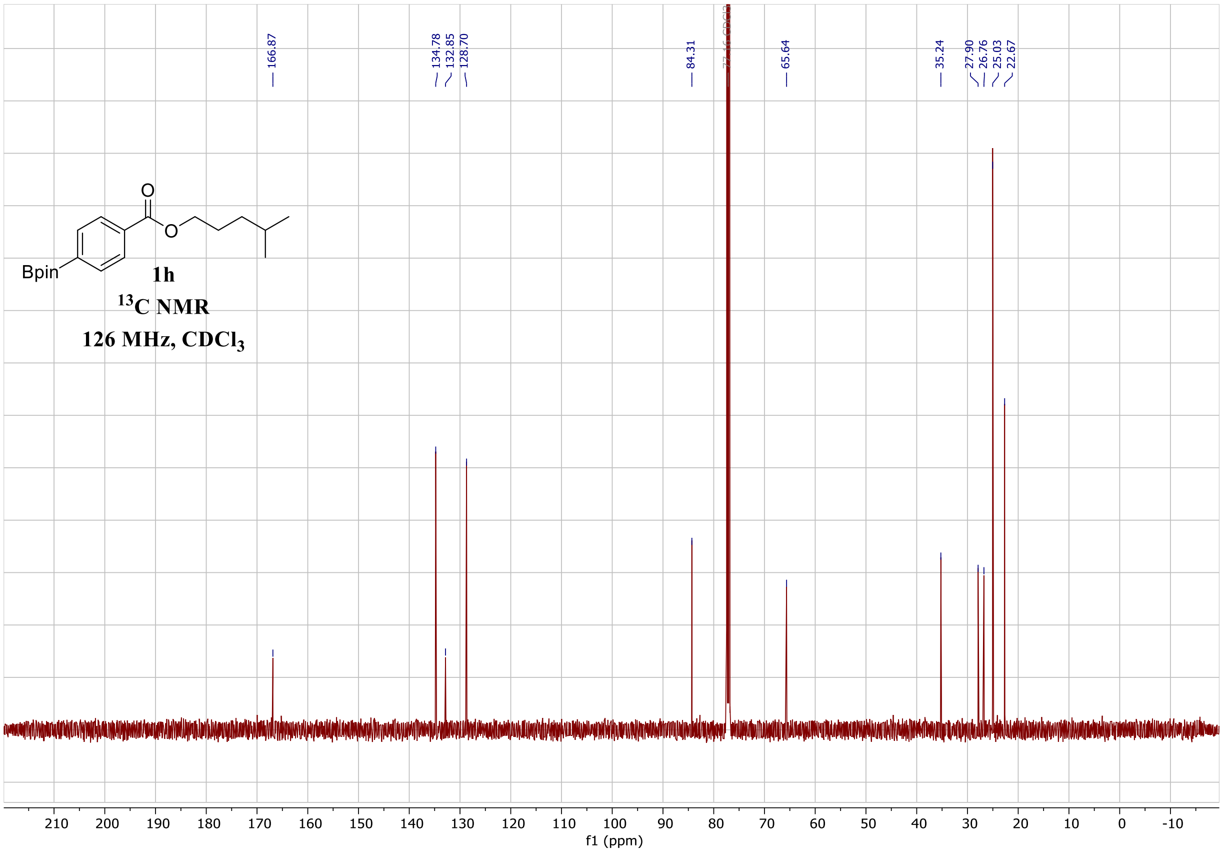
166.87
134.78
132.85
128.70
84.31
65.64
35.24
27.90
26.76
25.03
22.67
O
O
Bpin
1h
^{13}C NMR
126 MHz, $CDCl_3$
210 200 190 180 170 160 150 140 130 120 110 100 90 80 70 60 50 40 30 20 10 0 -10
f1 (ppm)

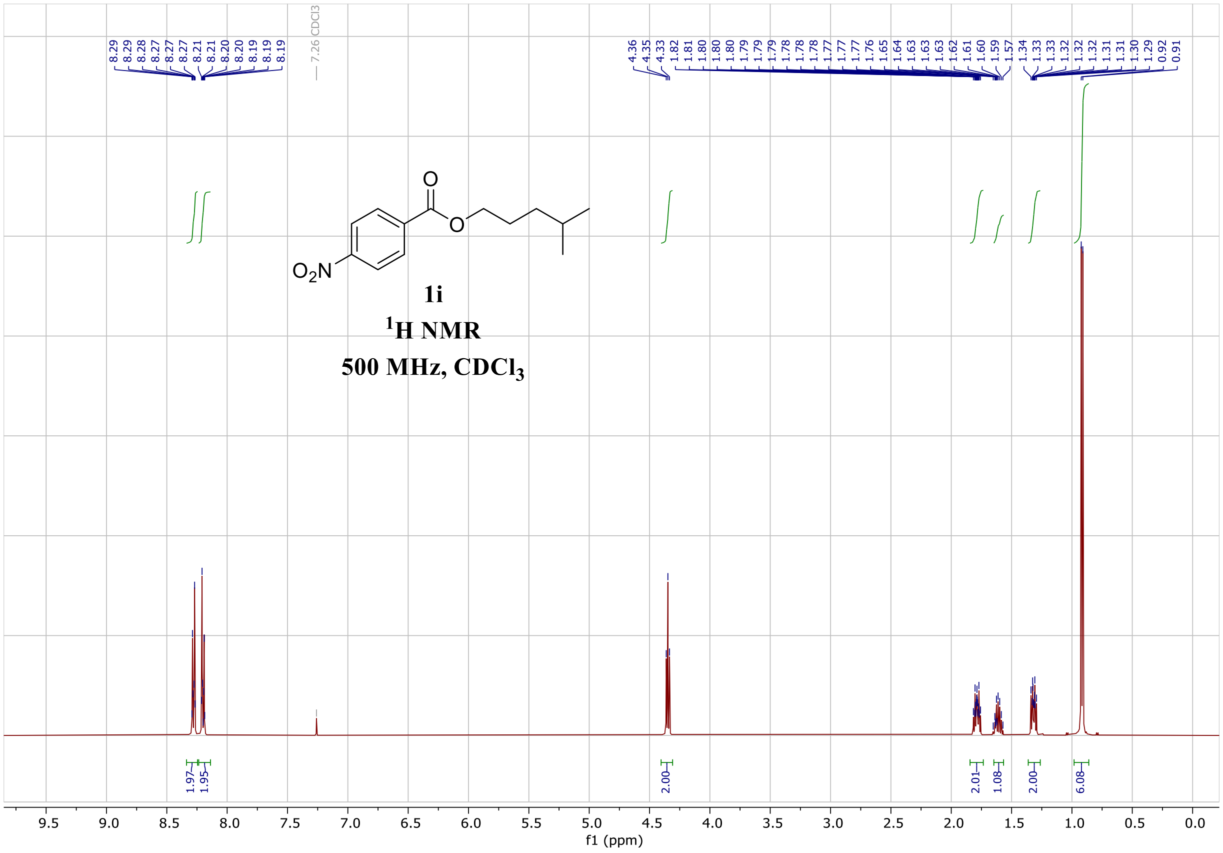
8.29 8.29 8.28 8.27 8.27 8.27 8.21 8.21 8.20 8.20 8.19 8.19 8.19
7.26 CDCl3
4.36 4.35 4.33 1.82 1.81 1.80 1.80 1.80 1.79 1.79 1.79 1.78 1.78 1.78 1.77 1.77 1.77 1.76 1.65 1.64 1.63 1.63 1.63 1.62 1.61 1.60 1.59 1.57 1.34 1.33 1.33 1.32 1.32 1.32 1.31 1.31 1.30 1.29 0.92 0.91
O
O
O_2N
1i
^{1}H NMR
500 MHz, $CDCl_3$
1.97
1.95
2.00
2.01
1.08
2.00
6.08
9.5 9.0 8.5 8.0 7.5 7.0 6.5 6.0 5.5 5.0 4.5 4.0 3.5 3.0 2.5 2.0 1.5 1.0 0.5 0.0
f1 (ppm)

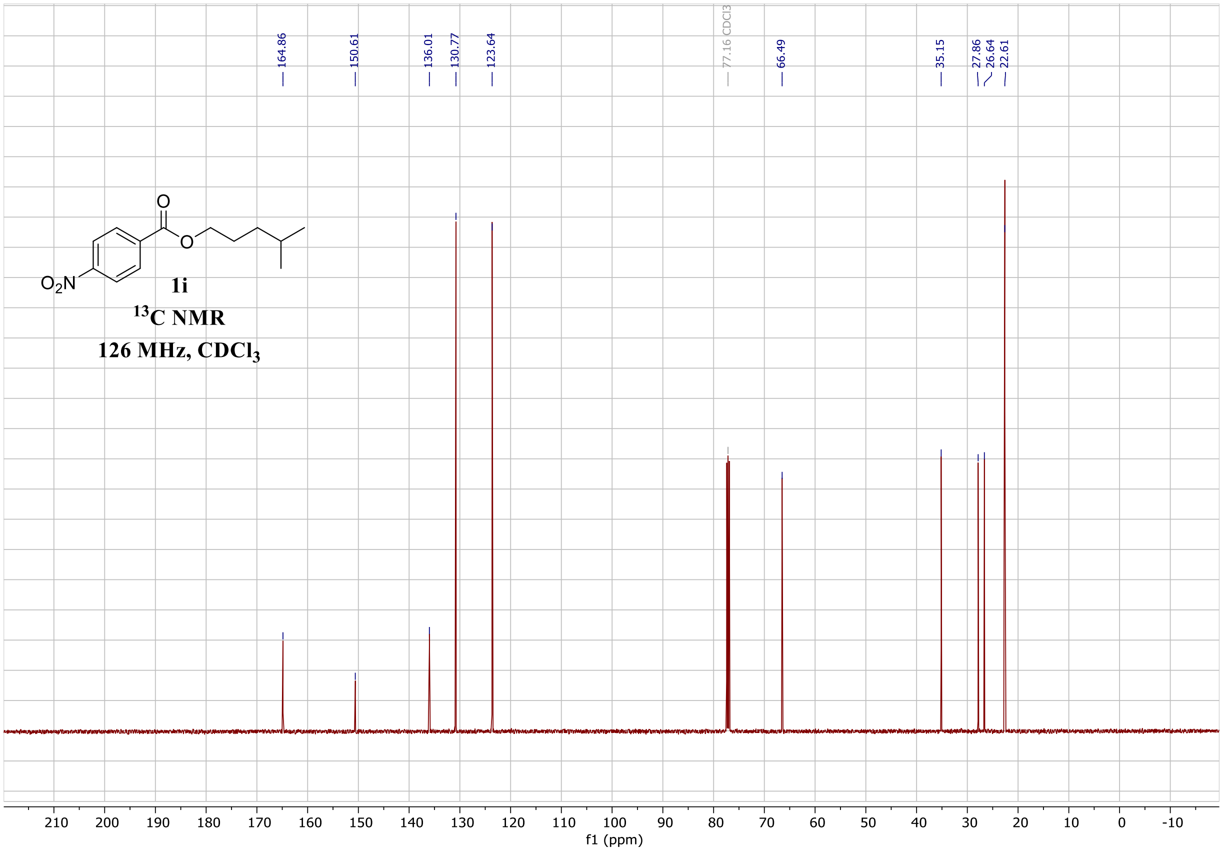

164.86
150.61
136.01
130.77
123.64
77.16 CDCl3
66.49
35.15
27.86
26.64
22.61
O
O
O_2N
1i
^{13}C NMR
126 MHz, $CDCl_3$
210 200 190 180 170 160 150 140 130 120 110 100 90 80 70 60 50 40 30 20 10 0 -10
f1 (ppm)

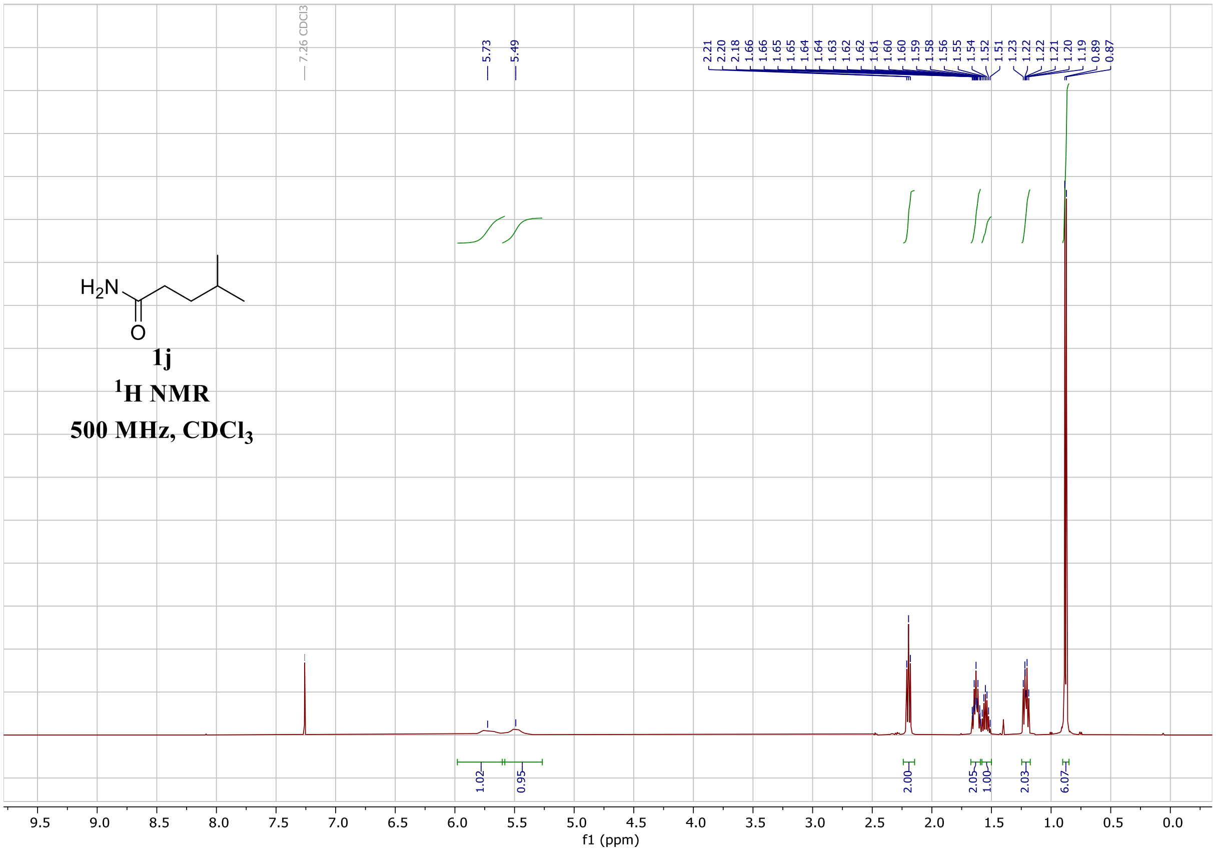

7.26 CDCl3
5.73
5.49
2.21 2.20 2.18 1.66 1.65 1.65 1.64 1.64 1.63 1.62 1.62 1.61 1.60 1.60 1.59 1.58 1.56 1.55 1.54 1.52 1.51 1.23 1.22 1.22 1.21 1.20 1.19 0.89 0.87
H_2N
O
1j
^1H NMR
500 MHz, $CDCl_3$
1.02
0.95
2.00
2.05
1.00
2.03
6.07
9.5 9.0 8.5 8.0 7.5 7.0 6.5 6.0 5.5 5.0 4.5 4.0 3.5 3.0 2.5 2.0 1.5 1.0 0.5 0.0
f1 (ppm)

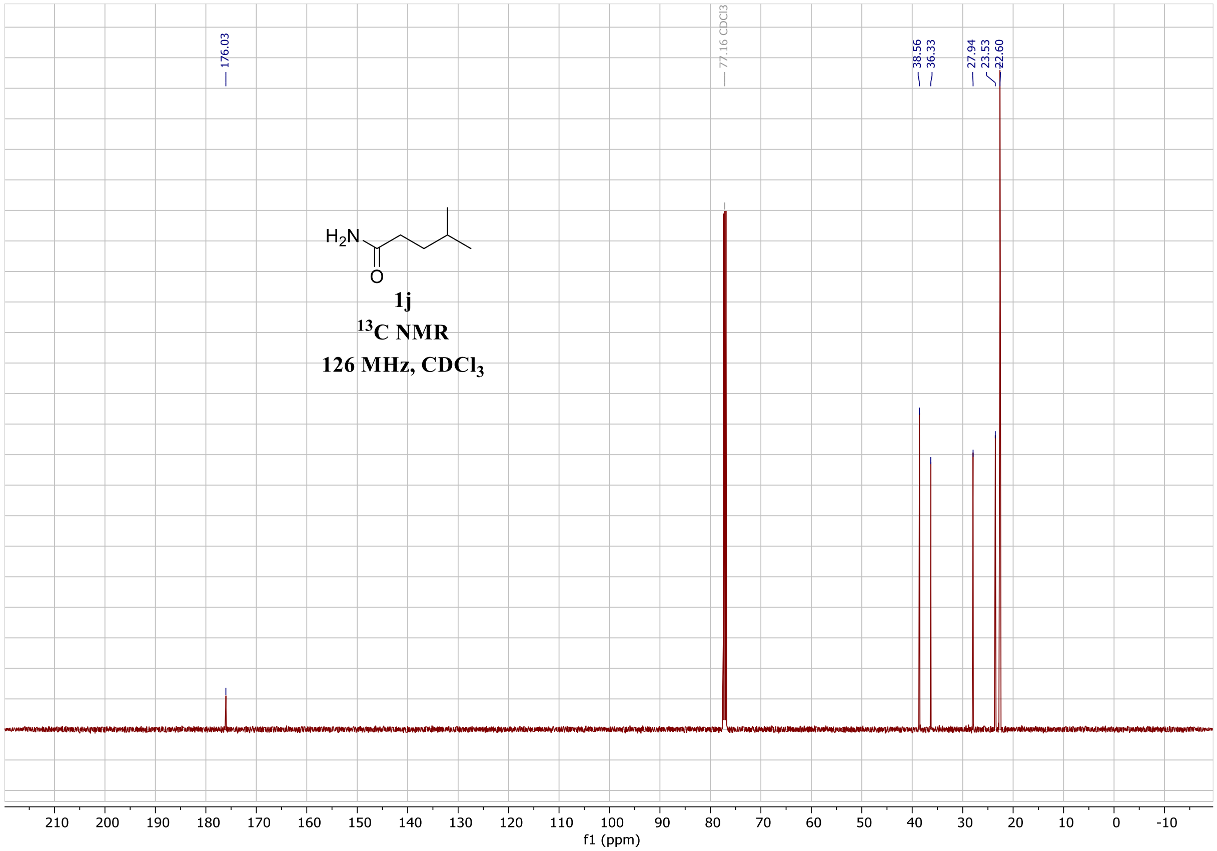

176.03
77.16 CDCl3
38.56
36.33
27.94
23.53
22.60
H_2N
O
1j
^{13}C NMR
126 MHz, $CDCl_3$
f1 (ppm)

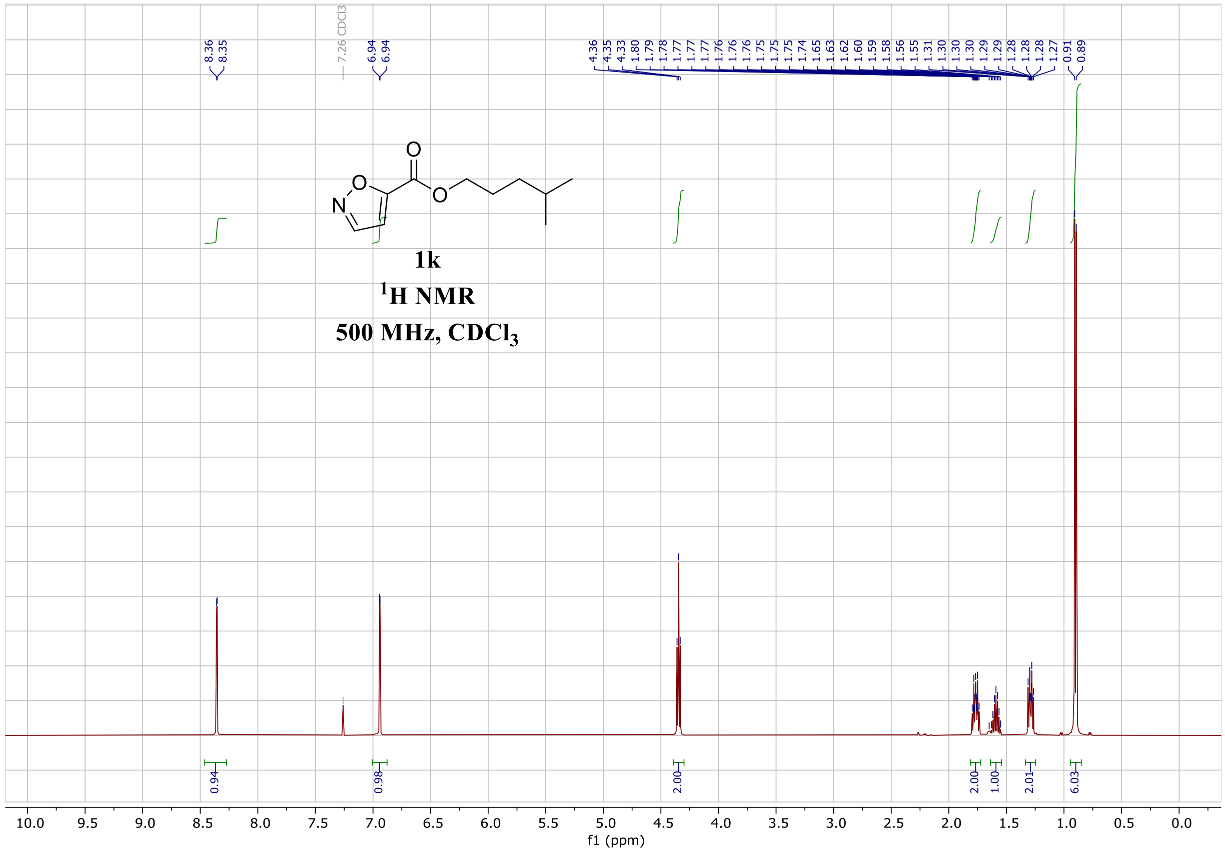

8.36
8.35
7.26 CDCl3
6.94
6.94
4.36 4.35 4.33 1.80 1.79 1.79 1.78 1.77 1.77 1.77 1.76 1.76 1.75 1.75 1.75 1.74 1.65 1.63 1.62 1.60 1.59 1.58 1.56 1.55 1.31 1.30 1.30 1.29 1.29 1.28 1.28 1.28 1.27 0.91 0.89
O
N
1k
^{1}H NMR
500 MHz, $CDCl_3$
0.94
0.98
2.00
2.00
1.00
2.01
6.03
f1 (ppm)

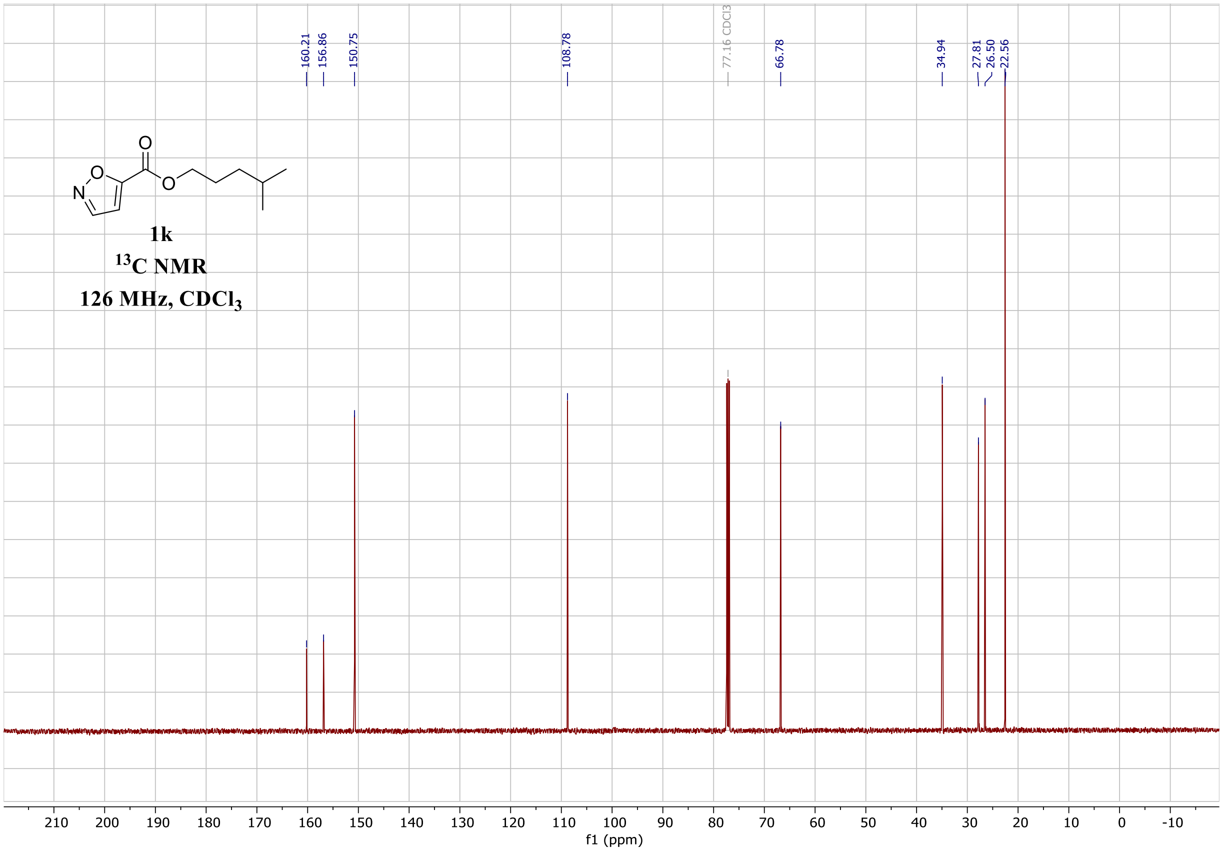

1k
13C NMR
126 MHz, CDCl3
160.21
156.86
150.75
108.78
77.16 CDCl3
66.78
34.94
27.81
26.50
22.56
f1 (ppm)

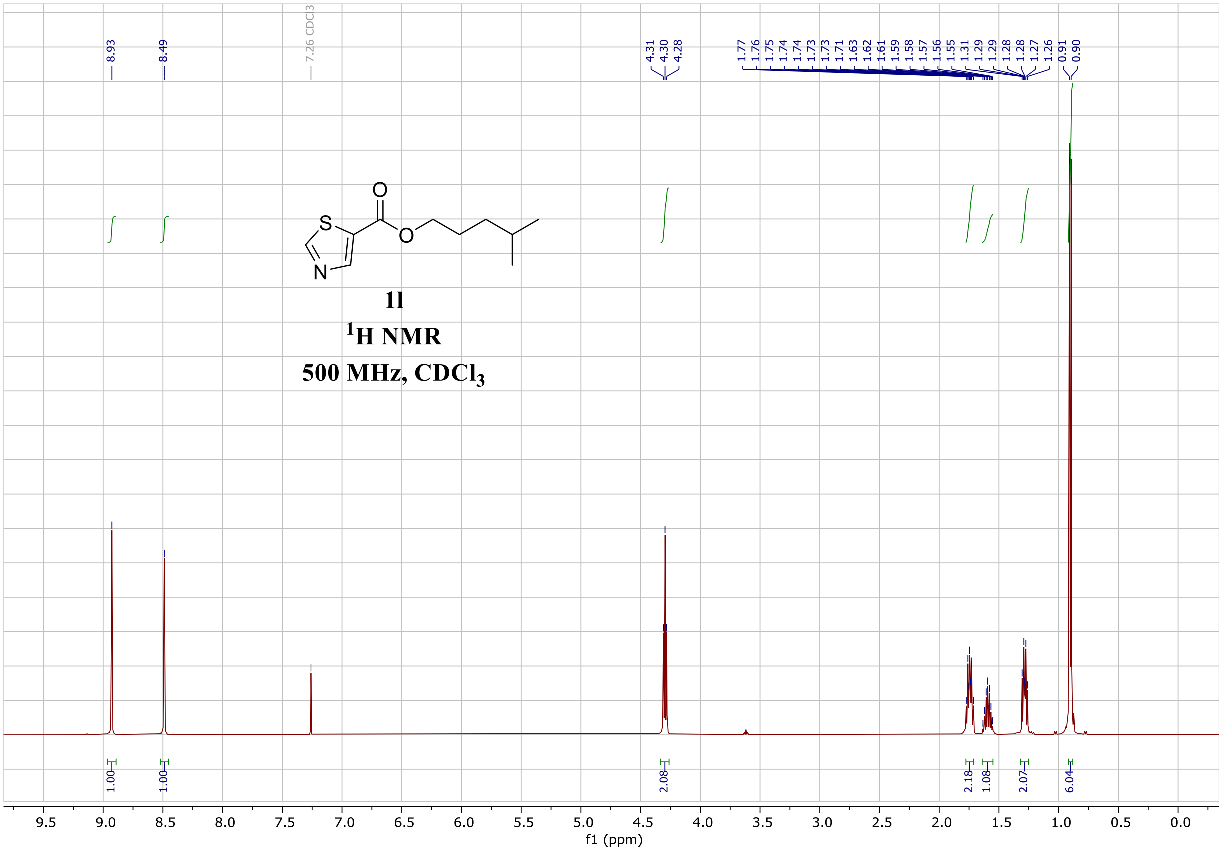

1l
1H NMR
500 MHz, CDCl3
8.93
8.49
7.26 CDCl3
4.31
4.30
4.28
1.77
1.76
1.75
1.74
1.74
1.73
1.73
1.71
1.63
1.62
1.61
1.59
1.58
1.57
1.56
1.55
1.31
1.29
1.29
1.28
1.28
1.27
1.26
0.91
0.90
1.00
1.00
2.08
2.18
1.08
2.07
6.04
f1 (ppm)

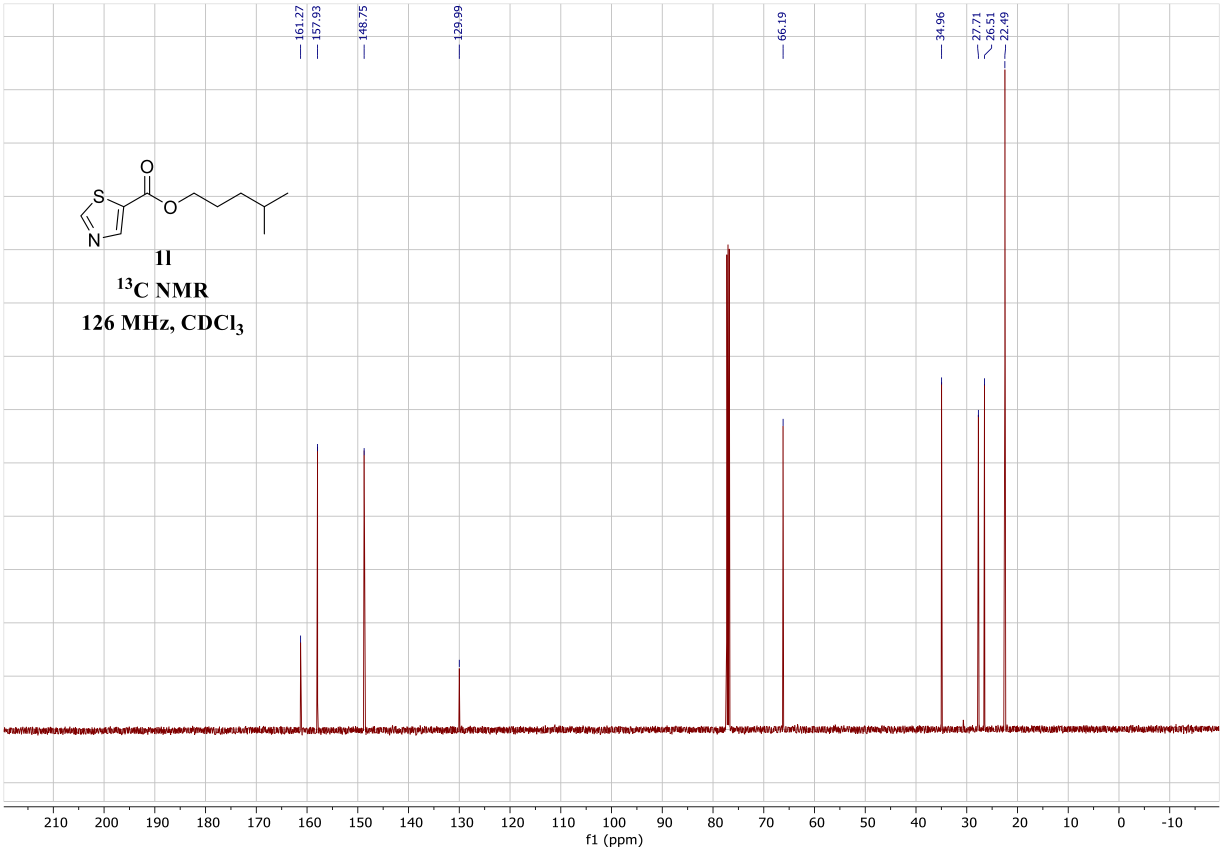
161.27
157.93
148.75
129.99
66.19
34.96
27.71
26.51
22.49
1l
^{13}C NMR
126 MHz, $CDCl_3$
f1 (ppm)

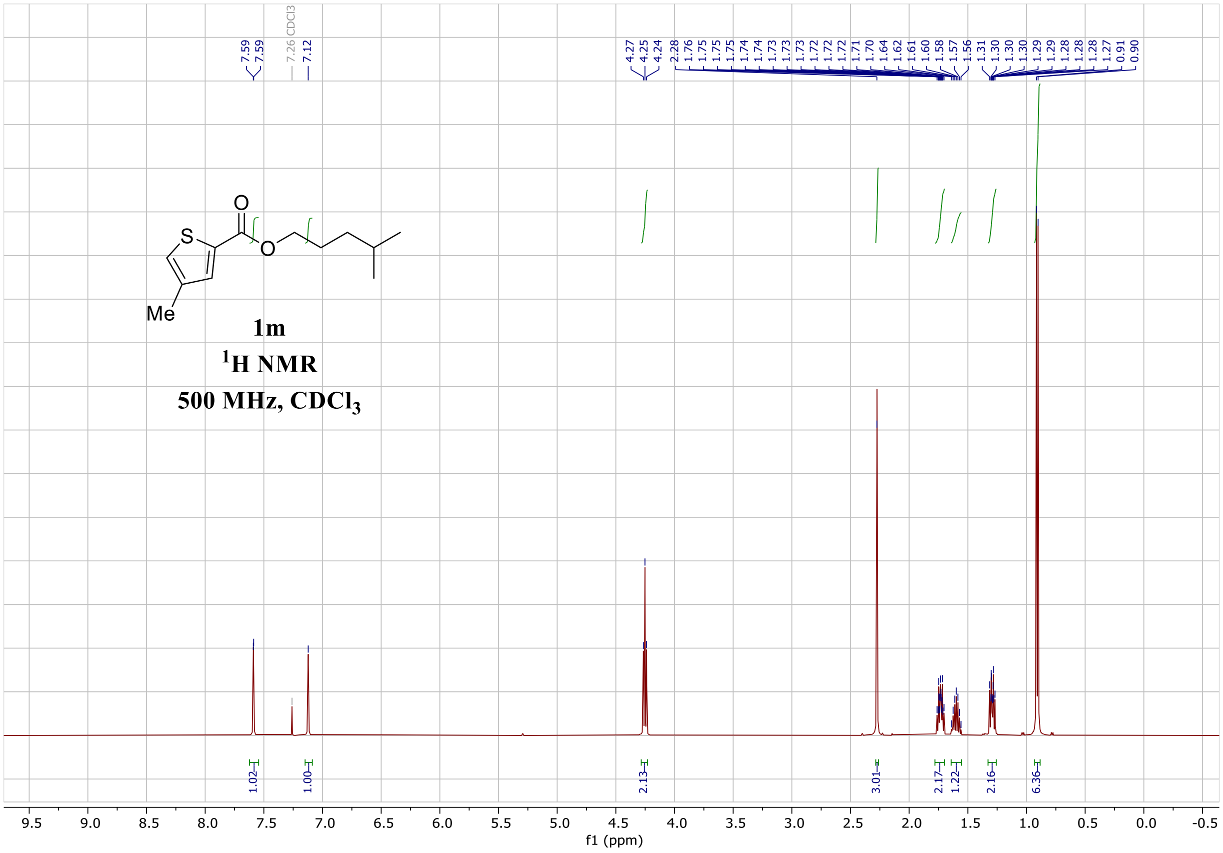
7.59
7.59
7.26 CDCl3
7.12
4.27
4.25
4.24
2.28
0.91
0.90
Me
1m
^{1}H NMR
500 MHz, $CDCl_3$
1.02
1.00
2.13
3.01
2.17
1.22
2.16
6.36
f1 (ppm)

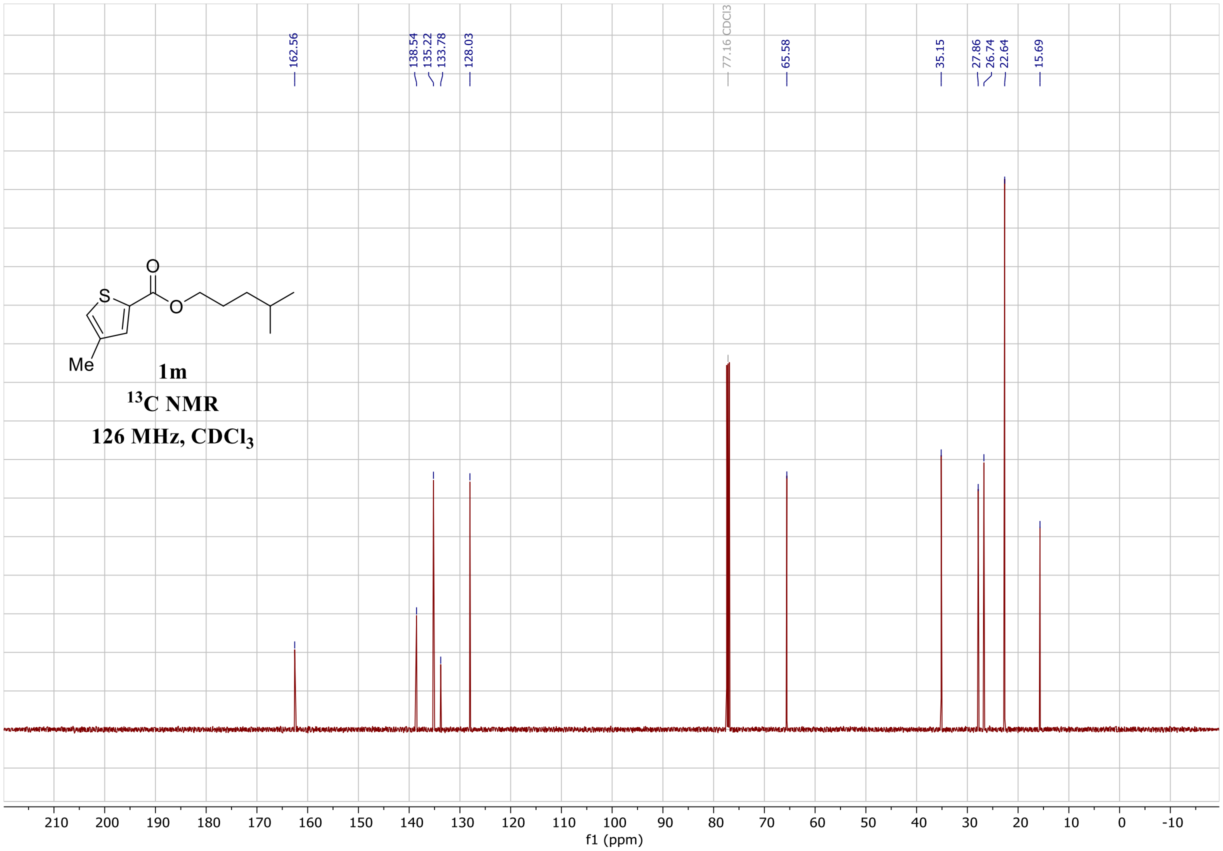
162.56
138.54
135.22
133.78
128.03
77.16 CDCl3
65.58
35.15
27.86
26.74
22.64
15.69
Me
S
O
1m
^{13}C NMR
126 MHz, $CDCl_3$
f1 (ppm)

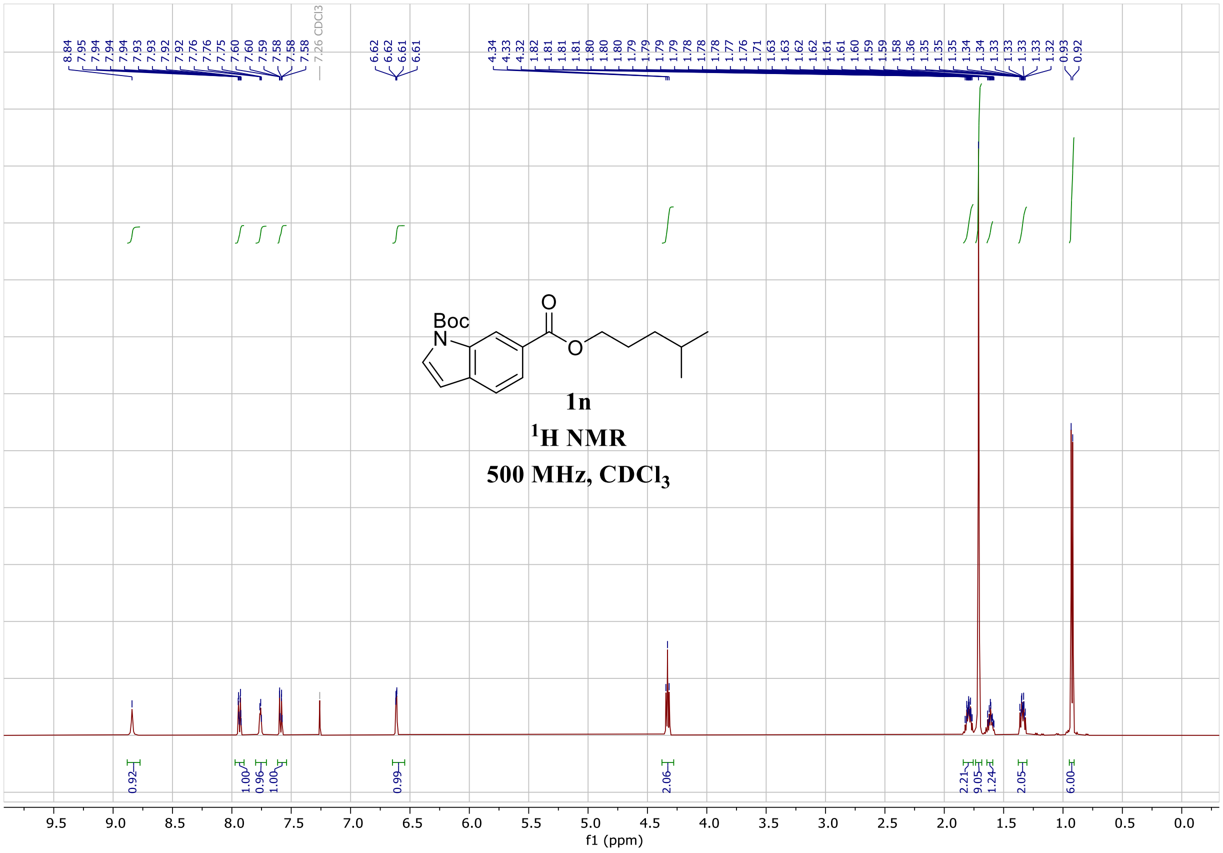
8.84
7.95
7.94
7.94
7.93
7.93
7.92
7.92
7.76
7.76
7.75
7.60
7.60
7.59
7.58
7.58
7.58
7.26 CDCl3
6.62
6.62
6.61
6.61
4.34
4.33
4.32
1.82
1.81
1.81
1.81
1.80
1.80
1.80
1.79
1.79
1.79
1.78
1.78
1.78
1.77
1.76
1.71
1.63
1.63
1.62
1.62
1.61
1.61
1.60
1.59
1.59
1.58
1.36
1.35
1.35
1.35
1.34
1.34
1.33
1.33
1.33
1.33
1.32
0.93
0.92
Boc
N
O
1n
^{1}H NMR
500 MHz, $CDCl_3$
0.92
1.00
0.96
1.00
0.99
2.06
2.21
9.05
1.24
2.05
6.00
f1 (ppm)

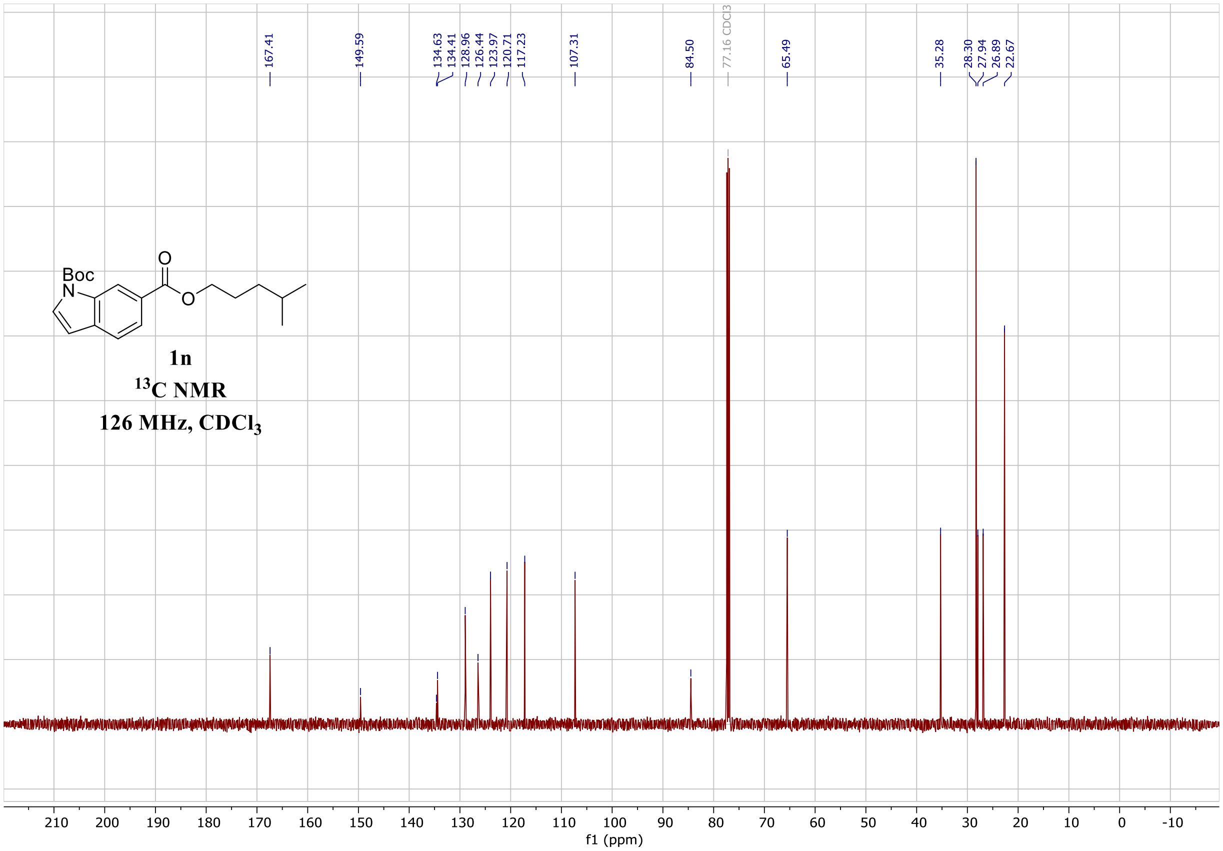
Boc
N
O
O
1n
^{13}C NMR
126 MHz, CDCl$_3$
f1 (ppm)

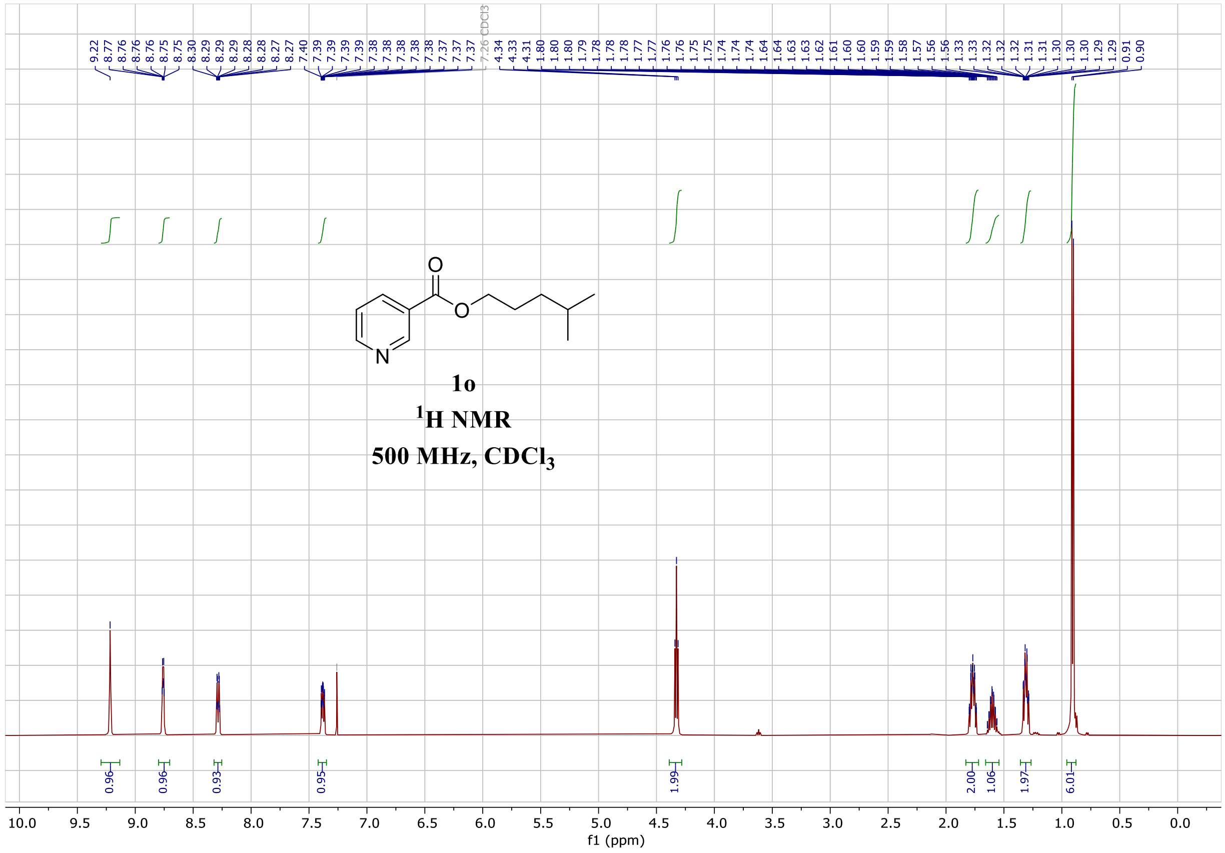
O
O
N
1o
^{1}H NMR
500 MHz, CDCl$_3$
f1 (ppm)

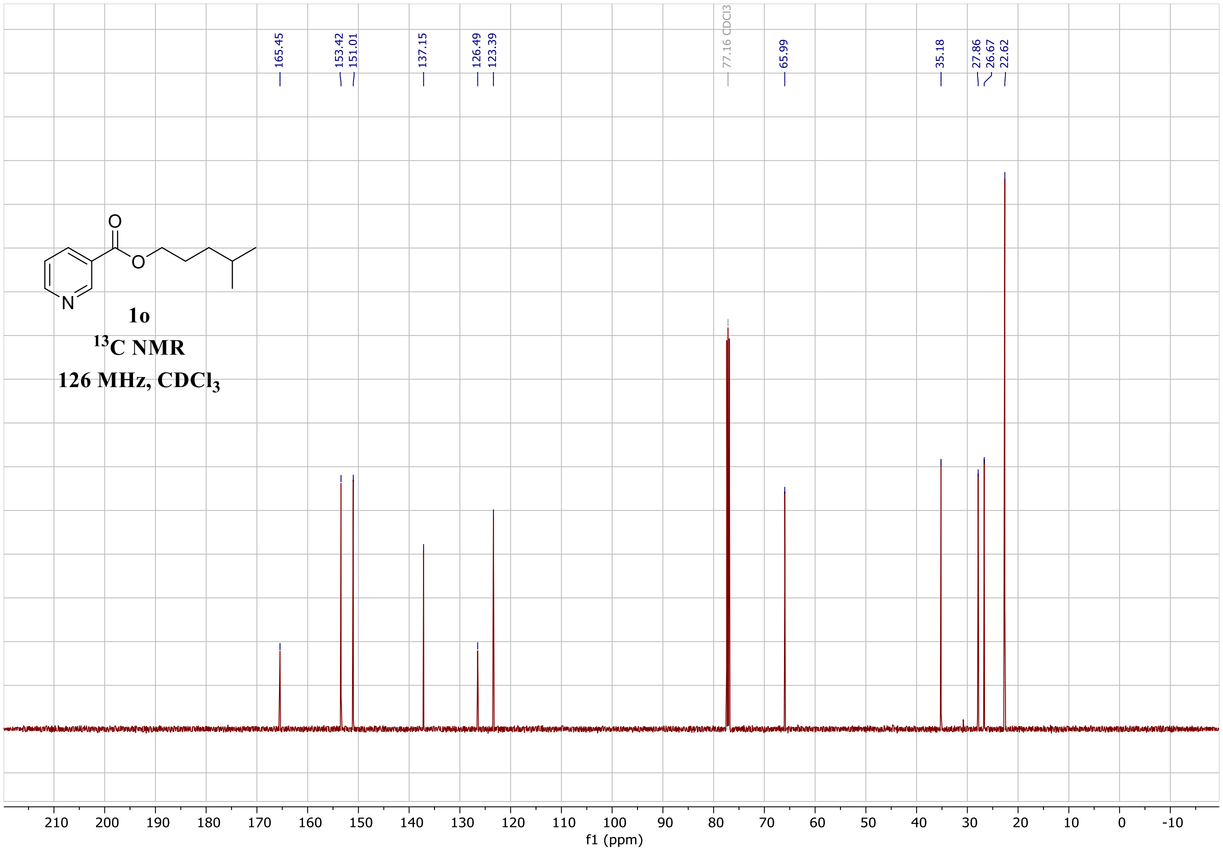
165.45
153.42
151.01
137.15
126.49
123.39
77.16 CDCl3
65.99
35.18
27.86
26.67
22.62
O
O
N
1o
^{13}C NMR
126 MHz, $CDCl_3$
210 200 190 180 170 160 150 140 130 120 110 100 90 80 70 60 50 40 30 20 10 0 -10
f1 (ppm)

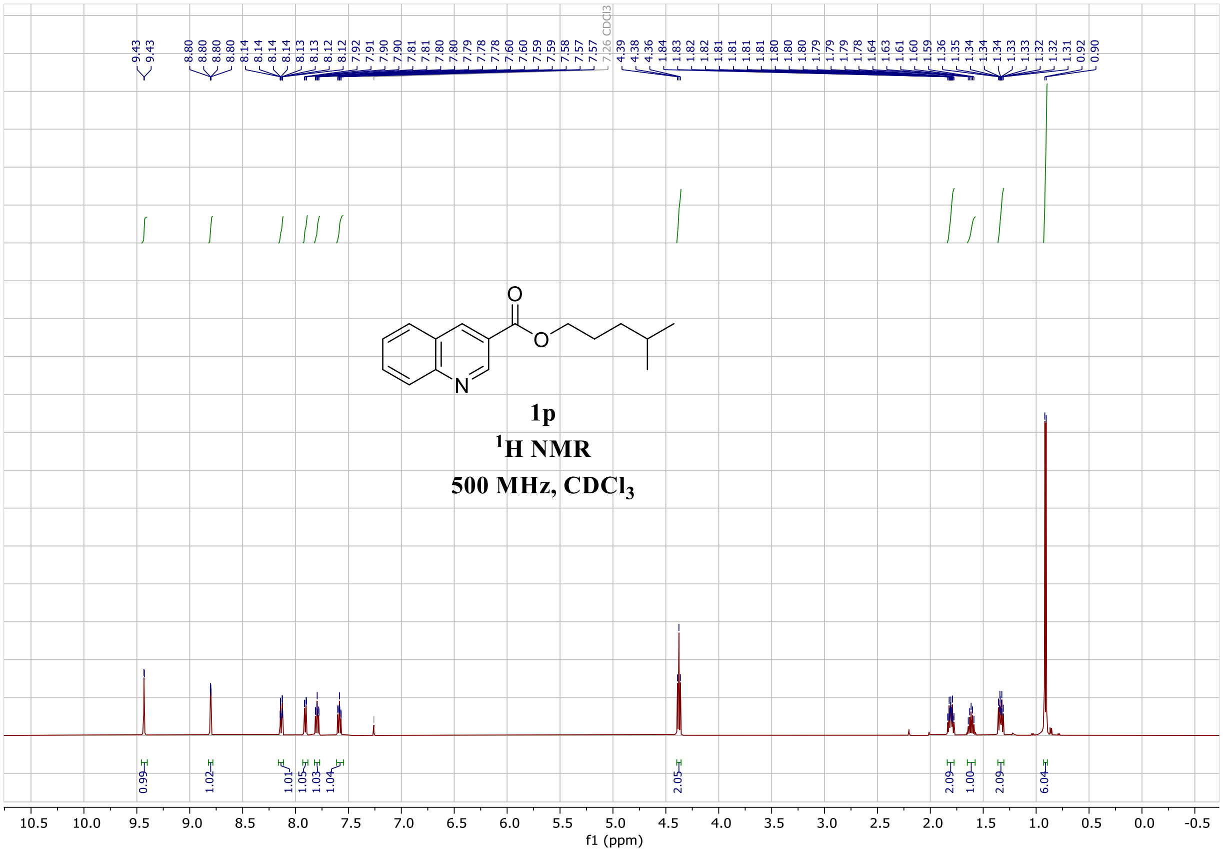
9.43
9.43
8.80
8.80
8.80
8.80
8.14
8.14
8.14
8.13
8.13
8.12
8.12
7.92
7.91
7.90
7.90
7.81
7.81
7.80
7.80
7.79
7.78
7.78
7.60
7.60
7.59
7.58
7.57
7.57
7.26 CDCl3
4.39
4.38
4.36
1.84
1.83
1.82
1.82
1.81
1.81
1.81
1.80
1.80
1.80
1.79
1.79
1.78
1.64
1.63
1.61
1.60
1.59
1.36
1.35
1.34
1.34
1.33
1.33
1.32
1.32
1.31
0.92
0.90
O
O
N
1p
^{1}H NMR
500 MHz, $CDCl_3$
0.99
1.02
1.01
1.05
1.03
1.04
2.05
2.09
1.00
2.09
6.04
10.5 10.0 9.5 9.0 8.5 8.0 7.5 7.0 6.5 6.0 5.5 5.0 4.5 4.0 3.5 3.0 2.5 2.0 1.5 1.0 0.5 0.0 -0.5
f1 (ppm)

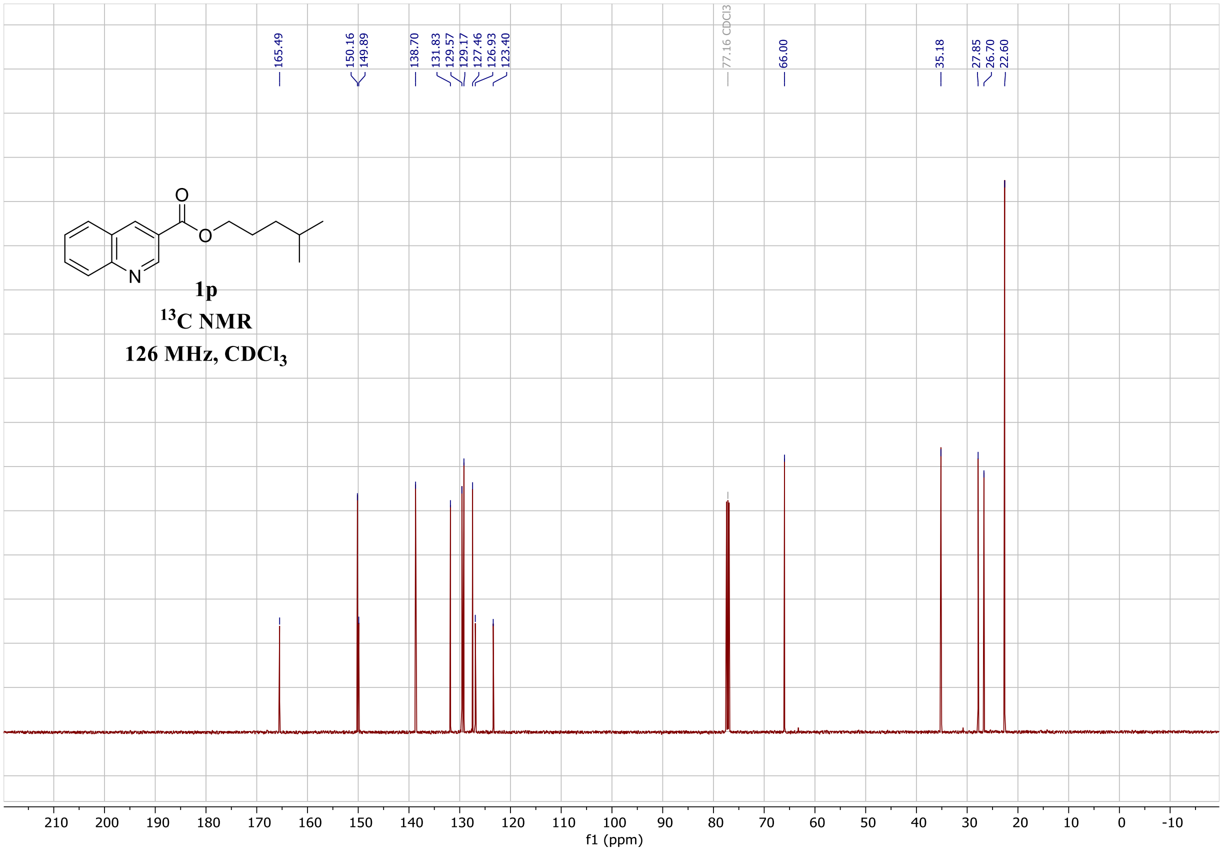
1p
^{13}C NMR
126 MHz, $CDCl_3$
165.49
150.16
149.89
138.70
131.83
129.57
129.17
127.46
126.93
123.40
77.16 CDCl3
66.00
35.18
27.85
26.70
22.60
f1 (ppm)

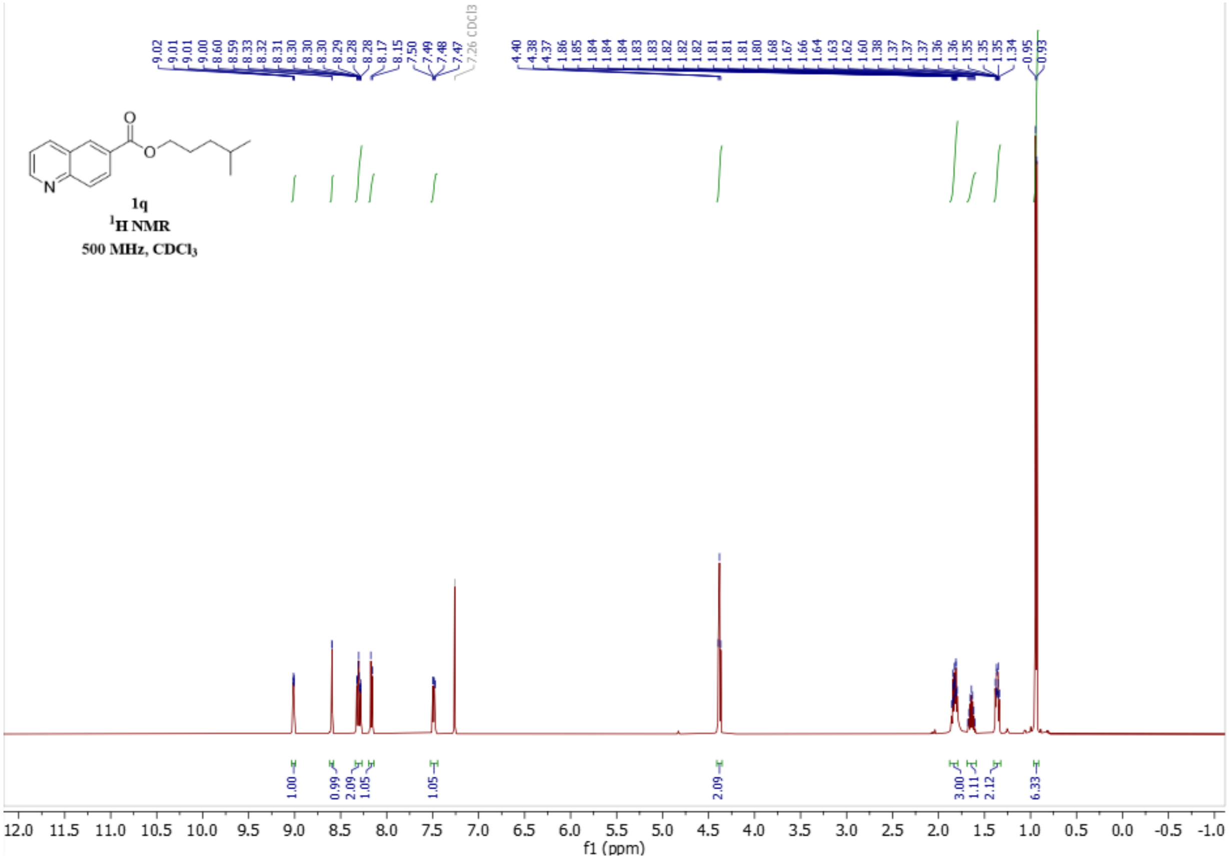
1q
^{1}H NMR
500 MHz, $CDCl_3$
9.02
9.01
9.01
9.00
8.60
8.59
8.33
8.32
8.31
8.30
8.30
8.30
8.29
8.28
8.28
8.17
8.15
7.50
7.49
7.48
7.47
7.26 CDCl3
4.40
4.38
4.37
1.86
1.85
1.84
1.84
1.84
1.83
1.83
1.82
1.82
1.81
1.81
1.81
1.80
1.68
1.67
1.66
1.64
1.63
1.62
1.60
1.38
1.37
1.37
1.37
1.36
1.35
1.35
1.35
1.34
0.95
0.93
1.00
0.99
2.09
1.05
1.05
2.09
3.00
1.11
2.12
6.33
f1 (ppm)

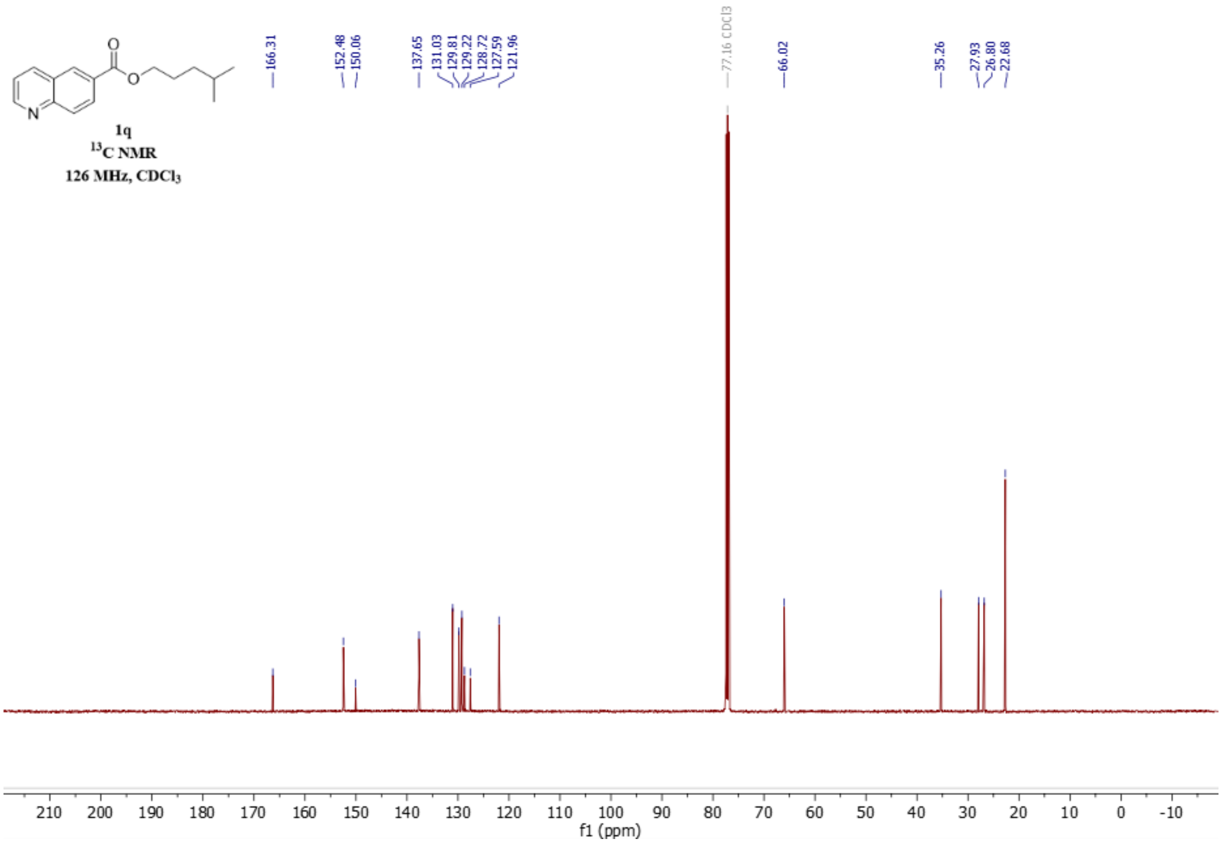
1q
^{13}C NMR
126 MHz, $CDCl_3$
166.31
152.48
150.06
137.65
131.03
129.81
129.22
128.72
127.59
121.96
77.16 CDCl3
66.02
35.26
27.93
26.80
22.68
f1 (ppm)

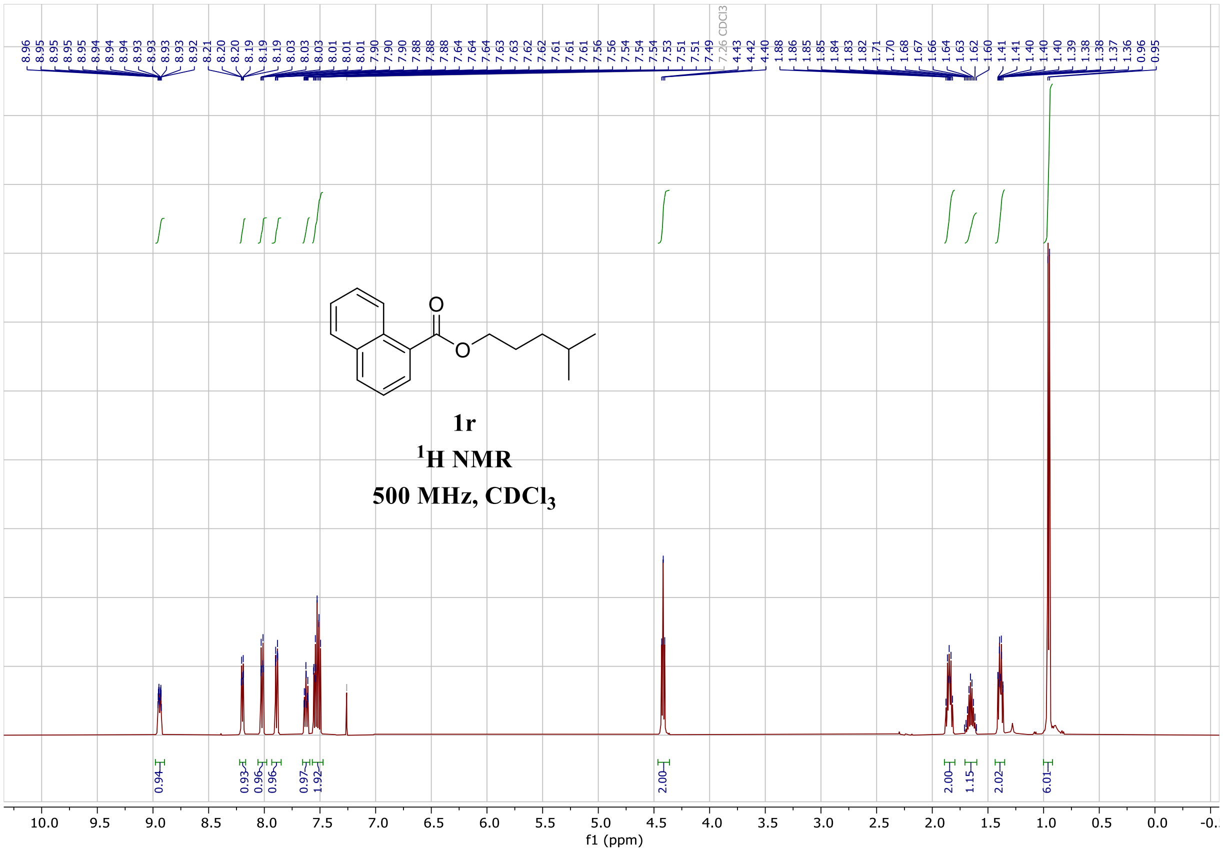
1r
^{1}H NMR
500 MHz, $CDCl_3$
8.96 8.95 8.95 8.95 8.94 8.94 8.94 8.93 8.93 8.93 8.93 8.92 8.21 8.20 8.19 8.19 8.19 8.03 8.03 8.03 8.01 8.01 8.01 7.90 7.90 7.90 7.88 7.88 7.64 7.64 7.64 7.63 7.63 7.62 7.62 7.61 7.61 7.61 7.56 7.56 7.54 7.54 7.54 7.53 7.51 7.49
7.26 CDCl3
4.43 4.42 4.40 1.88 1.86 1.85 1.84 1.83 1.82 1.71 1.70 1.68 1.67 1.66 1.64 1.63 1.62 1.60 1.41 1.41 1.40 1.40 1.39 1.38 1.38 1.37 1.36 0.96 0.95
0.94 0.93 0.96 0.96 0.97 1.92 2.00 2.00 1.15 2.02 6.01
f1 (ppm)

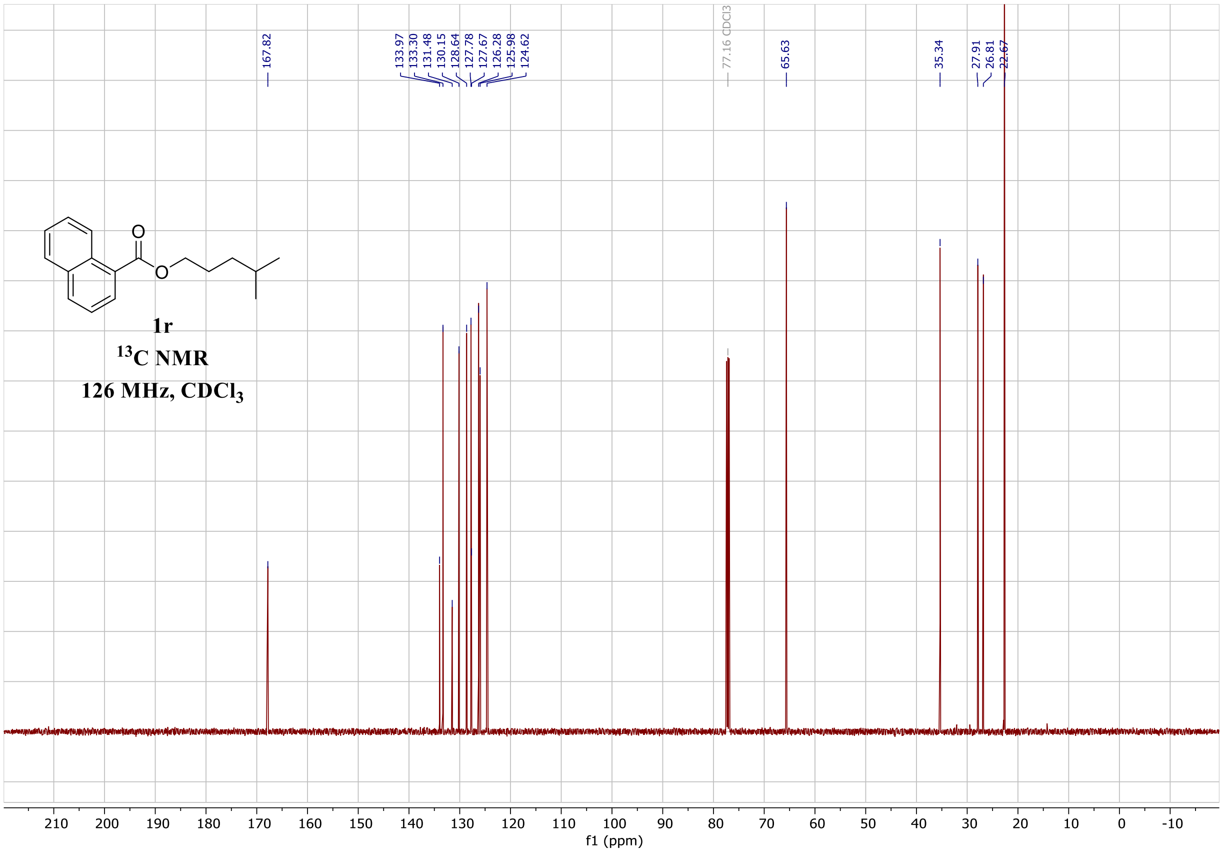
167.82
133.97
133.30
131.48
130.15
128.64
127.78
127.67
126.28
125.98
124.62
77.16 CDCl3
65.63
35.34
27.91
26.81
22.67
1r
13C NMR
126 MHz, CDCl3
f1 (ppm)

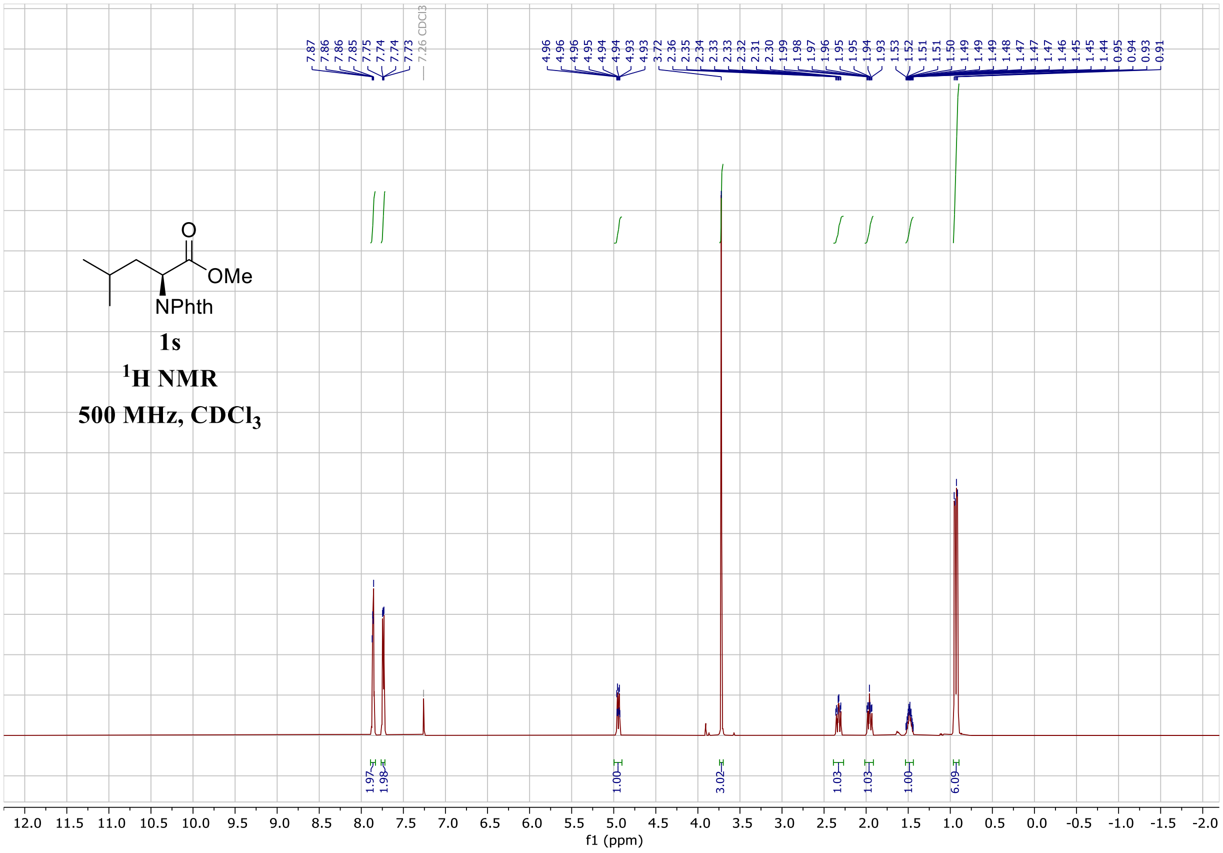
7.87
7.86
7.85
7.75
7.74
7.74
7.73
7.26 CDCl3
4.96
4.96
4.95
4.94
4.94
4.93
4.93
3.72
2.36
2.35
2.34
2.33
2.33
2.32
2.31
2.30
1.99
1.98
1.97
1.96
1.95
1.95
1.94
1.93
1.53
1.52
1.51
1.51
1.50
1.49
1.49
1.49
1.48
1.47
1.47
1.46
1.45
1.44
0.95
0.94
0.93
0.91
OMe
NPhth
1s
1H NMR
500 MHz, CDCl3
1.97
1.98
1.00
3.02
1.03
1.03
1.00
6.09
f1 (ppm)

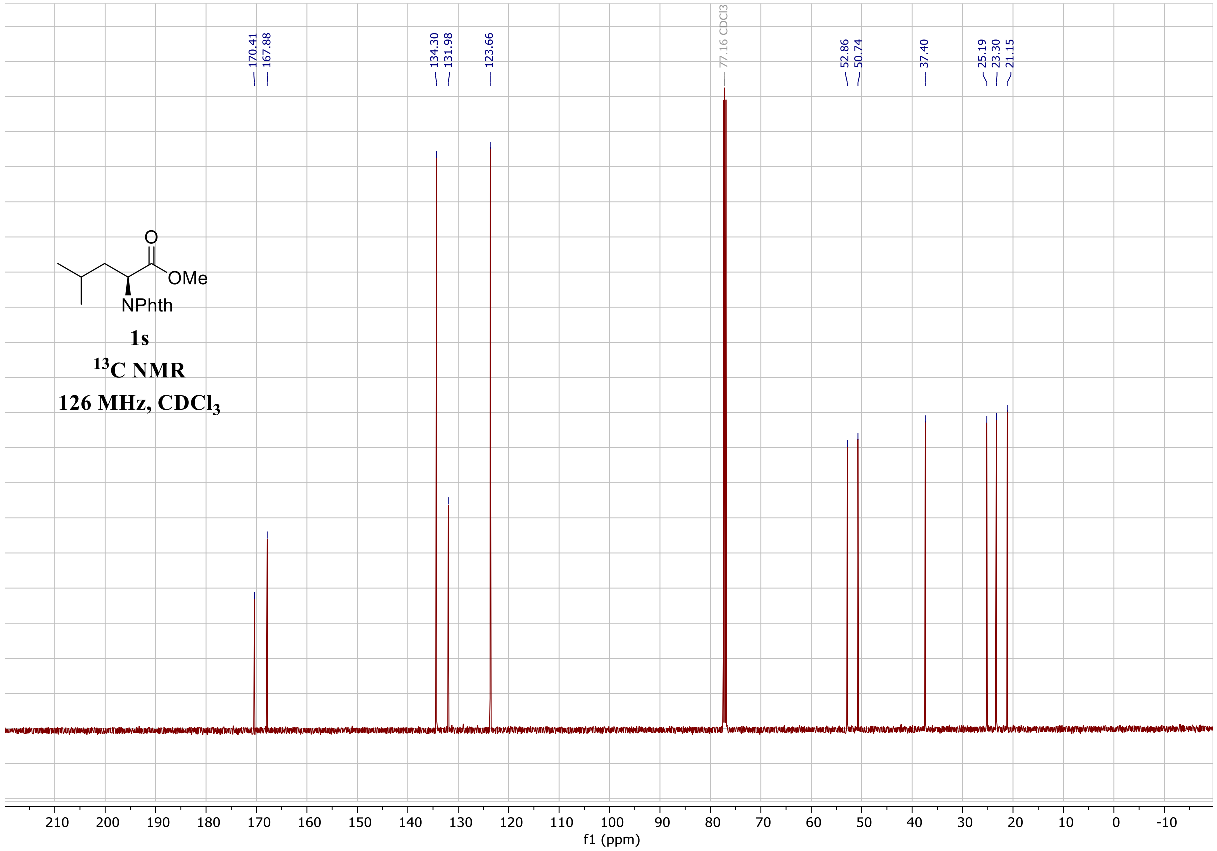
170.41
167.88
134.30
131.98
123.66
77.16 CDCl3
52.86
50.74
37.40
25.19
23.30
21.15
O
OMe
NPhth
1s
13C NMR
126 MHz, CDCl3
f1 (ppm)

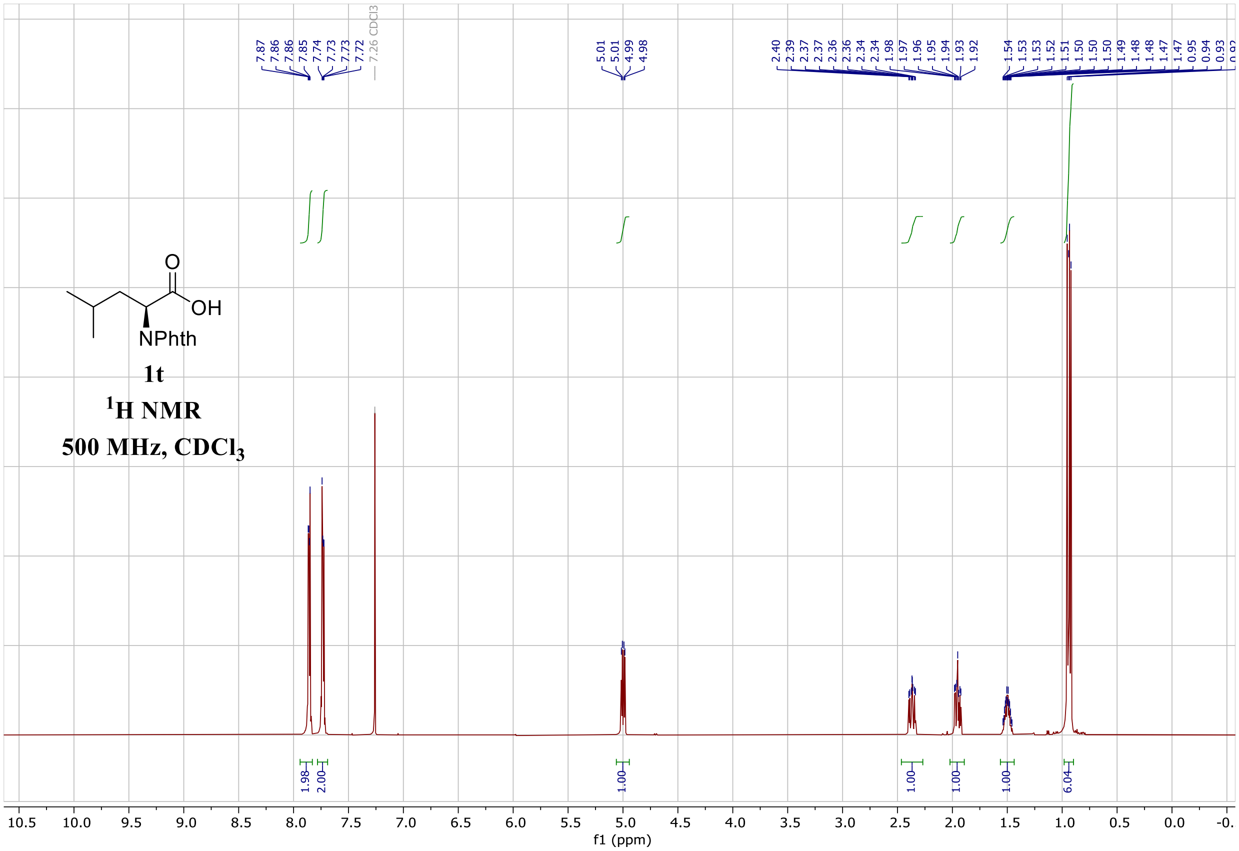
7.87
7.86
7.86
7.85
7.74
7.73
7.73
7.72
7.26 CDCl3
5.01
5.01
4.99
4.98
2.40
2.39
2.37
2.37
2.36
2.34
2.34
1.98
1.97
1.96
1.95
1.94
1.93
1.92
1.54
1.53
1.53
1.52
1.51
1.50
1.50
1.50
1.49
1.48
1.48
1.47
1.47
0.95
0.94
0.93
O
OH
NPhth
1t
1H NMR
500 MHz, CDCl3
1.98
2.00
1.00
1.00
1.00
1.00
6.04
f1 (ppm)

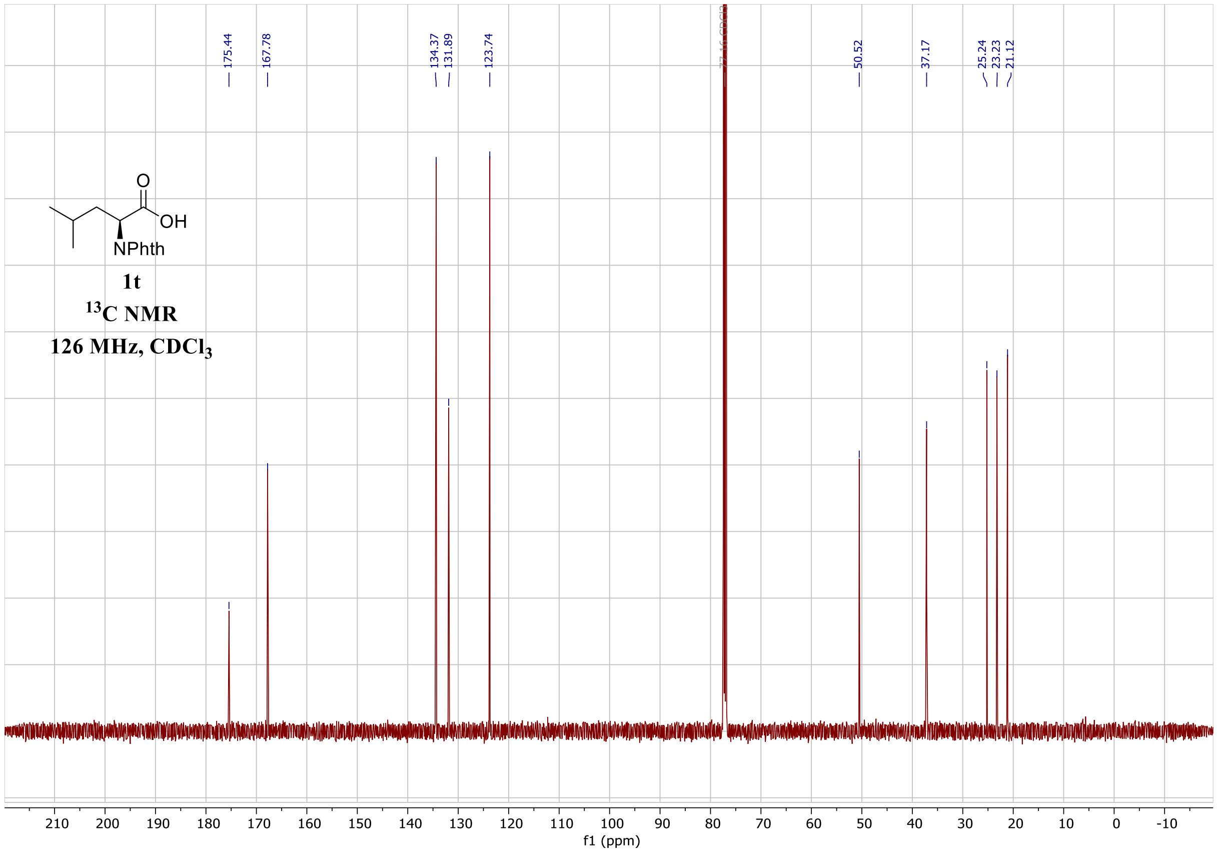
175.44
167.78
134.37
131.89
123.74
77.16 CDCl3
50.52
37.17
25.24
23.23
21.12
OH
O
NPhth
1t
^{13}C NMR
126 MHz, $CDCl_3$
f1 (ppm)

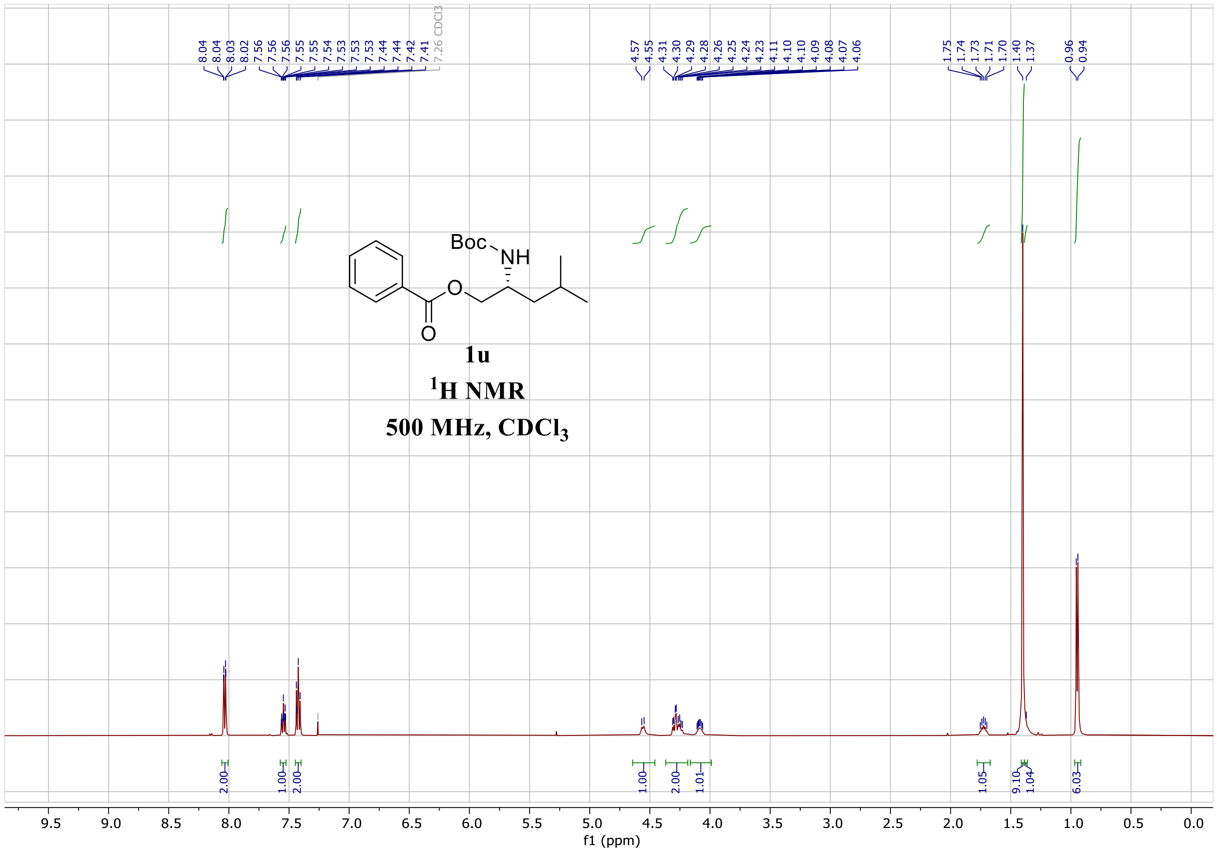
8.04
8.04
8.03
8.02
7.56
7.56
7.56
7.55
7.55
7.54
7.53
7.53
7.53
7.44
7.44
7.42
7.41
7.26 CDCl3
4.57
4.55
4.31
4.30
4.29
4.28
4.26
4.25
4.24
4.23
4.11
4.10
4.10
4.09
4.08
4.07
4.06
1.75
1.74
1.73
1.71
1.70
1.40
1.37
0.96
0.94
Boc
NH
O
O
1u
^{1}H NMR
500 MHz, $CDCl_3$
2.00
1.00
2.00
1.00
2.00
1.01
1.05
9.10
1.04
6.03
f1 (ppm)

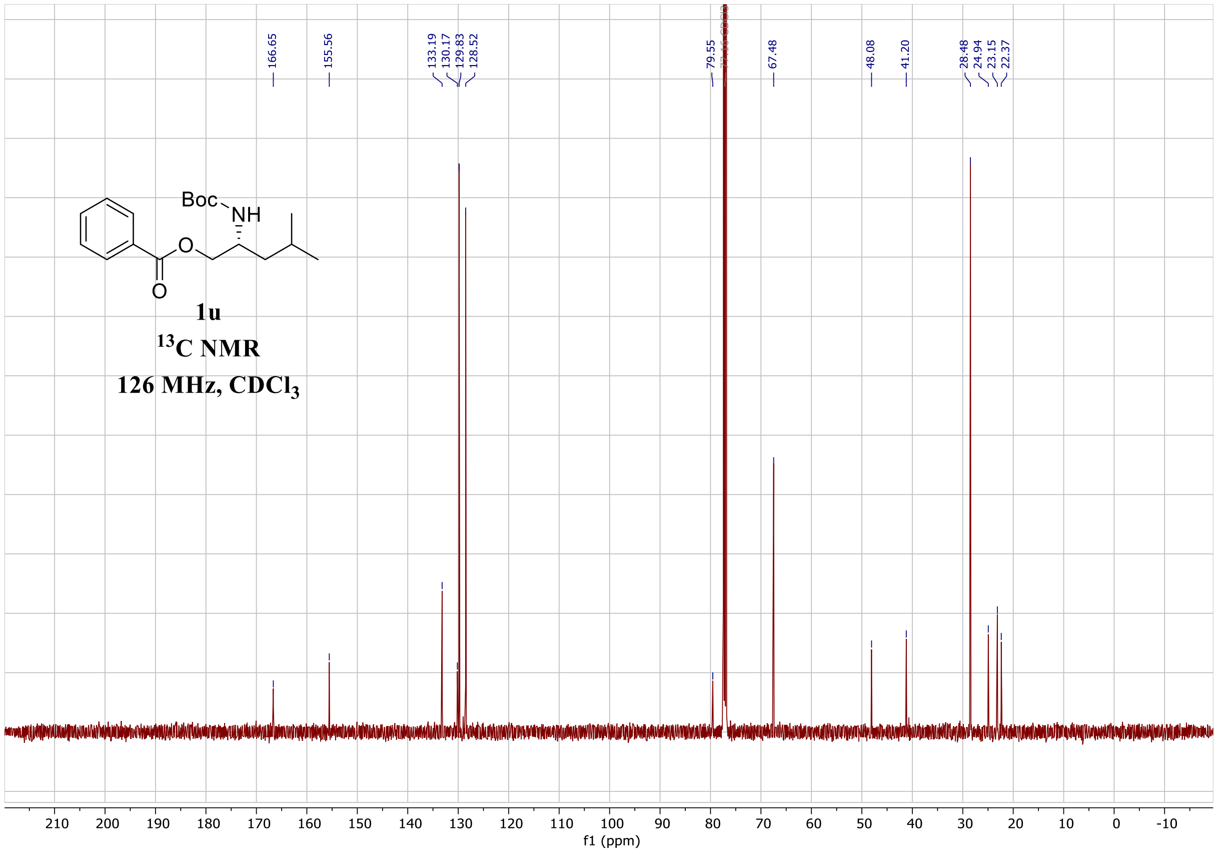
166.65
155.56
133.19
130.17
129.83
128.52
79.55
67.48
48.08
41.20
28.48
24.94
23.15
22.37
Boc
NH
O
O
1u
^{13}C NMR
126 MHz, $CDCl_3$
f1 (ppm)

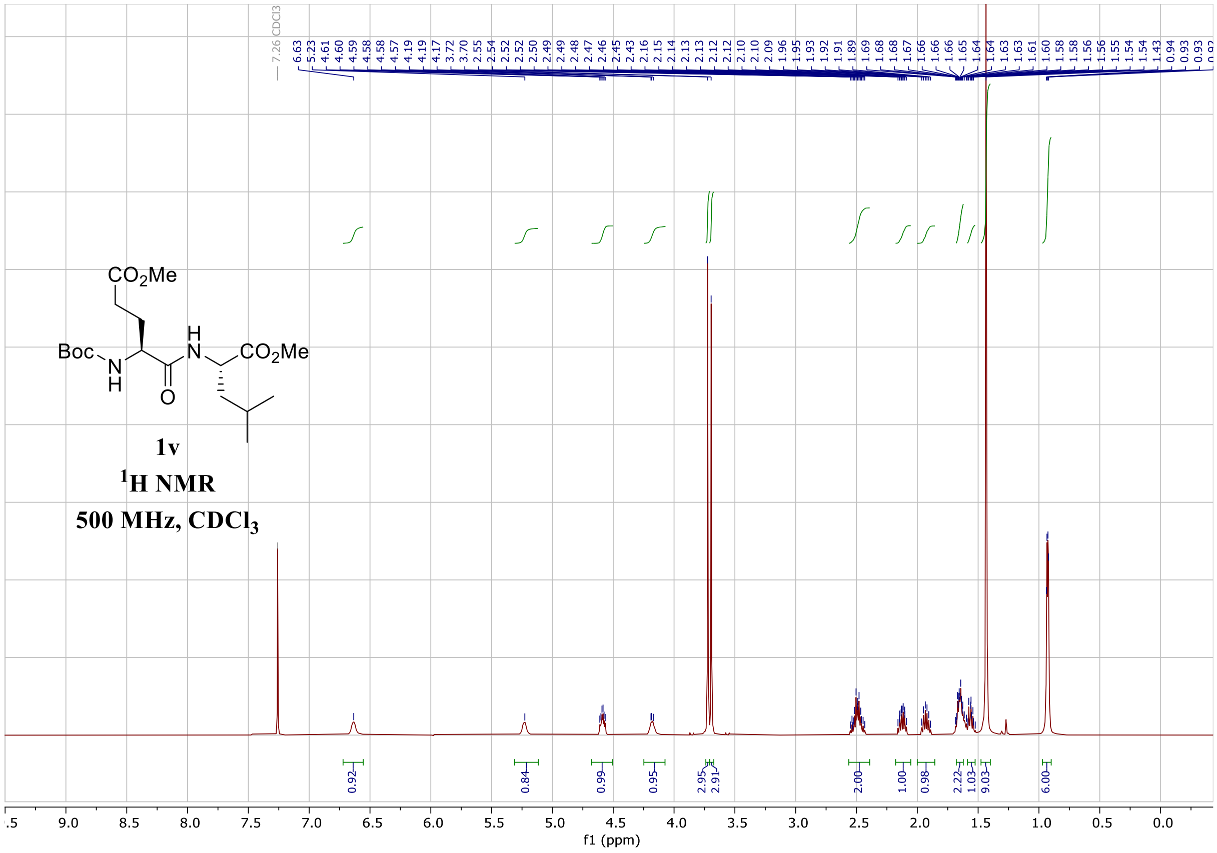
7.26 CDCl3
CO_2Me
Boc
N
H
H
N
CO_2Me
O
1v
^{1}H NMR
500 MHz, $CDCl_3$
0.92
0.84
0.99
0.95
2.95
2.91
2.00
1.00
0.98
2.22
1.03
9.03
6.00
f1 (ppm)

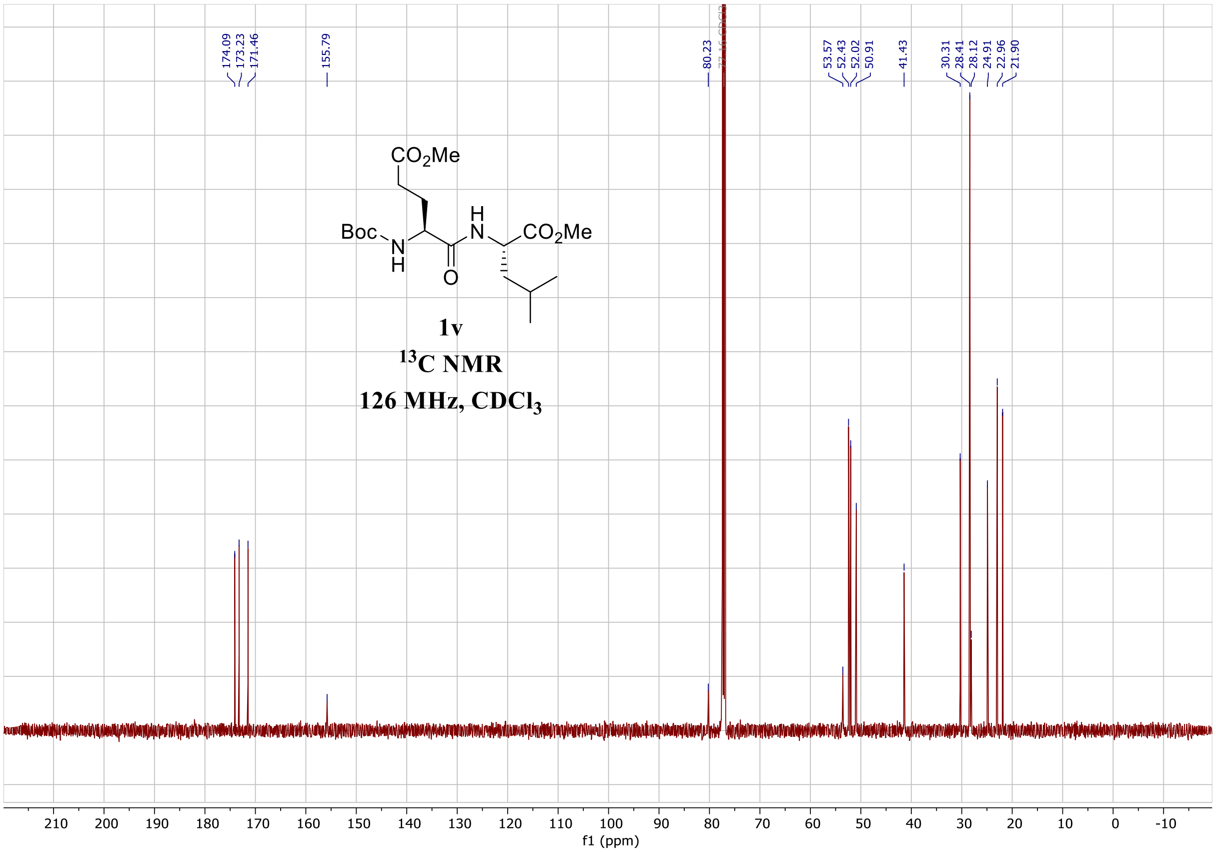

1v

^{13}C NMR

126 MHz, $CDCl_3$

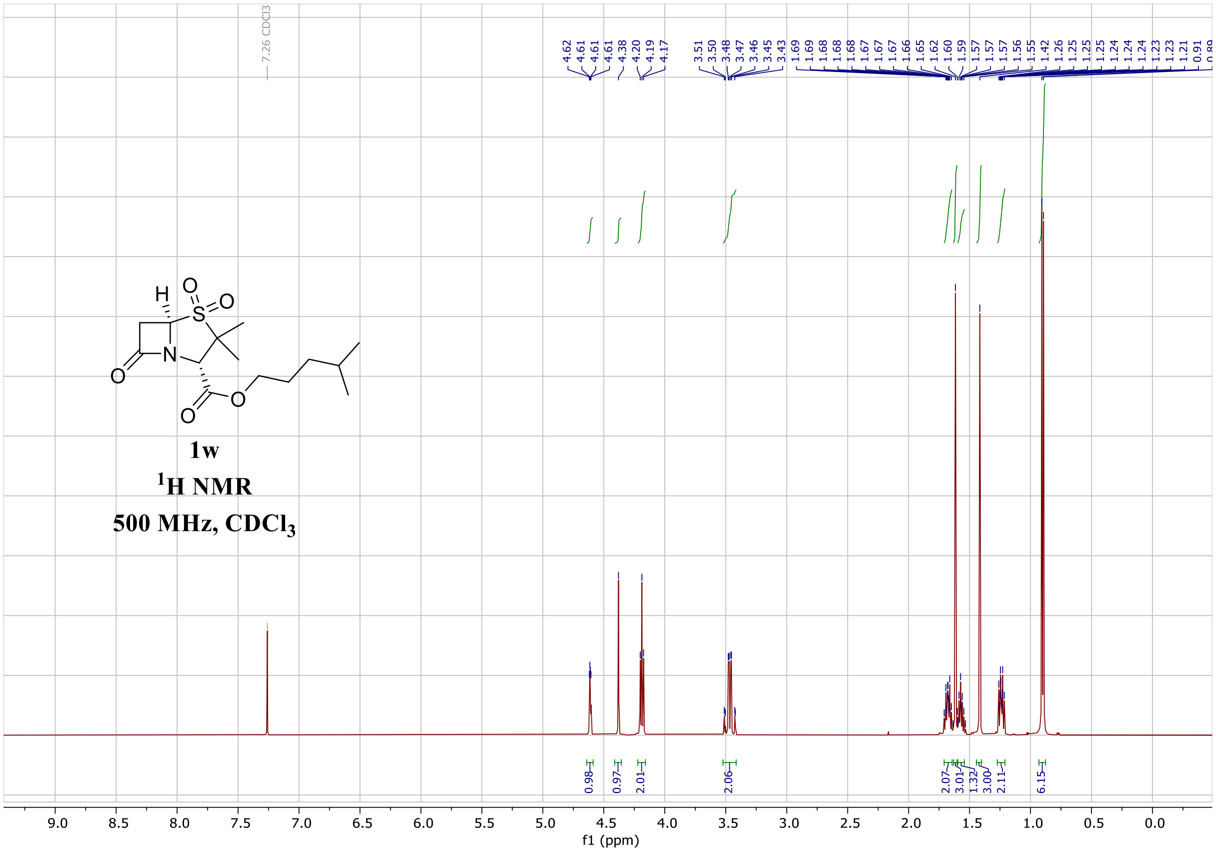

1w

^{1}H NMR

500 MHz, $CDCl_3$

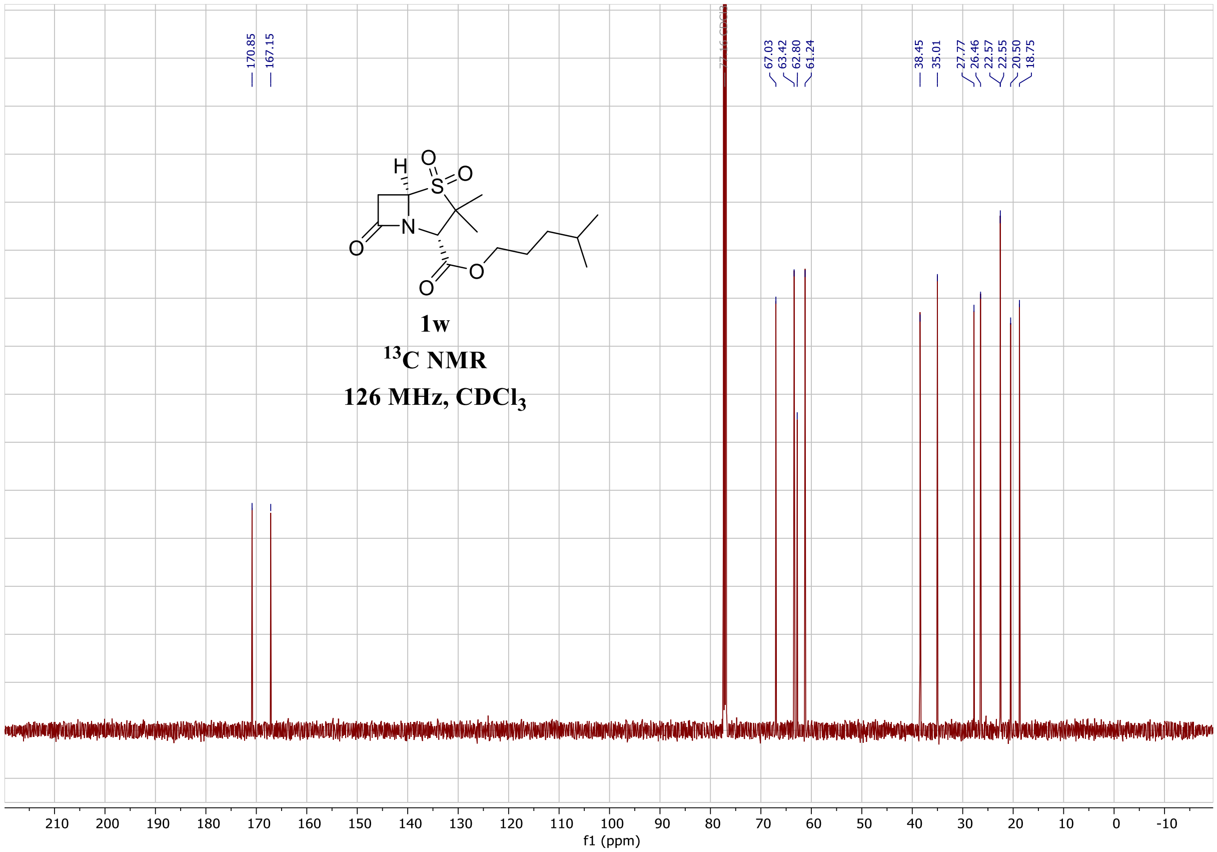

170.85
167.15
67.03
63.42
62.80
61.24
38.45
35.01
27.77
26.46
22.57
22.55
20.50
18.75
H
O
S
O
N
O
O
O
1w
^{13}C NMR
126 MHz, $CDCl_3$
210 200 190 180 170 160 150 140 130 120 110 100 90 80 70 60 50 40 30 20 10 0 -10
f1 (ppm)

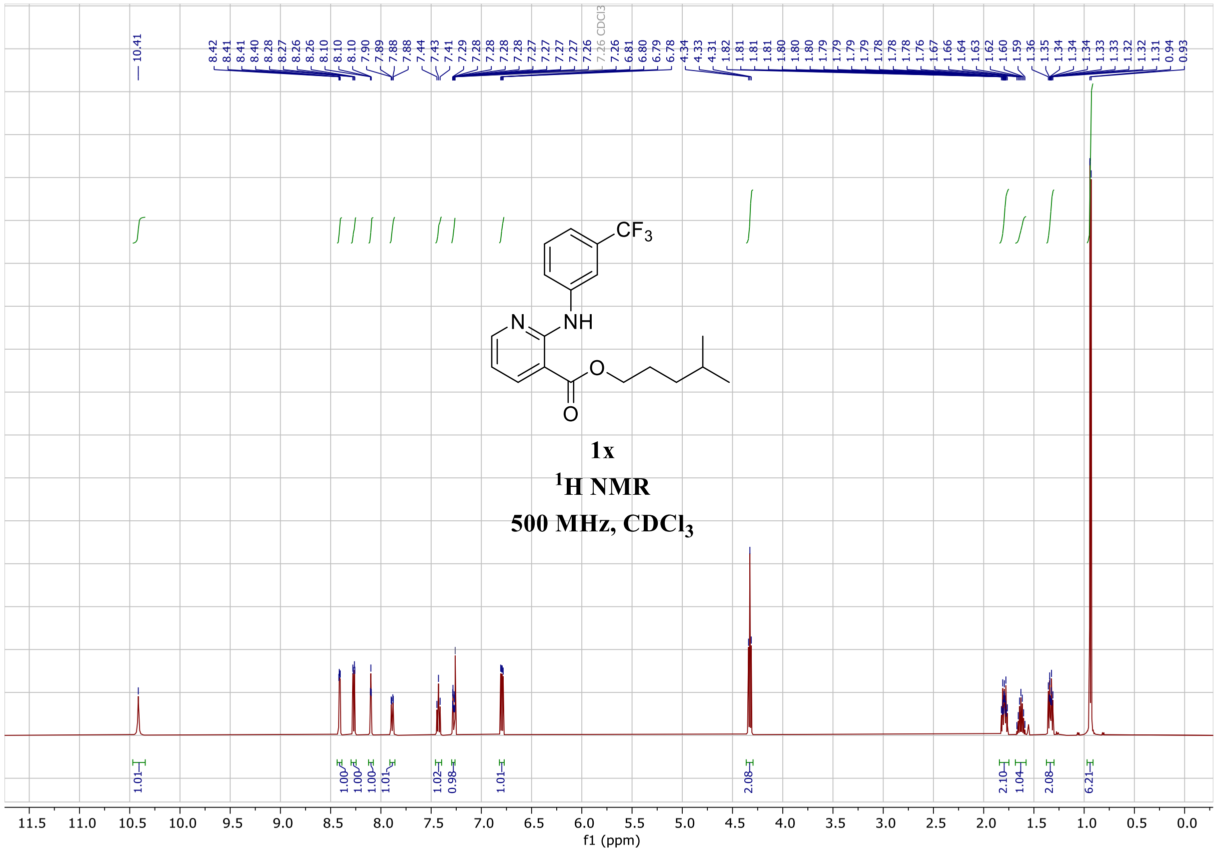

10.41
8.42 8.41 8.40 8.28 8.27 8.26 8.26 8.10 8.10 8.10 7.90 7.89 7.88 7.88 7.44 7.43 7.41 7.29 7.28 7.28 7.28 7.27 7.27 7.27 7.26 7.26 6.81 6.80 6.79 6.78
7.26 CDCl3
4.34 4.33 4.31 1.82 1.81 1.81 1.81 1.80 1.80 1.79 1.79 1.79 1.78 1.78 1.78 1.76 1.67 1.66 1.64 1.63 1.62 1.60 1.59 1.36 1.35 1.34 1.34 1.34 1.33 1.33 1.32 1.32 1.31 0.94 0.93
CF3
N
NH
O
O
1x
^{1}H NMR
500 MHz, $CDCl_3$
1.01 1.00 1.00 1.00 1.01 1.02 0.98 1.01 2.08 2.10 1.04 2.08 6.21
11.5 11.0 10.5 10.0 9.5 9.0 8.5 8.0 7.5 7.0 6.5 6.0 5.5 5.0 4.5 4.0 3.5 3.0 2.5 2.0 1.5 1.0 0.5 0.0
f1 (ppm)

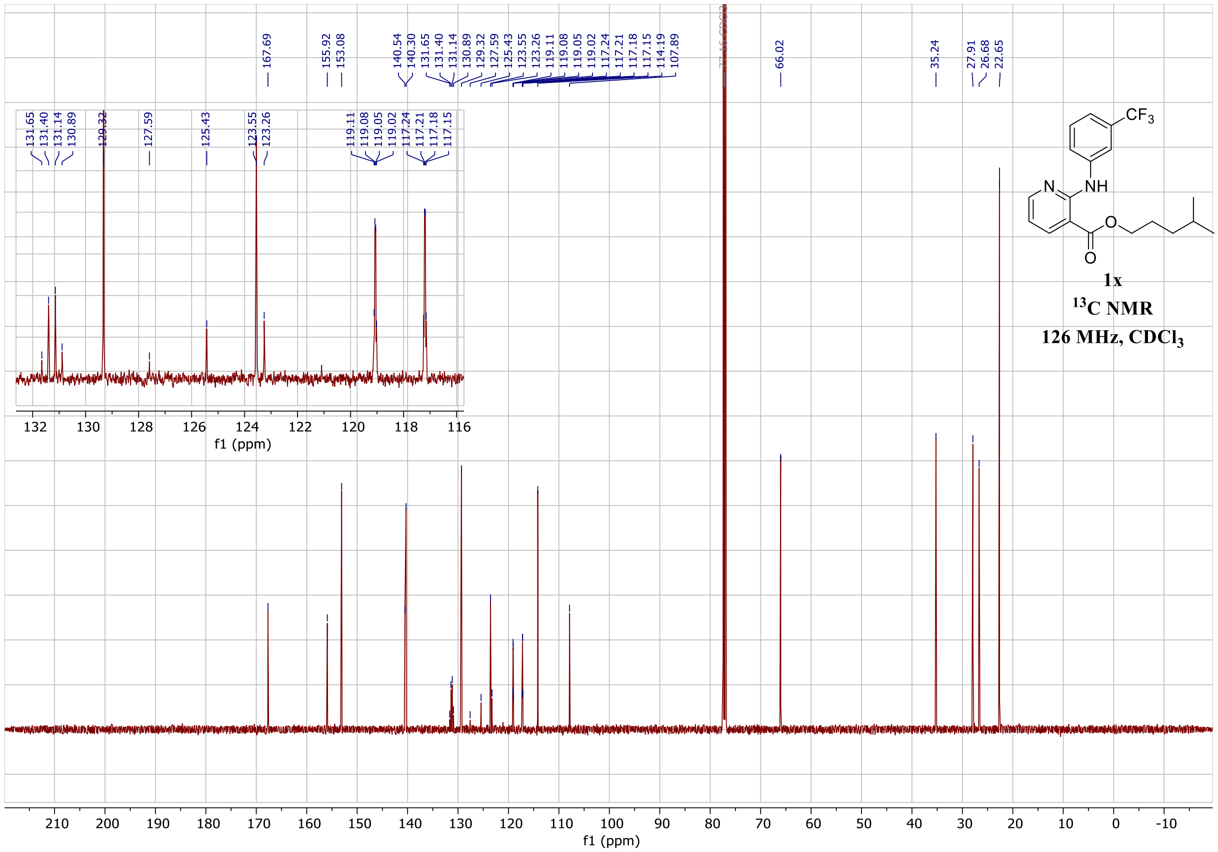
1x
^{13}C NMR
126 MHz, CDCl$_3$
f1 (ppm)

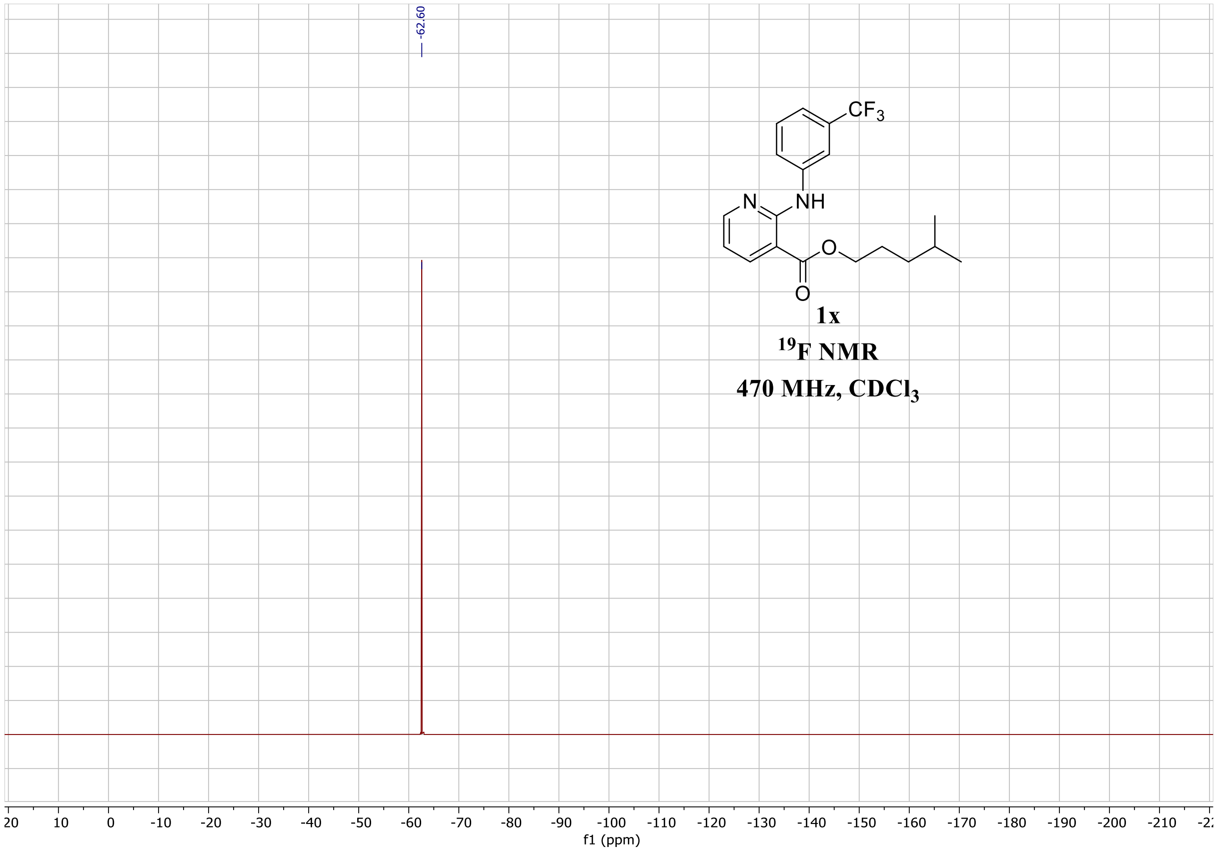
-62.60
1x
^{19}F NMR
470 MHz, CDCl$_3$
f1 (ppm)

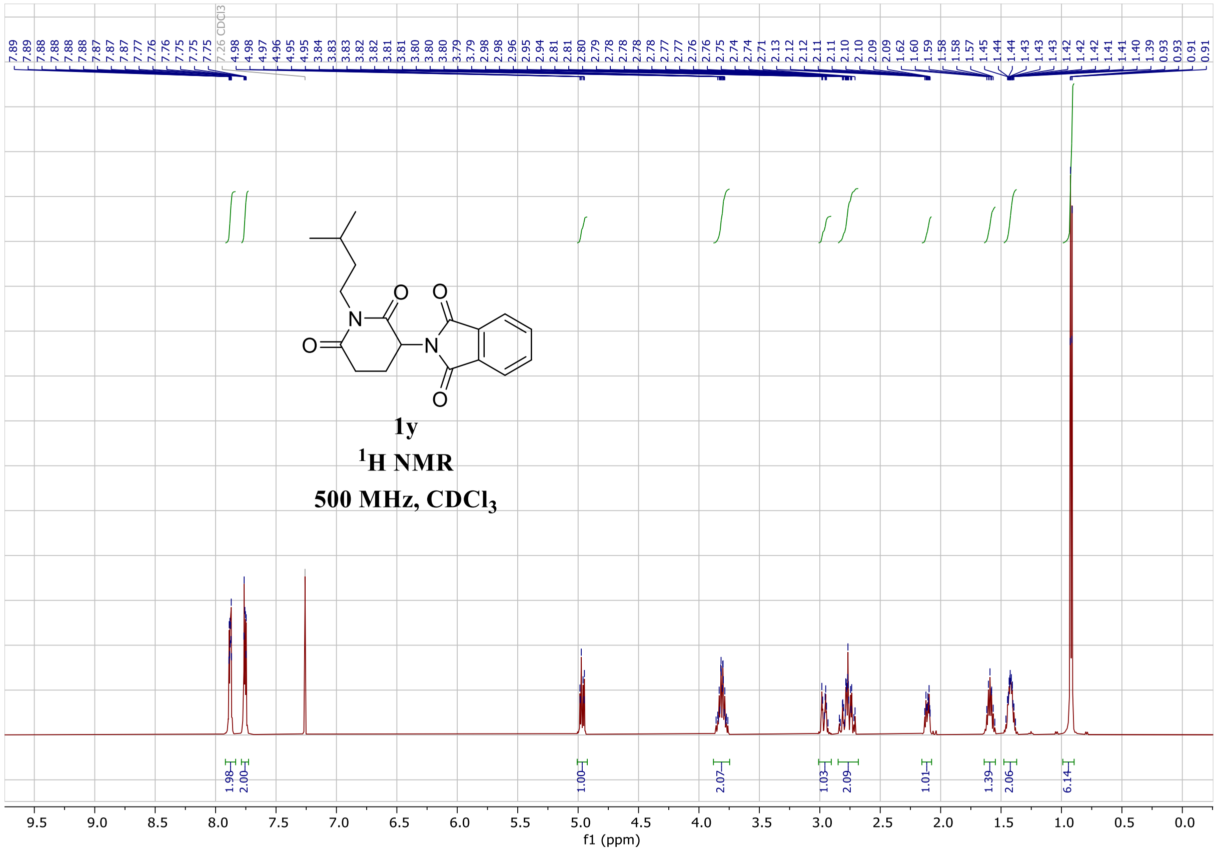

1y
^{1}H NMR
500 MHz, $CDCl_3$

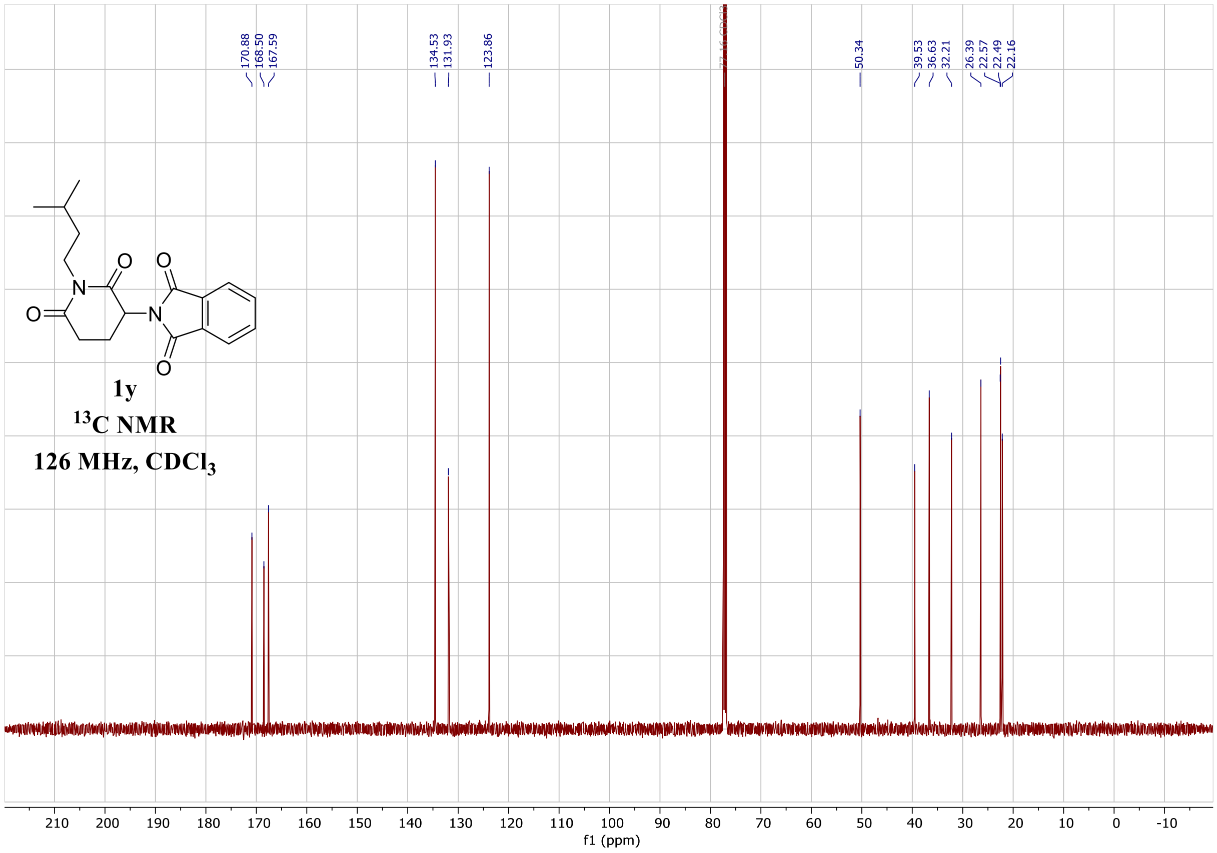

1y
^{13}C NMR
126 MHz, $CDCl_3$

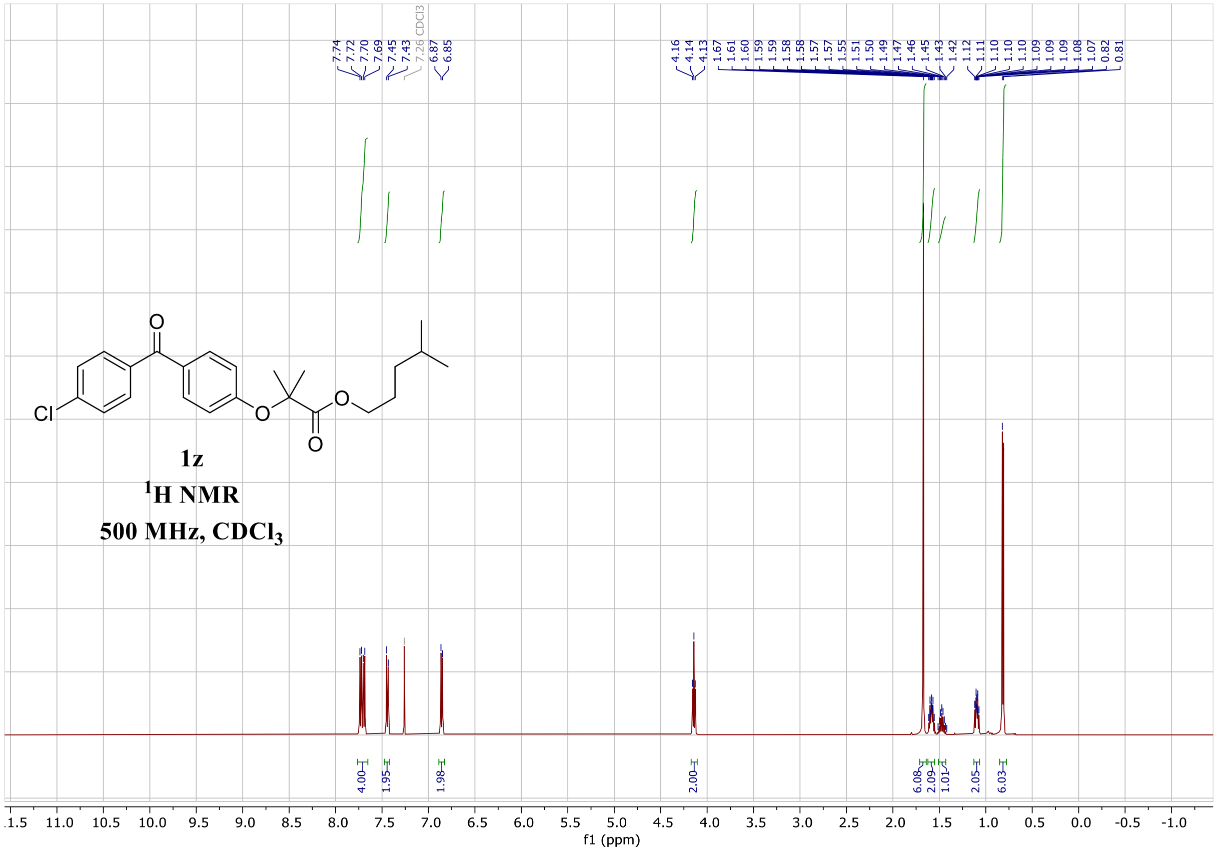
1z
1H NMR
500 MHz, CDCl3
f1 (ppm)

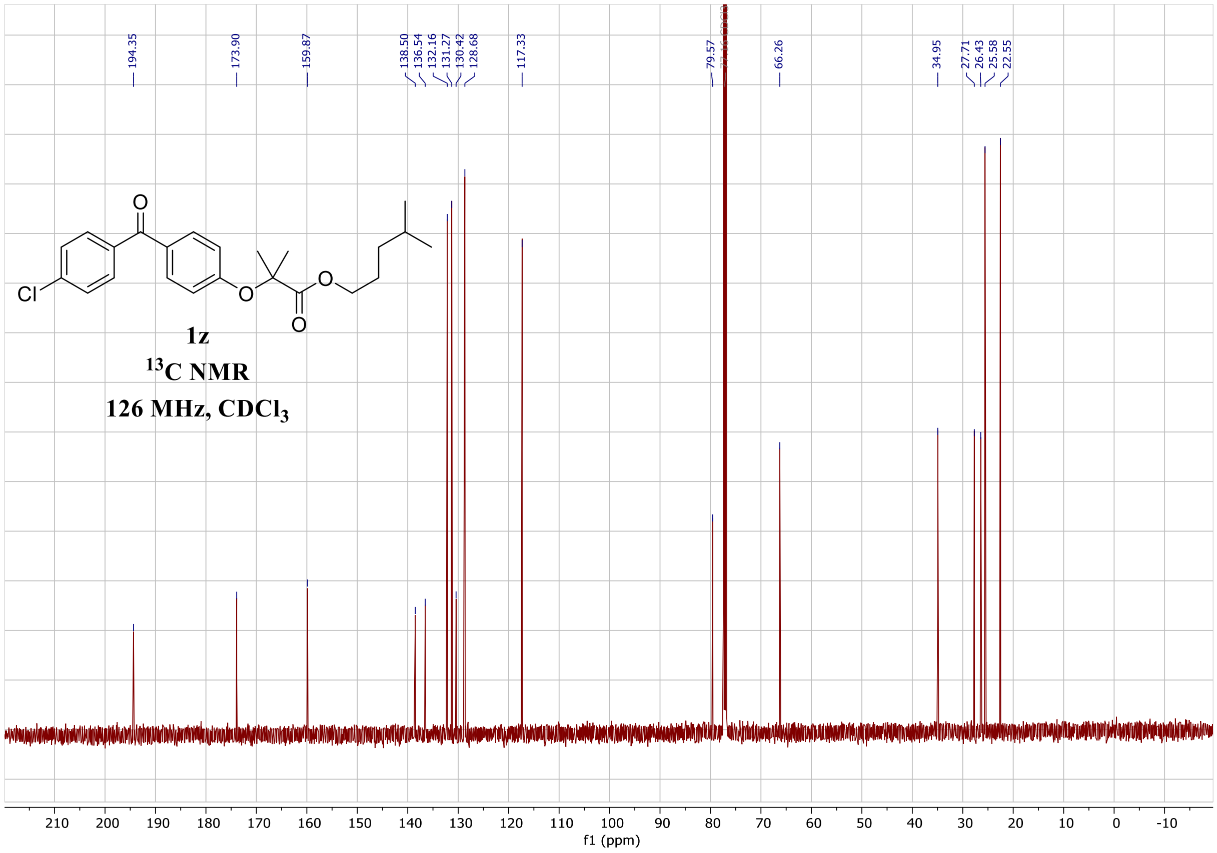
1z
13C NMR
126 MHz, CDCl3
f1 (ppm)

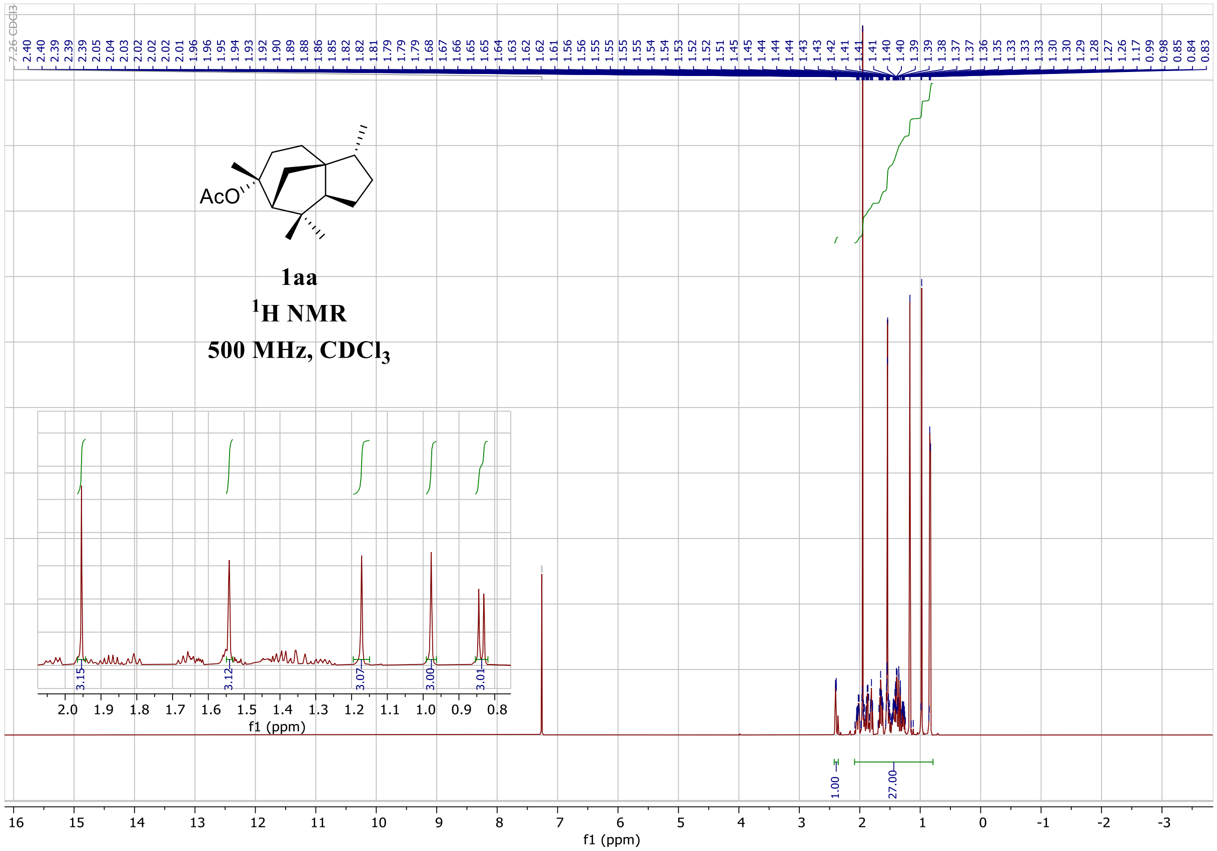
7.26 CDCl3
2.40 2.40 2.39 2.39 2.39 2.05 2.04 2.03 2.02 2.02 2.02 2.01 1.96 1.95 1.94 1.93 1.92 1.90 1.89 1.88 1.86 1.85 1.82 1.82 1.81 1.79 1.79 1.79 1.68 1.67 1.66 1.65 1.65 1.64 1.63 1.62 1.62 1.61 1.56 1.56 1.55 1.55 1.55 1.55 1.54 1.54 1.53 1.52 1.52 1.51 1.45 1.45 1.44 1.44 1.43 1.43 1.42 1.41 1.41 1.40 1.40 1.39 1.39 1.38 1.37 1.37 1.36 1.35 1.33 1.33 1.33 1.30 1.29 1.28 1.27 1.26 1.17 0.99 0.98 0.85 0.84 0.83
AcO
1aa
1H NMR
500 MHz, CDCl3
3.15
3.12
3.07
3.00
3.01
2.0 1.9 1.8 1.7 1.6 1.5 1.4 1.3 1.2 1.1 1.0 0.9 0.8
f1 (ppm)
1.00
27.00
16 15 14 13 12 11 10 9 8 7 6 5 4 3 2 1 0 -1 -2 -3
f1 (ppm)

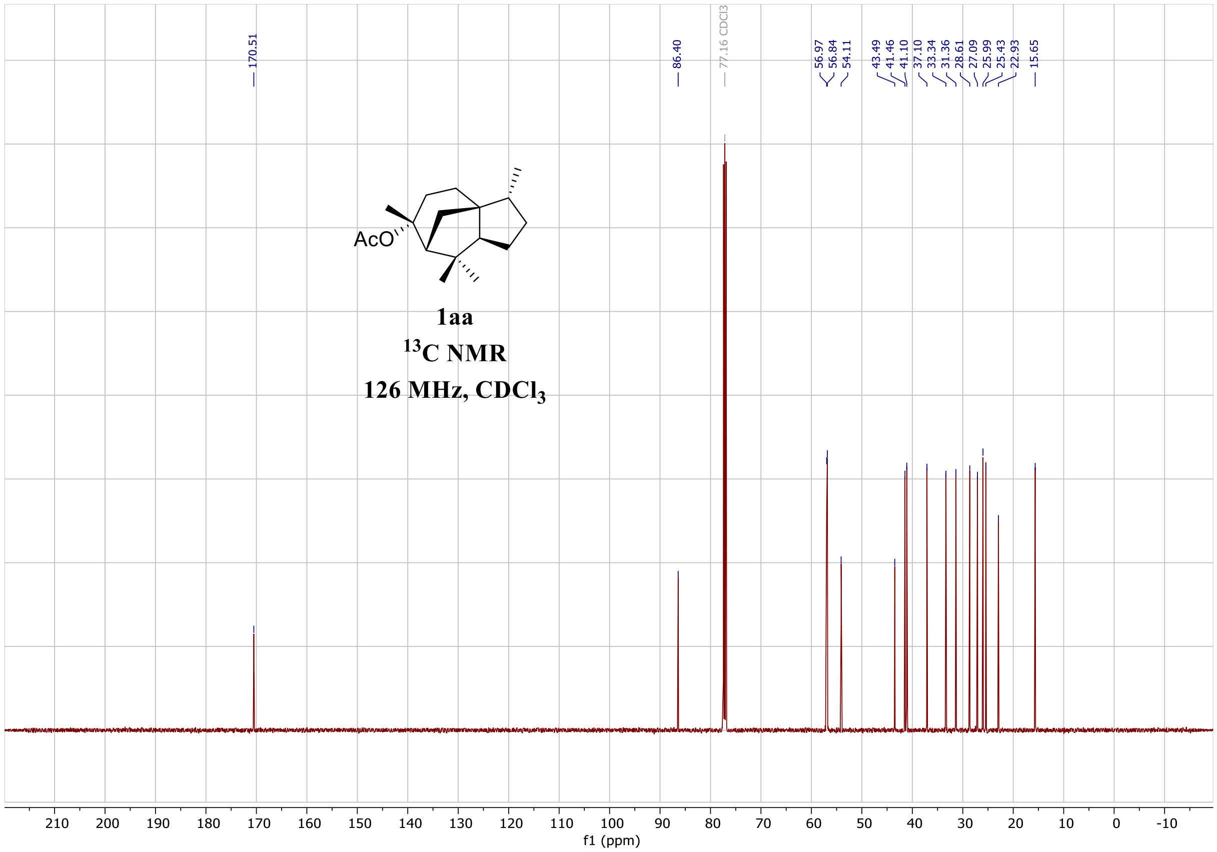
170.51
86.40
77.16 CDCl3
56.97 56.84 54.11
43.49 41.46 41.10 37.10 33.34 31.36 28.61 27.09 25.99 25.43 22.93
15.65
AcO
1aa
13C NMR
126 MHz, CDCl3
210 200 190 180 170 160 150 140 130 120 110 100 90 80 70 60 50 40 30 20 10 0 -10
f1 (ppm)

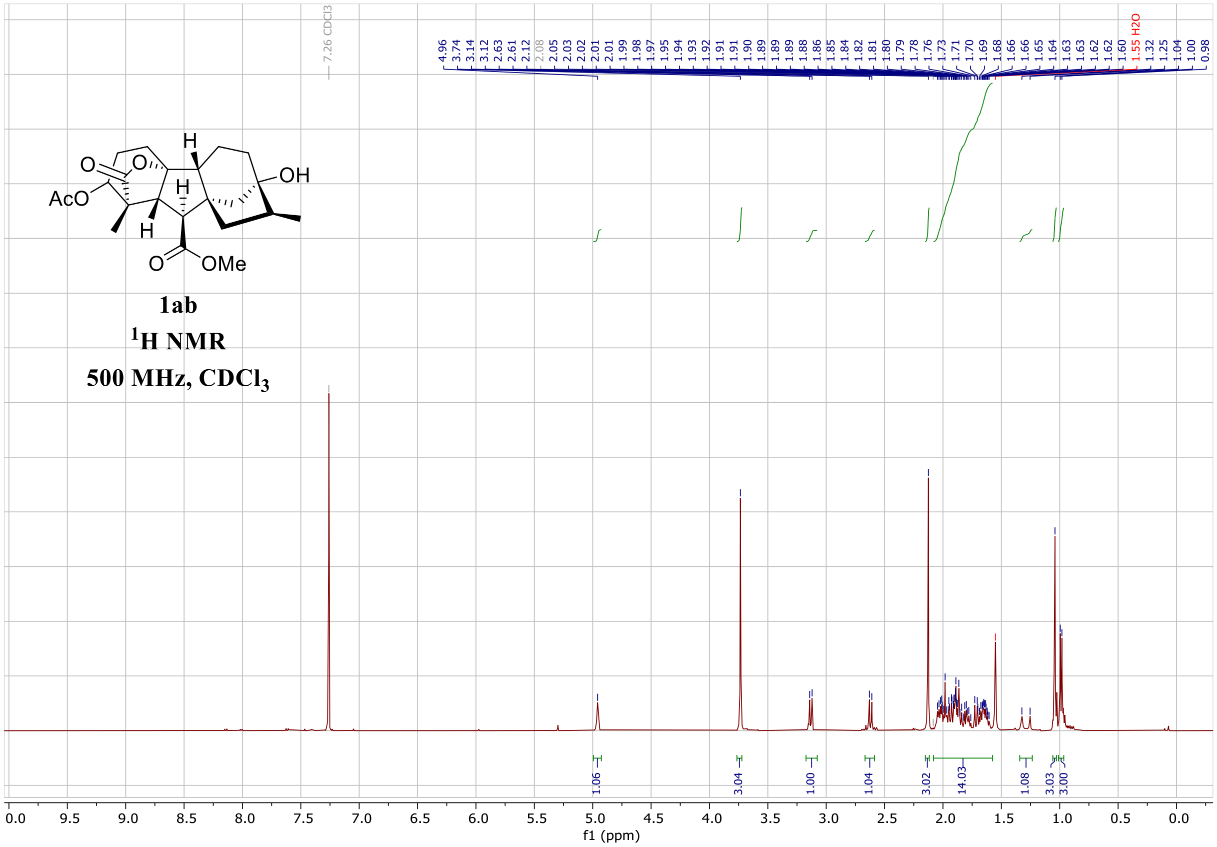
1ab
^{1}H NMR
500 MHz, $CDCl_3$
7.26 CDCl3
1.55 H2O
f1 (ppm)

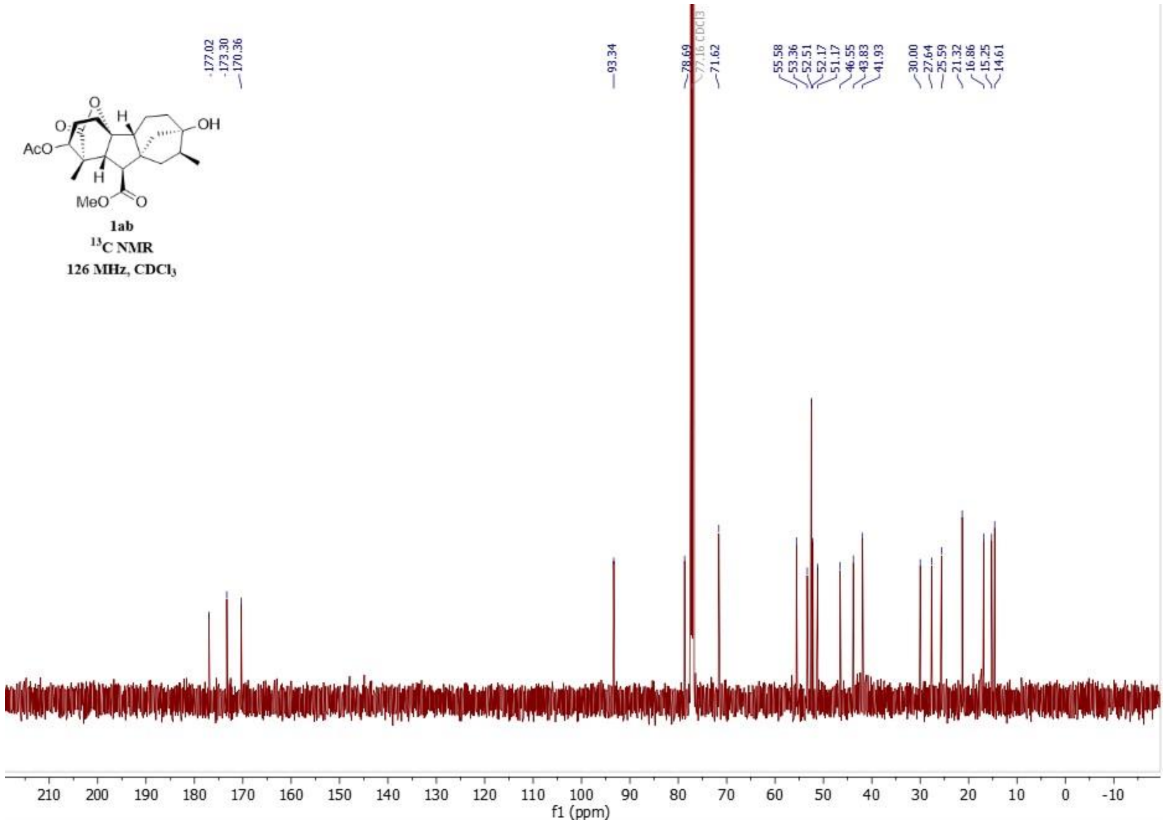
1ab
^{13}C NMR
126 MHz, $CDCl_3$
177.02
173.30
170.36
93.34
78.69
77.16 CDCl3
71.62
55.58
53.36
52.51
52.17
51.17
46.55
43.83
41.93
30.00
27.64
25.59
21.32
16.86
15.25
14.61
f1 (ppm)

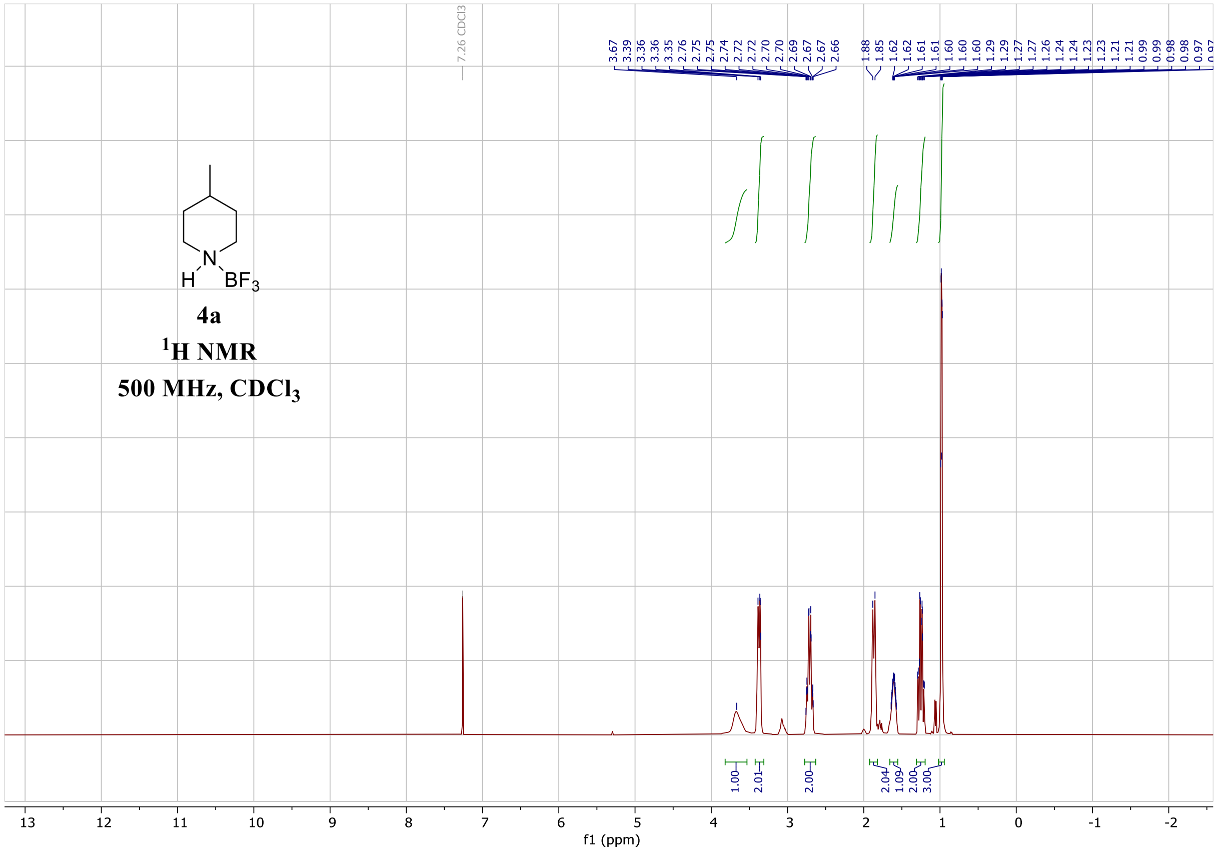
7.26 CDCl3
3.67
3.39
3.36
3.36
3.35
2.76
2.75
2.75
2.74
2.72
2.72
2.70
2.69
2.67
2.67
2.66
1.88
1.85
1.62
1.62
1.61
1.61
1.60
1.60
1.60
1.29
1.29
1.27
1.26
1.24
1.24
1.23
1.21
1.21
0.99
0.99
0.98
0.98
0.97
H
N
BF3
4a
1H NMR
500 MHz, CDCl3
1.00
2.01
2.00
2.04
1.09
2.00
3.00
f1 (ppm)

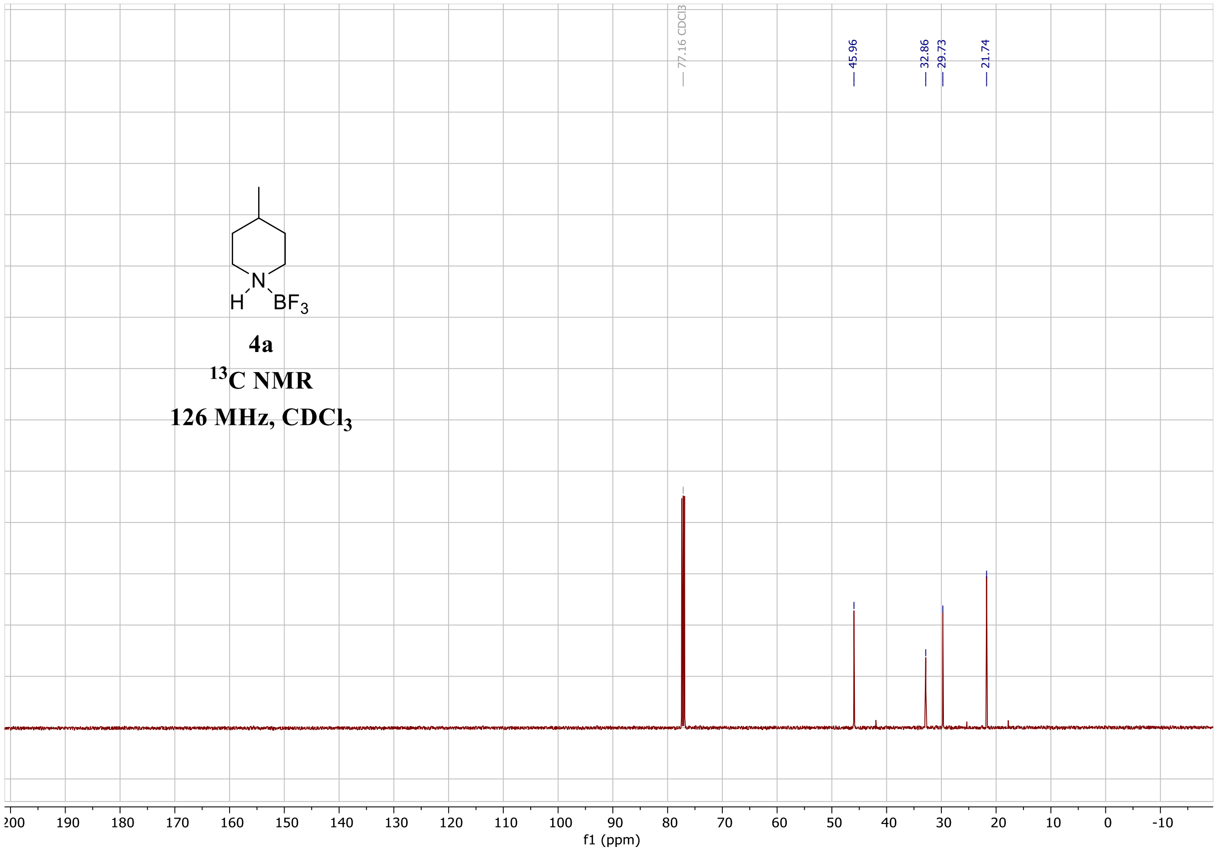
77.16 CDCl3
45.96
32.86
29.73
21.74
H
N
BF3
4a
13C NMR
126 MHz, CDCl3
f1 (ppm)

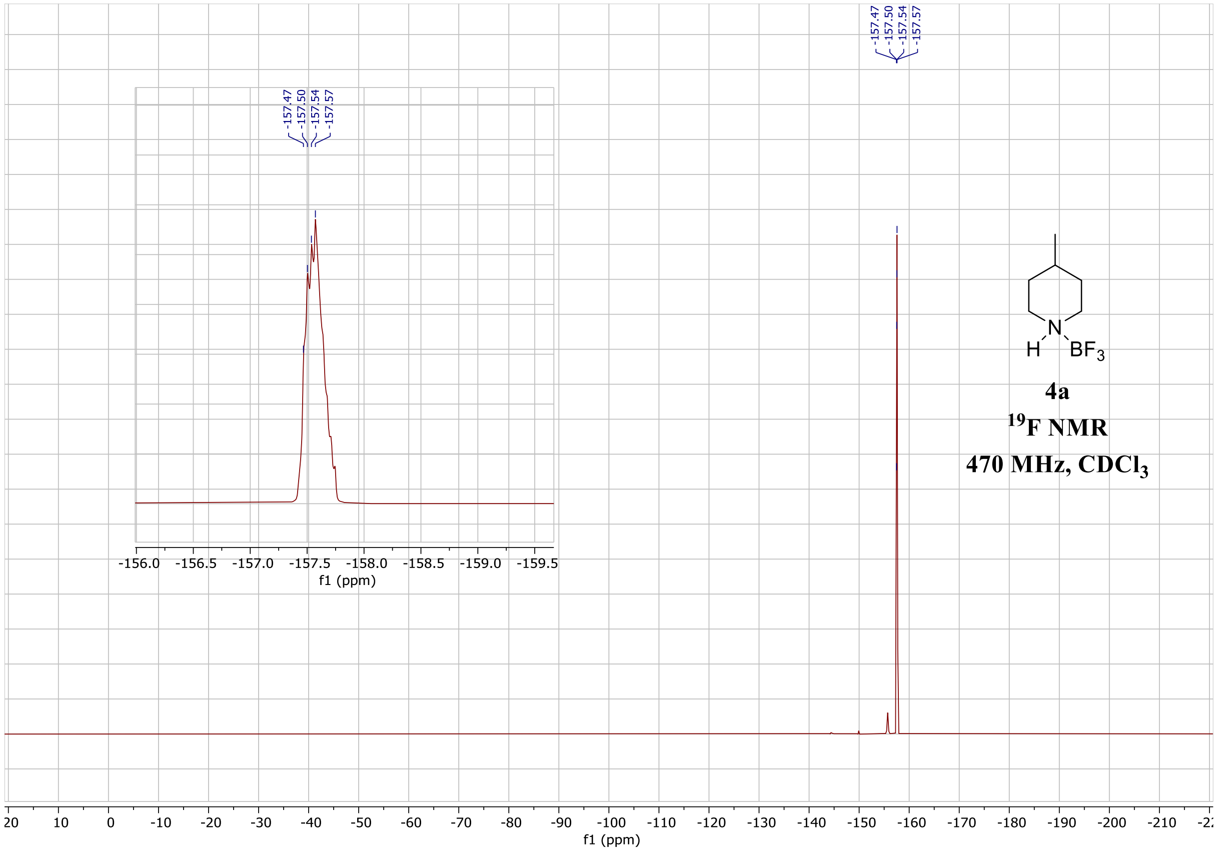

4a
^{19}F NMR
470 MHz, $CDCl_3$

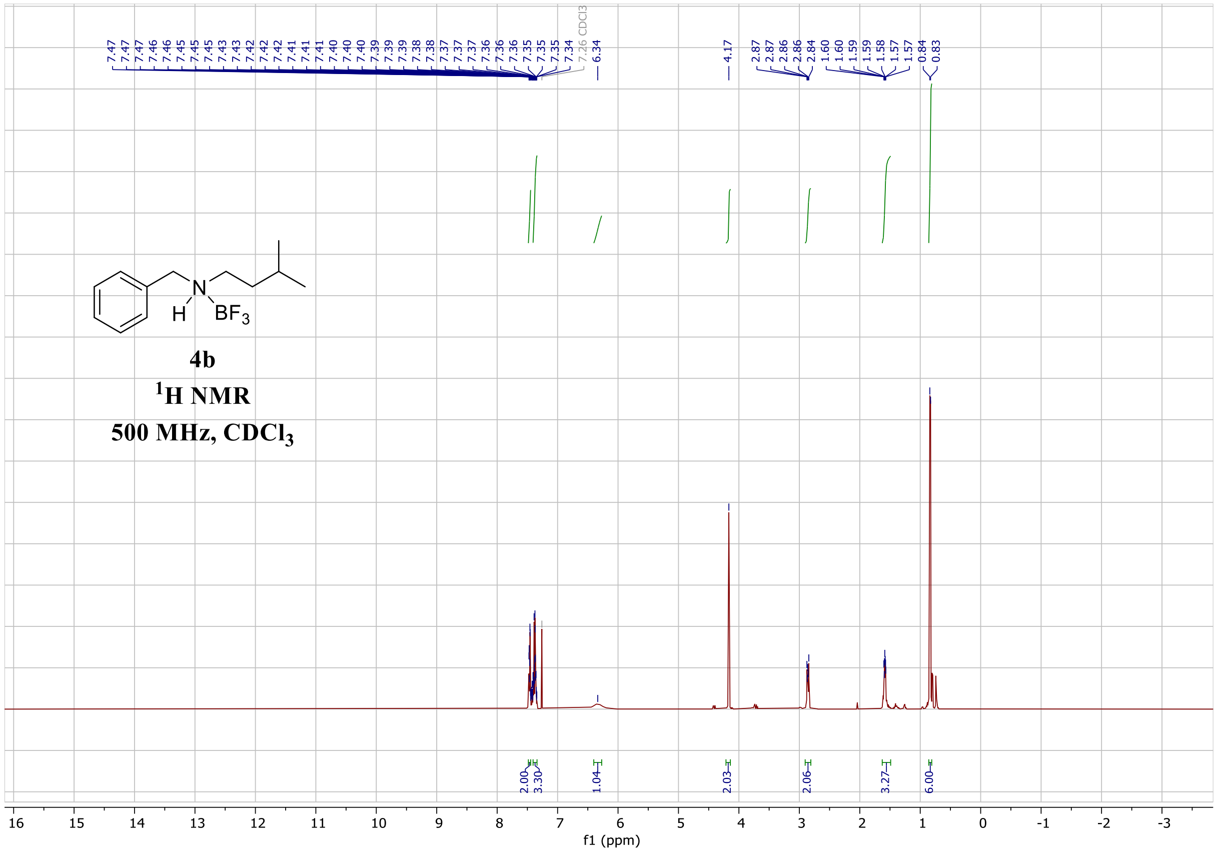

4b
^{1}H NMR
500 MHz, $CDCl_3$

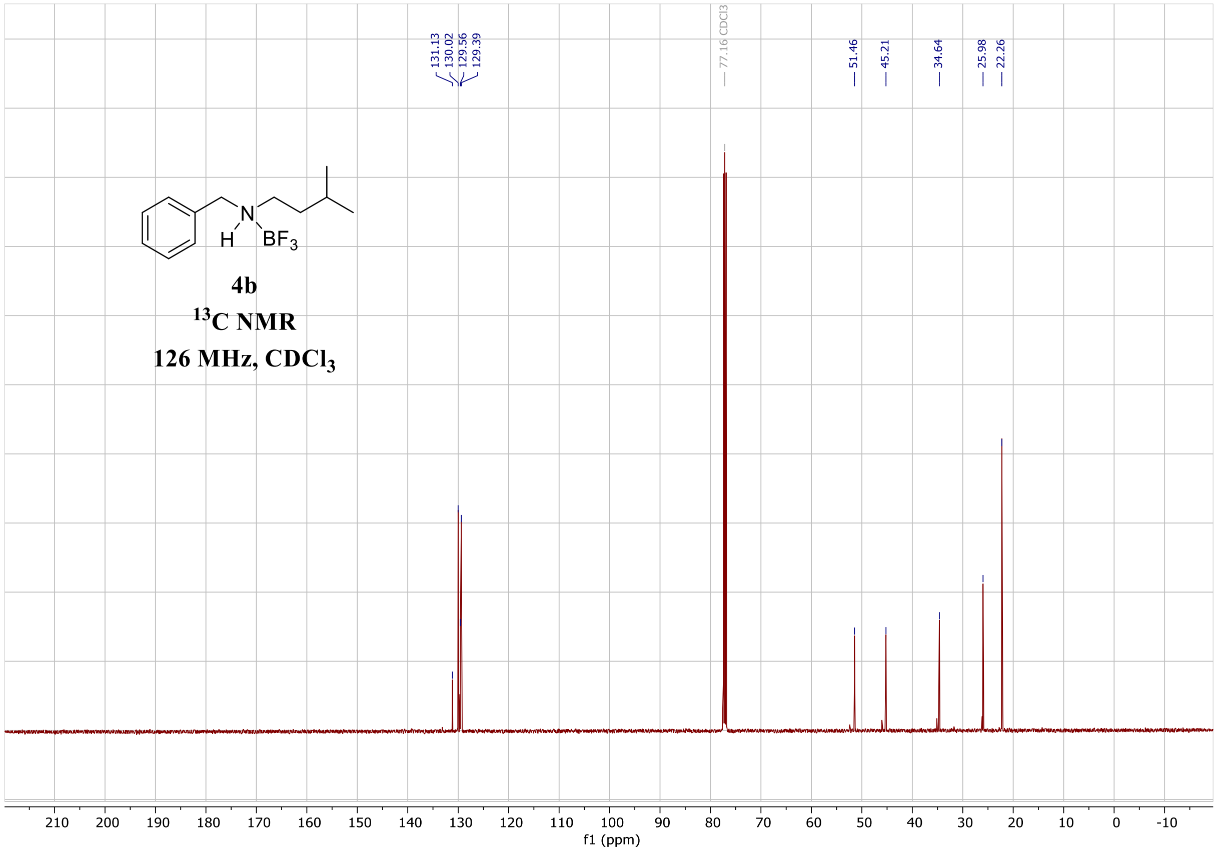
131.13
130.02
129.56
129.39
77.16 CDCl3
51.46
45.21
34.64
25.98
22.26
H
N
BF3
4b
13C NMR
126 MHz, CDCl3
f1 (ppm)

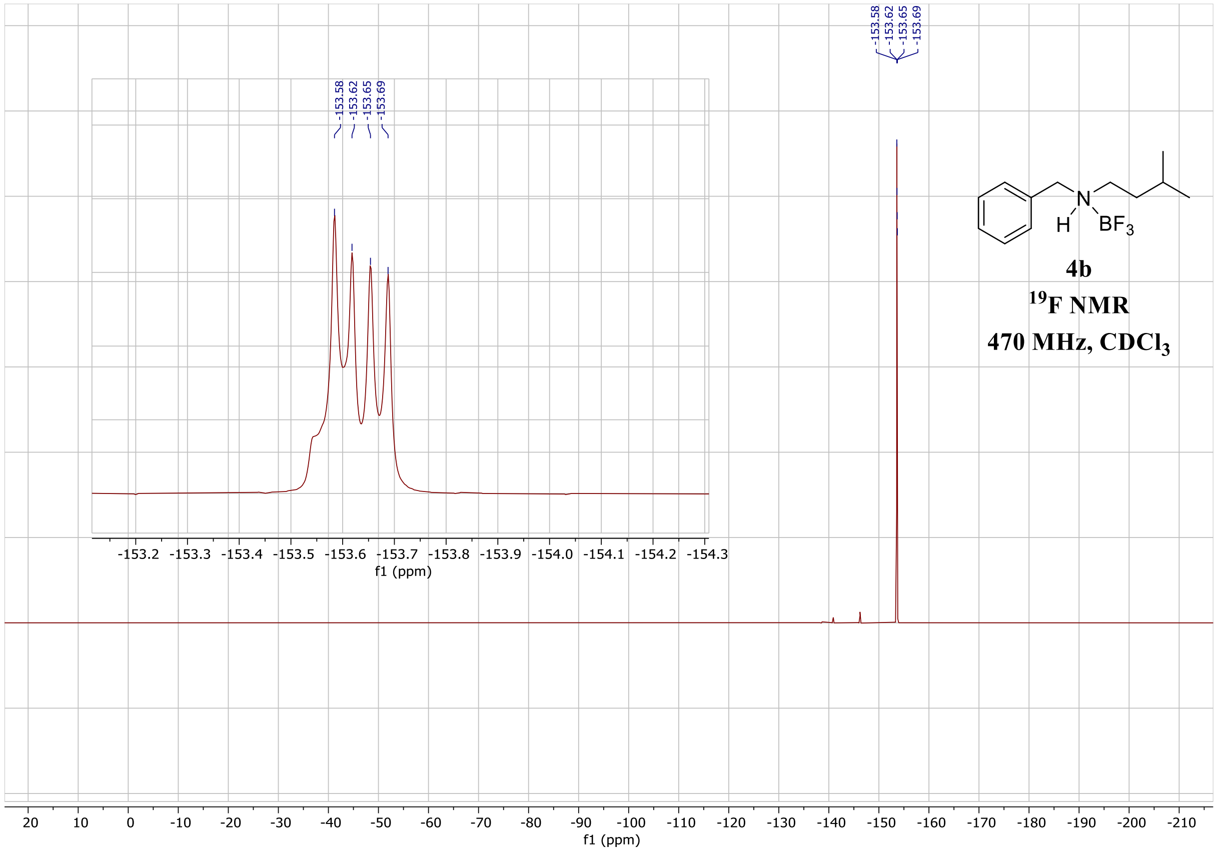
-153.58
-153.62
-153.65
-153.69
H
N
BF3
4b
19F NMR
470 MHz, CDCl3
f1 (ppm)

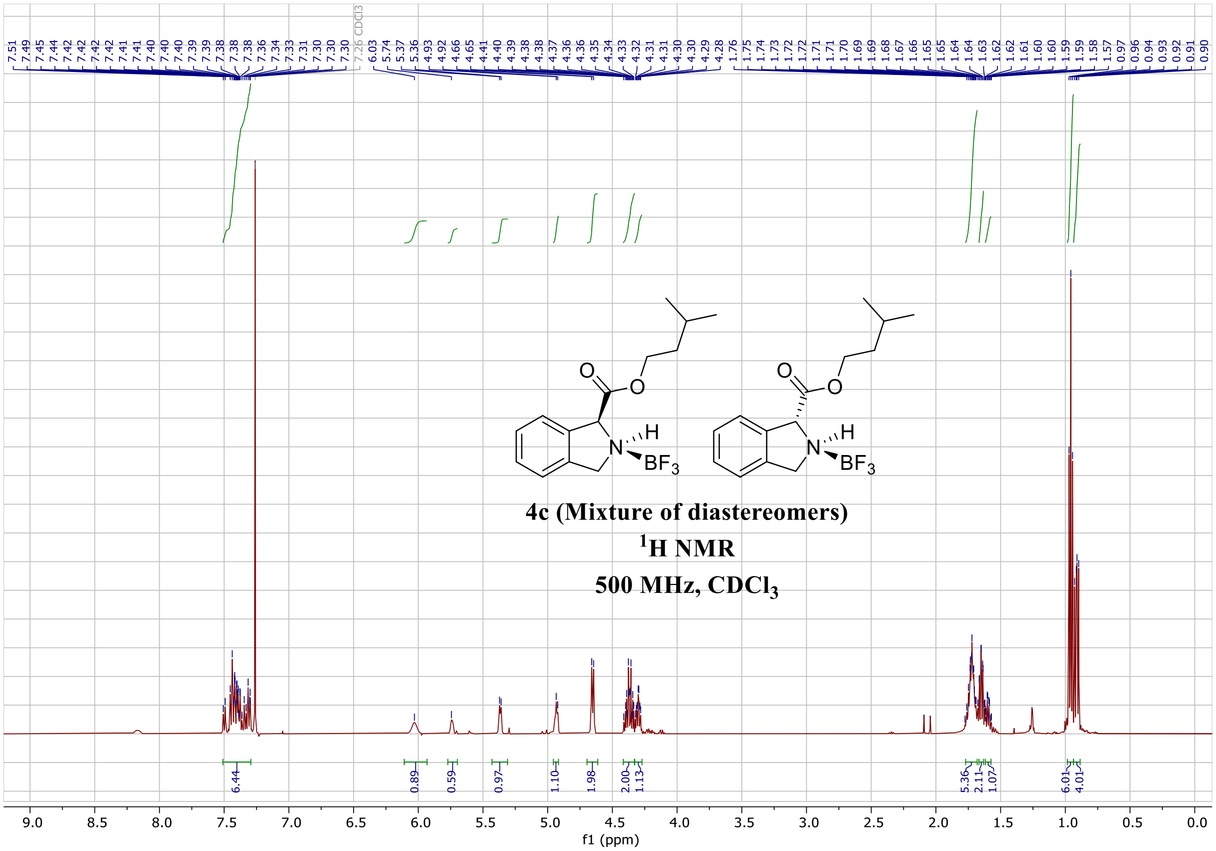

4c (Mixture of diastereomers)
^{1}H NMR
500 MHz, $CDCl_3$
7.51 7.49 7.45 7.44 7.42 7.42 7.42 7.42 7.41 7.41 7.40 7.40 7.40 7.39 7.39 7.38 7.38 7.38 7.36 7.34 7.33 7.31 7.30 7.30 7.30
7.26 CDCl3
6.03 5.74 5.37 5.36 4.93 4.92 4.66 4.65 4.41 4.40 4.39 4.38 4.38 4.37 4.36 4.36 4.35 4.34 4.33 4.32 4.31 4.31 4.30 4.30 4.29 4.28
1.76 1.75 1.74 1.73 1.72 1.72 1.71 1.71 1.70 1.69 1.69 1.68 1.67 1.66 1.65 1.65 1.64 1.64 1.63 1.63 1.62 1.62 1.61 1.60 1.60 1.59 1.59 1.59 1.58 1.57 0.97 0.96 0.94 0.93 0.92 0.91 0.90
6.44 0.89 0.59 0.97 1.10 1.98 2.00 1.13 5.36 2.11 1.07 6.01 4.01
f1 (ppm)

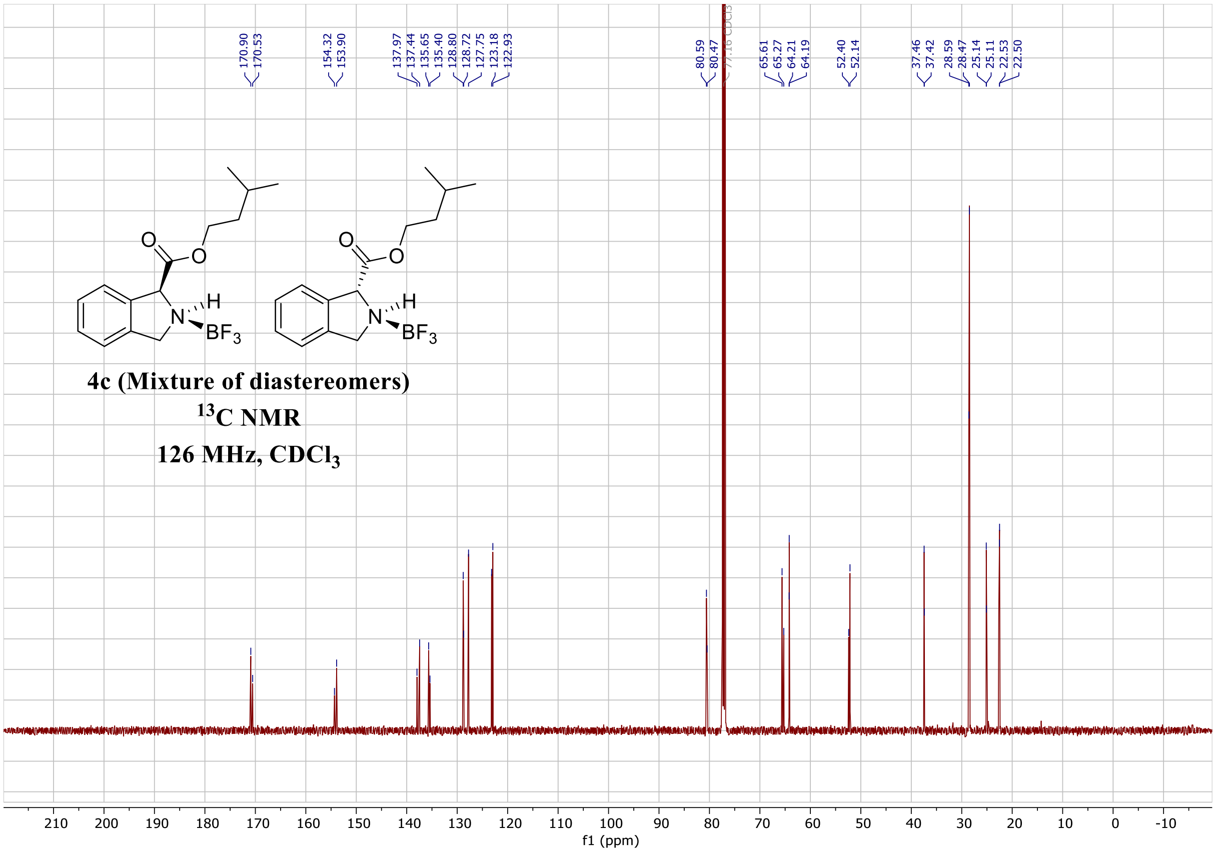

4c (Mixture of diastereomers)
^{13}C NMR
126 MHz, $CDCl_3$
170.90 170.53 154.32 153.90 137.97 137.44 135.65 135.40 128.80 128.72 127.75 123.18 122.93
80.59 80.47 77.16 CDCl3
65.61 65.27 64.21 64.19 52.40 52.14 37.46 37.42 28.59 28.47 25.14 25.11 22.53 22.50
f1 (ppm)

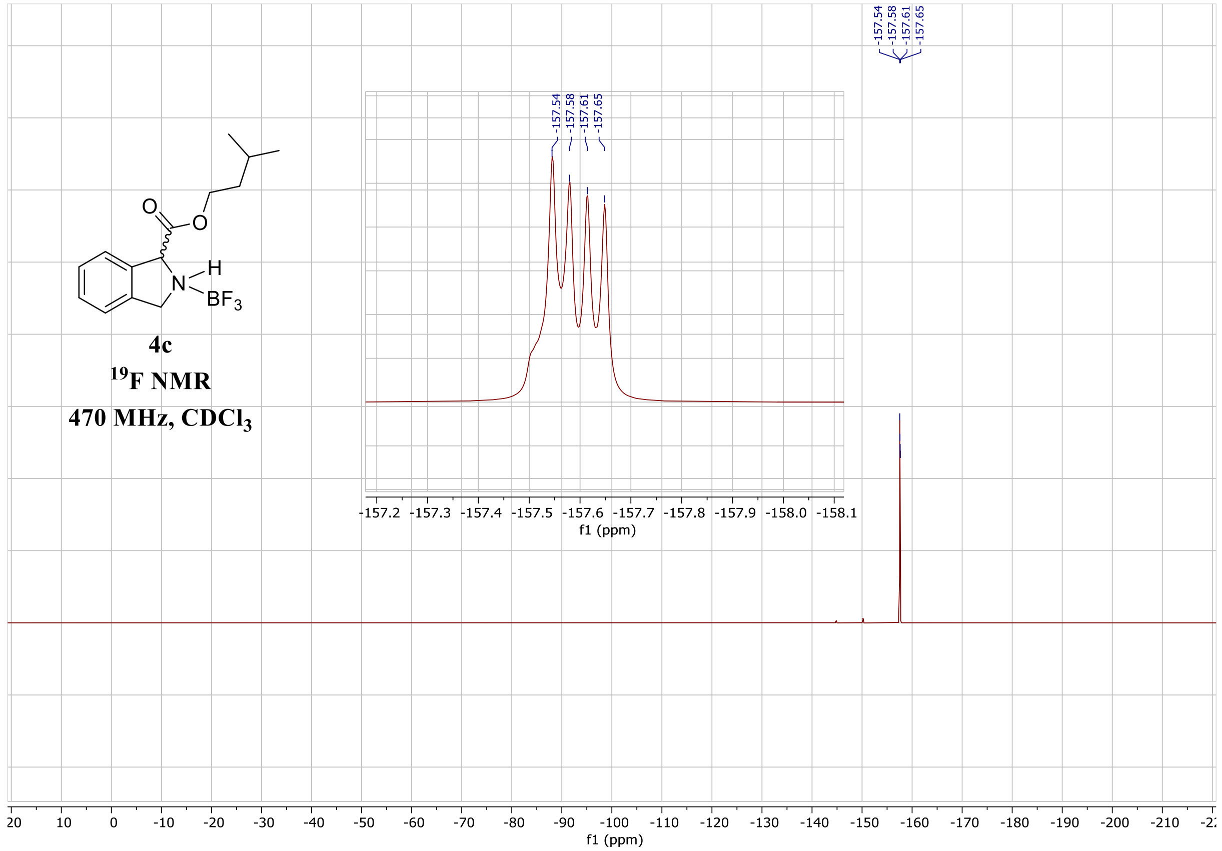
4c
^{19}F NMR
470 MHz, CDCl$_3$
f1 (ppm)

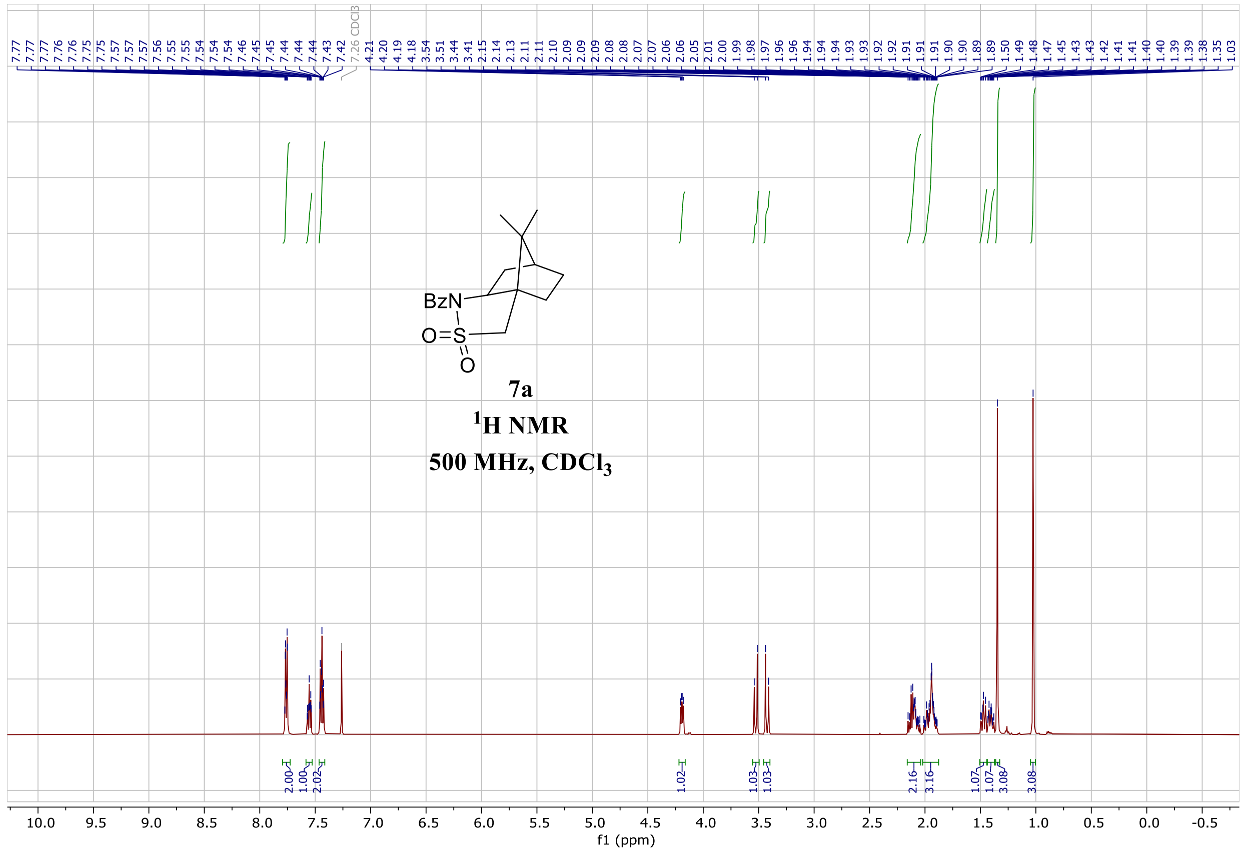
7a
^{1}H NMR
500 MHz, CDCl$_3$
f1 (ppm)

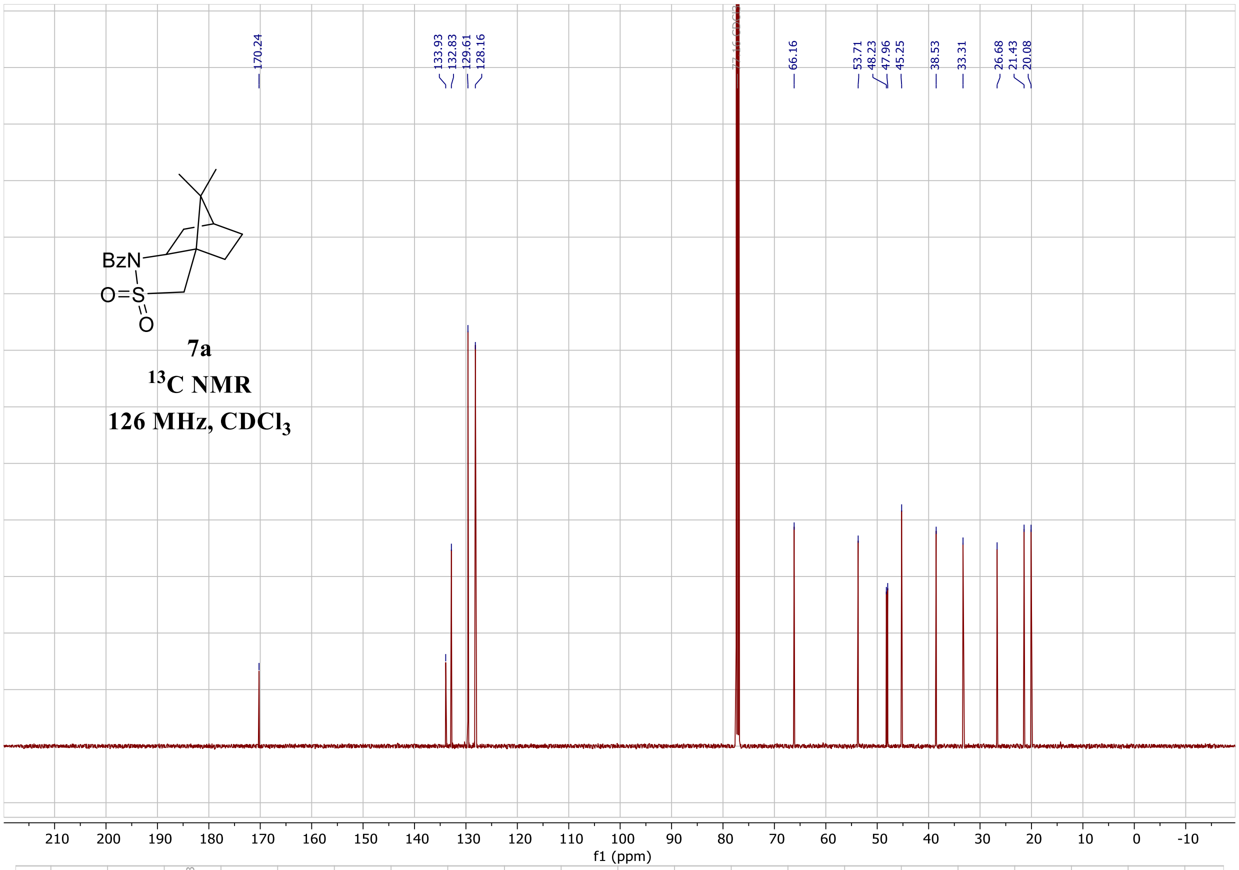

7a

^{13}C NMR

126 MHz, $CDCl_3$

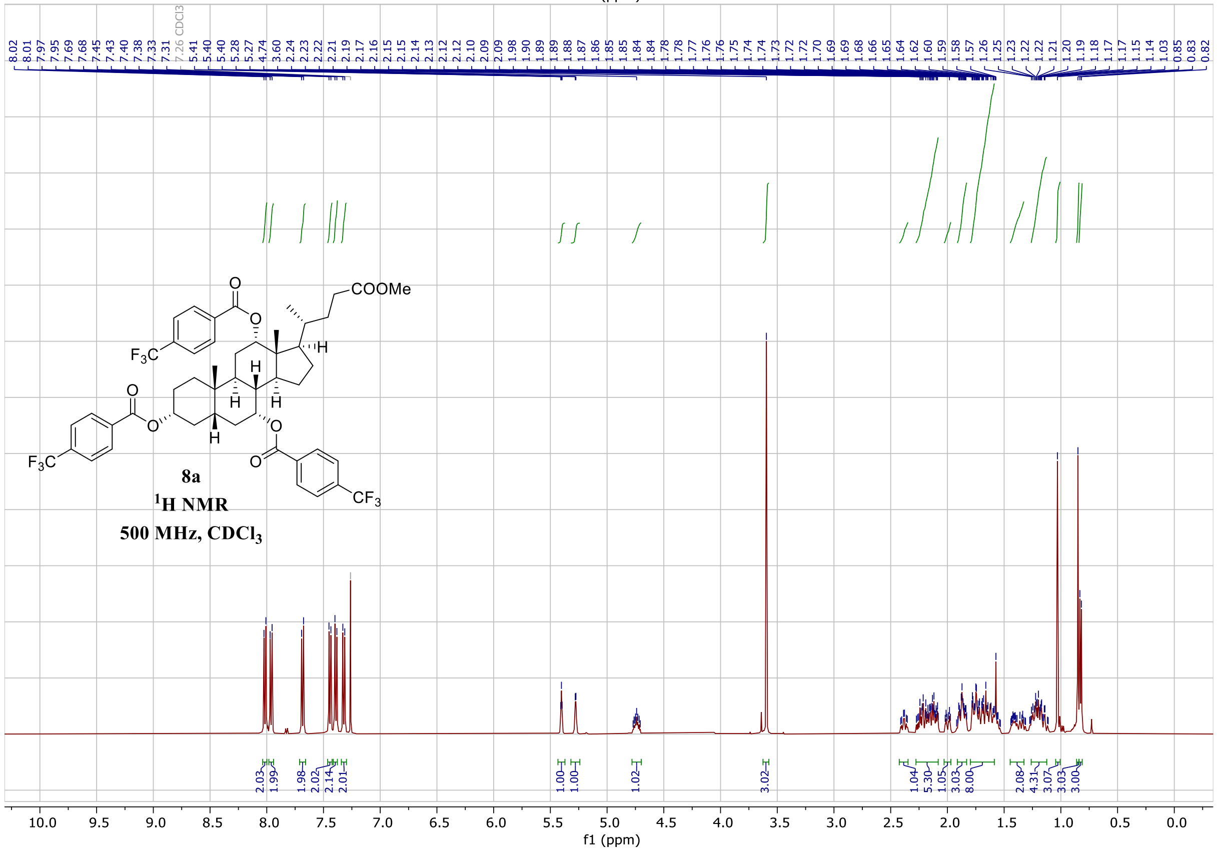

8a

^{1}H NMR

500 MHz, $CDCl_3$

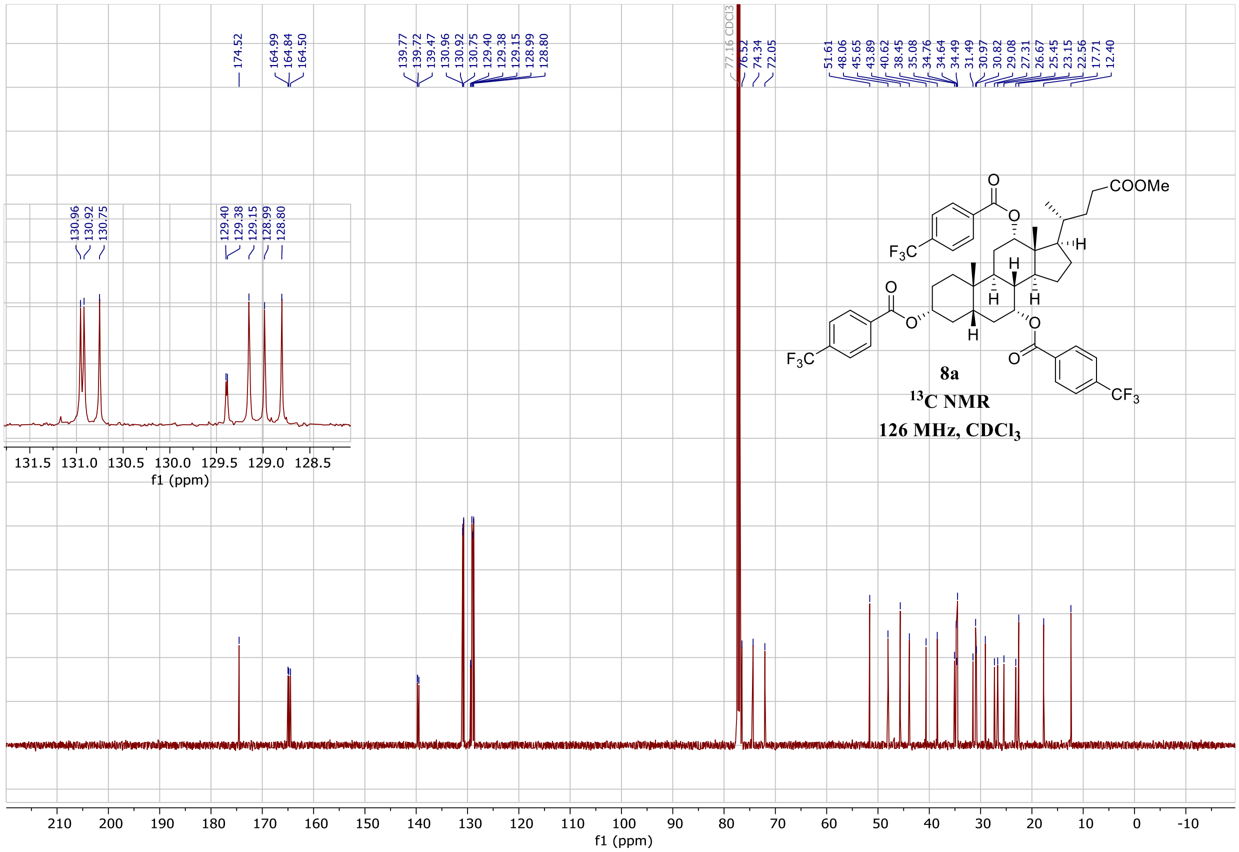
8a
^{13}C NMR
126 MHz, CDCl$_3$
COOMe
F_3C
CF_3
f1 (ppm)

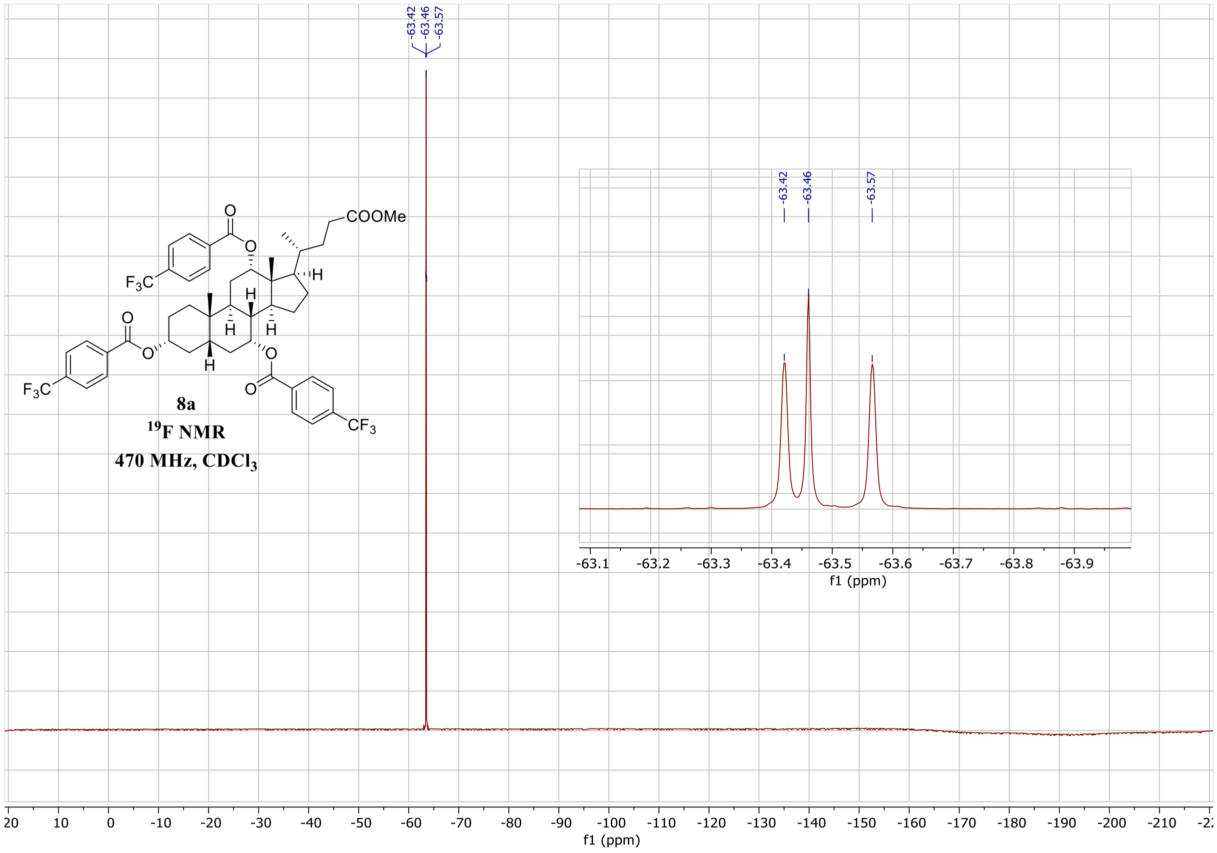
8a
^{19}F NMR
470 MHz, CDCl$_3$
COOMe
F_3C
CF_3
-63.42
-63.46
-63.57
f1 (ppm)

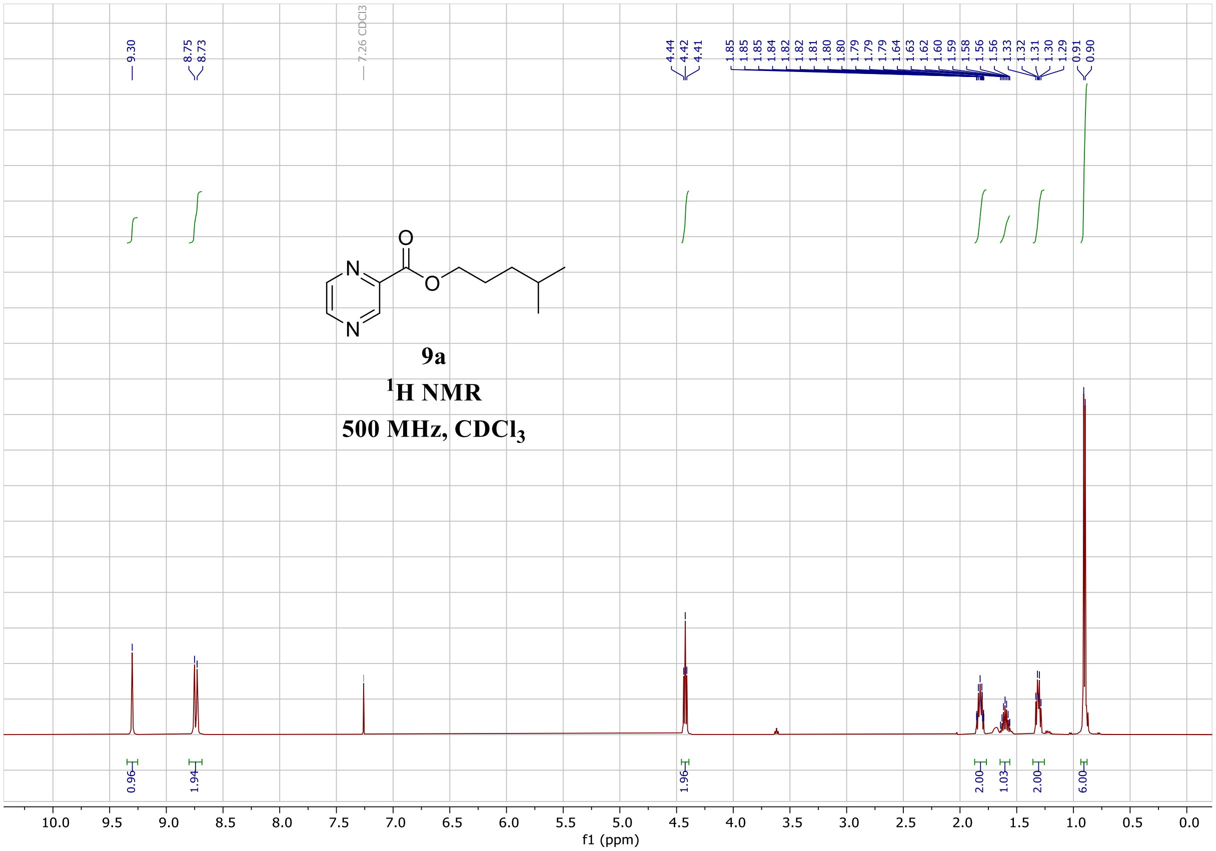
9a
^{1}H NMR
500 MHz, $CDCl_3$
f1 (ppm)

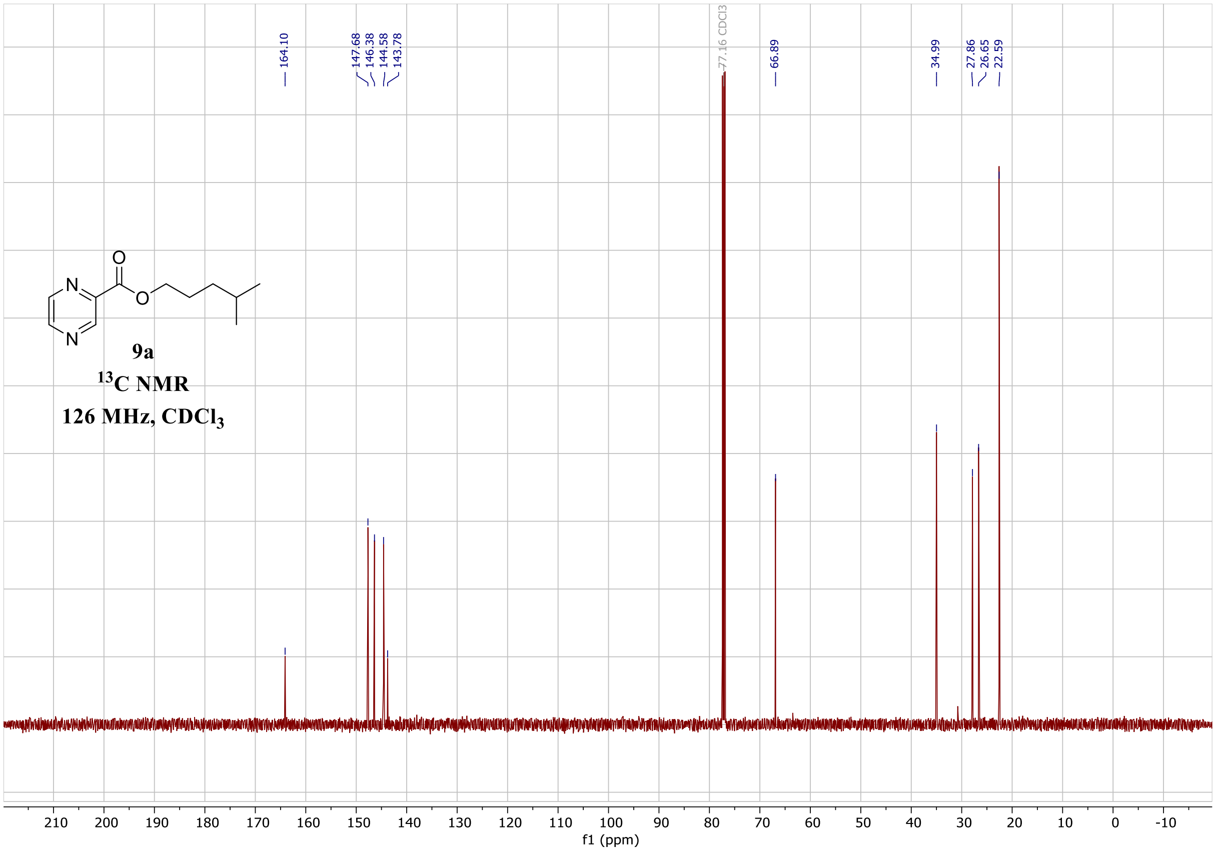
9a
^{13}C NMR
126 MHz, $CDCl_3$
f1 (ppm)

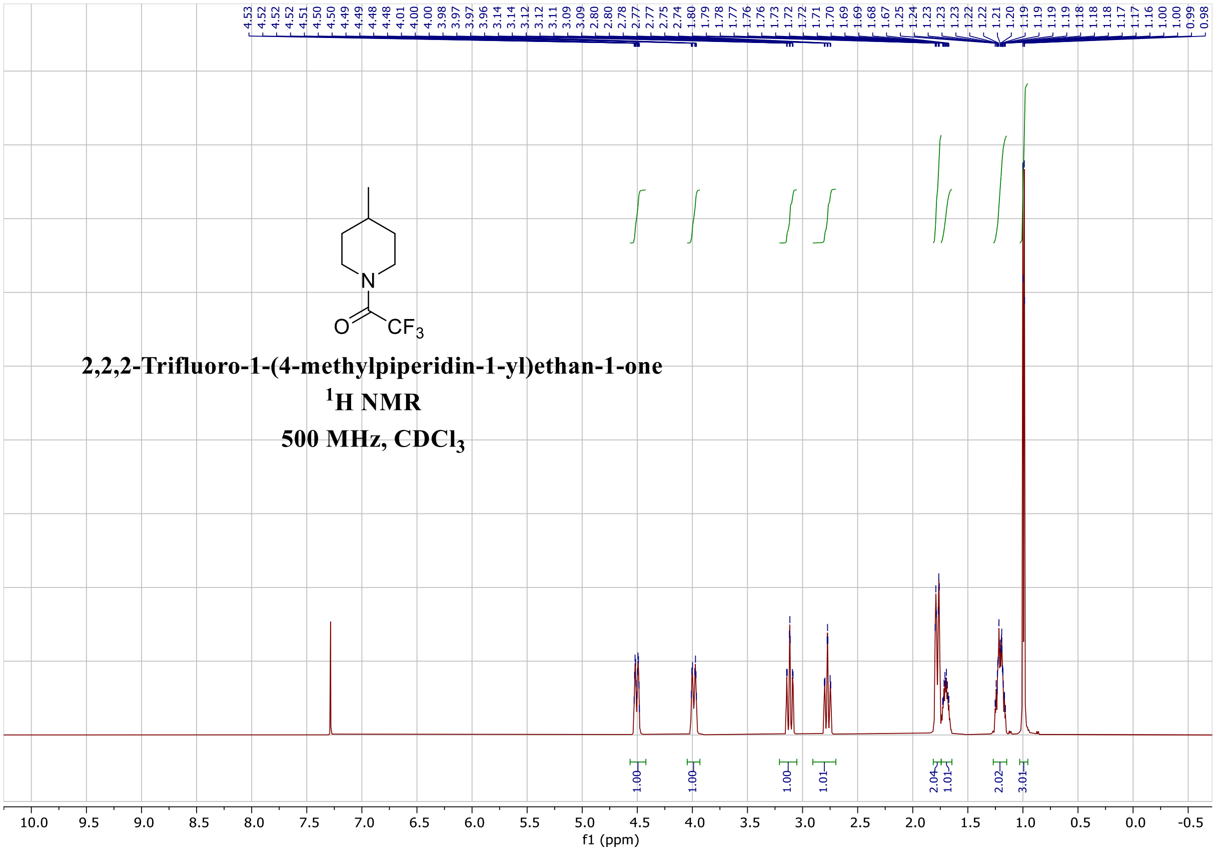

2,2,2-Trifluoro-1-(4-methylpiperidin-1-yl)ethan-1-one

^{1}H NMR

500 MHz, $CDCl_3$

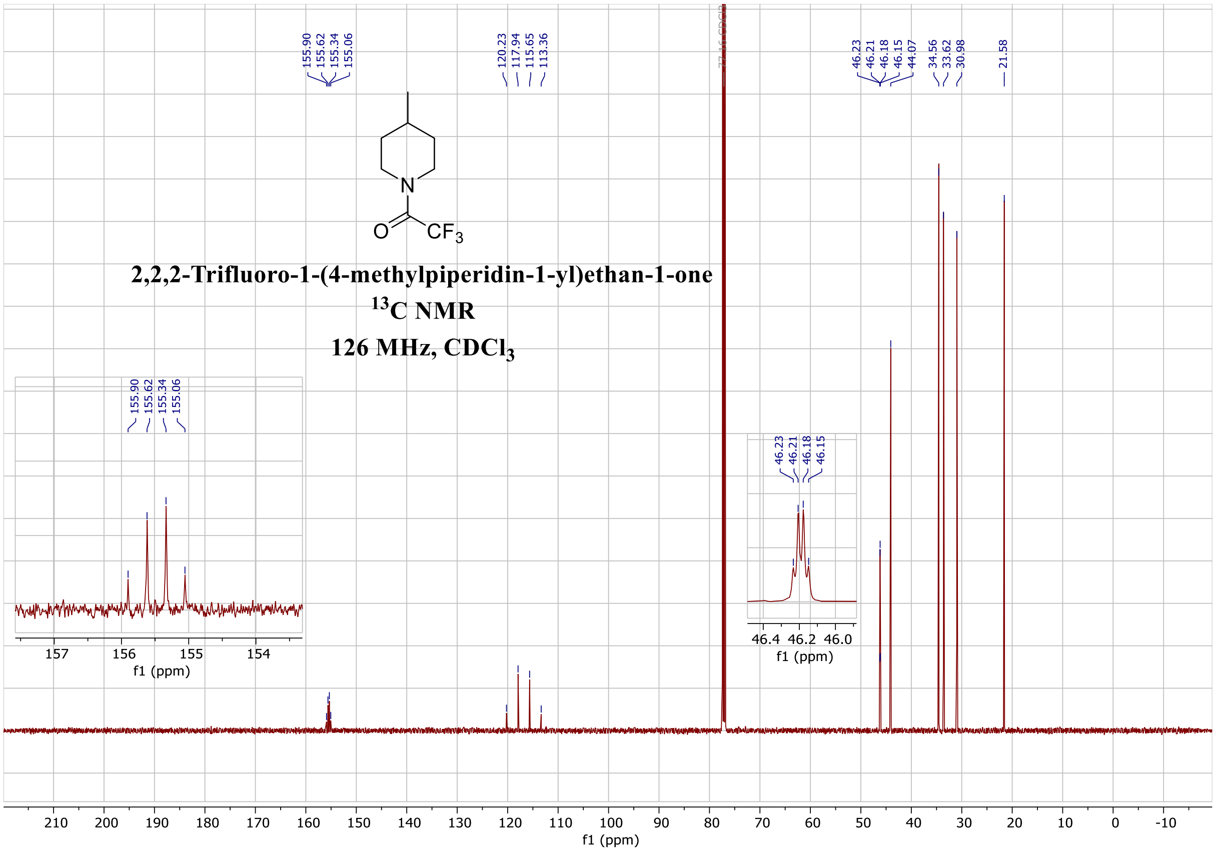

2,2,2-Trifluoro-1-(4-methylpiperidin-1-yl)ethan-1-one

^{13}C NMR

126 MHz, $CDCl_3$

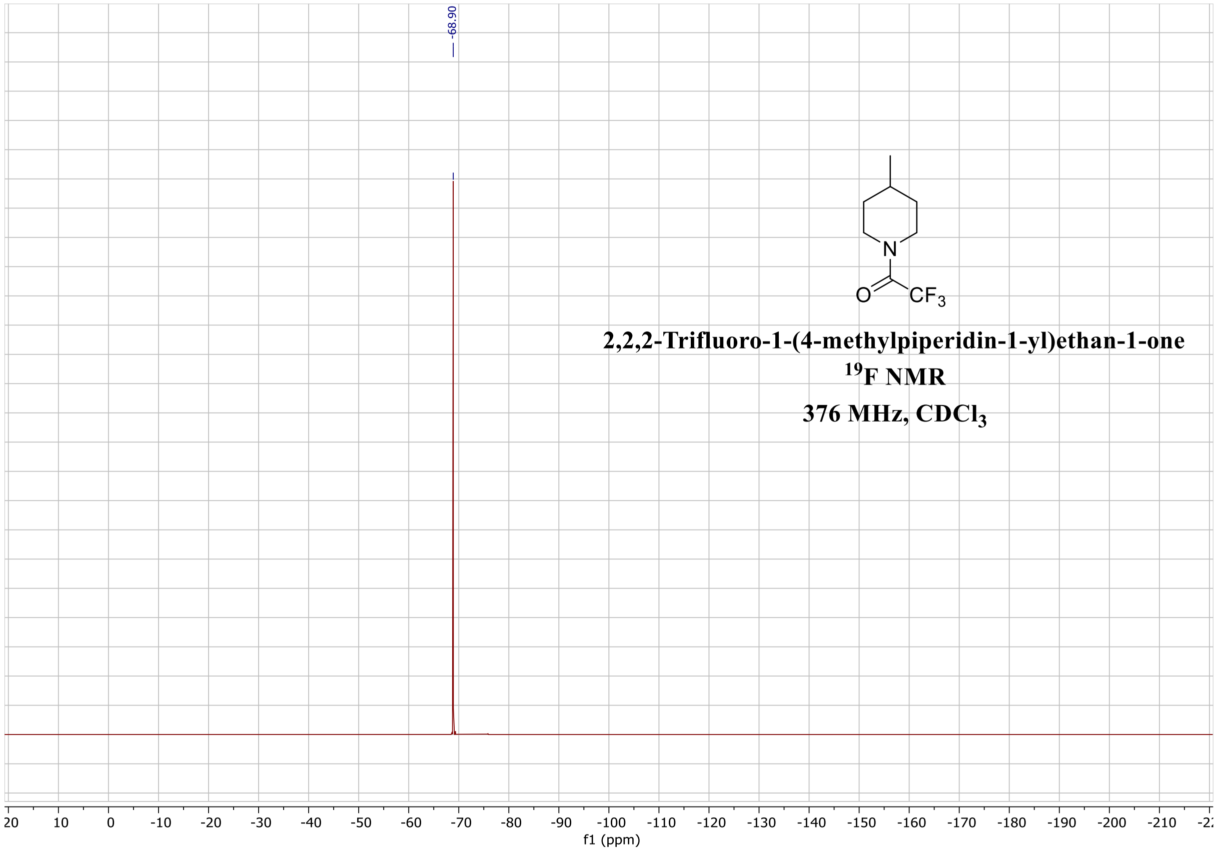

-68.90
N
O
CF3
2,2,2-Trifluoro-1-(4-methylpiperidin-1-yl)ethan-1-one
19F NMR
376 MHz, CDCl3
20 10 0 -10 -20 -30 -40 -50 -60 -70 -80 -90 -100 -110 -120 -130 -140 -150 -160 -170 -180 -190 -200 -210
f1 (ppm)

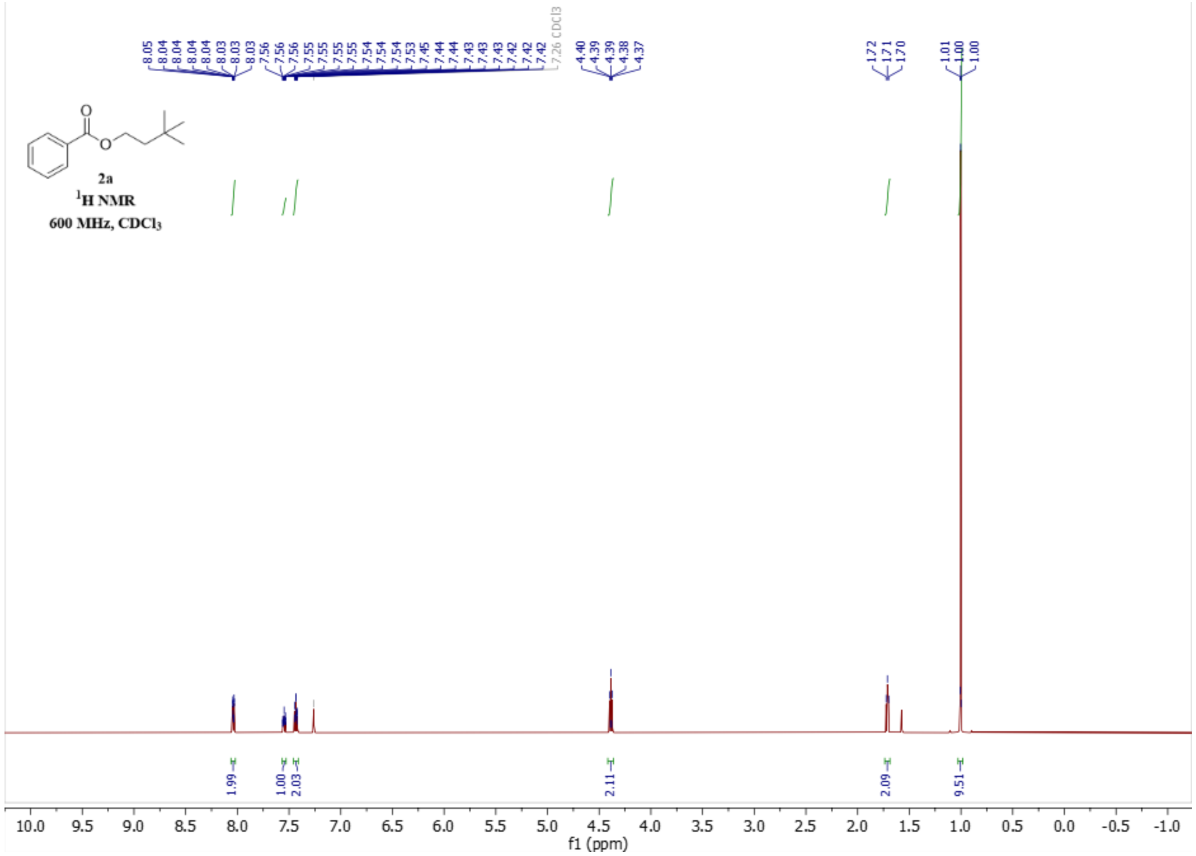

8.05 8.04 8.04 8.04 8.04 8.03 8.03 8.03 7.56 7.56 7.55 7.55 7.55 7.55 7.54 7.54 7.53 7.45 7.44 7.43 7.43 7.43 7.42 7.42 7.42
7.26 CDCl3
4.40 4.39 4.39 4.38 4.37
1.72 1.71 1.70
1.01 1.00 1.00
2a
1H NMR
600 MHz, CDCl3
1.99 1.00 2.03 2.11 2.09 9.51
10.0 9.5 9.0 8.5 8.0 7.5 7.0 6.5 6.0 5.5 5.0 4.5 4.0 3.5 3.0 2.5 2.0 1.5 1.0 0.5 0.0 -0.5 -1.0
f1 (ppm)

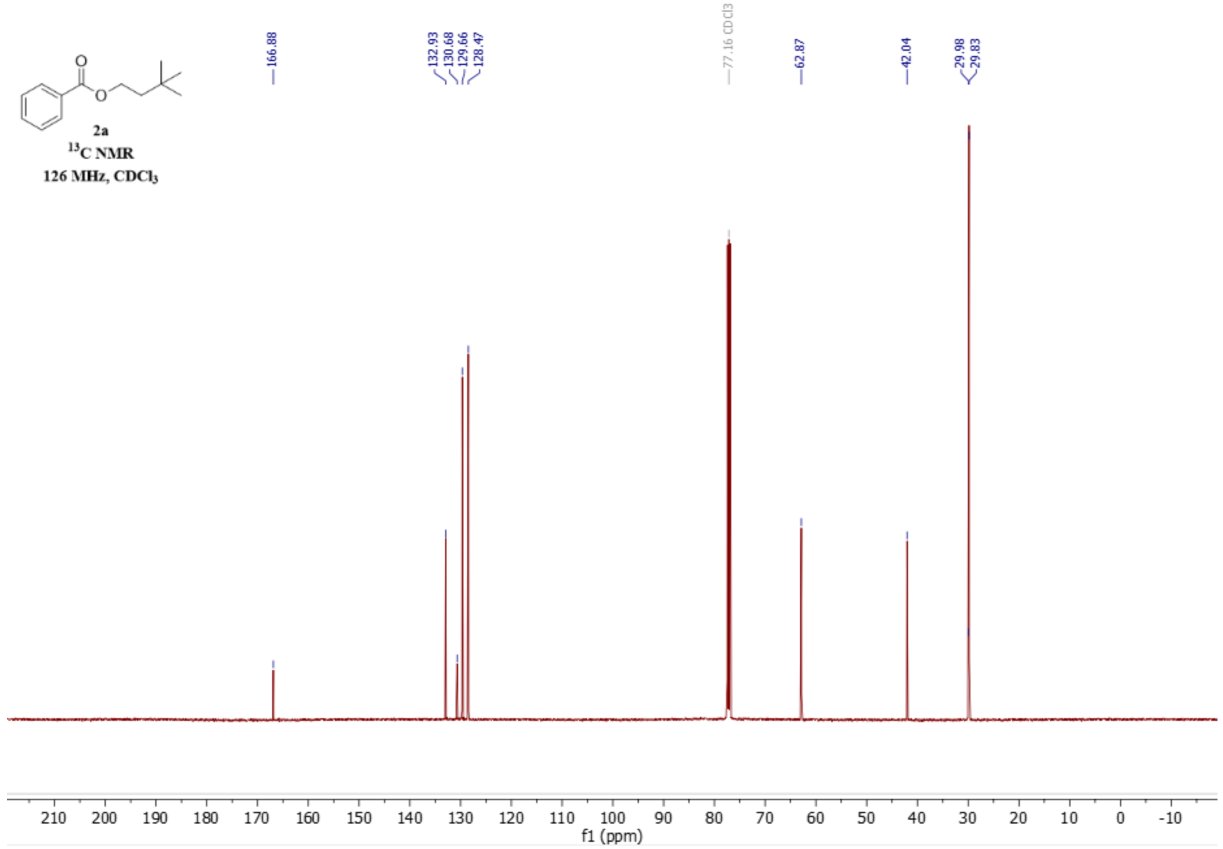
2a
^{13}C NMR
126 MHz, $CDCl_3$
166.88
132.93
130.68
129.66
128.47
77.16 CDCl3
62.87
42.04
29.98
29.83
f1 (ppm)

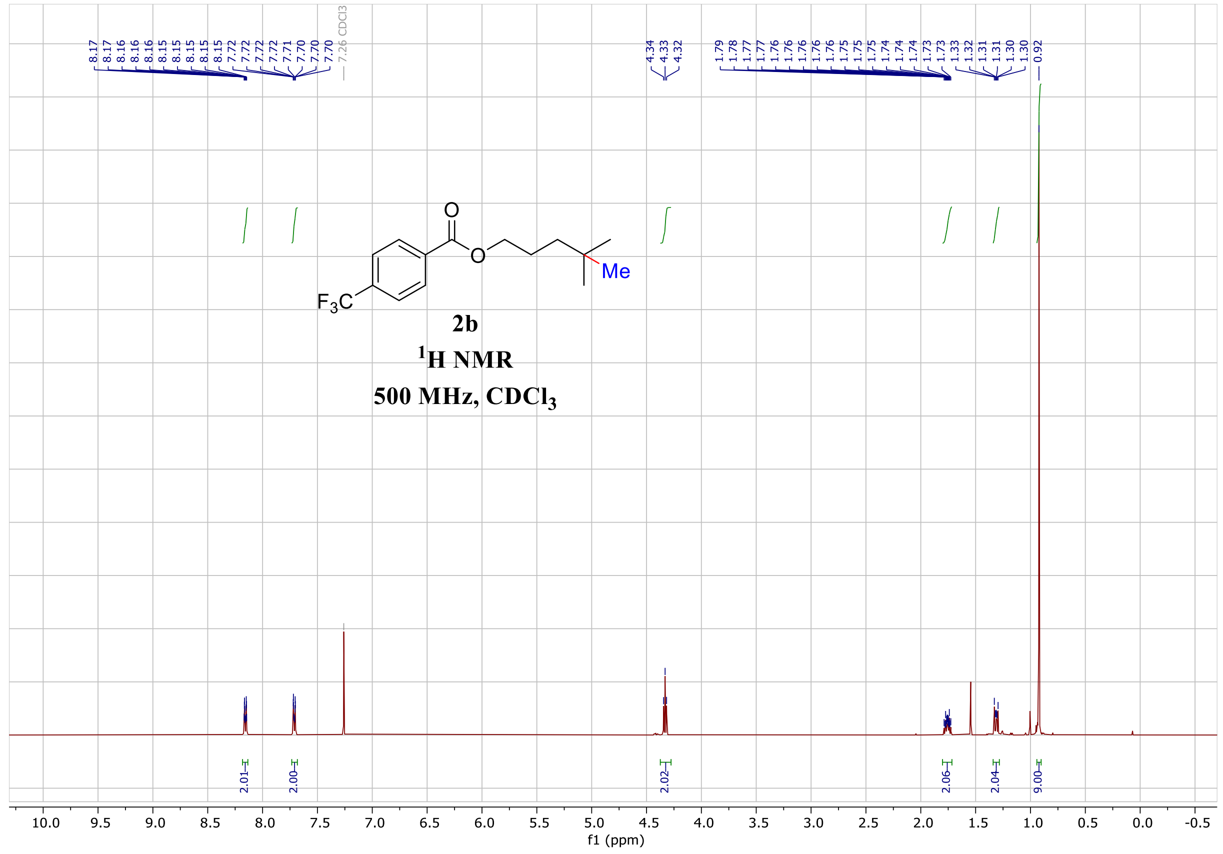
2b
^{1}H NMR
500 MHz, $CDCl_3$
Me
F3C
7.26 CDCl3
4.34
4.33
4.32
0.92
2.01
2.00
2.02
2.06
2.04
9.00
f1 (ppm)

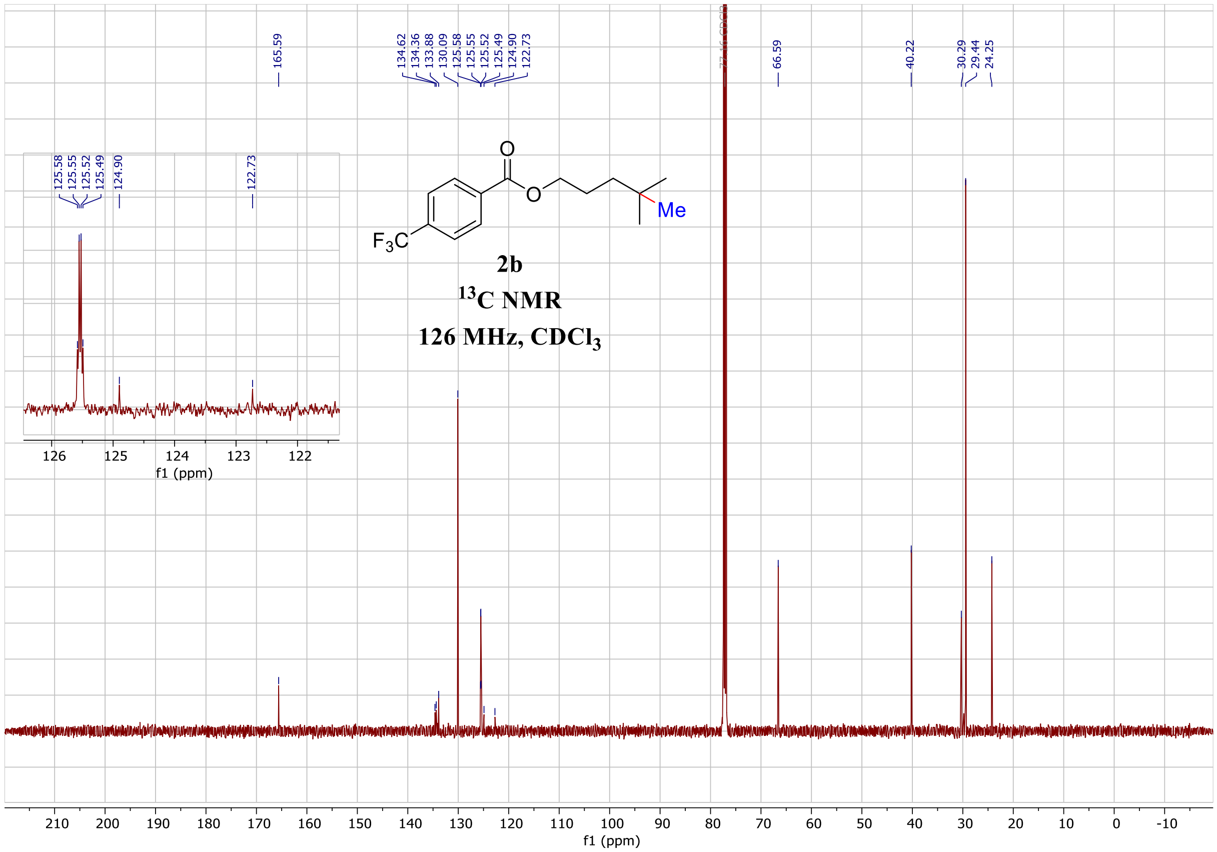
165.59
134.62
134.36
133.88
130.09
125.58
125.55
125.52
125.49
124.90
122.73
66.59
40.22
30.29
29.44
24.25
2b
^{13}C NMR
126 MHz, $CDCl_3$
f1 (ppm)

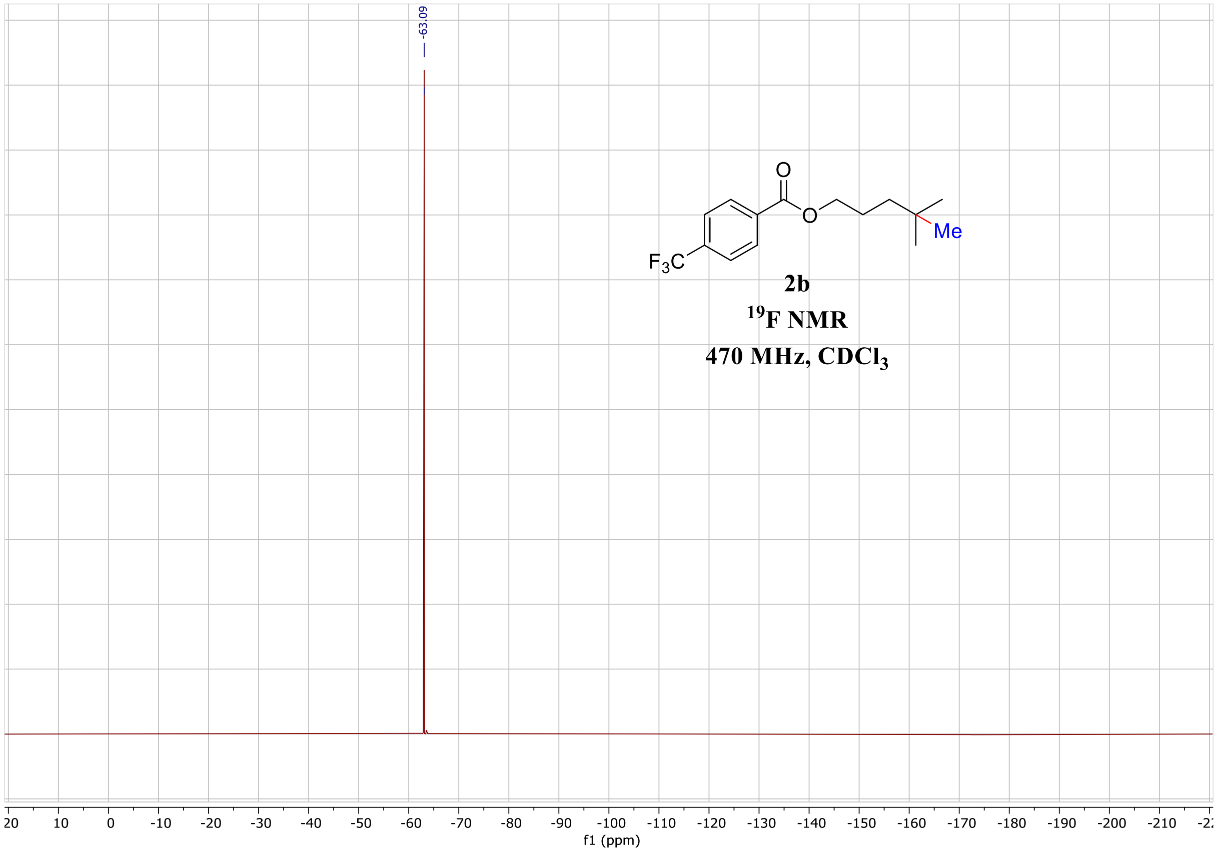
-63.09
2b
^{19}F NMR
470 MHz, $CDCl_3$
f1 (ppm)

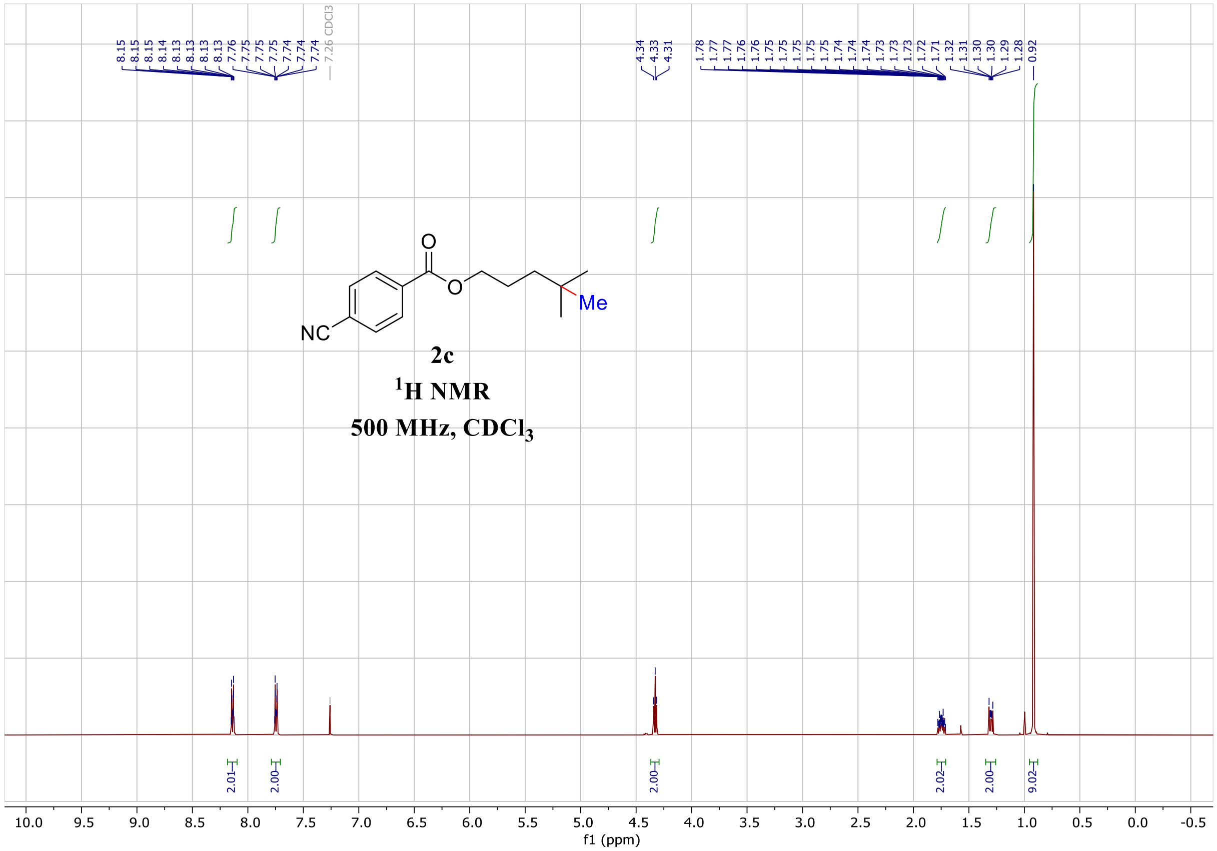
2c
1H NMR
500 MHz, CDCl3
f1 (ppm)

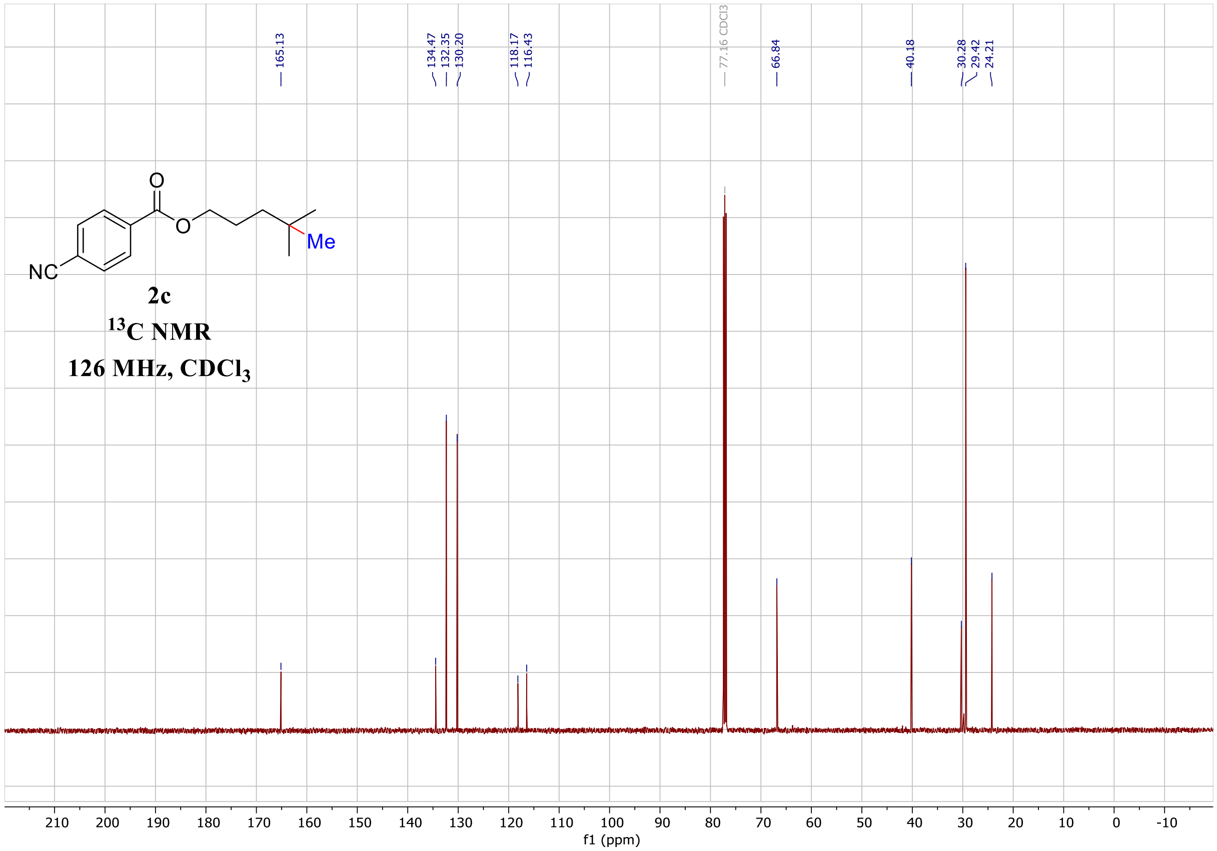
2c
13C NMR
126 MHz, CDCl3
165.13
134.47
132.35
130.20
118.17
116.43
77.16 CDCl3
66.84
40.18
30.28
29.42
24.21
f1 (ppm)

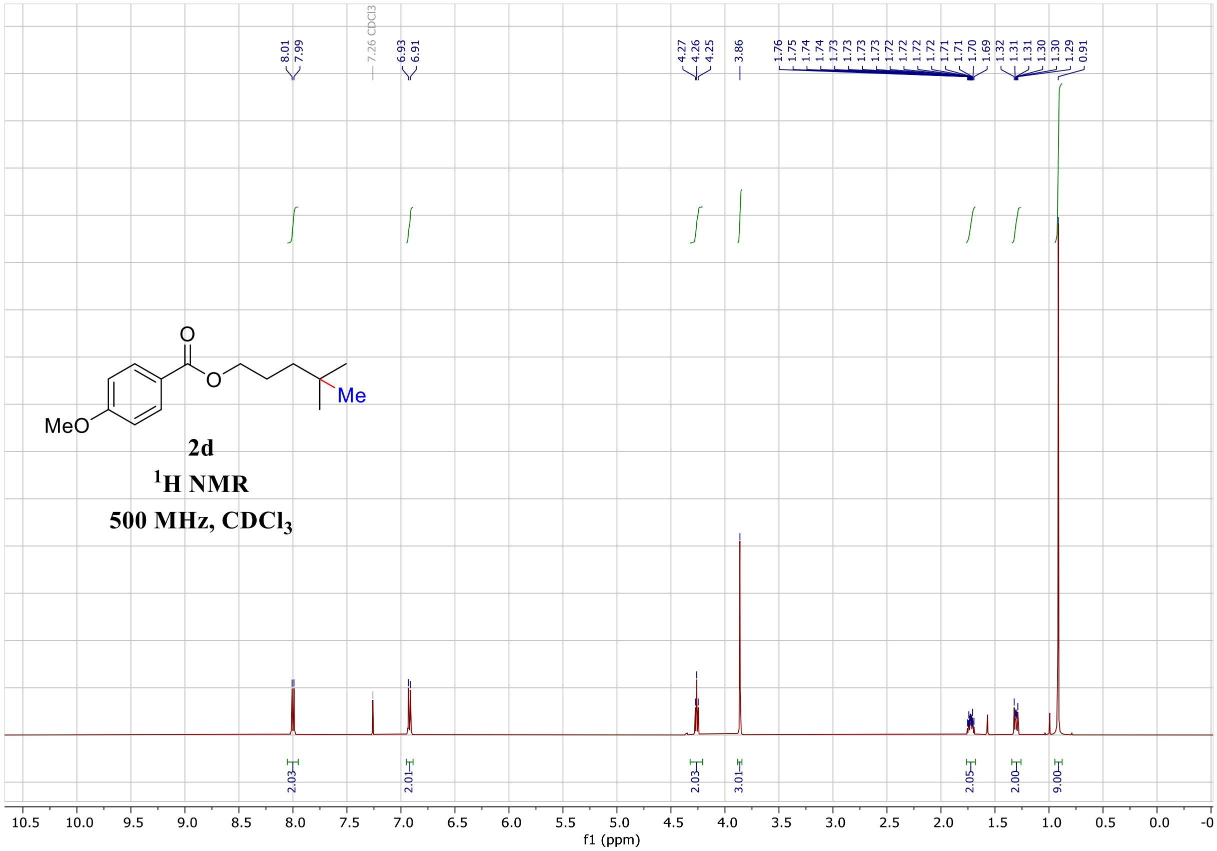
MeO
O
O
Me
2d
1H NMR
500 MHz, CDCl3
f1 (ppm)

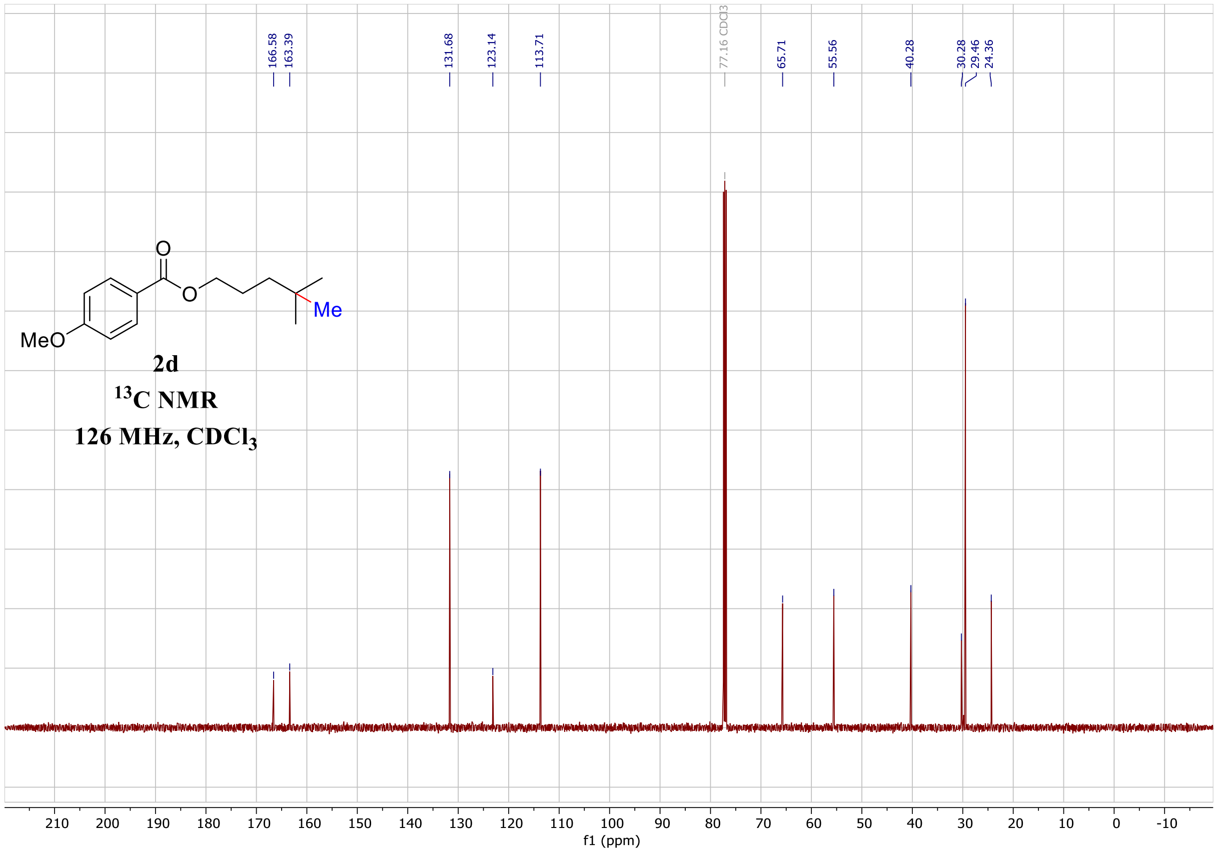
MeO
O
O
Me
2d
13C NMR
126 MHz, CDCl3
f1 (ppm)

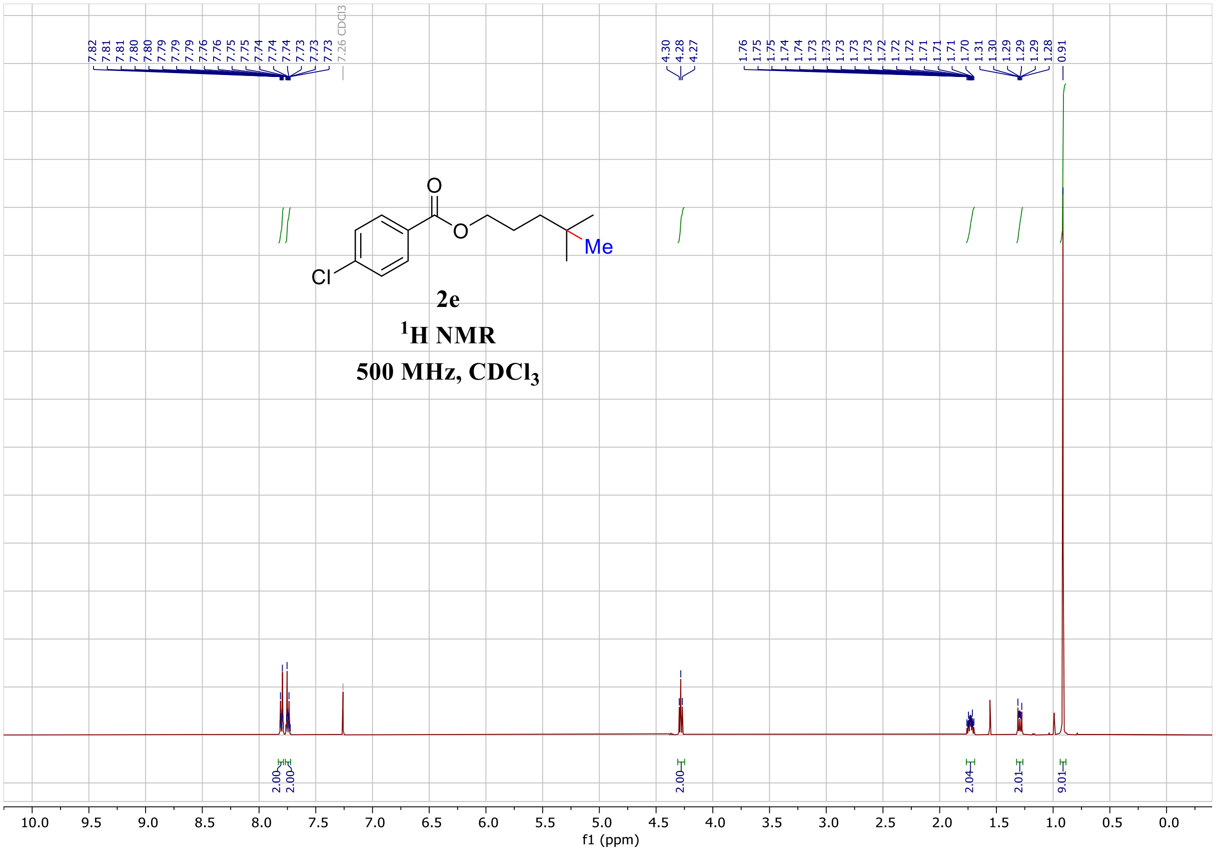
7.82
7.81
7.81
7.80
7.79
7.79
7.79
7.76
7.75
7.75
7.74
7.74
7.74
7.73
7.73
7.73
7.26 CDCl3
4.30
4.28
4.27
1.76
1.75
1.75
1.74
1.74
1.73
1.73
1.73
1.73
1.72
1.72
1.72
1.71
1.71
1.70
1.31
1.30
1.29
1.29
1.28
0.91
O
O
Cl
Me
2e
1H NMR
500 MHz, CDCl3
2.00
2.00
2.00
2.04
2.01
9.01
10.0
9.5
9.0
8.5
8.0
7.5
7.0
6.5
6.0
5.5
5.0
4.5
4.0
3.5
3.0
2.5
2.0
1.5
1.0
0.5
0.0
f1 (ppm)

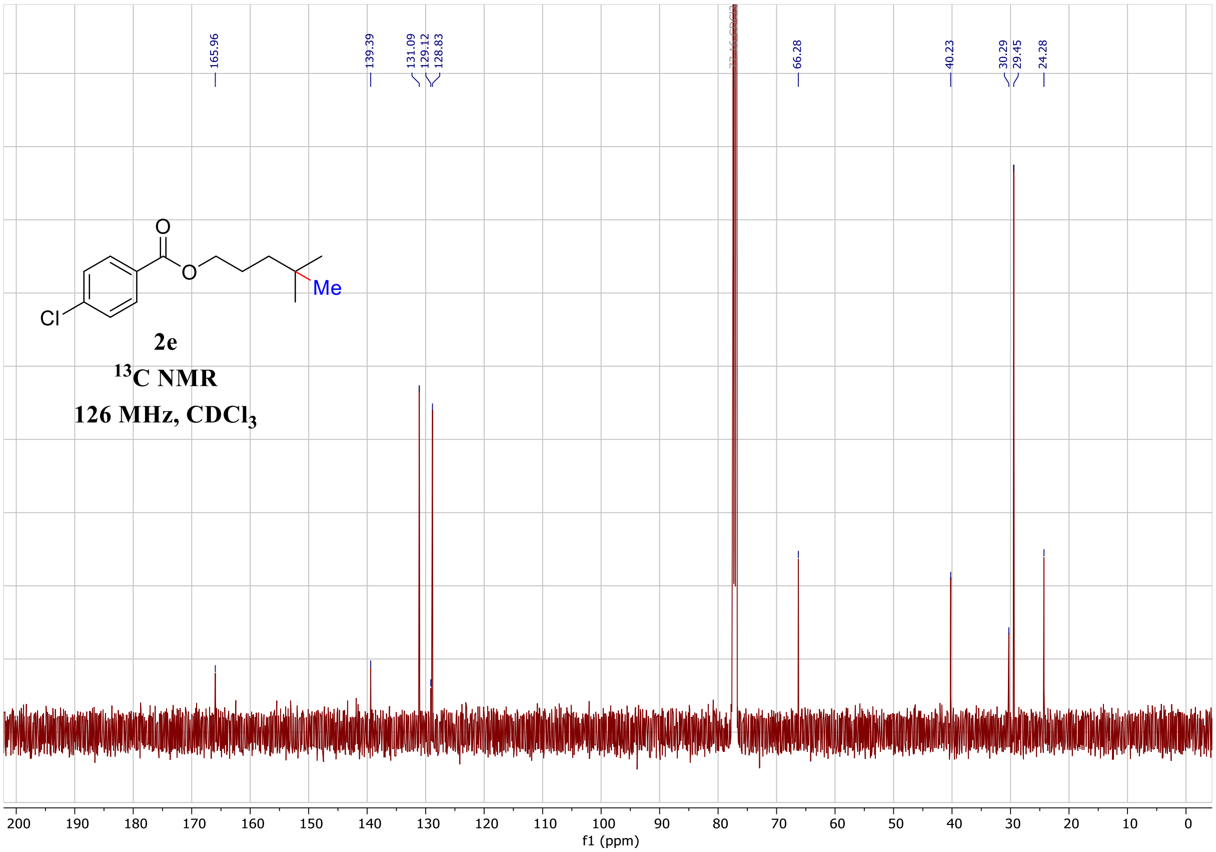
165.96
139.39
131.09
129.12
128.83
66.28
40.23
30.29
29.45
24.28
O
O
Cl
Me
2e
13C NMR
126 MHz, CDCl3
200
190
180
170
160
150
140
130
120
110
100
90
80
70
60
50
40
30
20
10
0
f1 (ppm)

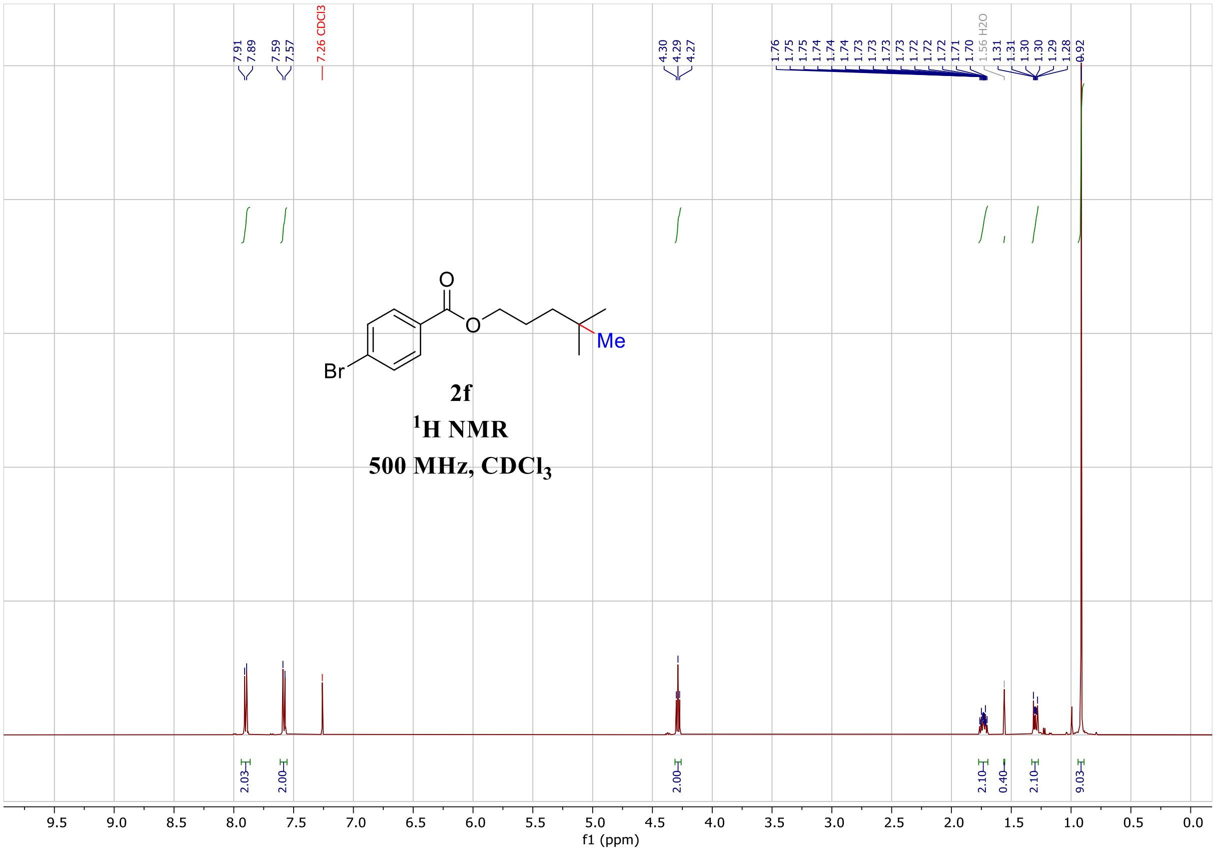
7.91
7.89
7.59
7.57
7.26 CDCl3
4.30
4.29
4.27
0.92
2f
1H NMR
500 MHz, CDCl3
2.03
2.00
2.00
2.10
0.40
2.10
9.03
f1 (ppm)

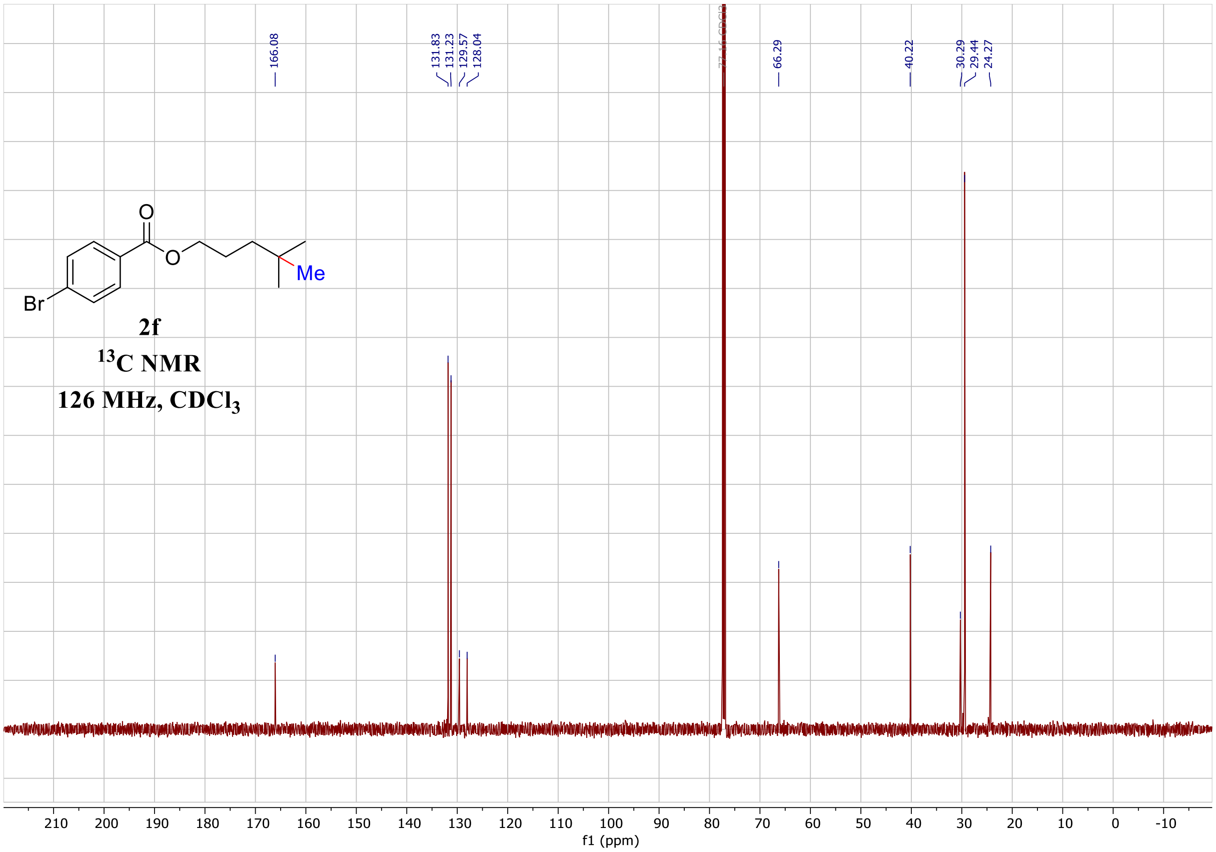
166.08
131.83
131.23
129.57
128.04
66.29
40.22
30.29
29.44
24.27
2f
13C NMR
126 MHz, CDCl3
f1 (ppm)

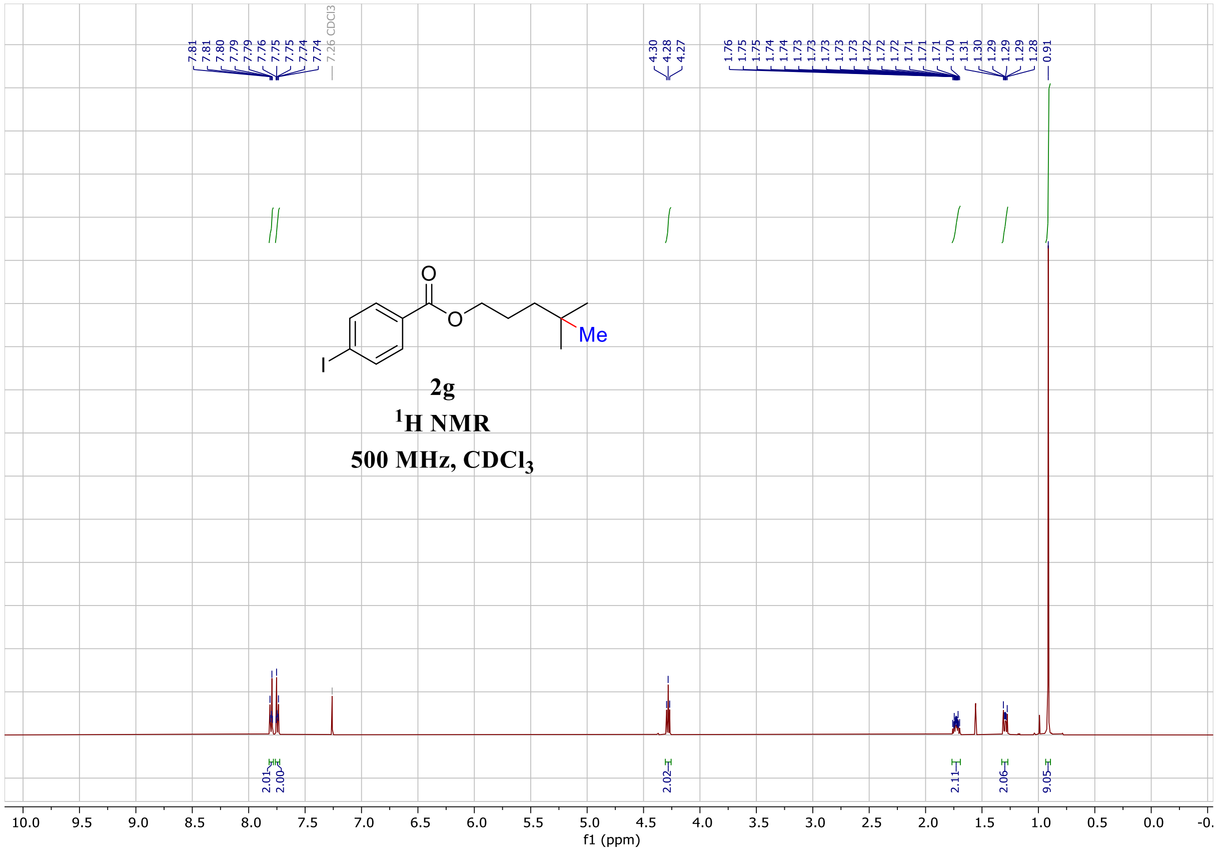

2g
^{1}H NMR
500 MHz, CDCl$_3$
Me
f1 (ppm)

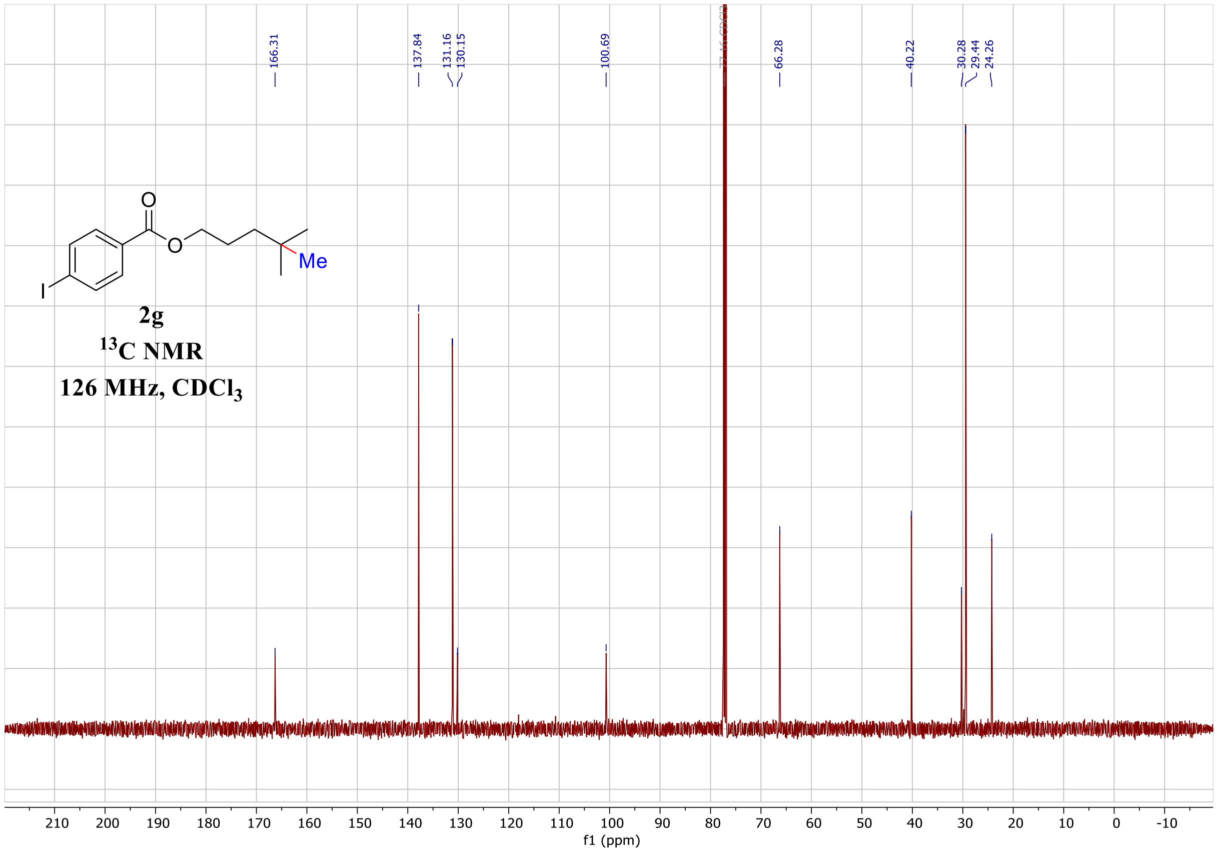

2g
^{13}C NMR
126 MHz, CDCl$_3$
Me
f1 (ppm)

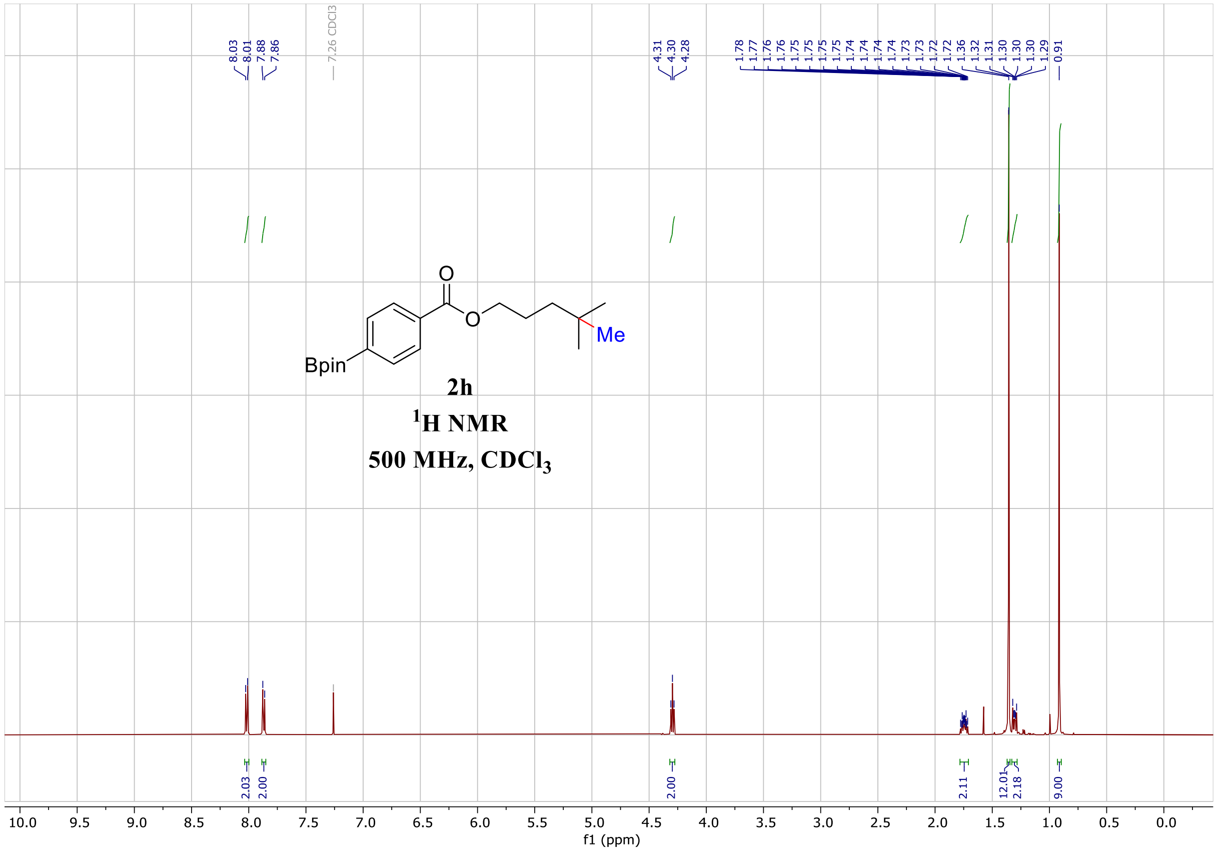
Bpin
O
O
Me
2h
1H NMR
500 MHz, CDCl3
f1 (ppm)

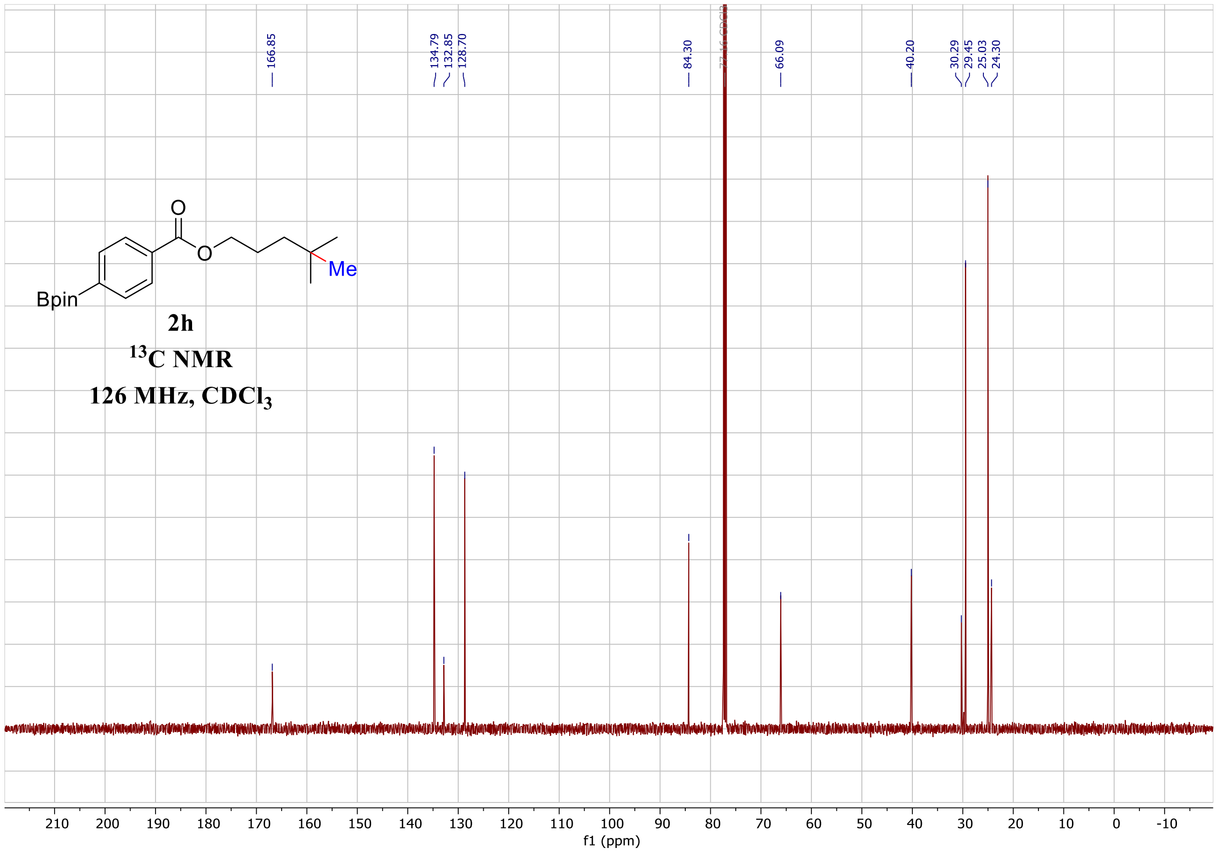
Bpin
O
O
Me
2h
13C NMR
126 MHz, CDCl3
f1 (ppm)

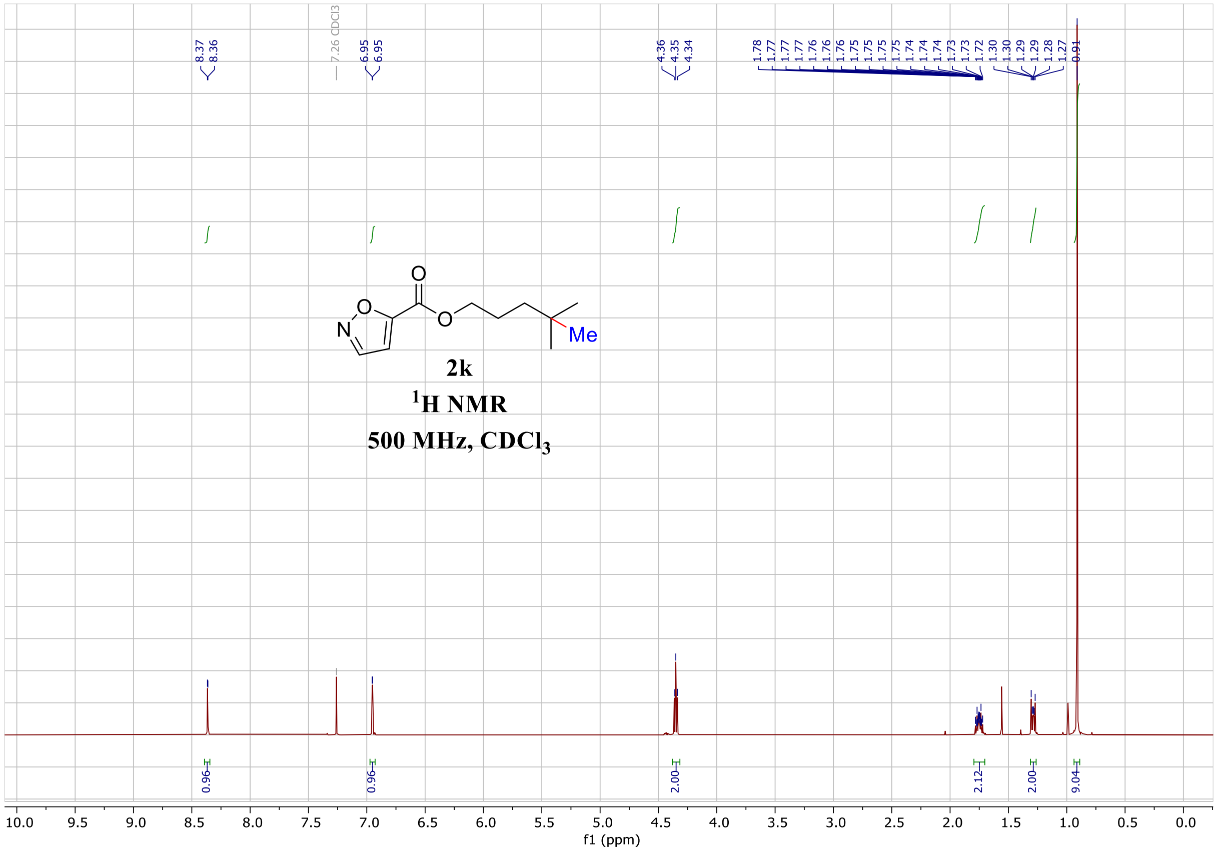

2k
^{1}H NMR
500 MHz, $CDCl_3$
f1 (ppm)

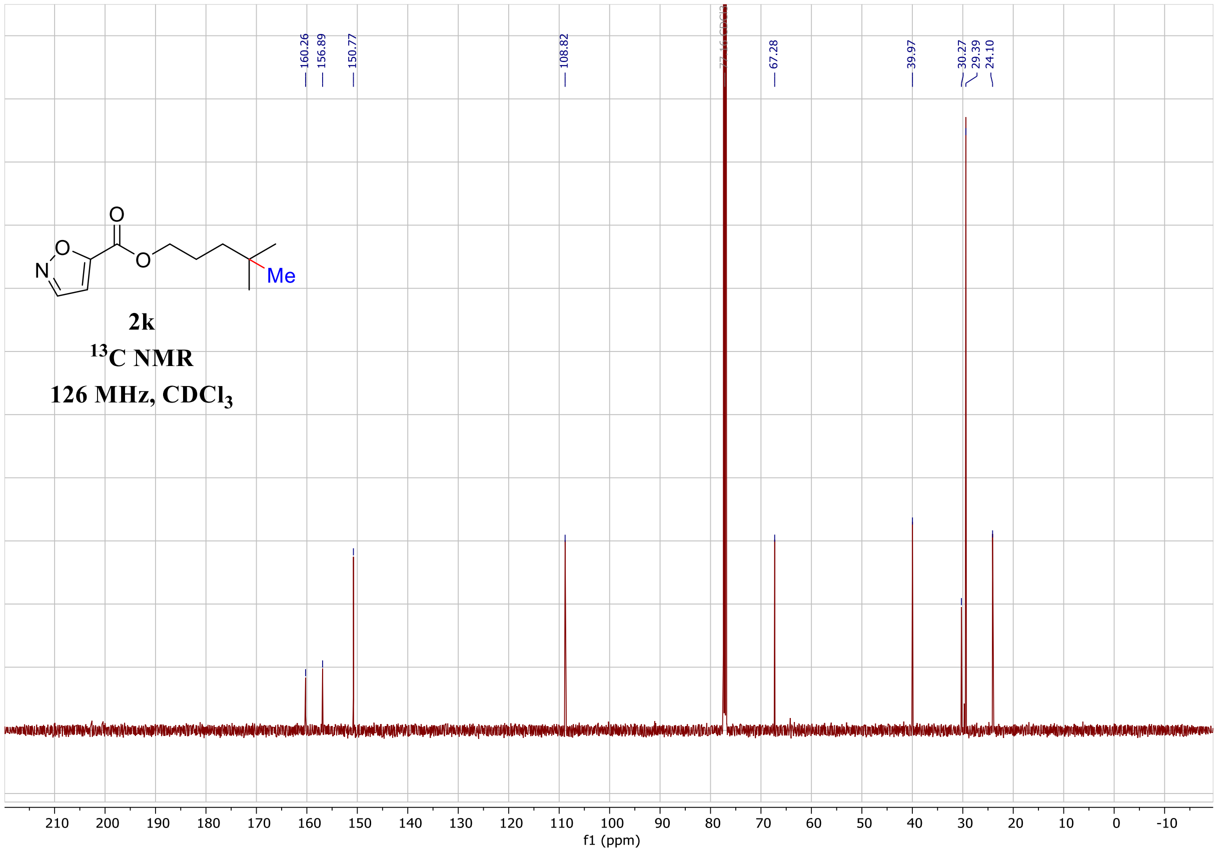

2k
^{13}C NMR
126 MHz, $CDCl_3$
f1 (ppm)

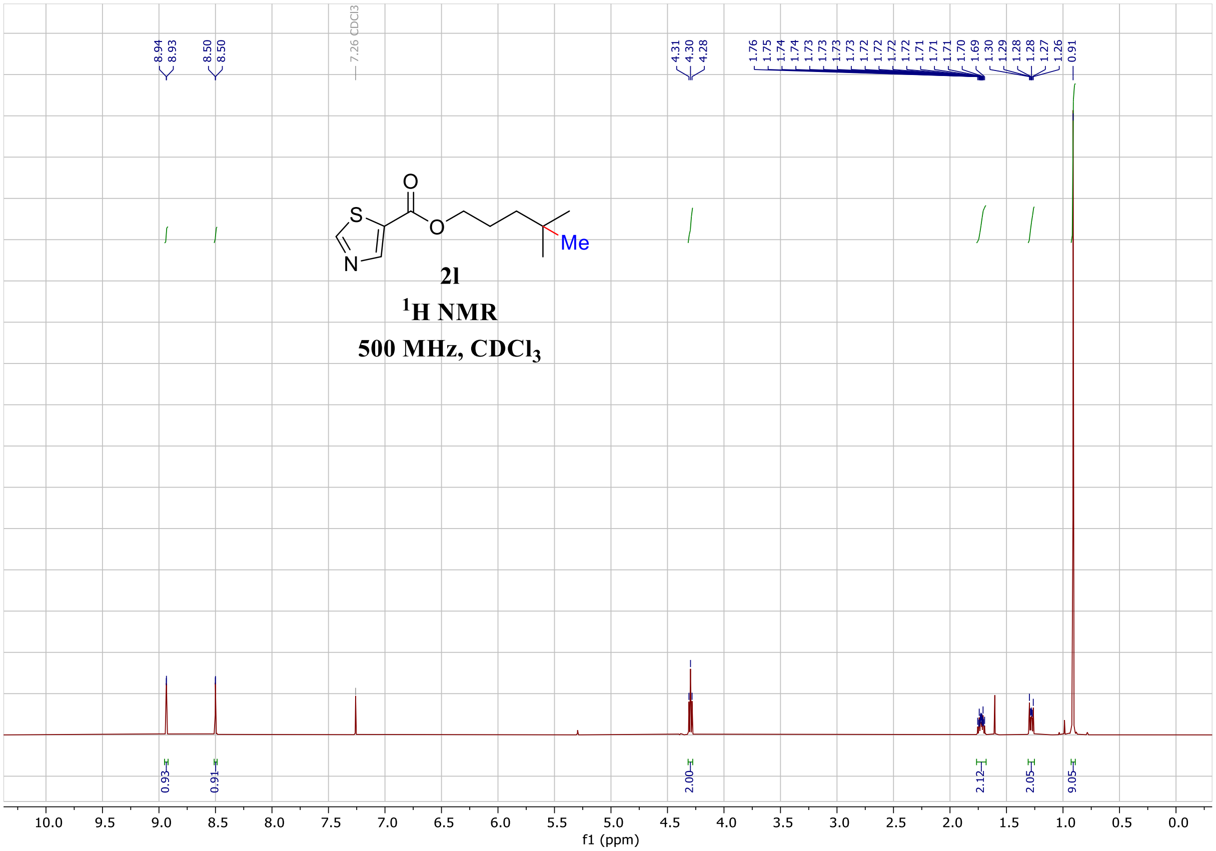
8.94
8.93
8.50
8.50
7.26 CDCl3
4.31
4.30
4.28
1.76
1.75
1.74
1.74
1.73
1.73
1.73
1.73
1.72
1.72
1.72
1.72
1.71
1.71
1.71
1.70
1.69
1.30
1.29
1.28
1.28
1.27
1.26
0.91
S
O
O
N
Me
2l
^{1}H NMR
500 MHz, $CDCl_3$
0.93
0.91
2.00
2.12
2.05
9.05
10.0 9.5 9.0 8.5 8.0 7.5 7.0 6.5 6.0 5.5 5.0 4.5 4.0 3.5 3.0 2.5 2.0 1.5 1.0 0.5 0.0
f1 (ppm)

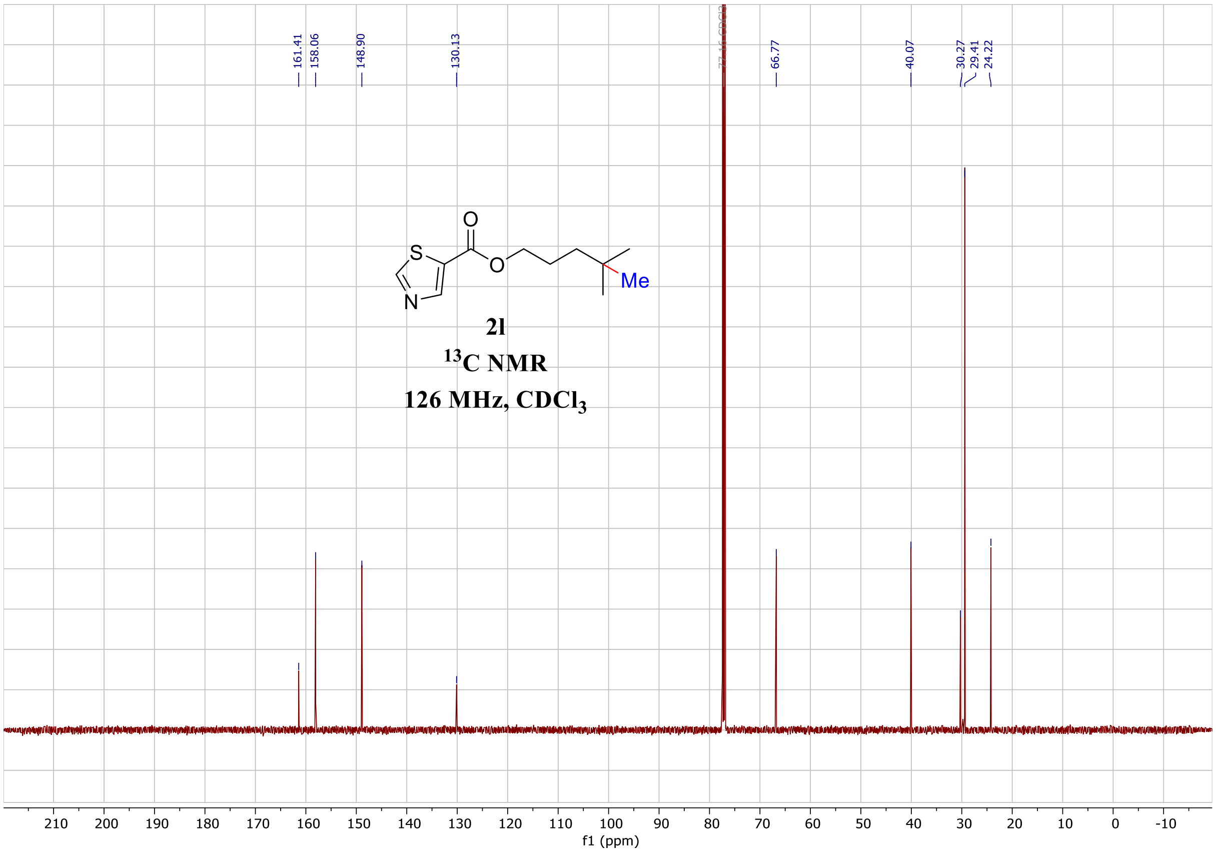
161.41
158.06
148.90
130.13
66.77
40.07
30.27
29.41
24.22
S
O
O
N
Me
2l
^{13}C NMR
126 MHz, $CDCl_3$
210 200 190 180 170 160 150 140 130 120 110 100 90 80 70 60 50 40 30 20 10 0 -10
f1 (ppm)

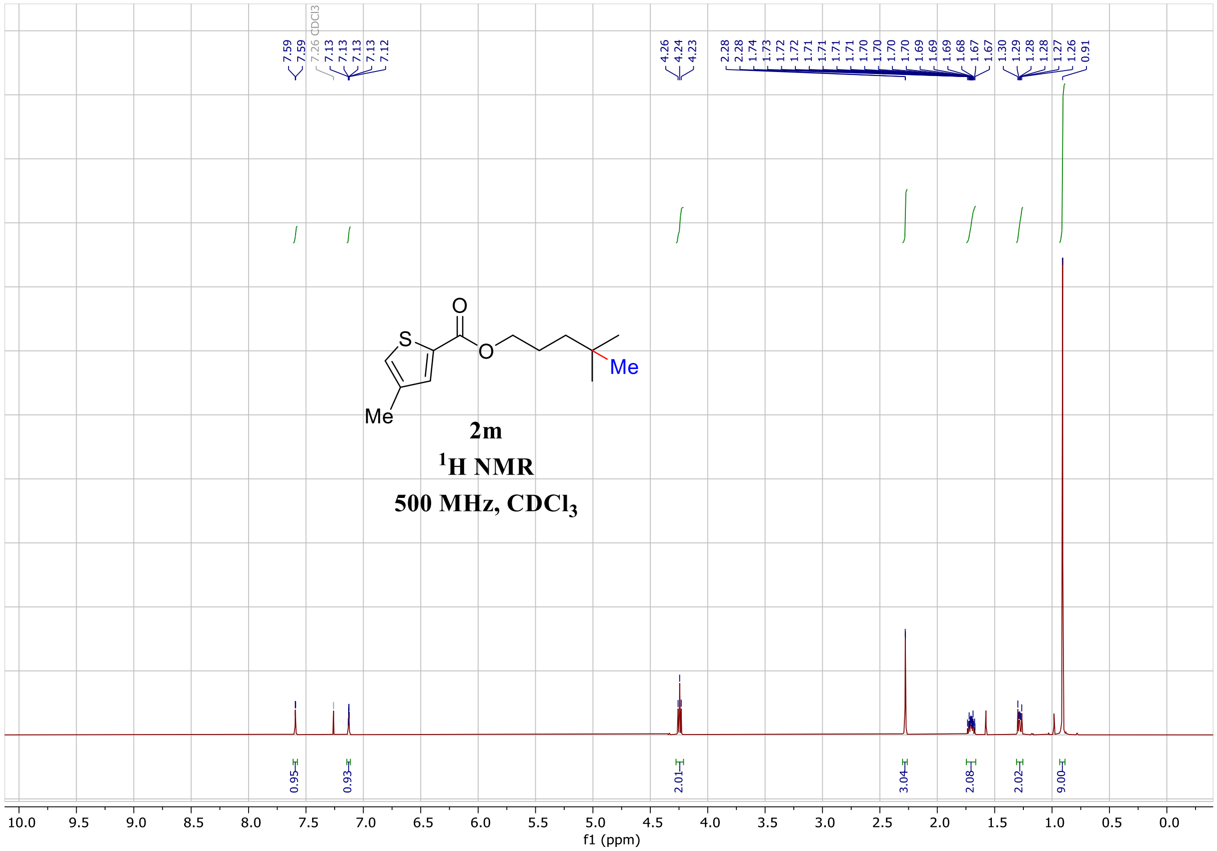

2m
^{1}H NMR
500 MHz, $CDCl_3$

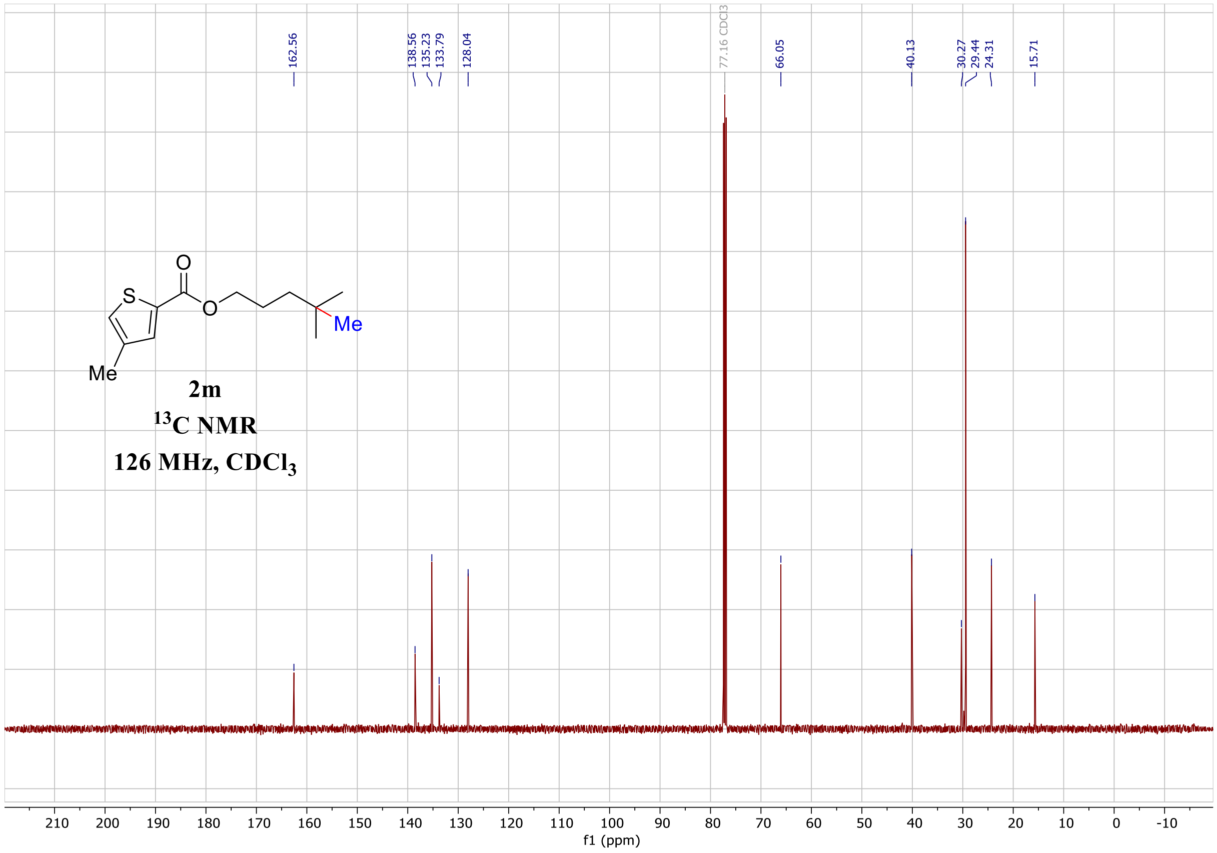

2m
^{13}C NMR
126 MHz, $CDCl_3$

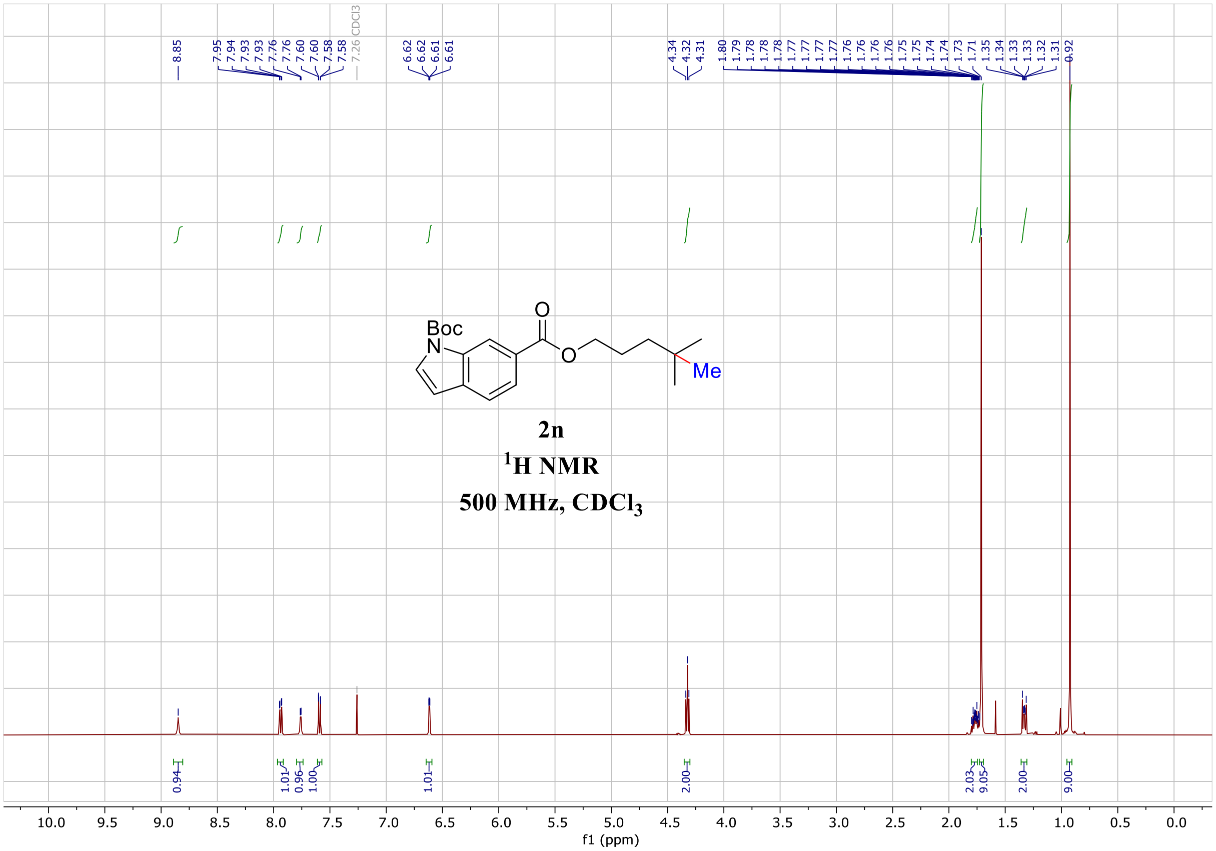
Boc
N
O
O
Me
2n
1H NMR
500 MHz, CDCl3
f1 (ppm)

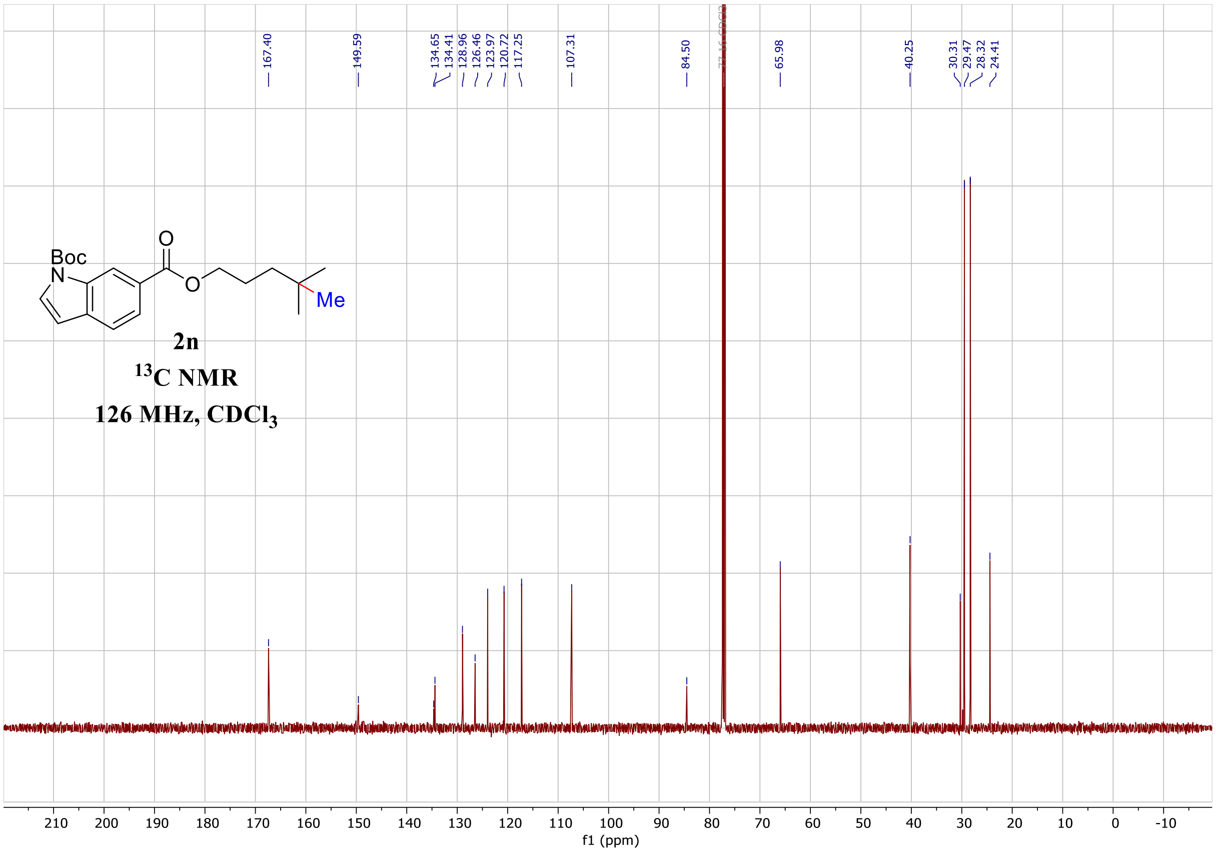
Boc
N
O
O
Me
2n
13C NMR
126 MHz, CDCl3
f1 (ppm)

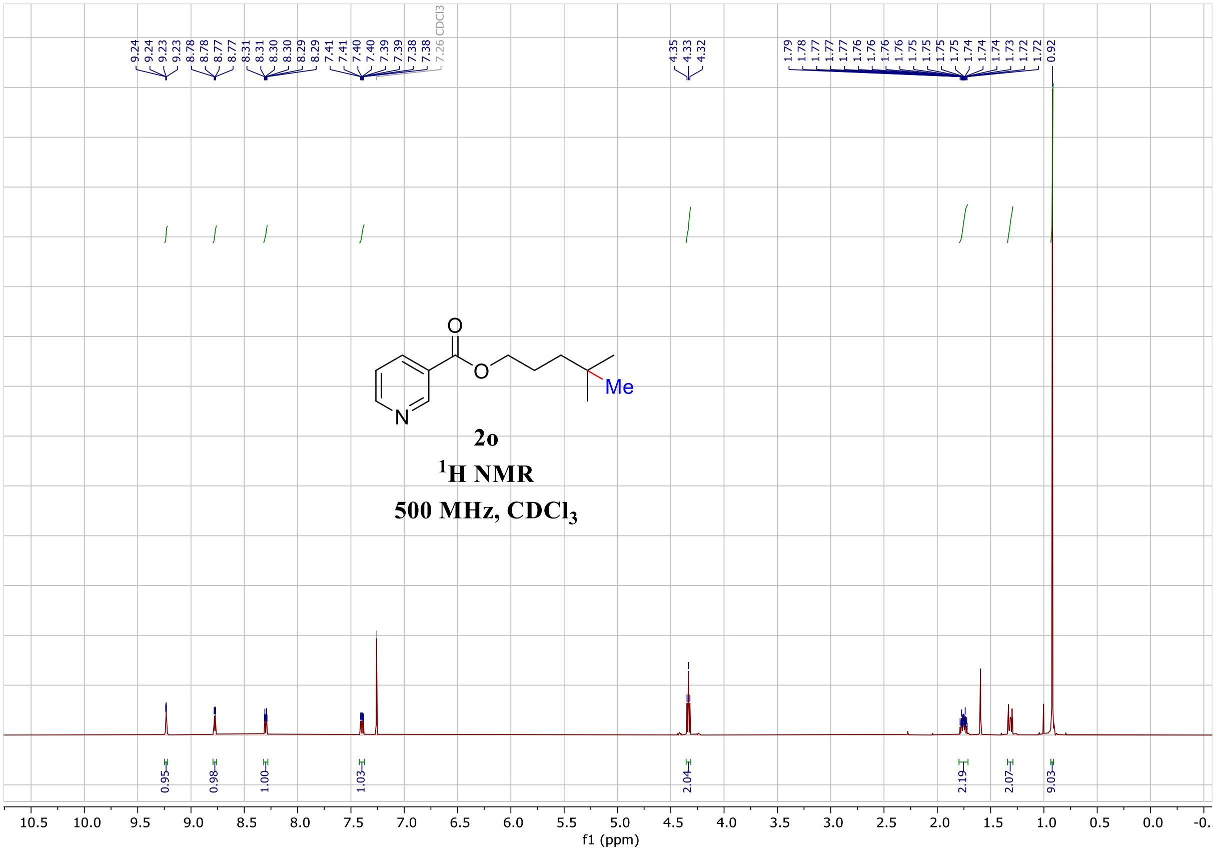

2o
^{1}H NMR
500 MHz, $CDCl_3$

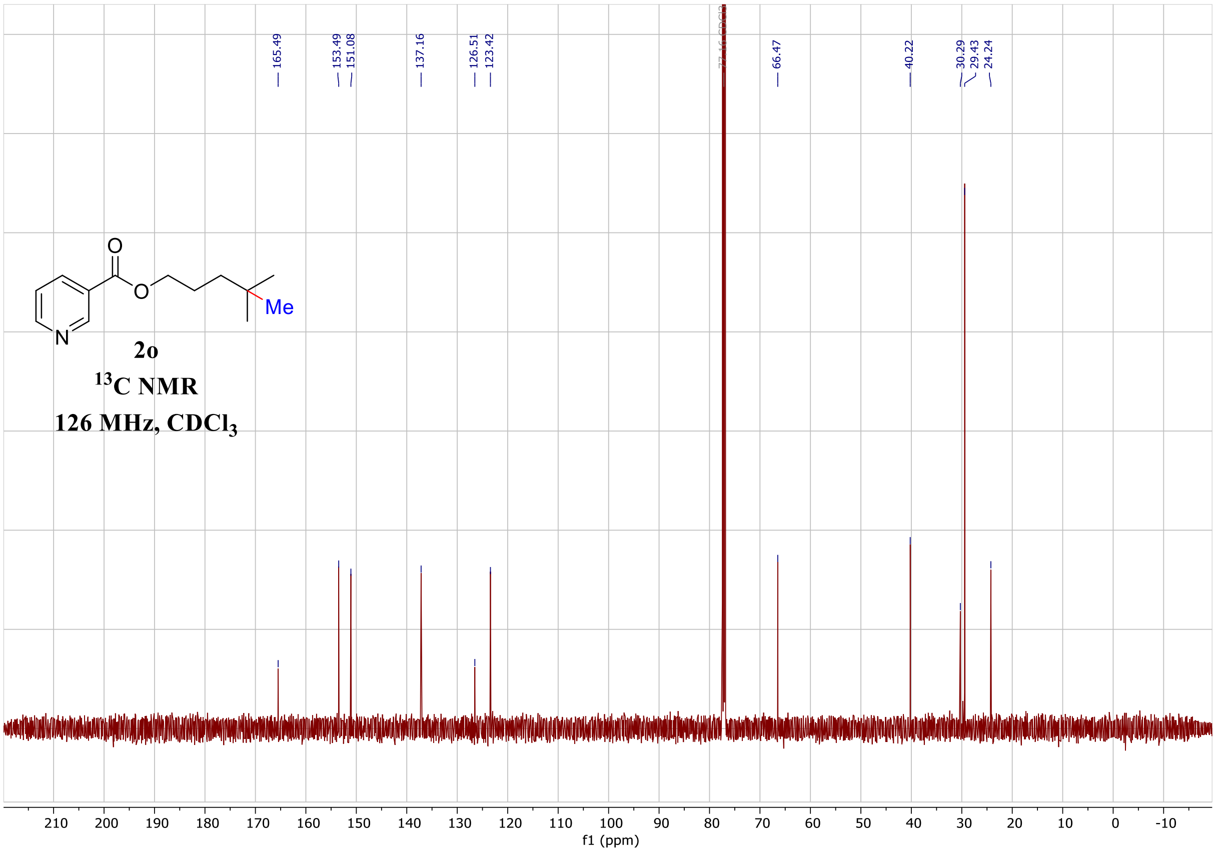

2o
^{13}C NMR
126 MHz, $CDCl_3$

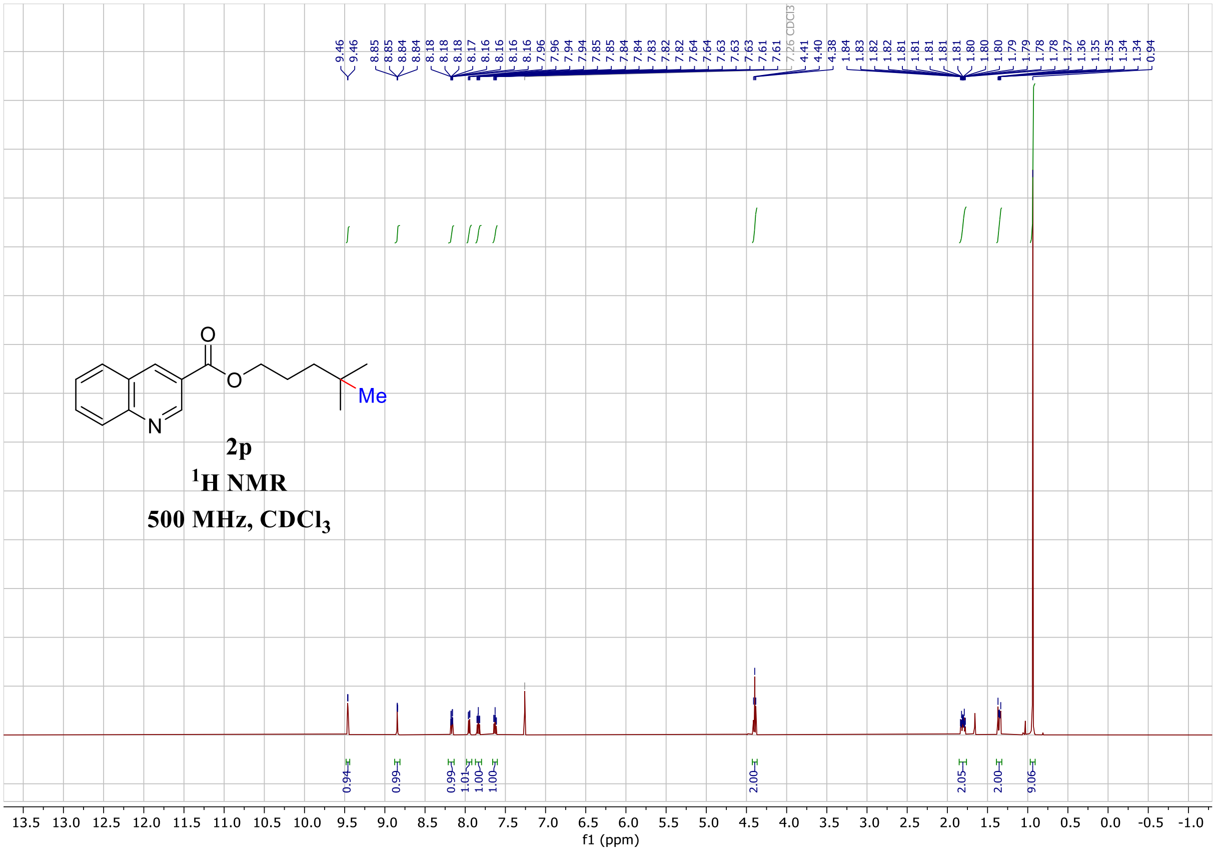
2p
^{1}H NMR
500 MHz, $CDCl_3$
f1 (ppm)

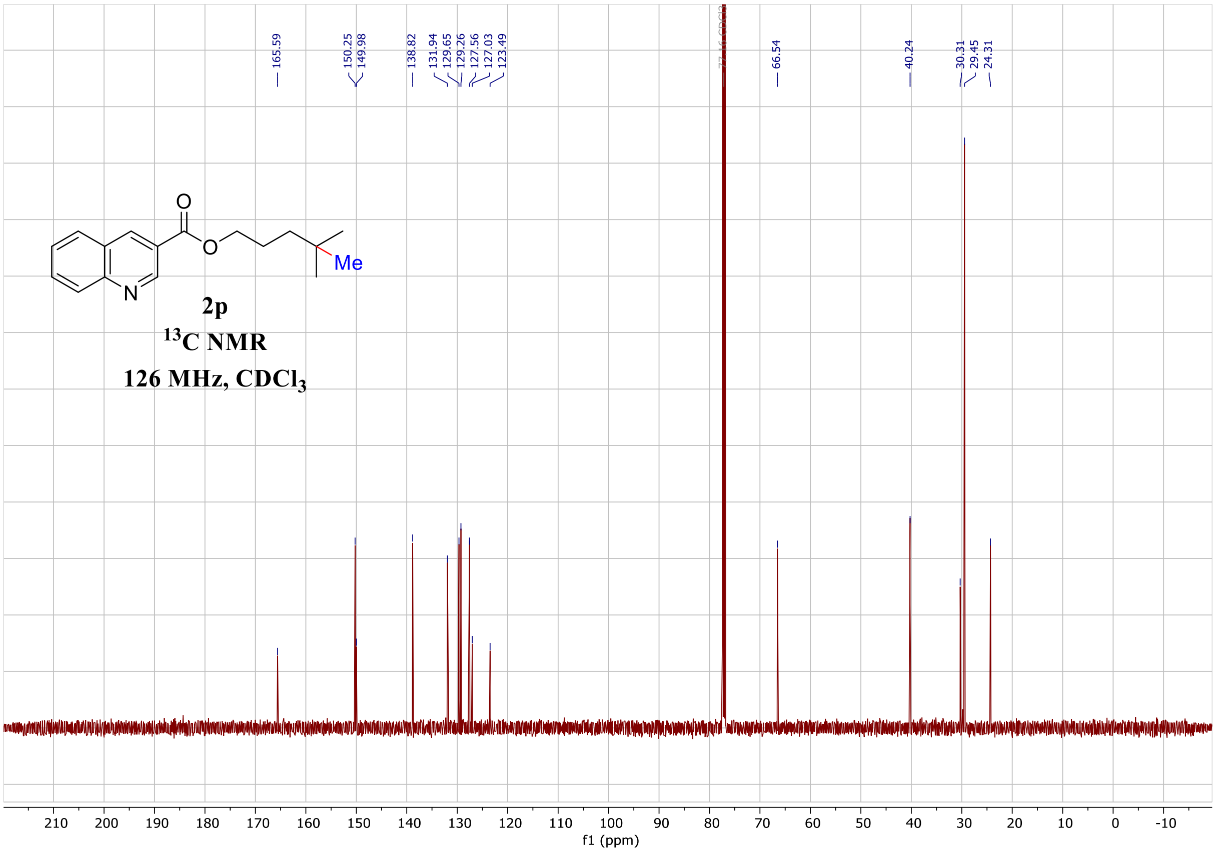
2p
^{13}C NMR
126 MHz, $CDCl_3$
f1 (ppm)

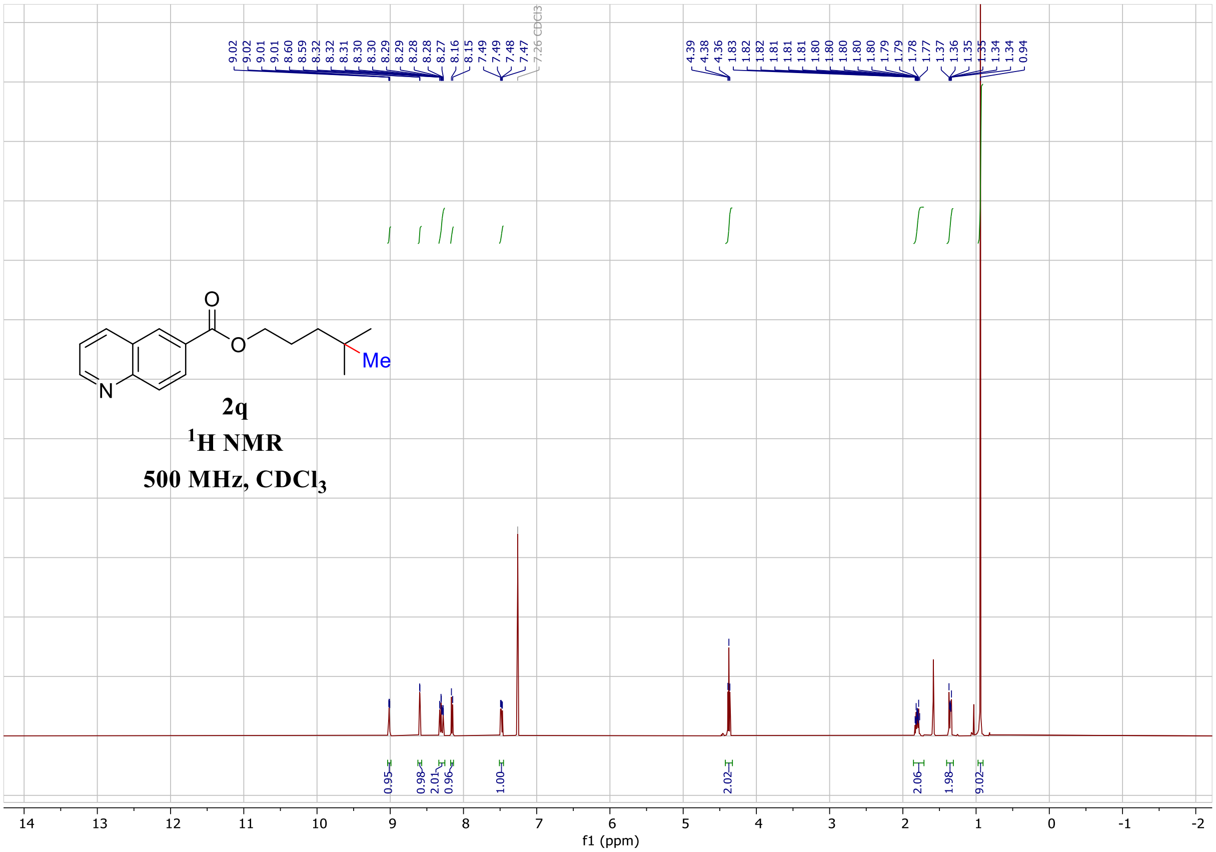
9.02 9.02 9.01 9.01 8.60 8.59 8.32 8.32 8.31 8.30 8.30 8.29 8.29 8.28 8.28 8.27 8.16 8.15 7.49 7.49 7.48 7.47
7.26 CDCl3
4.39 4.38 4.36 1.83 1.82 1.82 1.81 1.81 1.81 1.80 1.80 1.80 1.80 1.80 1.79 1.79 1.78 1.77 1.37 1.36 1.35 1.35 1.34 1.34 0.94
0.95 0.98 2.01 0.96 1.00 2.02 2.06 1.98 9.02
O
O
N
Me
2q
1H NMR
500 MHz, CDCl3
14 13 12 11 10 9 8 7 6 5 4 3 2 1 0 -1 -2
f1 (ppm)

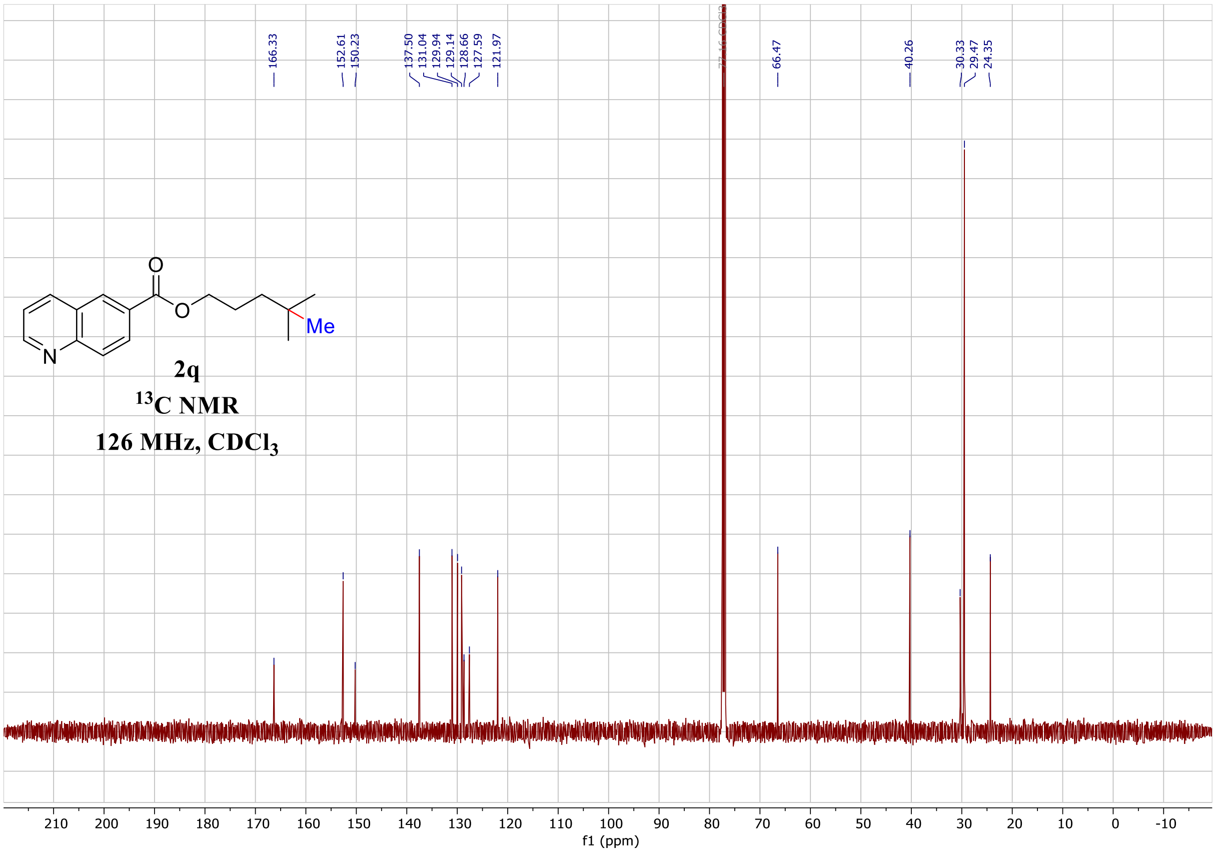
166.33
152.61 150.23
137.50 131.04 129.94 129.14 128.66 127.59 121.97
66.47
40.26
30.33 29.47 24.35
O
O
N
Me
2q
13C NMR
126 MHz, CDCl3
210 200 190 180 170 160 150 140 130 120 110 100 90 80 70 60 50 40 30 20 10 0 -10
f1 (ppm)

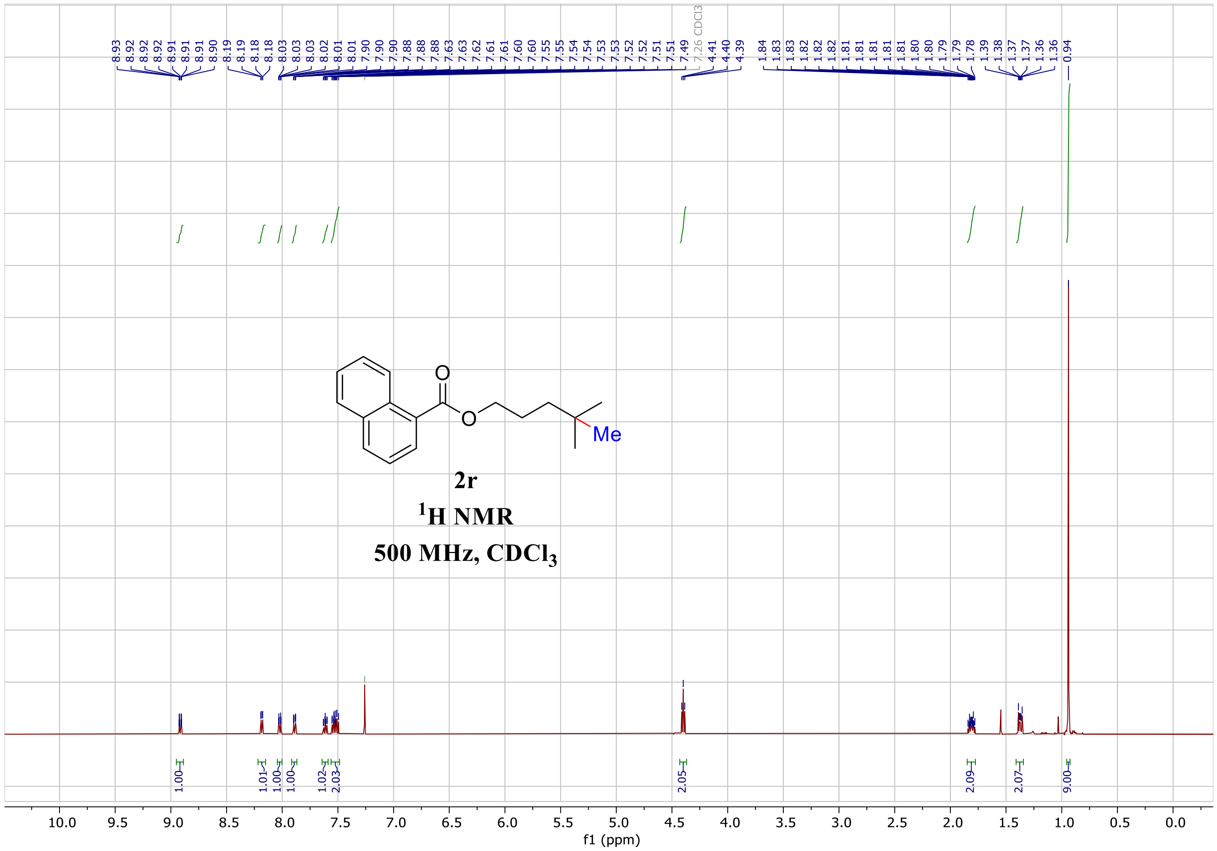
2r
^{1}H NMR
500 MHz, $CDCl_3$
f1 (ppm)

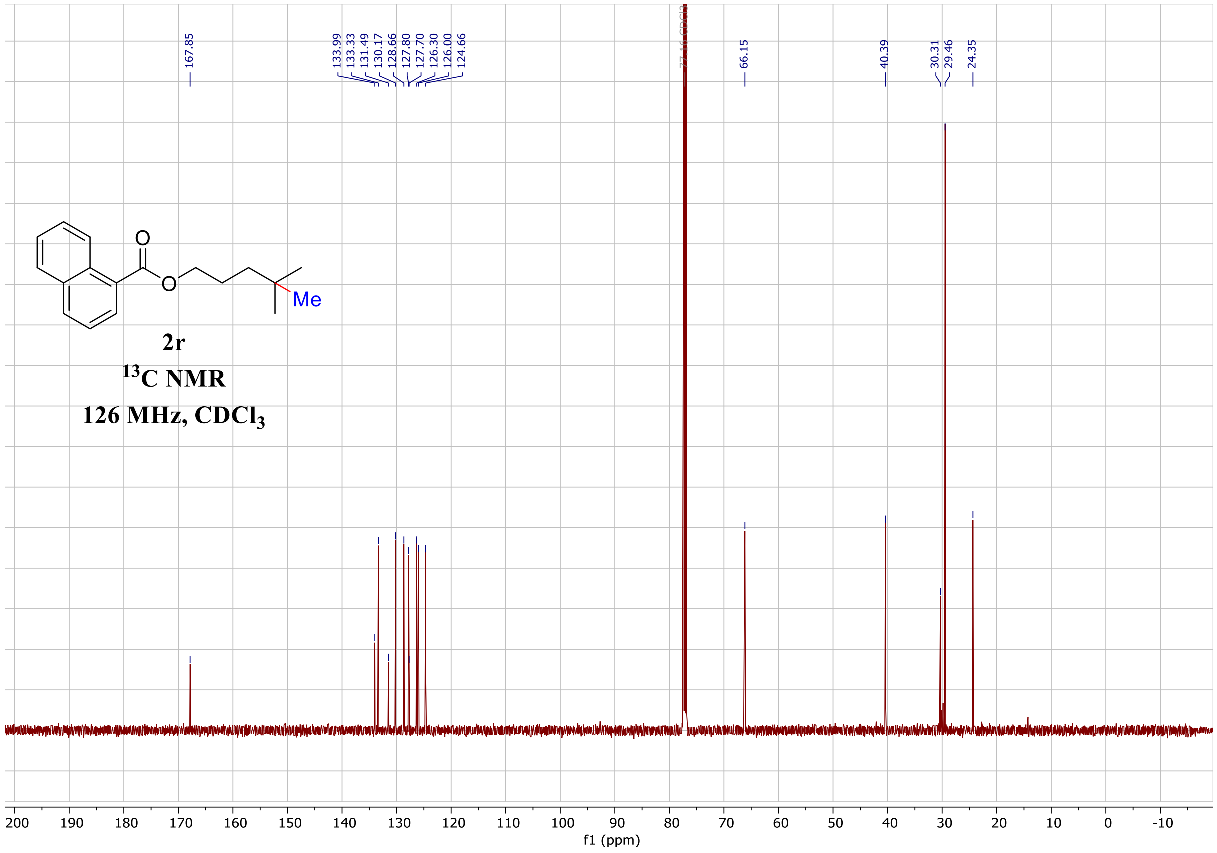
2r
^{13}C NMR
126 MHz, $CDCl_3$
167.85
133.99
133.33
131.49
130.17
128.66
127.80
127.70
126.30
126.00
124.66
66.15
40.39
30.31
29.46
24.35
f1 (ppm)

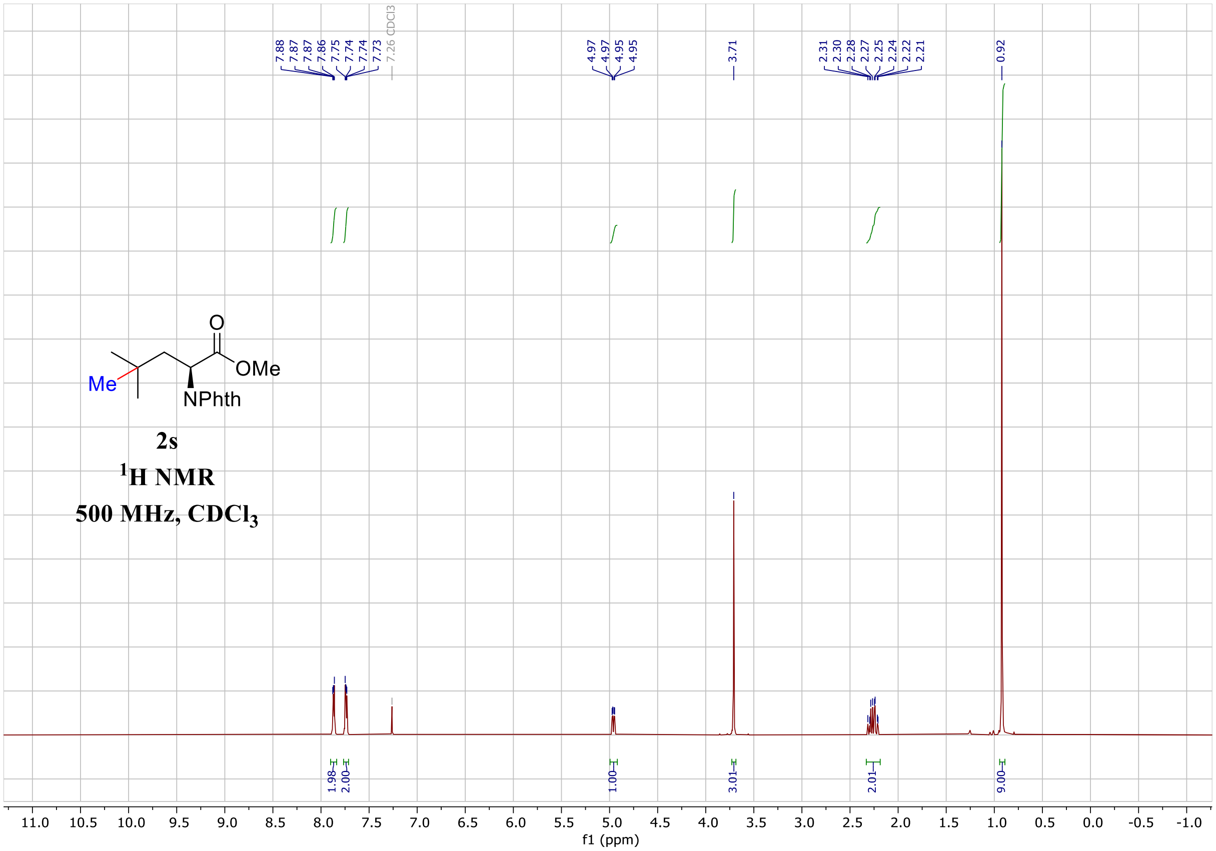
O
OMe
Me
NPhth
2s
1H NMR
500 MHz, CDCl3
7.88
7.87
7.87
7.86
7.75
7.74
7.74
7.73
7.26 CDCl3
4.97
4.97
4.95
4.95
3.71
2.31
2.30
2.28
2.27
2.25
2.24
2.22
2.21
0.92
1.98
2.00
1.00
3.01
2.01
9.00
f1 (ppm)

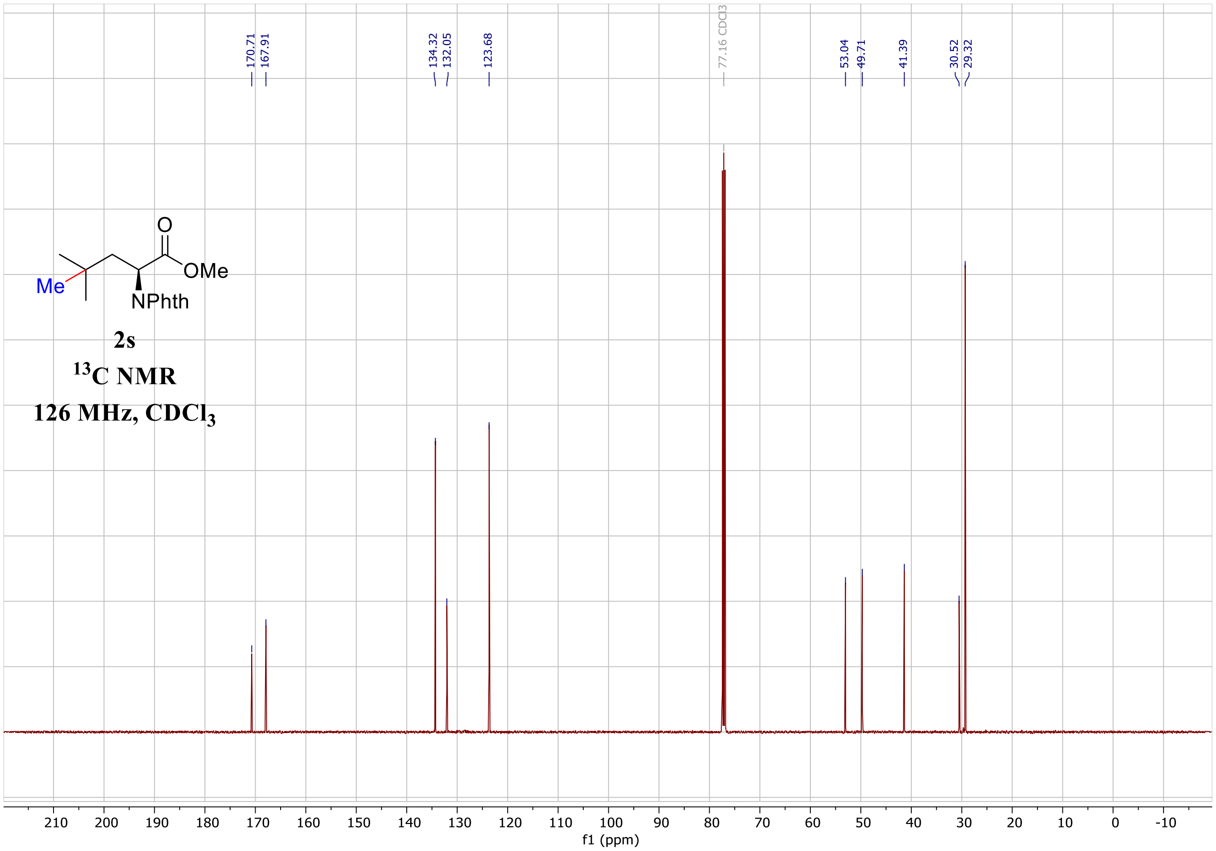
O
OMe
Me
NPhth
2s
13C NMR
126 MHz, CDCl3
170.71
167.91
134.32
132.05
123.68
77.16 CDCl3
53.04
49.71
41.39
30.52
29.32
f1 (ppm)

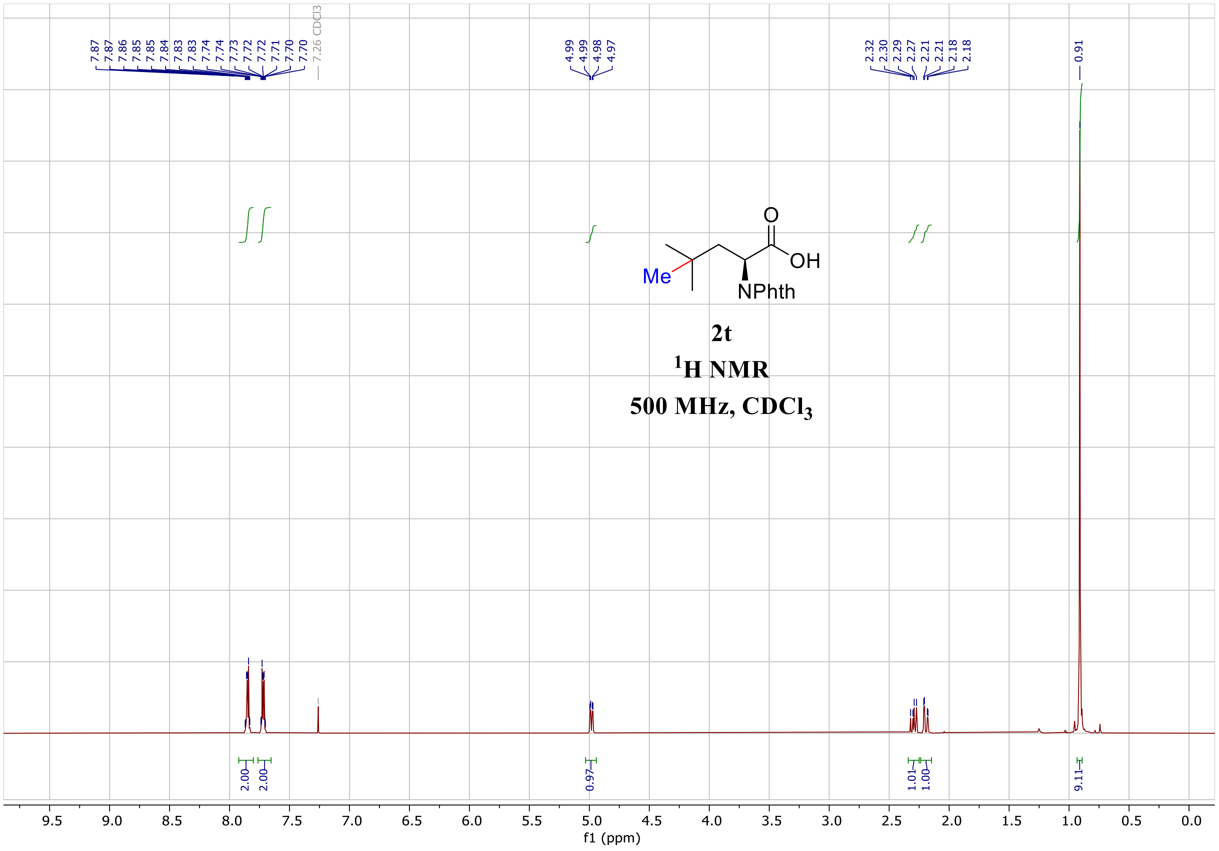
7.87 7.87 7.86 7.85 7.85 7.84 7.83 7.83 7.74 7.74 7.73 7.72 7.72 7.72 7.71 7.70 7.70
7.26 CDCl3
4.99 4.99 4.98 4.97
2.32 2.30 2.29 2.27 2.21 2.21 2.18 2.18
0.91
O
OH
Me
NPhth
2t
1H NMR
500 MHz, CDCl3
2.00
2.00
0.97
1.01
1.00
9.11
f1 (ppm)

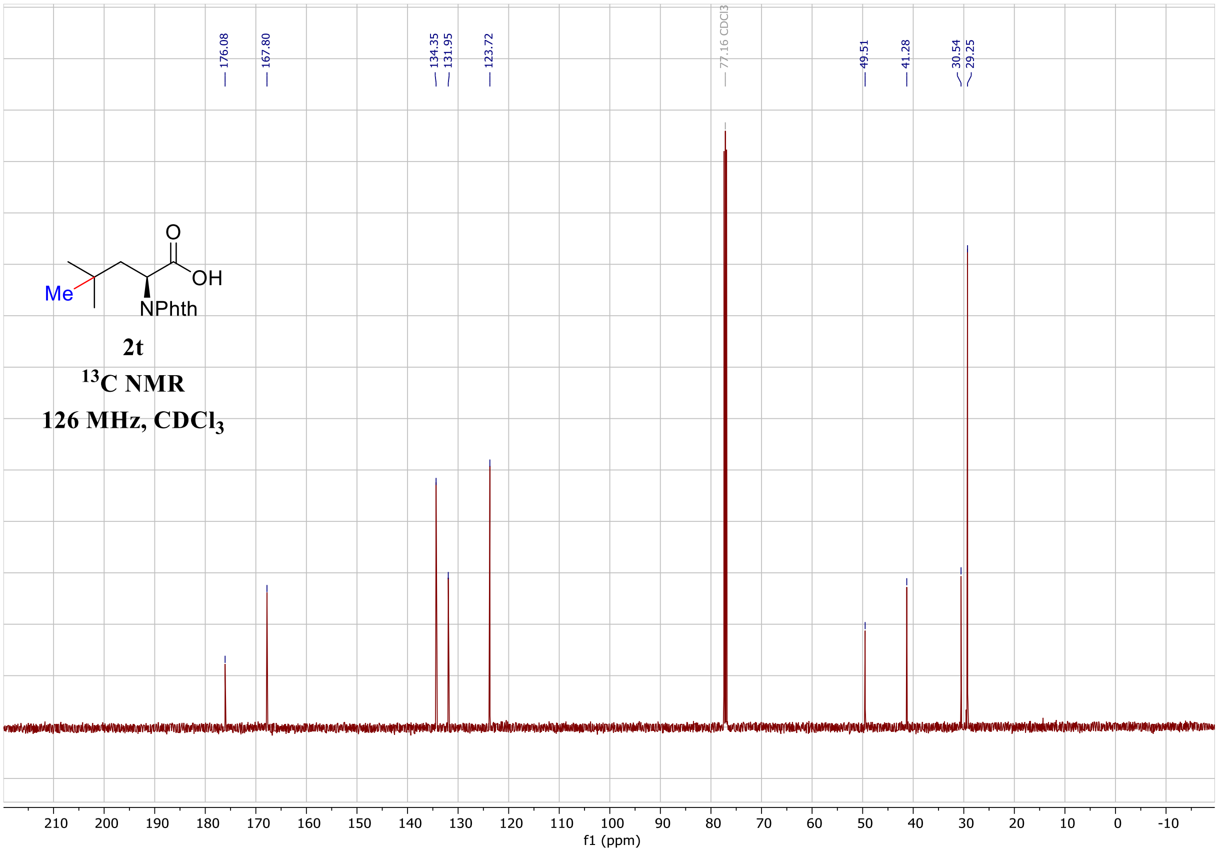
176.08
167.80
134.35
131.95
123.72
77.16 CDCl3
49.51
41.28
30.54
29.25
O
OH
Me
NPhth
2t
13C NMR
126 MHz, CDCl3
f1 (ppm)

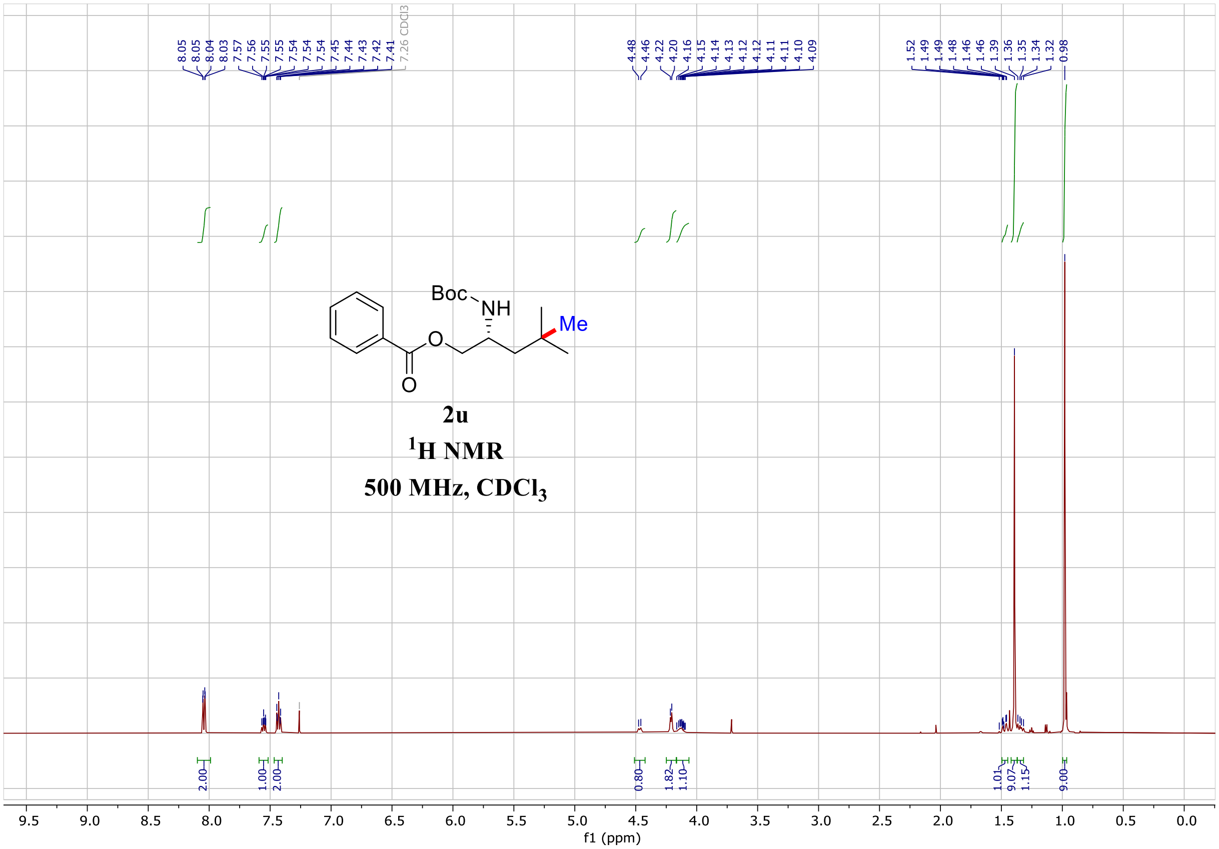

8.05
8.05
8.04
8.03
7.57
7.56
7.55
7.55
7.54
7.54
7.54
7.45
7.44
7.43
7.42
7.41
7.26 CDCl3
4.48
4.46
4.22
4.20
4.16
4.15
4.14
4.13
4.12
4.12
4.11
4.11
4.10
4.09
1.52
1.49
1.49
1.48
1.46
1.39
1.36
1.35
1.34
1.32
0.98
Boc
NH
Me
O
O
2u
^{1}H NMR
500 MHz, $CDCl_3$
2.00
1.00
2.00
0.80
1.82
1.10
1.01
9.07
1.15
9.00
9.5 9.0 8.5 8.0 7.5 7.0 6.5 6.0 5.5 5.0 4.5 4.0 3.5 3.0 2.5 2.0 1.5 1.0 0.5 0.0
f1 (ppm)

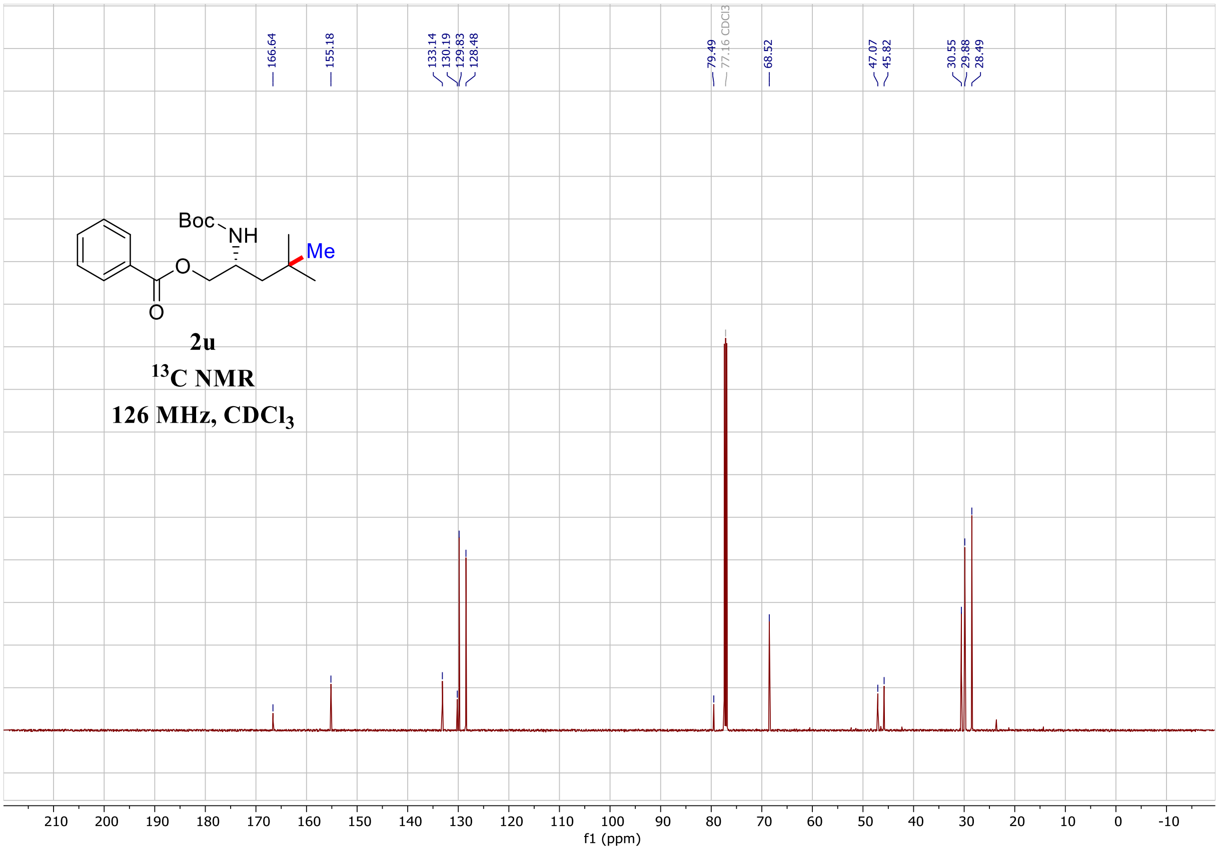

166.64
155.18
133.14
130.19
129.83
128.48
79.49
77.16 CDCl3
68.52
47.07
45.82
30.55
29.88
28.49
Boc
NH
Me
O
O
2u
^{13}C NMR
126 MHz, $CDCl_3$
210 200 190 180 170 160 150 140 130 120 110 100 90 80 70 60 50 40 30 20 10 0 -10
f1 (ppm)

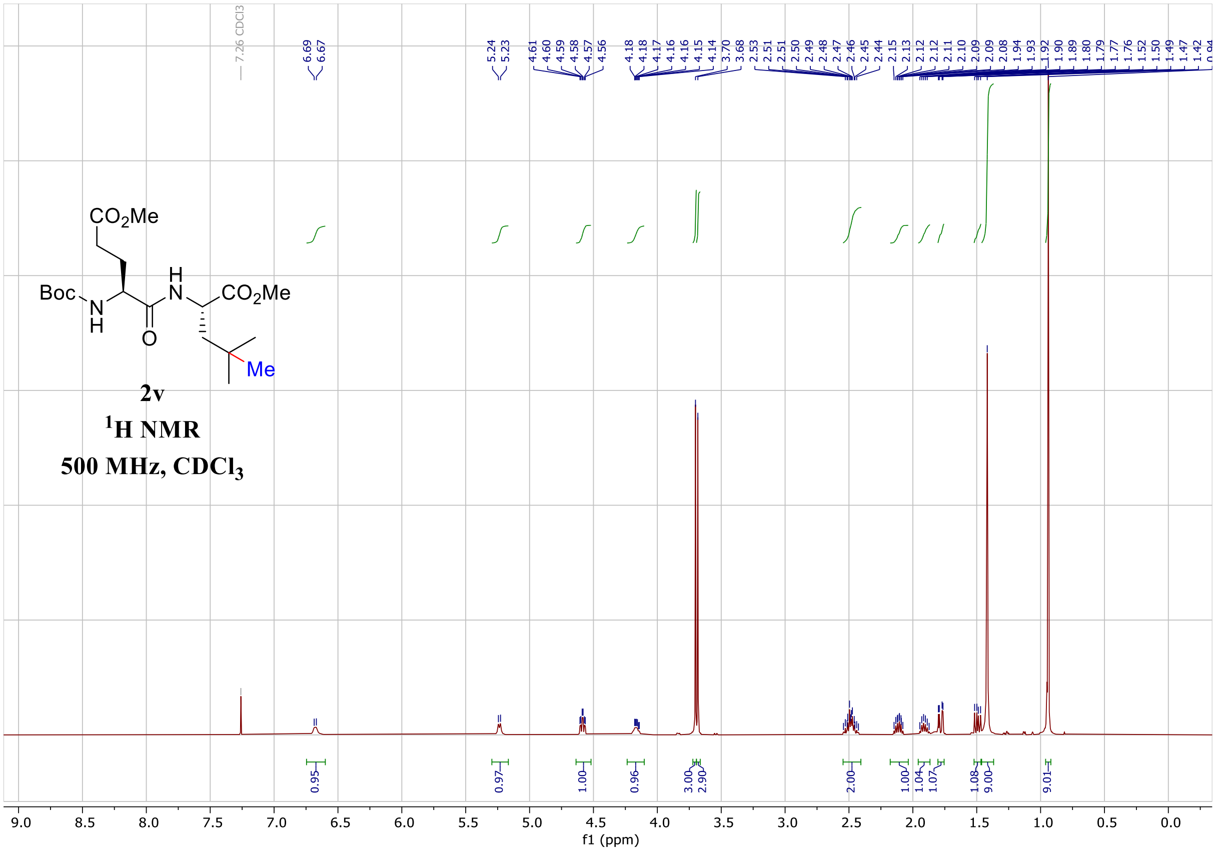
2v
^{1}H NMR
500 MHz, $CDCl_3$
f1 (ppm)

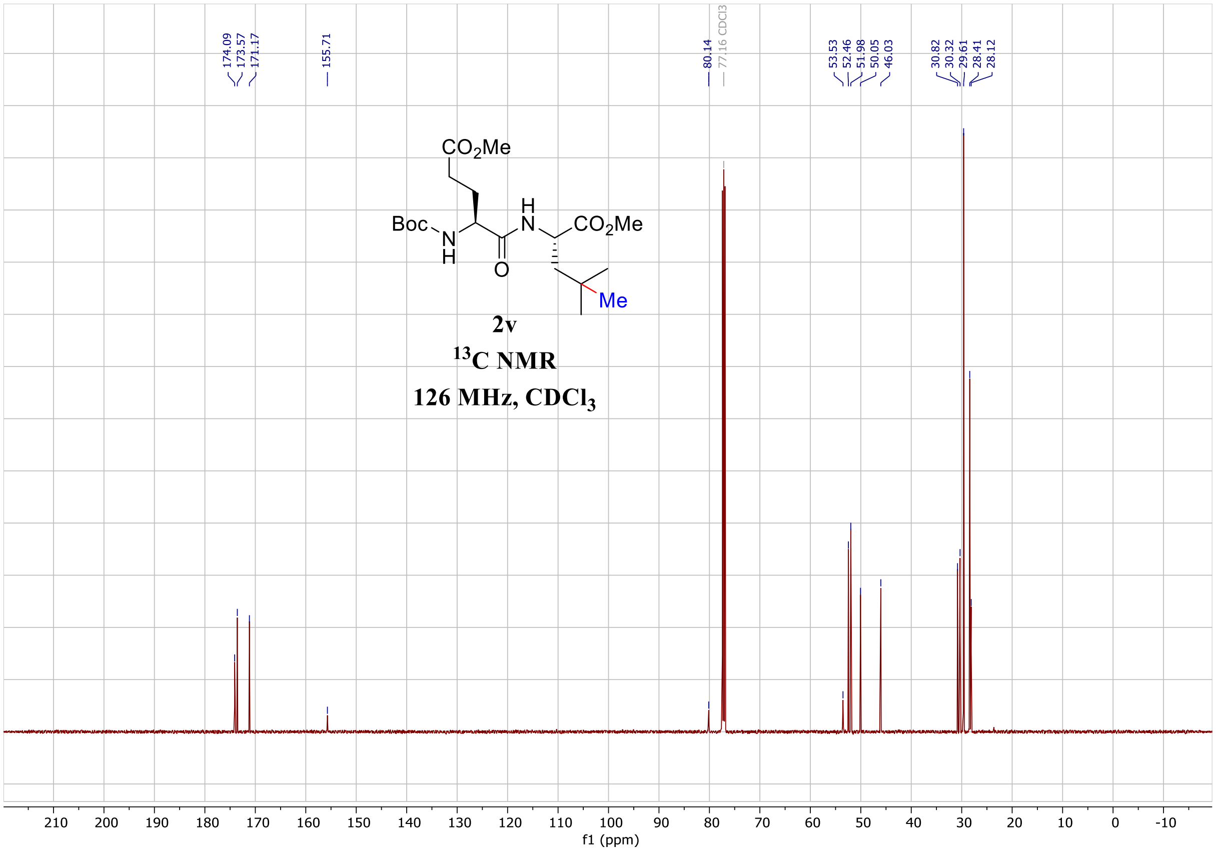
2v
^{13}C NMR
126 MHz, $CDCl_3$
f1 (ppm)

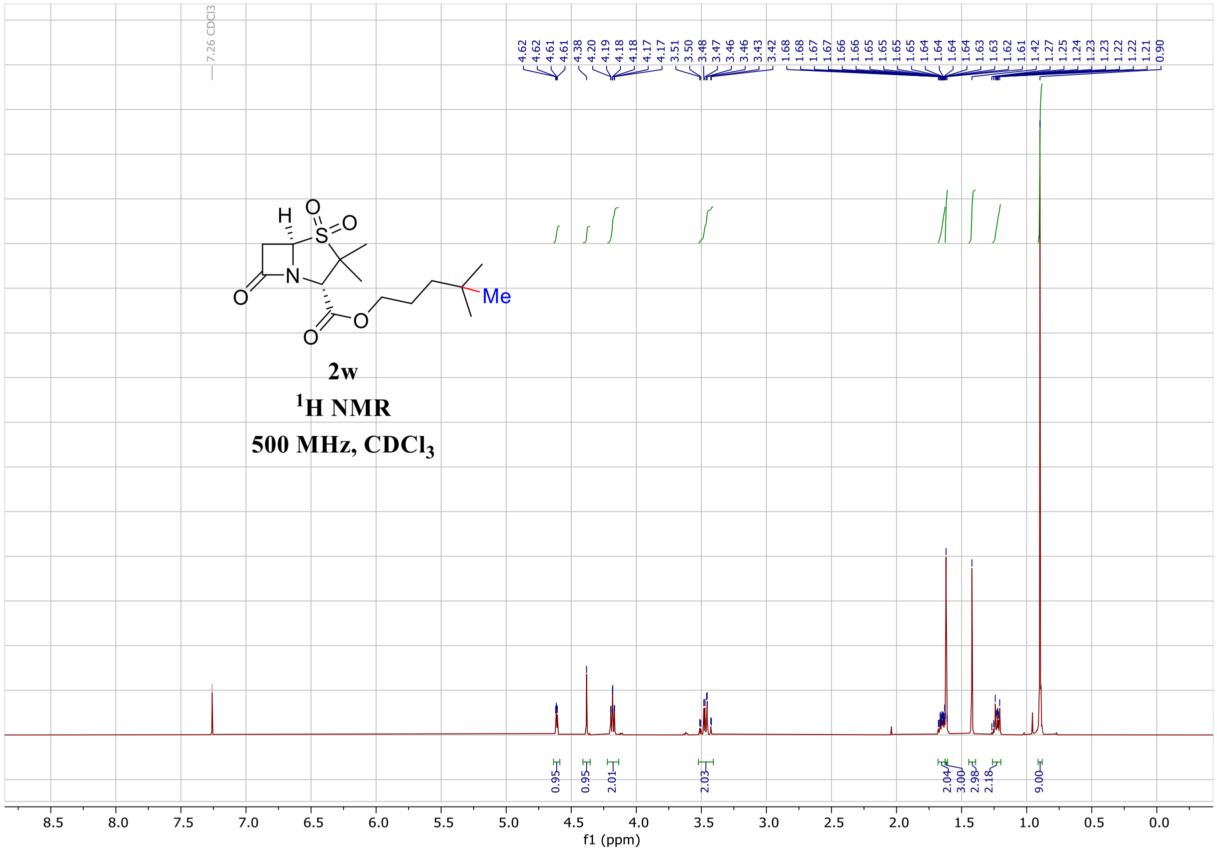
2w
^{1}H NMR
500 MHz, CDCl$_3$
f1 (ppm)

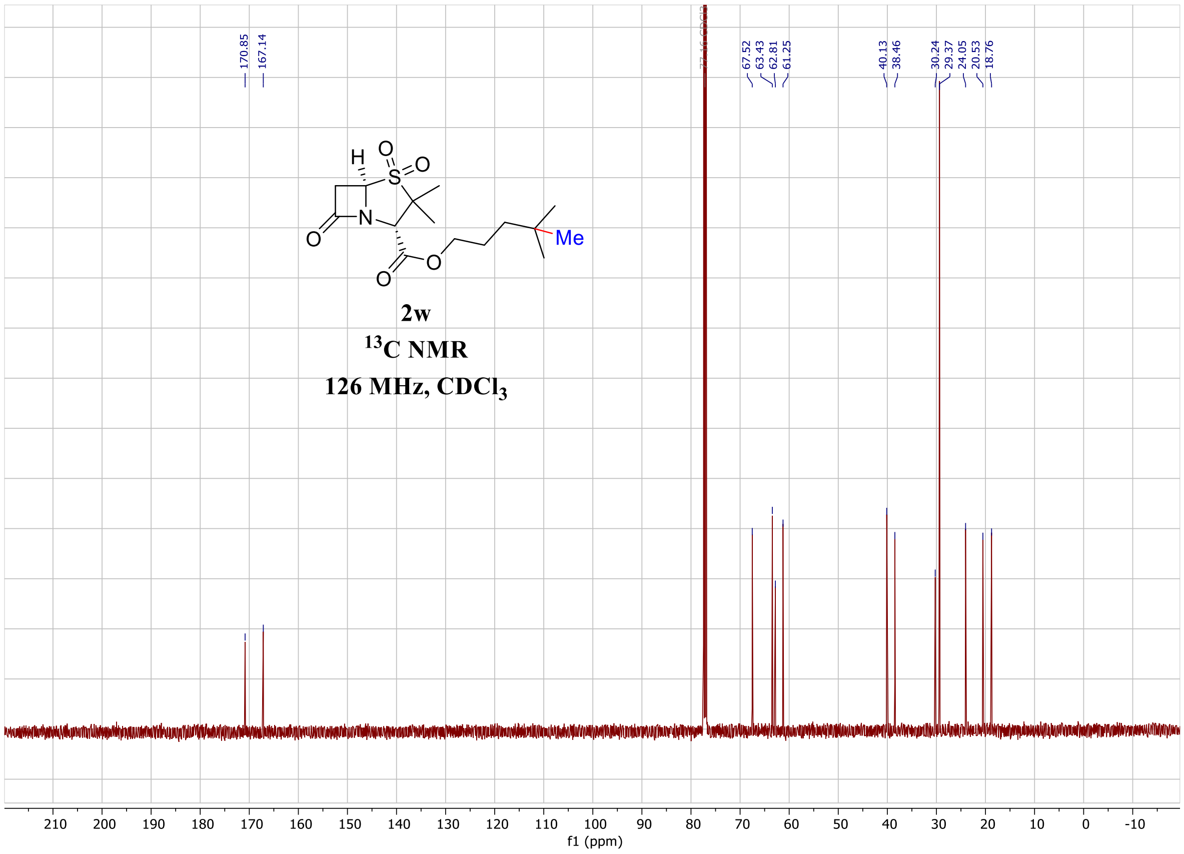
2w
^{13}C NMR
126 MHz, CDCl$_3$
f1 (ppm)

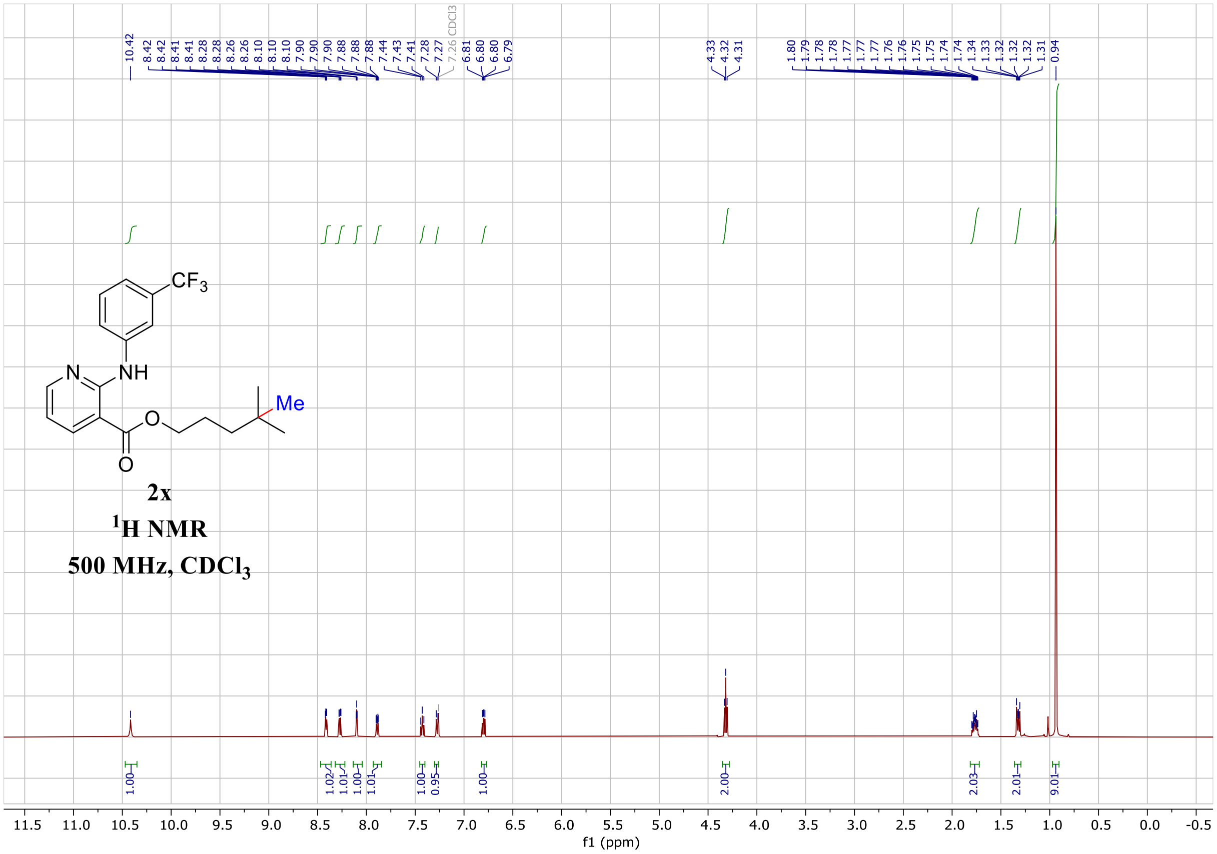

CF3
N
NH
O
Me
O
2x
1H NMR
500 MHz, CDCl3
f1 (ppm)

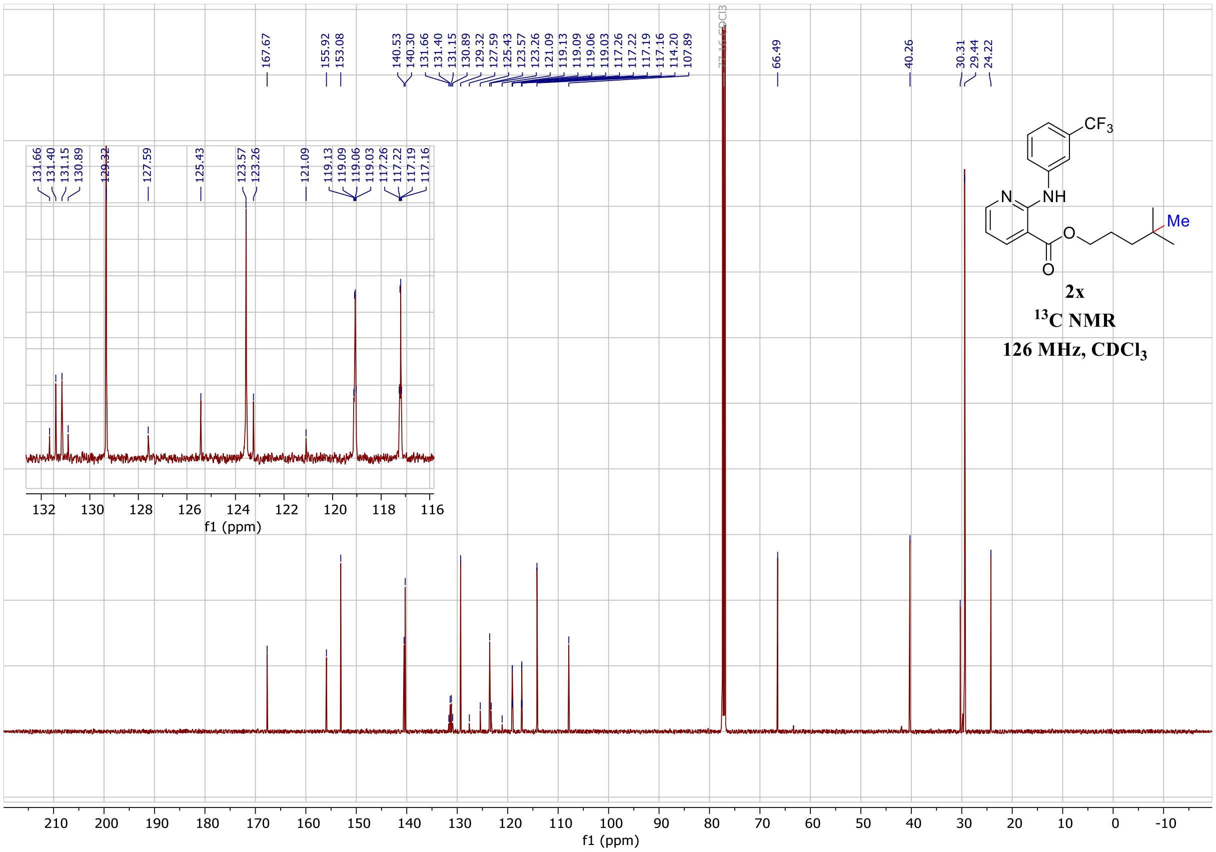

CF3
N
NH
O
Me
O
2x
13C NMR
126 MHz, CDCl3
f1 (ppm)

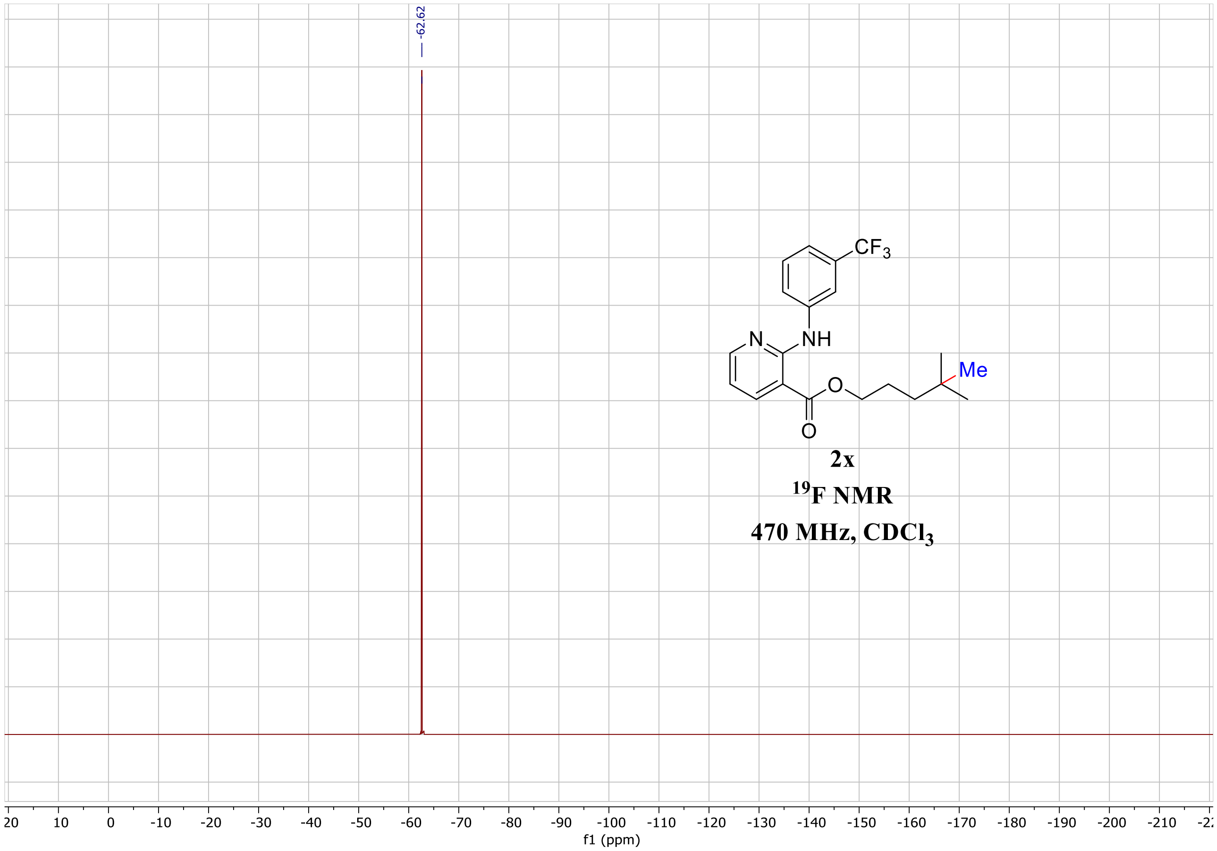
-62.62
CF3
NH
N
O
O
Me
2x
19F NMR
470 MHz, CDCl3
f1 (ppm)

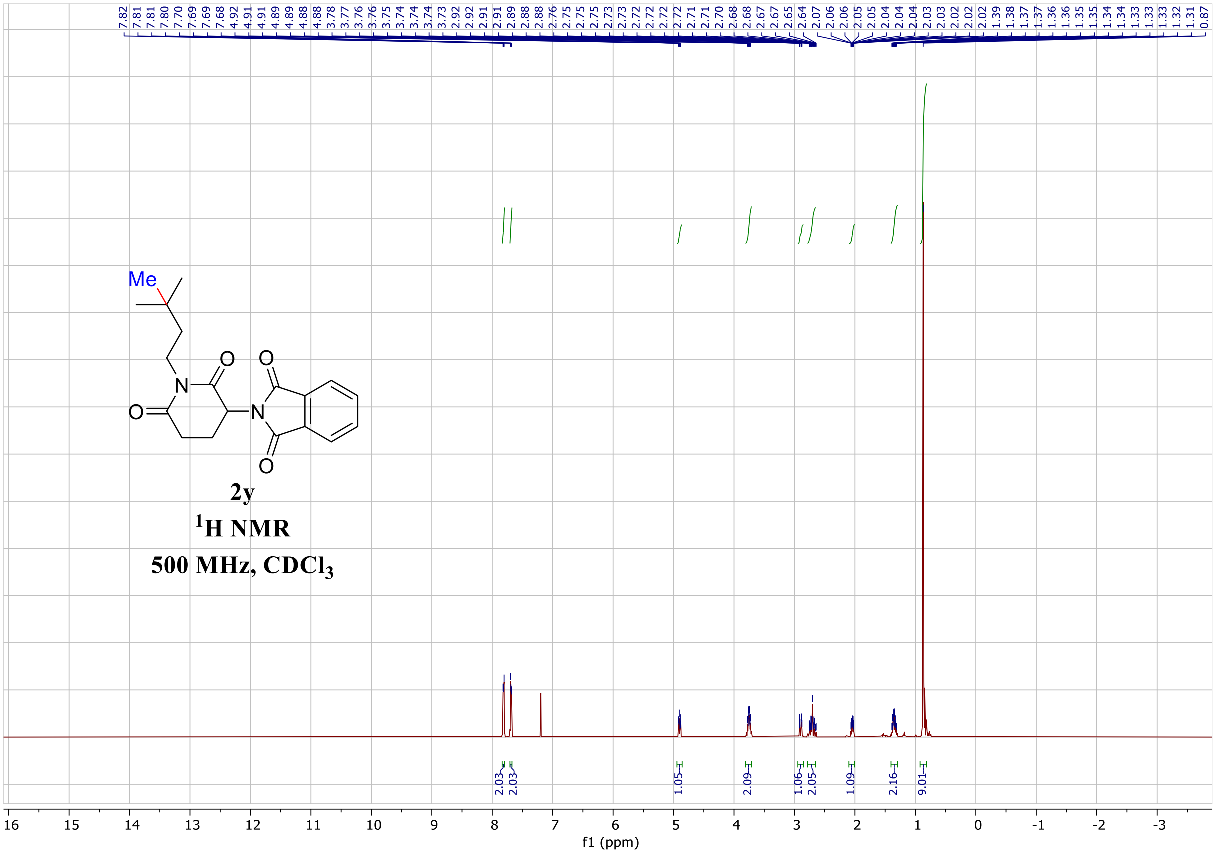
7.82 7.81 7.81 7.80 7.70 7.69 7.69 7.68 4.92 4.91 4.91 4.89 4.89 4.88 4.88 3.78 3.77 3.76 3.75 3.74 3.74 3.74 3.73 2.92 2.92 2.91 2.91 2.89 2.88 2.88 2.76 2.75 2.75 2.73 2.72 2.72 2.72 2.72 2.71 2.71 2.70 2.68 2.68 2.67 2.65 2.64 2.07 2.06 2.06 2.05 2.05 2.04 2.04 2.04 2.03 2.03 2.02 2.02 2.02 1.39 1.38 1.37 1.37 1.36 1.36 1.35 1.35 1.34 1.34 1.33 1.33 1.33 1.32 1.31 0.87
Me
N
O
O
N
O
O
2y
1H NMR
500 MHz, CDCl3
2.03 2.03 1.05 2.09 1.06 2.05 1.09 2.16 9.01
f1 (ppm)

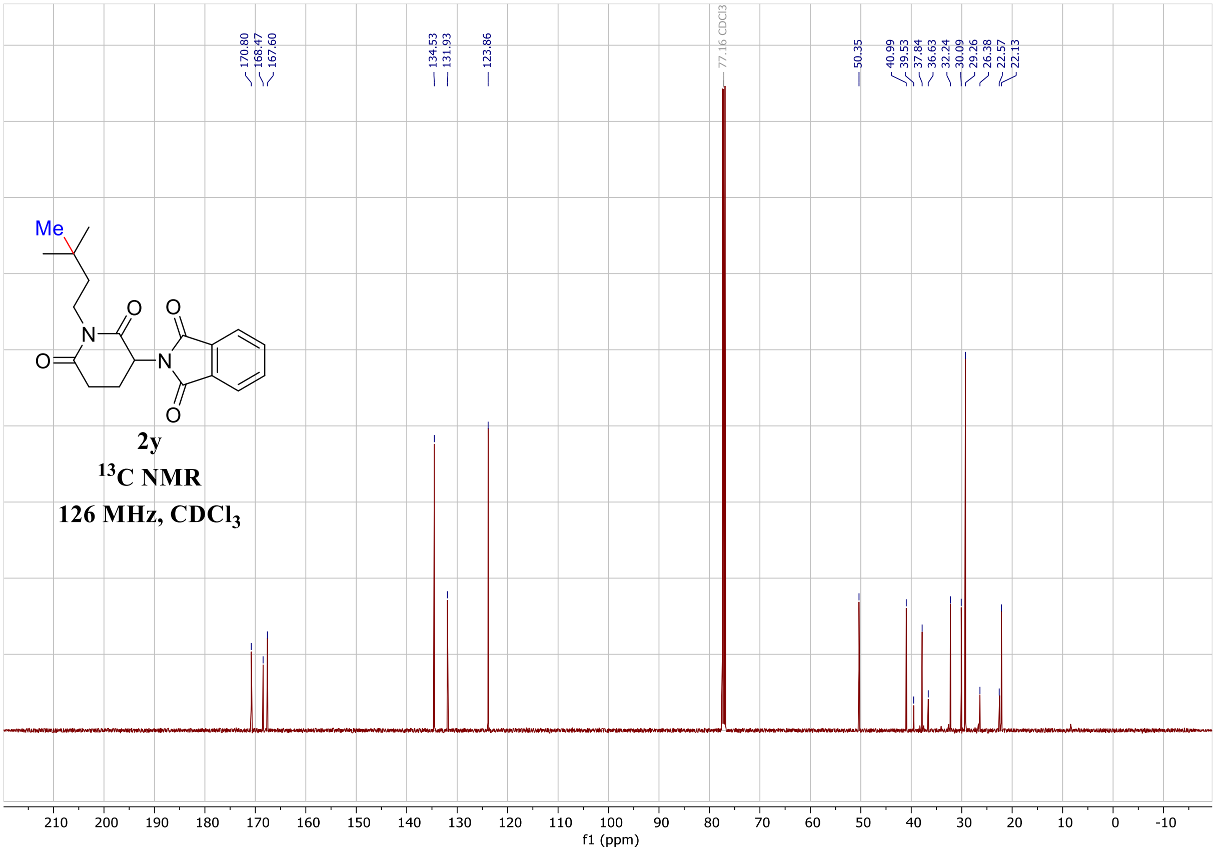
170.80
168.47
167.60
134.53
131.93
123.86
77.16 CDCl3
50.35
40.99
39.53
37.84
36.63
32.24
30.09
29.26
26.38
22.57
22.13
Me
2y
13C NMR
126 MHz, CDCl3
f1 (ppm)

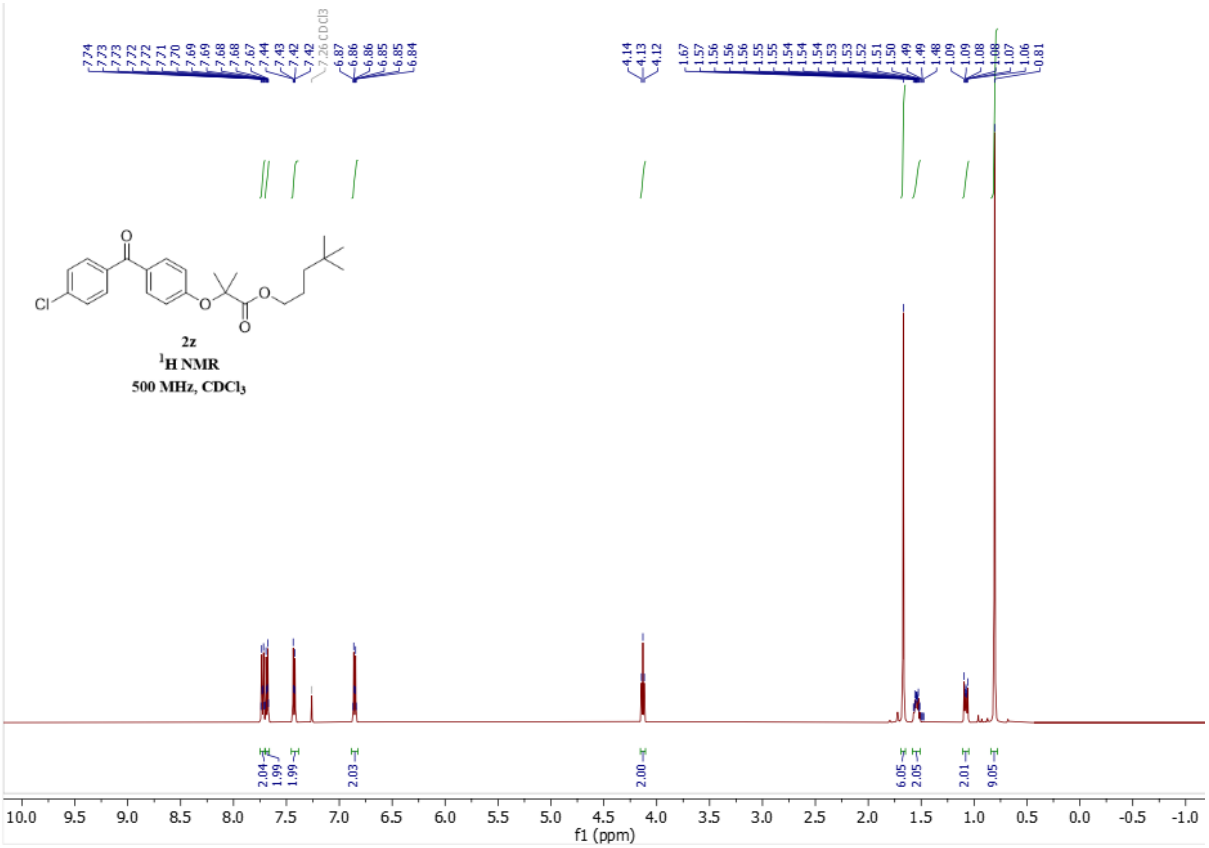
7.26 CDCl3
Cl
2z
1H NMR
500 MHz, CDCl3
2.04
1.99
1.99
2.03
2.00
6.05
2.05
2.01
9.05
f1 (ppm)

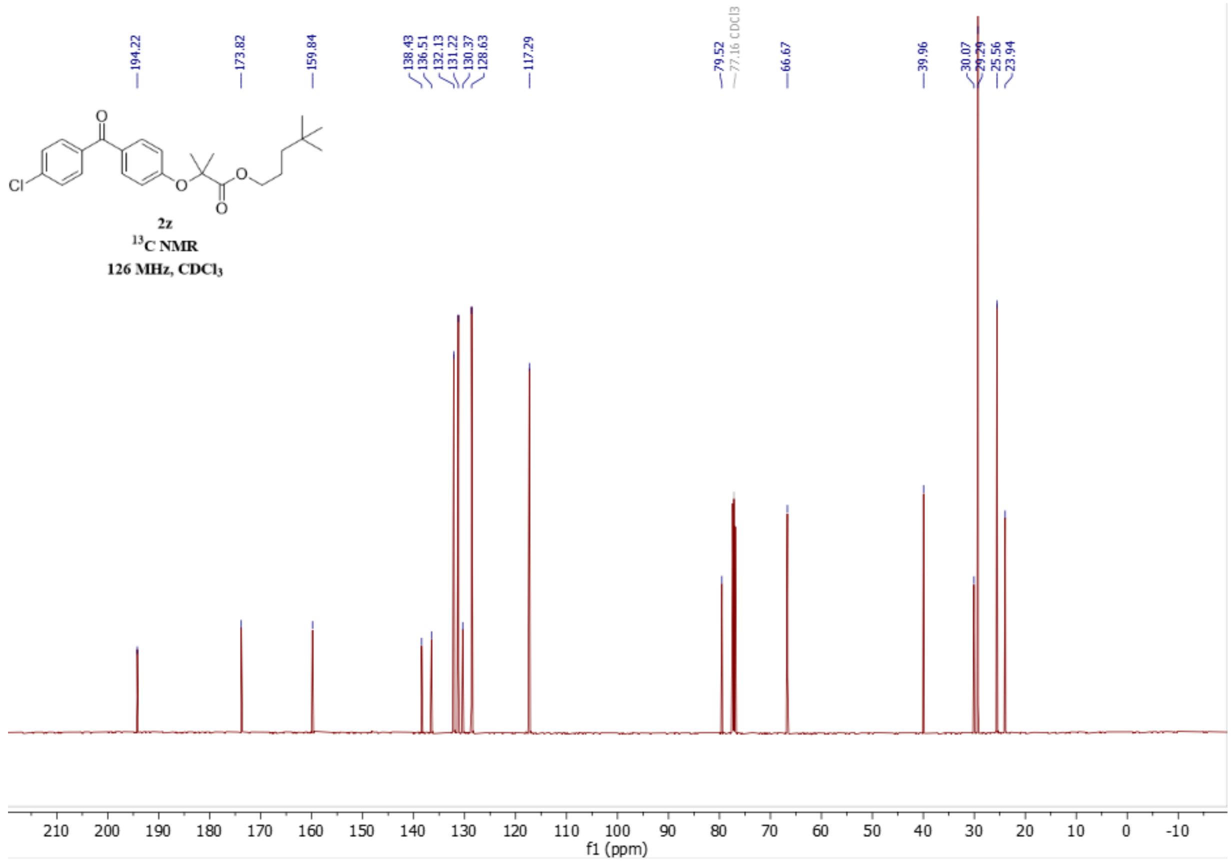
2z
^{13}C NMR
126 MHz, CDCl$_3$
f1 (ppm)

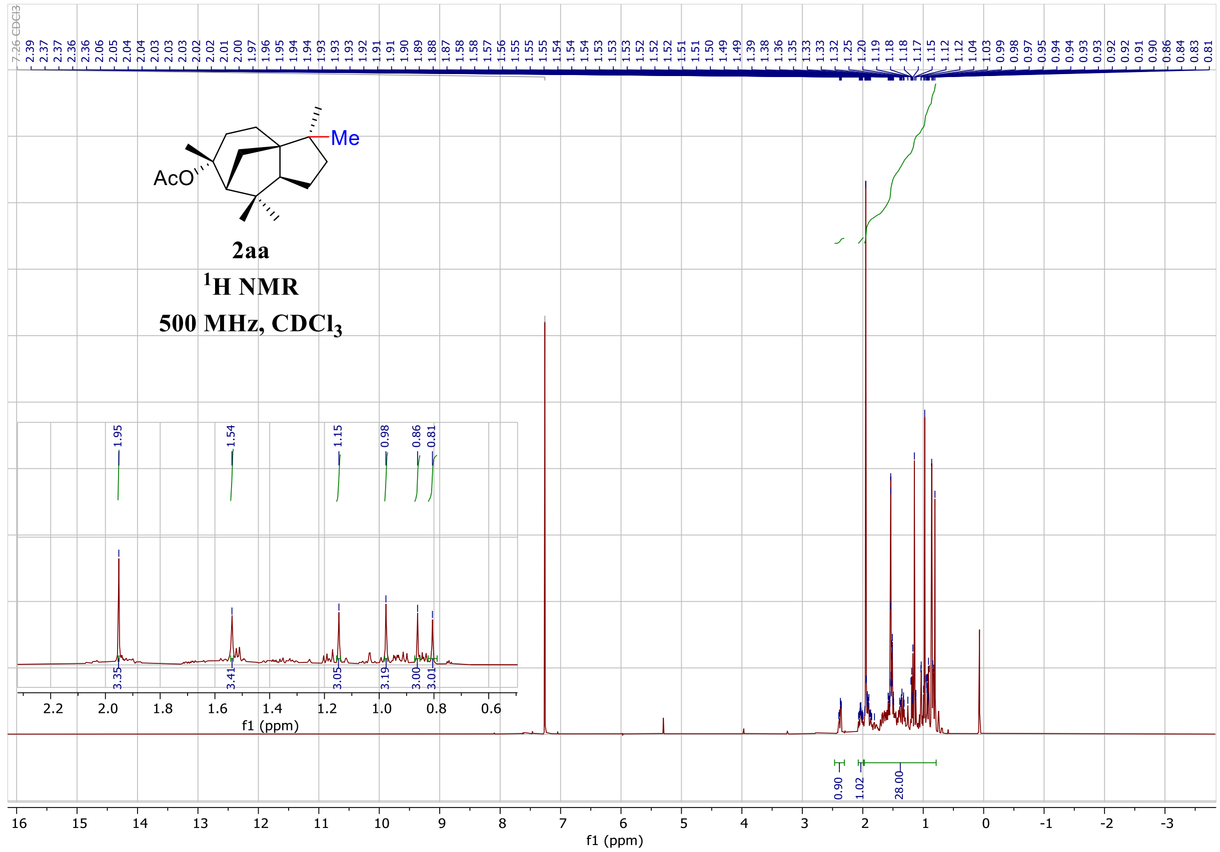
AcO
Me
2aa
^{1}H NMR
500 MHz, CDCl$_3$
f1 (ppm)

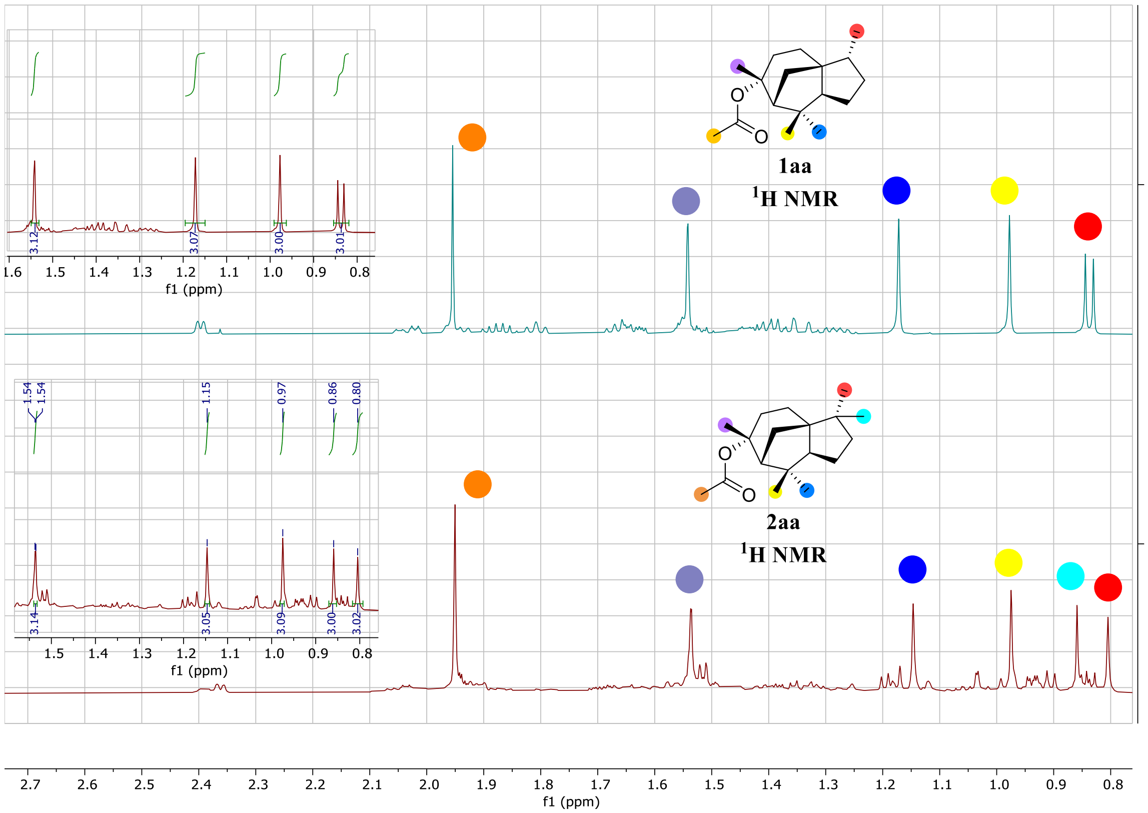
1aa
1H NMR
2aa
1H NMR
f1 (ppm)

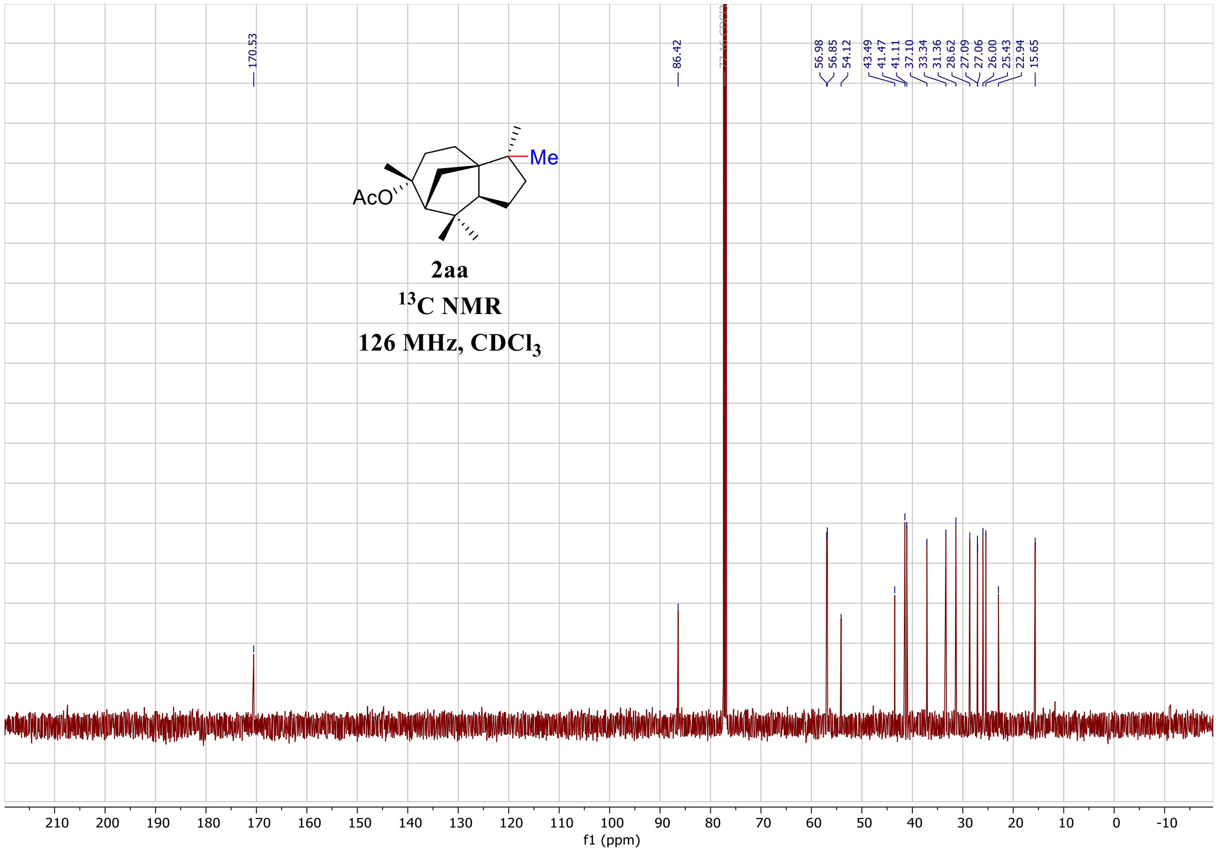
170.53
86.42
56.98
56.85
54.12
43.49
41.47
41.11
37.10
33.34
31.36
28.62
27.09
27.06
26.00
25.43
22.94
15.65
AcO
Me
2aa
13C NMR
126 MHz, CDCl3
f1 (ppm)

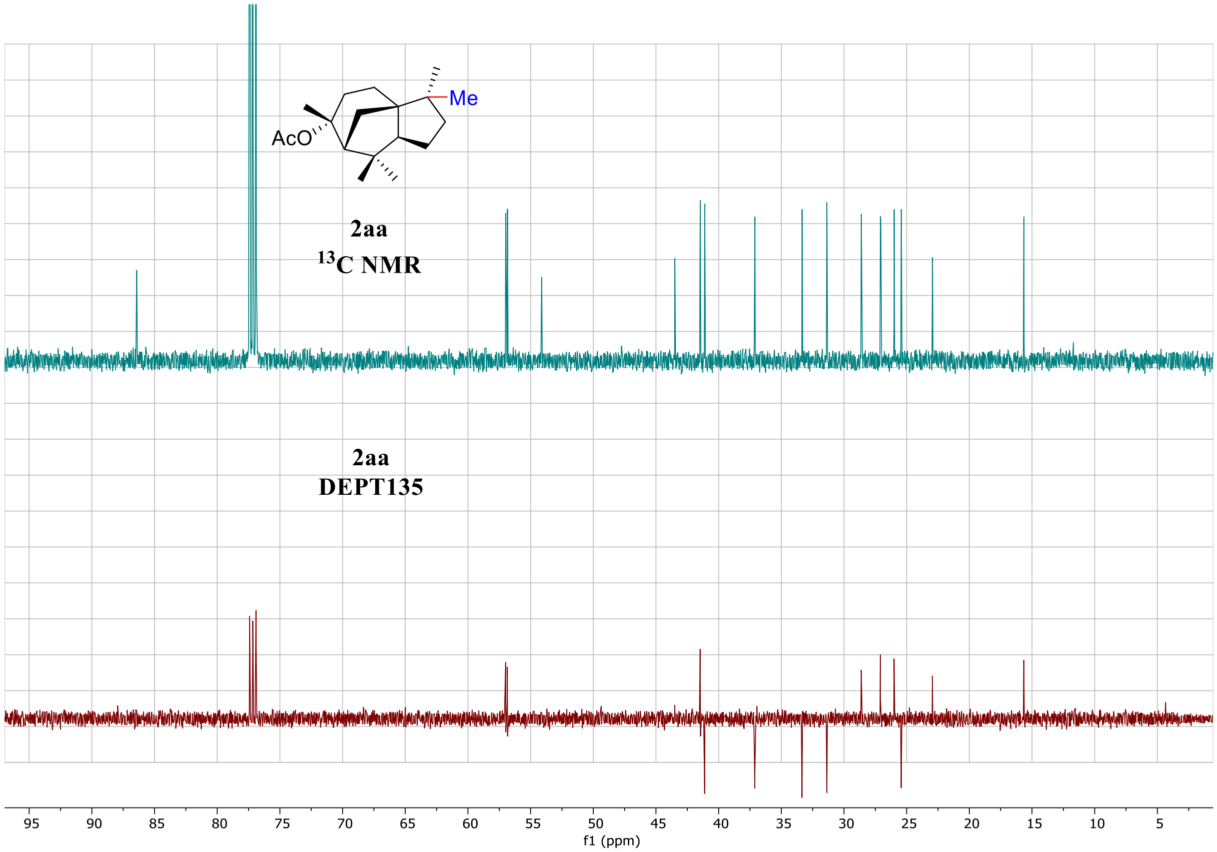
AcO
Me
2aa
13C NMR
2aa
DEPT135
95
90
85
80
75
70
65
60
55
50
45
40
35
30
25
20
15
10
5
f1 (ppm)

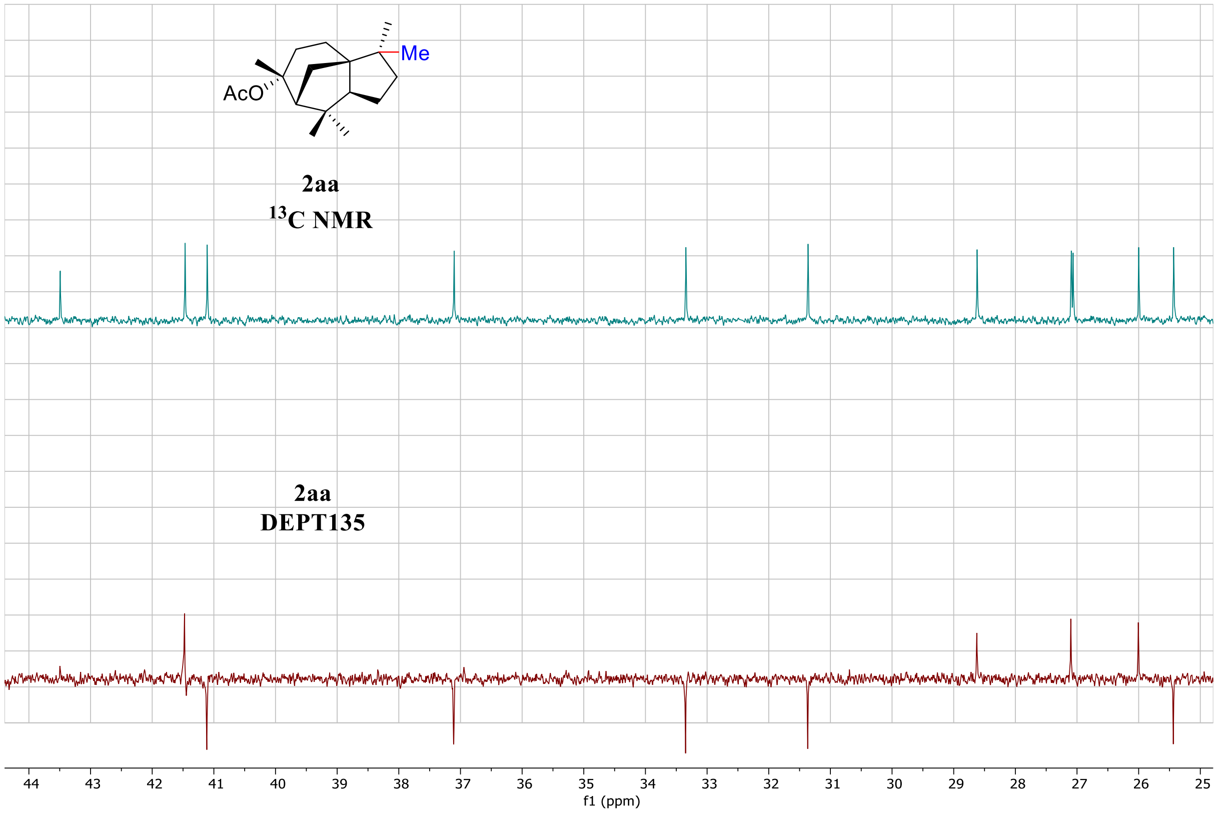
AcO
Me
2aa
13C NMR
2aa
DEPT135
44
43
42
41
40
39
38
37
36
35
34
33
32
31
30
29
28
27
26
25
f1 (ppm)

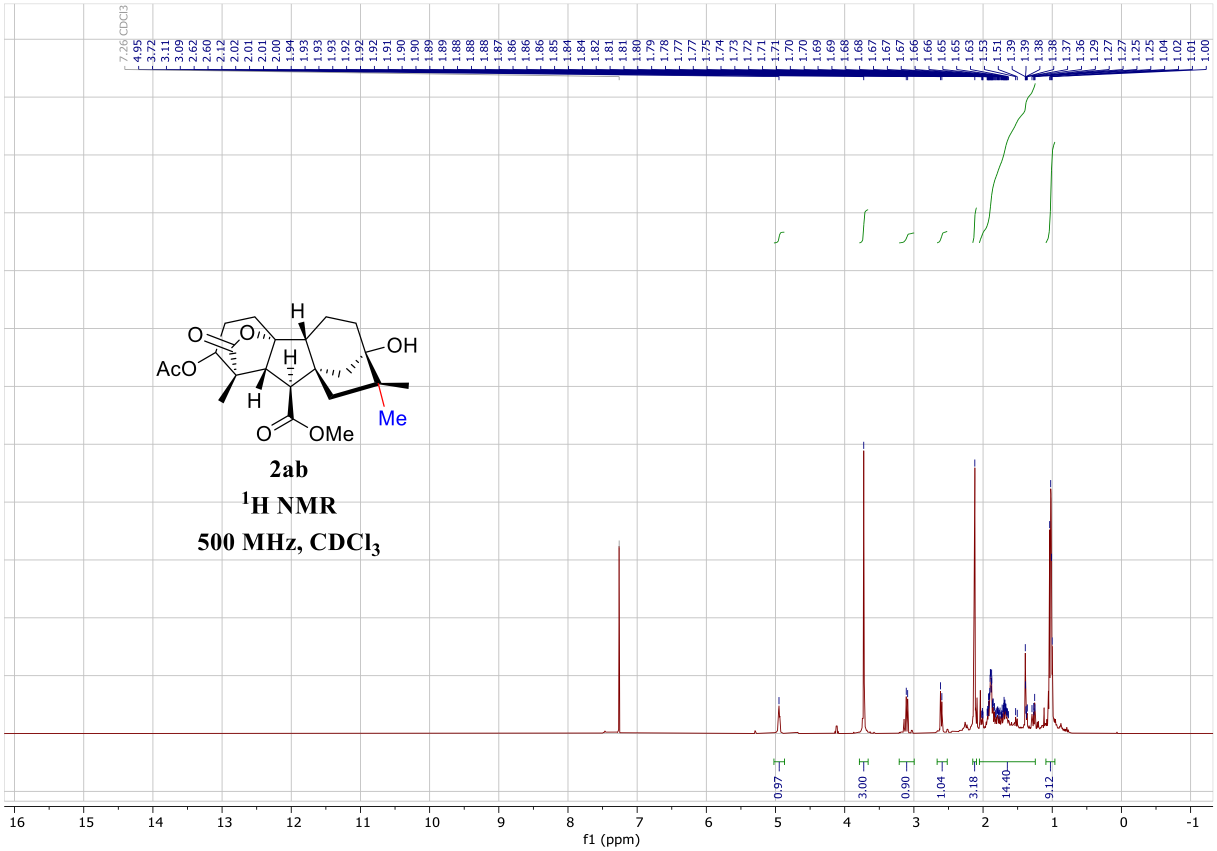

7.26 CDCl3
4.95
3.72
3.11
3.09
2.62
2.60
2.12
2.02
2.01
2.01
2.00
1.94
1.93
1.93
1.93
1.92
1.92
1.91
1.90
1.90
1.89
1.89
1.88
1.88
1.88
1.87
1.86
1.86
1.86
1.85
1.84
1.84
1.82
1.81
1.81
1.80
1.79
1.78
1.77
1.77
1.75
1.74
1.73
1.72
1.71
1.71
1.70
1.70
1.69
1.69
1.68
1.67
1.67
1.67
1.66
1.65
1.65
1.63
1.53
1.51
1.39
1.39
1.38
1.37
1.36
1.29
1.27
1.25
1.25
1.04
1.02
1.01
1.00
O
O
H
OH
AcO
H
H
O
OMe
Me
2ab
1H NMR
500 MHz, CDCl3
0.97
3.00
0.90
1.04
3.18
14.40
9.12
16 15 14 13 12 11 10 9 8 7 6 5 4 3 2 1 0 -1
f1 (ppm)

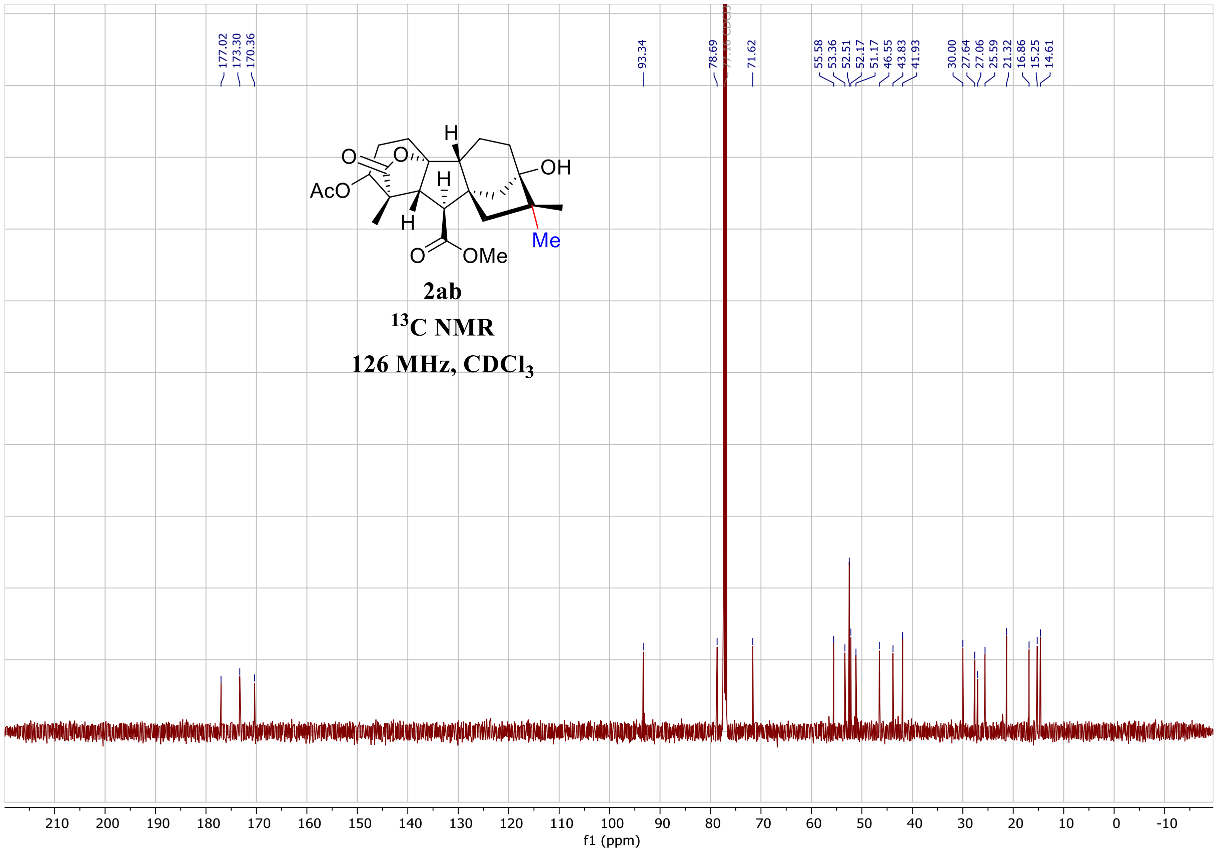

177.02
173.30
170.36
93.34
78.69
77.16 CDCl3
71.62
55.58
53.36
52.51
52.17
51.17
46.55
43.83
41.93
30.00
27.64
27.06
25.59
21.32
16.86
15.25
14.61
O
O
H
OH
AcO
H
H
O
OMe
Me
2ab
13C NMR
126 MHz, CDCl3
210 200 190 180 170 160 150 140 130 120 110 100 90 80 70 60 50 40 30 20 10 0 -10
f1 (ppm)

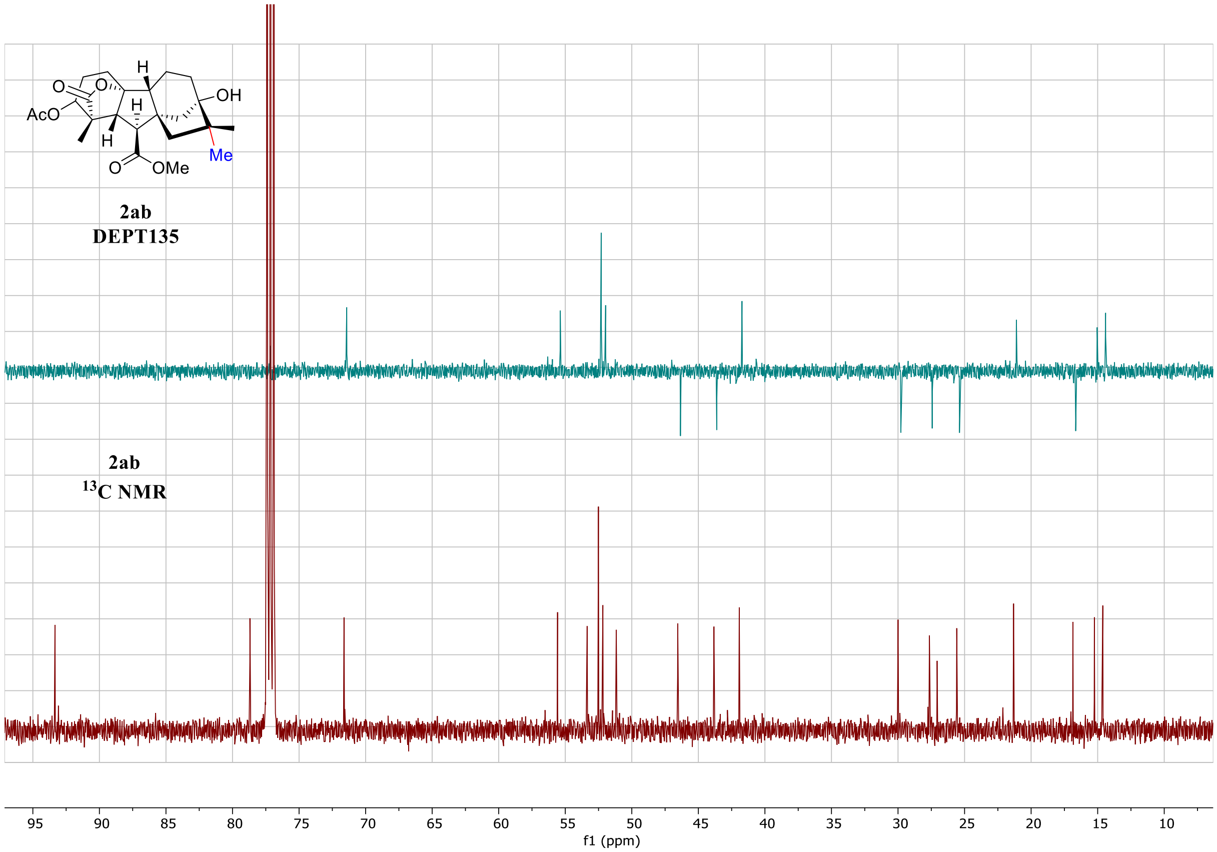
AcO
OH
Me
OMe
2ab
DEPT135
2ab
13C NMR
f1 (ppm)

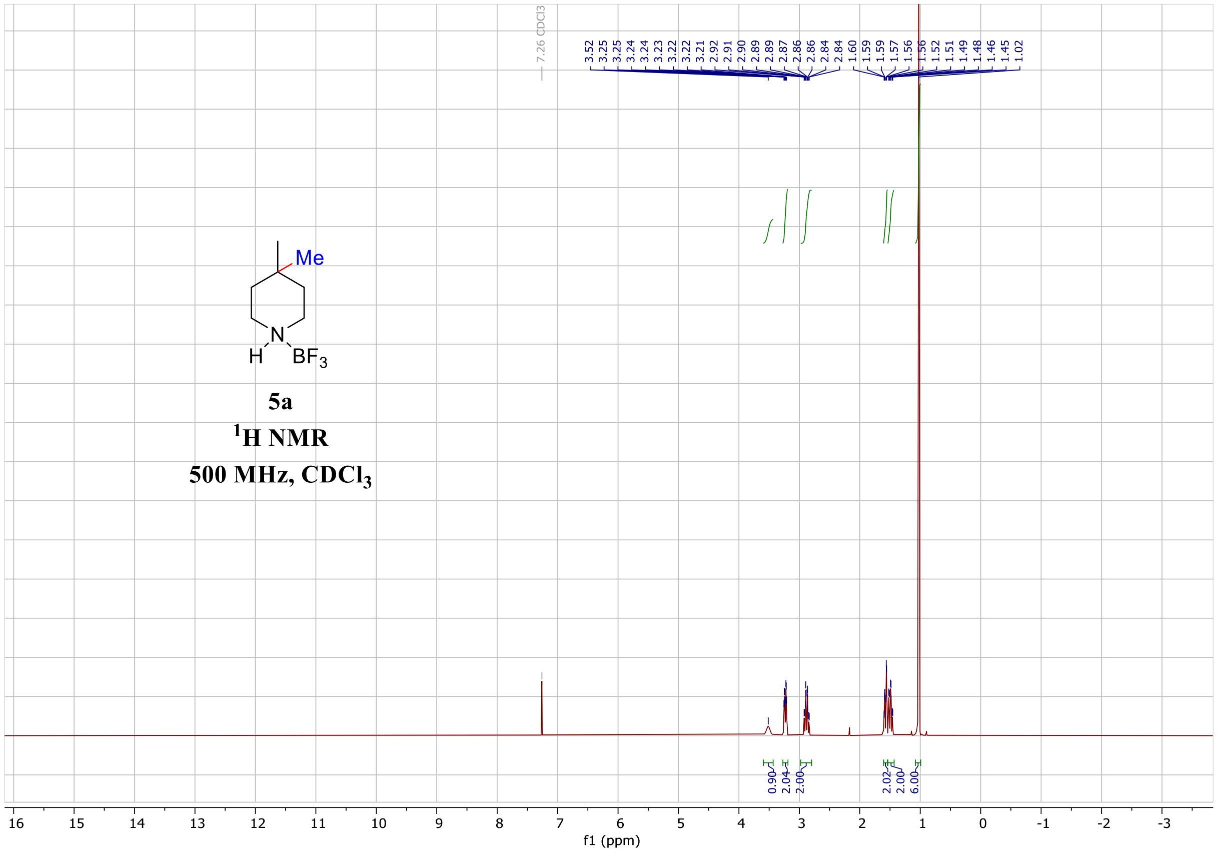
7.26 CDCl3
Me
BF3
5a
1H NMR
500 MHz, CDCl3
f1 (ppm)

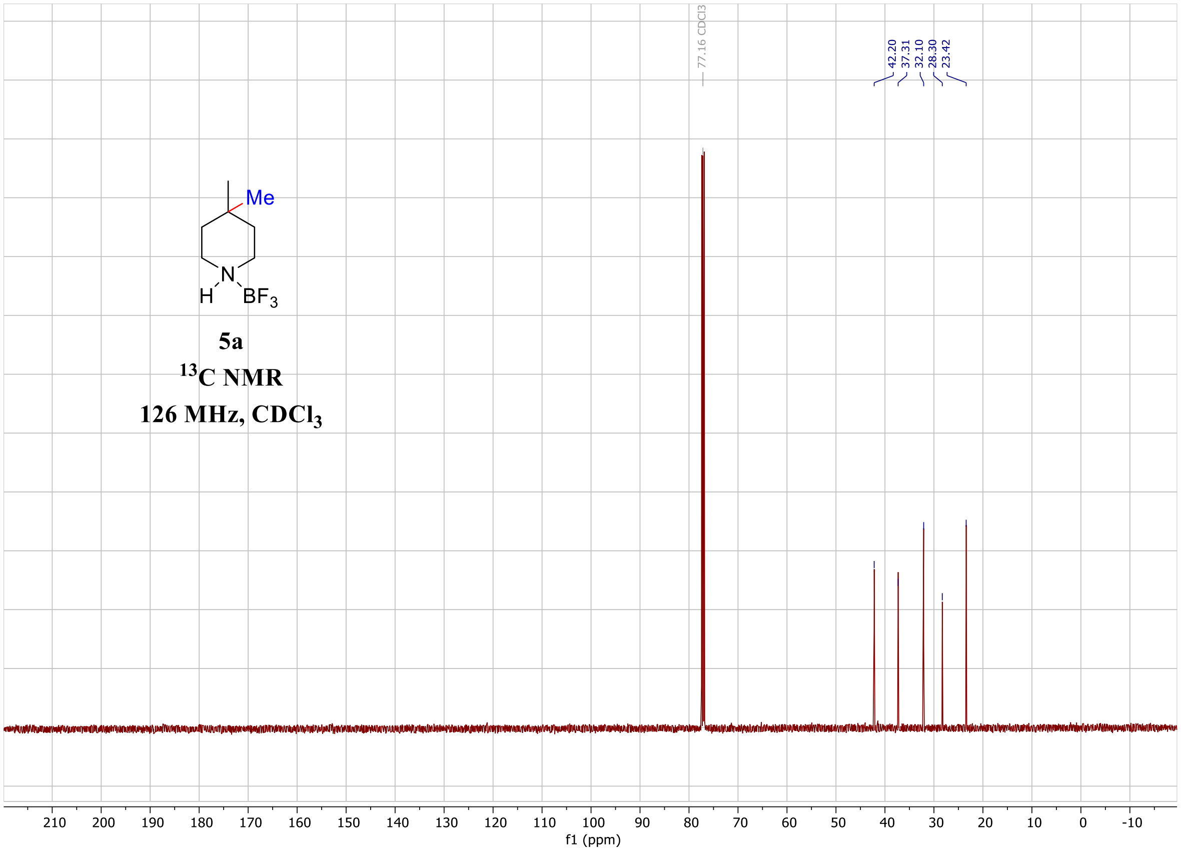
77.16 CDCl3
42.20
37.31
32.10
28.30
23.42
Me
N
H
BF3
5a
13C NMR
126 MHz, CDCl3
210 200 190 180 170 160 150 140 130 120 110 100 90 80 70 60 50 40 30 20 10 0 -10
f1 (ppm)

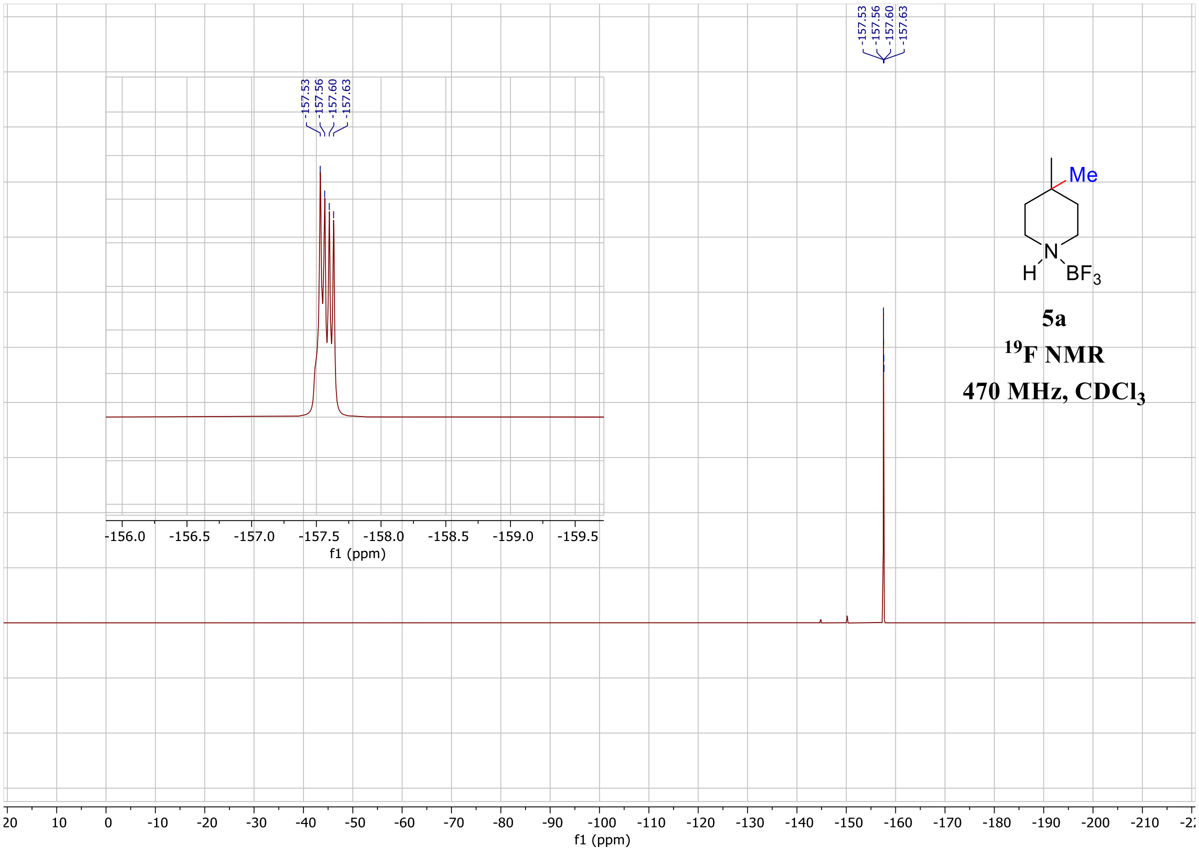
-157.53
-157.56
-157.60
-157.63
-156.0 -156.5 -157.0 -157.5 -158.0 -158.5 -159.0 -159.5
f1 (ppm)
Me
N
H
BF3
5a
19F NMR
470 MHz, CDCl3
20 10 0 -10 -20 -30 -40 -50 -60 -70 -80 -90 -100 -110 -120 -130 -140 -150 -160 -170 -180 -190 -200 -210
f1 (ppm)

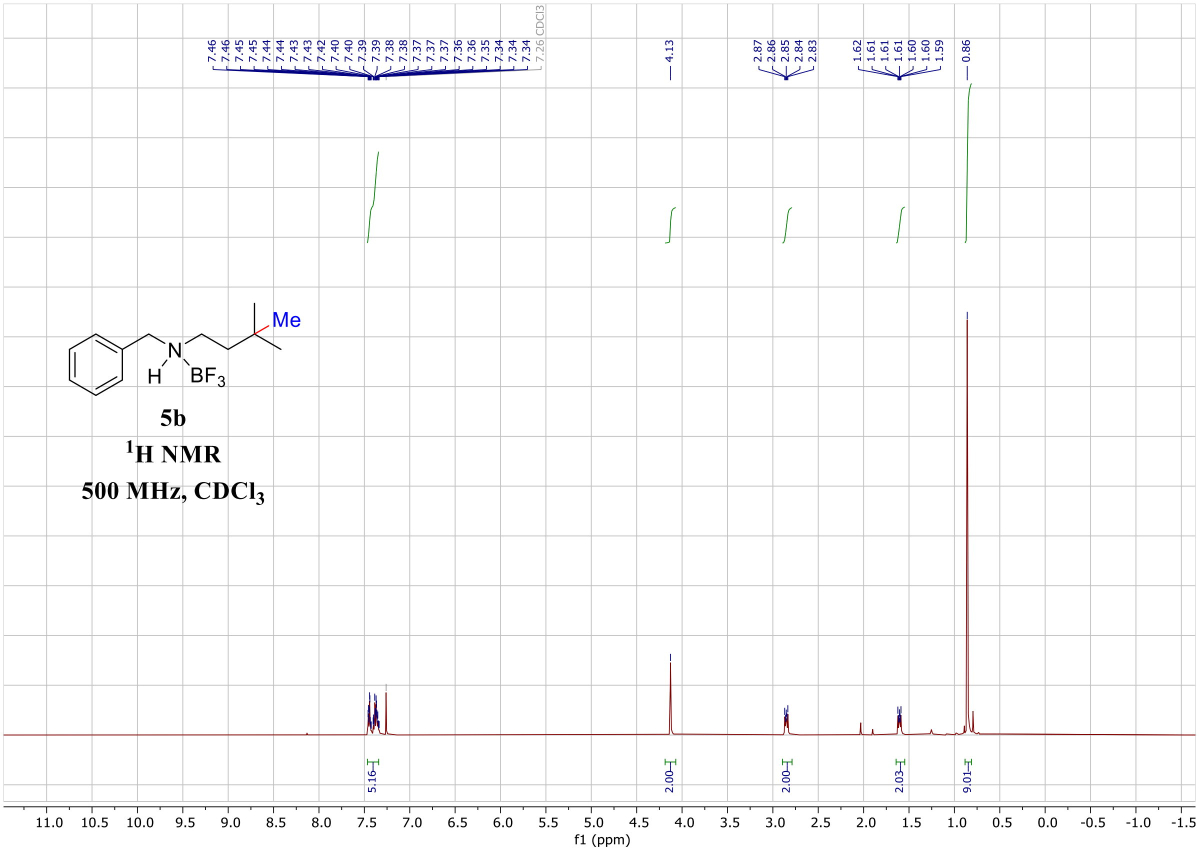
7.46
7.46
7.45
7.45
7.44
7.44
7.43
7.43
7.42
7.40
7.40
7.39
7.39
7.38
7.38
7.37
7.37
7.37
7.36
7.36
7.35
7.34
7.34
7.34
7.26 CDCl3
4.13
2.87
2.86
2.85
2.84
2.83
1.62
1.61
1.61
1.61
1.60
1.60
1.59
0.86
Me
N
H
BF3
5b
1H NMR
500 MHz, CDCl3
5.16
2.00
2.00
2.03
9.01
11.0 10.5 10.0 9.5 9.0 8.5 8.0 7.5 7.0 6.5 6.0 5.5 5.0 4.5 4.0 3.5 3.0 2.5 2.0 1.5 1.0 0.5 0.0 -0.5 -1.0 -1.5
f1 (ppm)

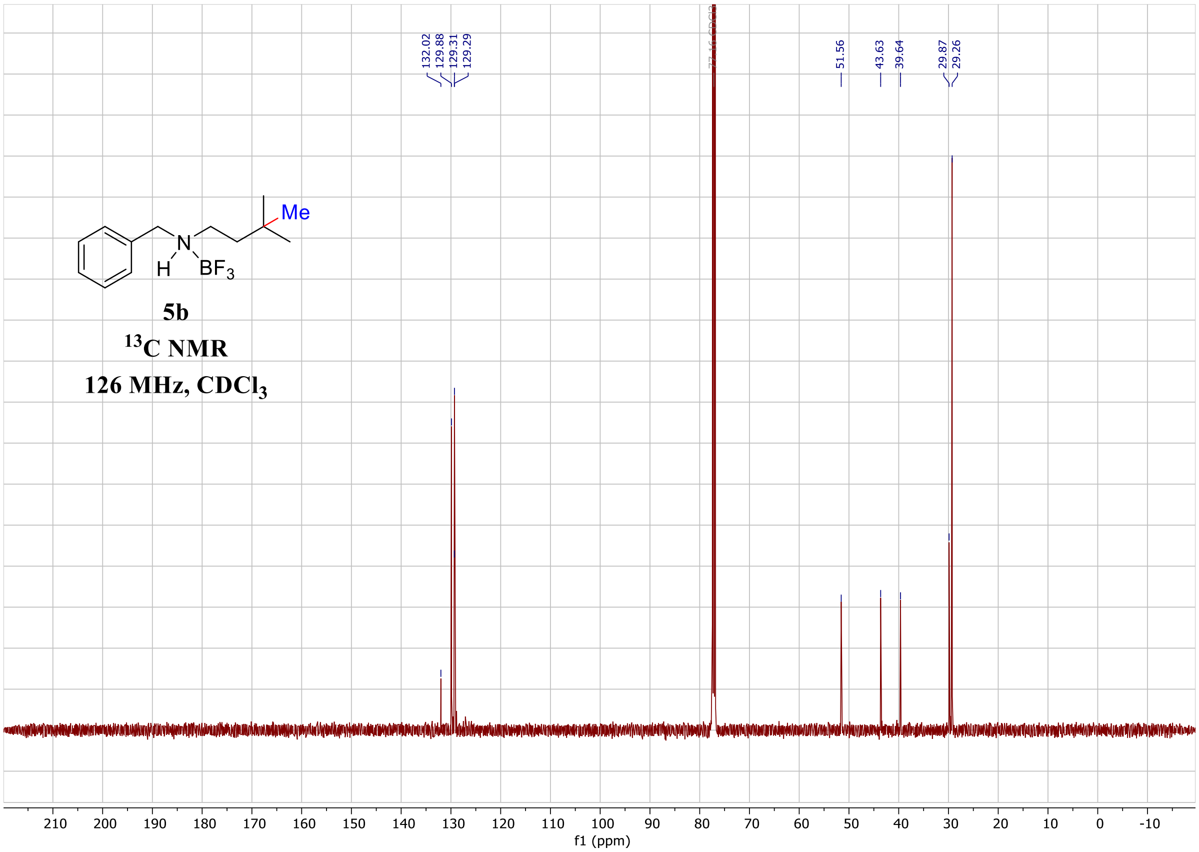
132.02
129.88
129.31
129.29
51.56
43.63
39.64
29.87
29.26
Me
N
H
BF3
5b
13C NMR
126 MHz, CDCl3
210 200 190 180 170 160 150 140 130 120 110 100 90 80 70 60 50 40 30 20 10 0 -10
f1 (ppm)

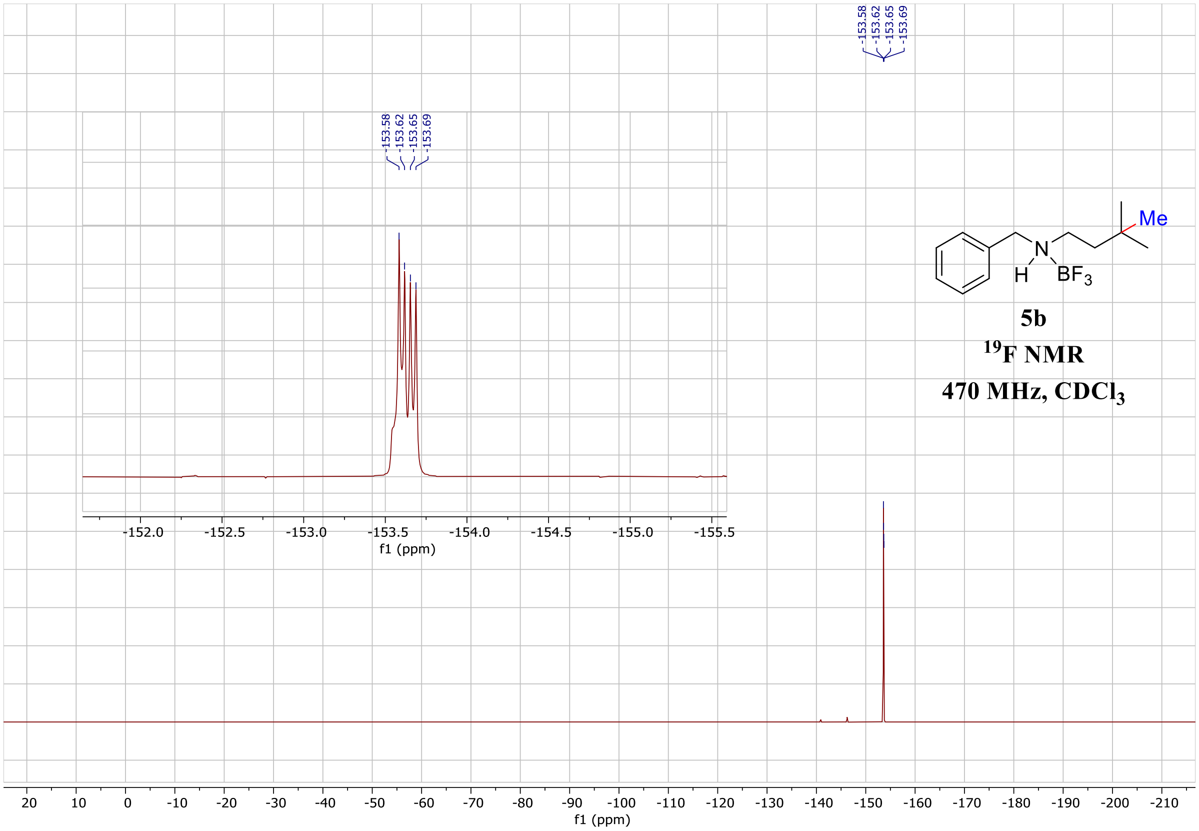
5b
^{19}F NMR
470 MHz, $CDCl_3$
f1 (ppm)

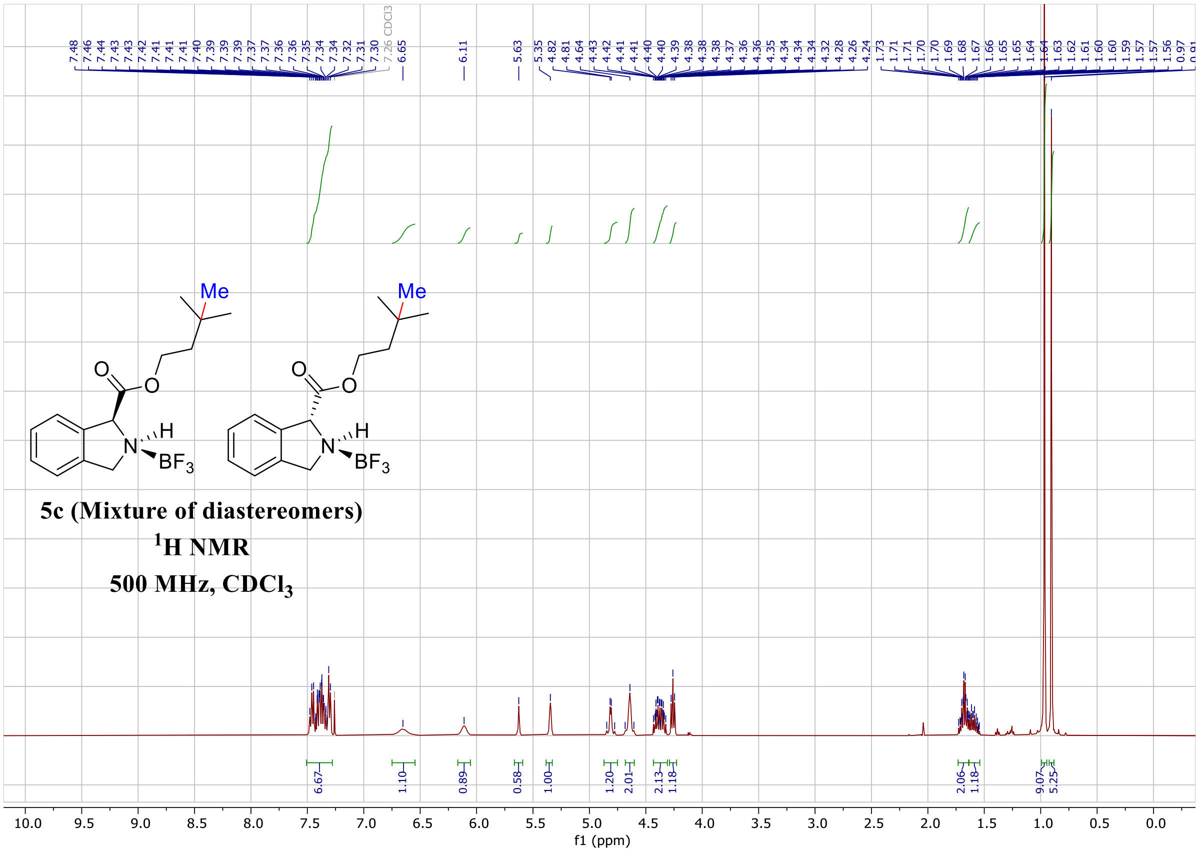
5c (Mixture of diastereomers)
^{1}H NMR
500 MHz, $CDCl_3$
f1 (ppm)

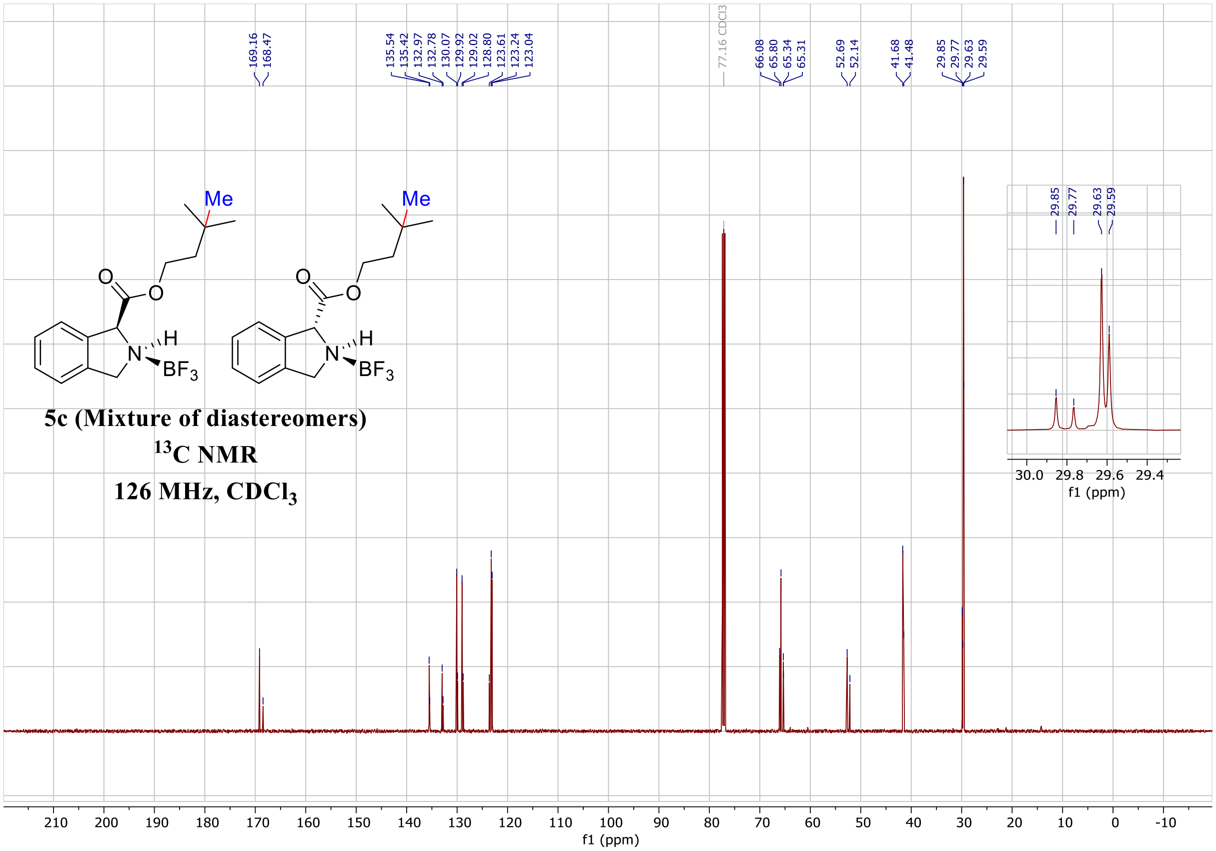

5c (Mixture of diastereomers)
^{13}C NMR
126 MHz, CDCl$_3$
f1 (ppm)

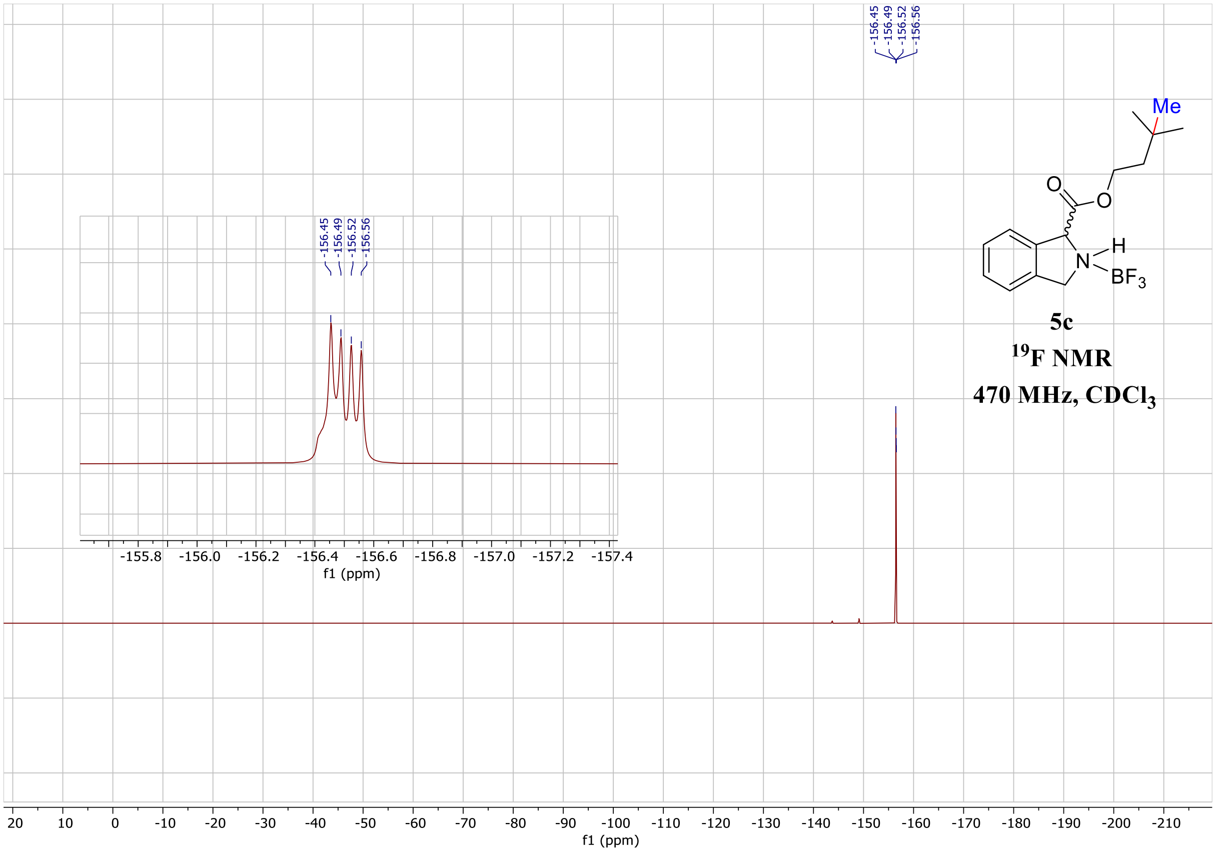

5c
^{19}F NMR
470 MHz, CDCl$_3$
f1 (ppm)

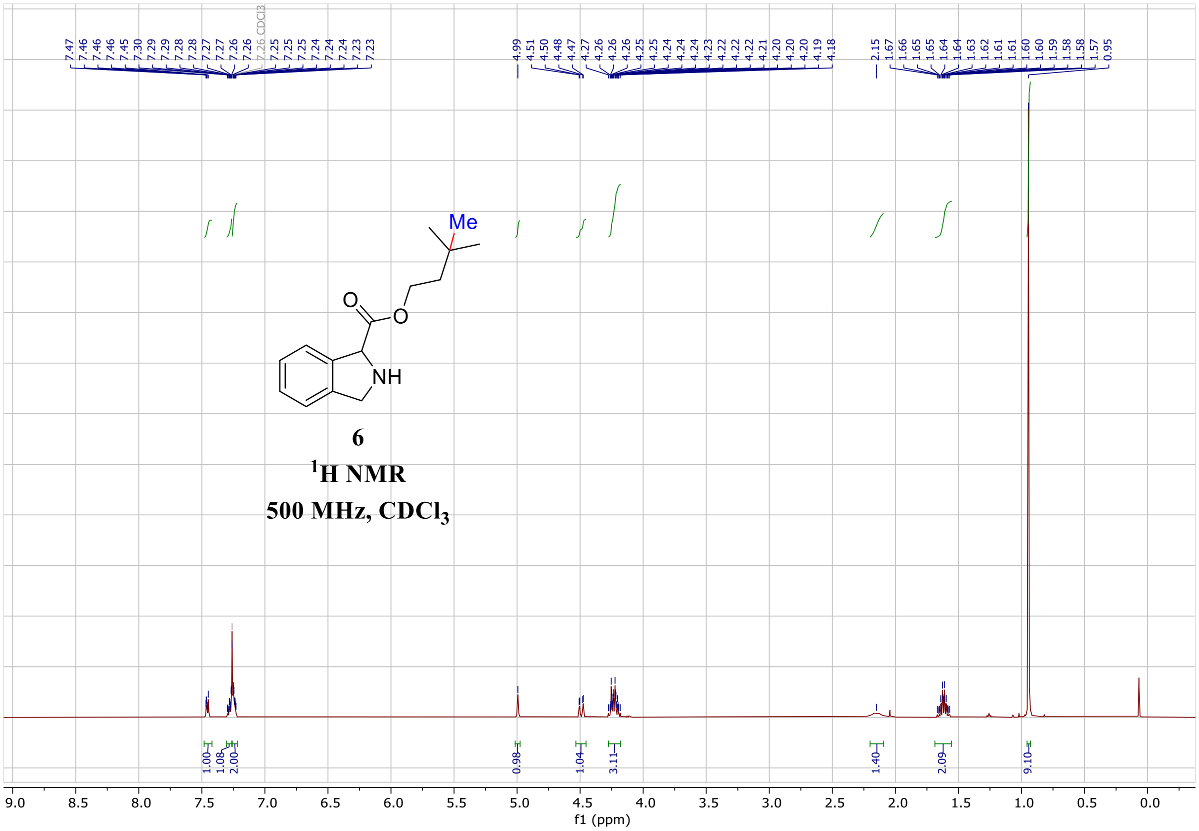
Me
O
O
NH
6
1H NMR
500 MHz, CDCl3
f1 (ppm)

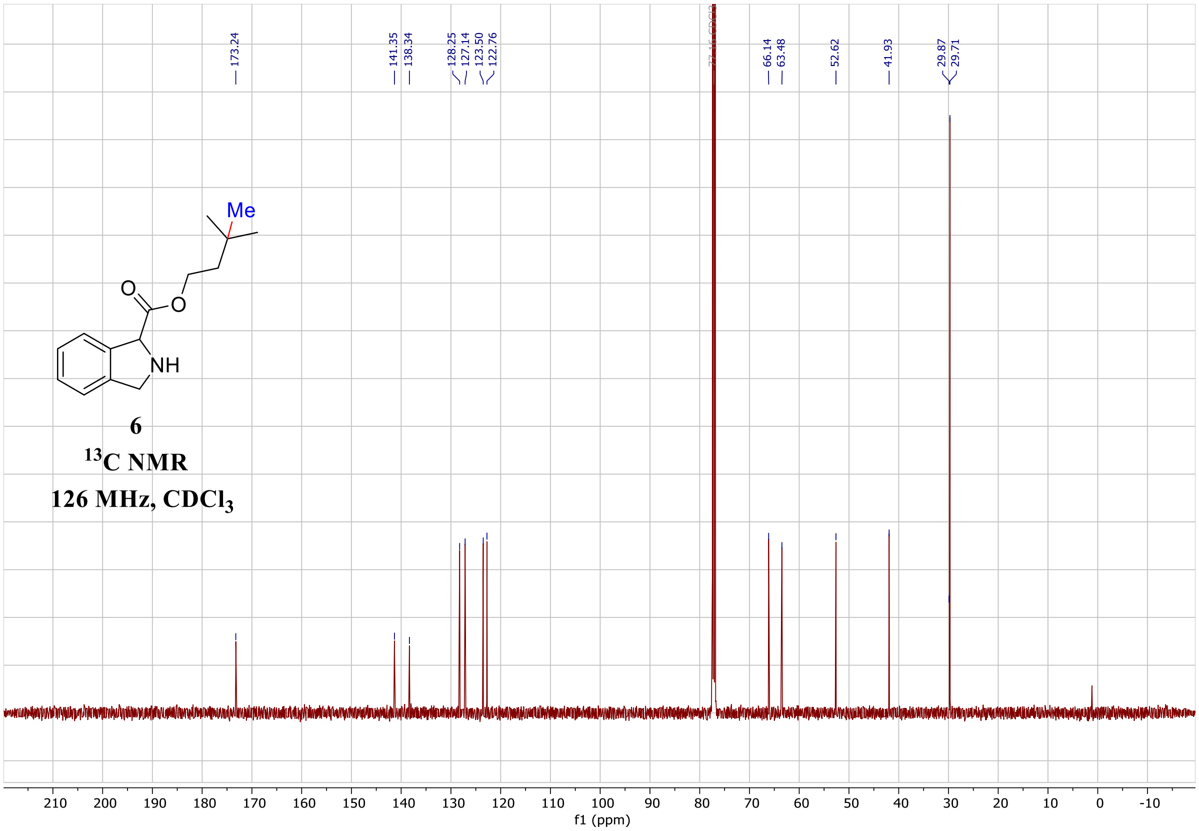
173.24
141.35
138.34
128.25
127.14
123.50
122.76
66.14
63.48
52.62
41.93
29.87
29.71
Me
O
O
NH
6
13C NMR
126 MHz, CDCl3
f1 (ppm)

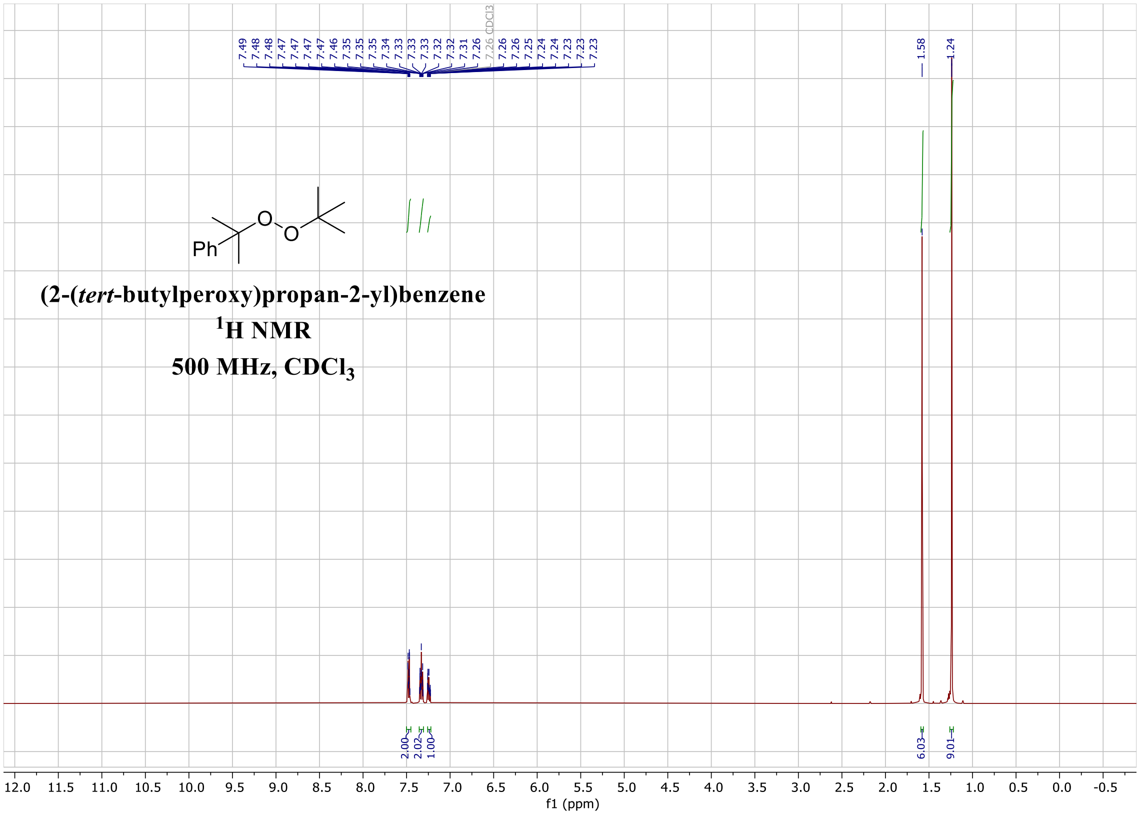
(2-(*tert*-butylperoxy)propan-2-yl)benzene
^{1}H NMR
500 MHz, $CDCl_3$
7.49
7.48
7.48
7.47
7.47
7.47
7.46
7.35
7.35
7.35
7.34
7.33
7.33
7.33
7.32
7.32
7.31
7.26
7.26 CDCl3
7.26
7.26
7.25
7.24
7.24
7.23
7.23
7.23
7.23
1.58
1.24
2.00
2.02
1.00
6.03
9.01
f1 (ppm)

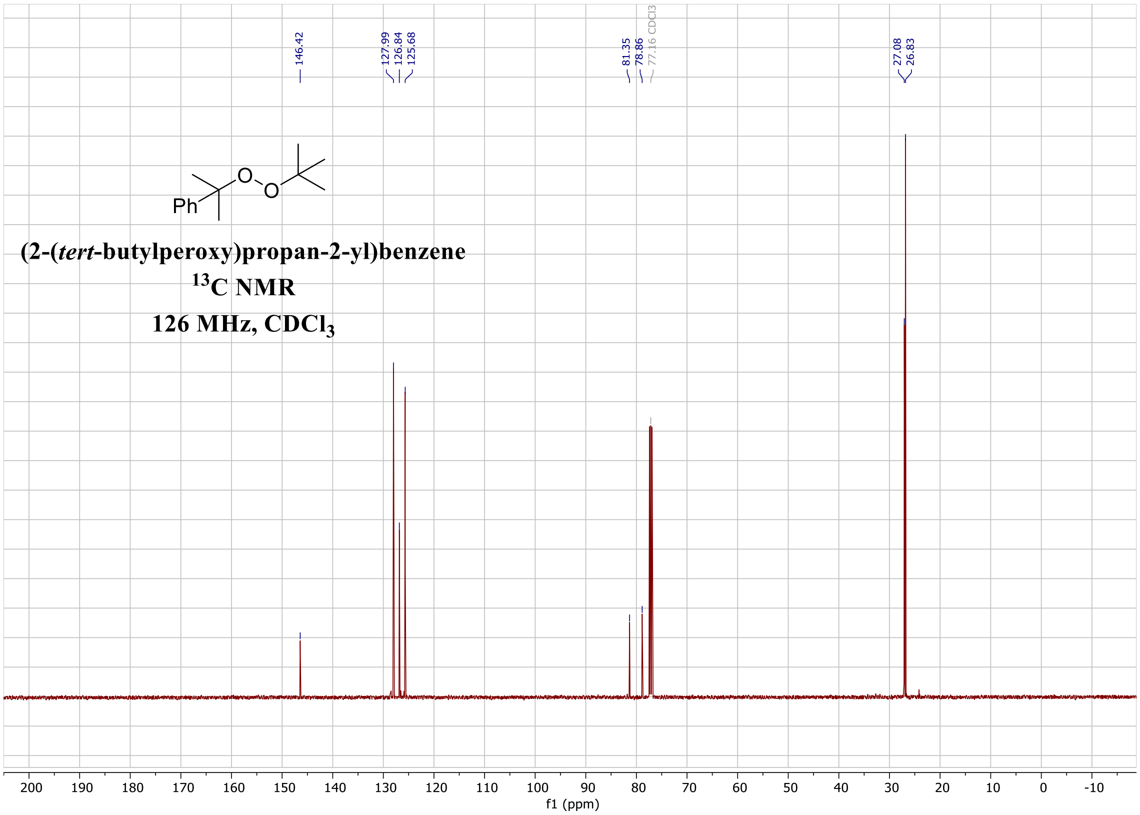
(2-(*tert*-butylperoxy)propan-2-yl)benzene
^{13}C NMR
126 MHz, $CDCl_3$
146.42
127.99
126.84
125.68
81.35
78.86
77.16 CDCl3
27.08
26.83
f1 (ppm)

Supplementary References

1. Oyama, R. & Abe, M. Reactivity and Product Analysis of a Pair of Cumyloxyl and *tert*-Butoxyl Radicals Generated in Photolysis of *tert*-Butyl Cumyl Peroxide. *J. Org. Chem.* **85**, 8627–8638 (2020).

2. Fawcett, A., Keller, M. J., Herrera, Z. & Hartwig, J. F. Site Selective Chlorination of C(sp^3)−H Bonds Suitable for Late-Stage Functionalization. *Angew. Chem. Int. Ed.* **60**, 8276−8283 (2021).

3. Mukherjee, S., Maji, B., Tlahuext-Aca, A. and Glorius, F. Visible-Light-Promoted Activation of Unactivated C(sp^3)−H Bonds and Their Selective Trifluoromethylthiolation. *J. Am. Chem. Soc.* **138**, 16200−16203 (2016).

4. Wei, Q., Ma, Y., Li, L., Liu, Q., Liu, Z. & Liu, G. Synthesis of Quaternary α-Fluorinated α-Amino Acid Derivatives via Coordinating Cu(II) Catalytic α-C(sp^3)−H Direct Fluorination. *Org. Lett.* **20**, 7100−7103 (2018).

5. Wang, Y., Li, G.-X., Yang, G., He, G. & Chen, G. A Visible-Light-Promoted Radical Reaction System for Azidation and Halogenation of Tertiary Aliphatic C−H Bonds. *Chem. Sci.* **7**, 2679−2683 (2016).

6. Barber, T., Argent, S. P. & Ball, L. T. Expanding Ligand Space: Preparation, Characterization, and Synthetic Applications of Air-Stable, Odorless Di-tert-alkylphosphine Surrogates. *ACS Catal.* **10**, 5454−5461 (2020).

7. Zhang, X., Guo, Shuo. & Tang, P. Transition-metal Free Oxidative Aliphatic C–H Fluorination. *Org. Chem. Front.* **2**, 806–810 (2015).

8. Howell, J. M., Feng, K., Clark, J. R., Trzepkowski, L. J. & White, M. C. Remote Oxidation of Aliphatic C–H Bonds in Nitrogen-Containing Molecules. *J. Am. Chem. Soc.* **137**, 14590–14593 (2015).

9. Yates, M. H., Kallman, N. J., Ley, C. P. & Wei, J. N. Development of an Acyl Sulfonamide Anti-Proliferative Agent, LY573636·Na[‡]. *Org. Process Res. Dev.* **13**, 255–262 (2009).

10. Vasilopoulos, A., Krska, S. W. & Stahl, S. S. C(sp^3)−H Methylation Enabled by Peroxide Photosensitization and Ni-mediated Radical Coupling. *Science* **372**, 398−403 (2021).

11. Dieter, R. K. & Watson, R. Synthesis of (±)-Isoretronecanol, (±)-Curassanecine, (±)-Heliotridane, (±)-Tashiromine and (±)-5-Epitashiromine via α-(*N*-Carbamoyl)alkylcuprate Chemistry. *Tetrahedron Lett.* **43**, 7725−7728 (2002).

12. Messa, F., Paparella, A. N., Veselý, D., Krajčovič, J., Papadia, P., Perrone, S. & Salomone, A. Gas-Free Amino- and Alkoxycarbonylation of Aryl Iodides in a Bioinspired Deep Eutectic Solvent with $Mo(CO)_6$ as a Safe CO Source. *Eur. J. Org. Chem.* **26**, DOI: e202300309 (2023).

13. Zhou, Z. H., Li, C.-K., Zhou, S.-F., Shoberu, A. & Zou, J.-P. Copper-catalyzed Methylation of 1,3-Diketones with *tert*-Butyl Peroxybenzoate. *Tetrahedron* **73**, 2740−2746 (2017)**.**

14. Guo, S., Wang, Q., Jiang, Y. & Yu, J.-T. *tert*-Butyl Peroxybenzoate-Promoted α-Methylation of 1,3-Dicarbonyl Compounds. *J. Org. Chem.* **79**, 11285–11289 (2014).

15. Shen, T., Li, Y.-L., Ye. K.-Y. & Lambert, T. H. Electrophotocatalytic Oxygenation of Multiple Adjacent C–H Bonds *Nature* **614**, 275–280 (2023).

16. Underiner, T. L. & Goering, H. L. Preparation and Assignment of Configuration of Cis- and Trans-2,3,4,4a,5,6-hexanhydro-2-naphthalenol *J. Org. Chem.* **52**, 897–900 (1987).

17. Liu, M., Qiu, Z., Tan, L., Rashid, R. T., Chu, S., Cen, Y., Luo, Z., Khaliulin, R. Z., Mi, Z. & Li, C.-J. Photocatalytic Methylation of Nonactivated sp^3 and sp^2 C–H Bonds Using Methanol on GaN. *ACS Catal*. **11**, 6248−6253 (2020).
